# The osteology of *Periptychus carinidens*: A robust, ungulate-like placental mammal (Mammalia: Periptychidae) from the Paleocene of North America

**DOI:** 10.1371/journal.pone.0200132

**Published:** 2018-07-18

**Authors:** Sarah L. Shelley, Thomas E. Williamson, Stephen L. Brusatte

**Affiliations:** 1 School of GeoSciences, University of Edinburgh, Edinburgh, Scotland, United Kingdom; 2 New Mexico Museum of Natural History and Science, Albuquerque, New Mexico, United States of America; Royal Belgian Institute of Natural Sciences, BELGIUM

## Abstract

*Periptychus* is the archetypal genus of Periptychidae, a clade of prolific Paleocene ‘condylarth’ mammals from North America that were among the first placental mammals to radiate after the end-Cretaceous extinction, remarkable for their distinctive dental anatomy. A comprehensive understanding of the anatomy of *Periptychus* has been hindered by a lack of cranial and postcranial material and only cursory description of existing material. We comprehensively describe the cranial, dental and postcranial anatomy of *Periptychus carinidens* based on new fossil material from the early Paleocene (Torrejonian) of New Mexico, USA. The cranial anatomy of *Periptychus* is broadly concurrent with the inferred plesiomorphic eutherian condition, albeit more robust in overall construction. The rostrum is moderately elongate with no constriction, the facial region is broad, and the braincase is small with a well-exposed mastoid on the posterolateral corner and tall sagittal and nuchal crests. The dentition of *Periptychus* is characterized by strongly crenulated enamel, enlarged upper and lower premolars with a tall centralised paracone/protoconid. The postcranial skeleton of *Periptychus* is that of a robust, medium-sized (~20 Kg) stout-limbed animal that was incipiently mediportal and adopted a plantigrade stance. The structure of the fore- and hindlimb of *Periptychus* corresponds to that of a typically terrestrial mammal, while morphological features of the forelimb such as the low tubercles of the humerus, long and prominent deltopectoral crest, pronounced medial epicondyle, and hemispherical capitulum indicate some scansorial and/or fossorial ability. Most striking is the strongly dorsoplantarly compressed astragalus of *Periptychus*, which in combination with the distal crus and calcaneal morphology indicates a moderately mobile cruropedal joint. The anatomy of *Periptychus* is unique and lacks any extant analogue; it combines a basic early placental body plan with numerous unique specializations in its dental, cranial and postcranial anatomy that exemplify the ability of mammals to adapt and evolve following catastrophic environmental upheaval.

## Introduction

The diversification of mammals following the end-Cretaceous mass extinction was a critical event in evolutionary history. The proliferation of eutherian mammals—placentals that give birth to live to well-developed young, and their closest fossil relatives—during this time produced numerous clades of ‘archaic’ mammals which did not survive beyond the Paleogene and whose phylogenetic affinities with extant mammals remain contentious, but which promise to help disentangle the early history of mammals if their anatomy and relationships can be better understood. One such group are the Periptychidae, a clade of morphologically robust, ungulate-like, ‘condylarths’ known from North America. The Periptychidae were among the placental mammals to appear after the end-Cretaceous extinction. Thus, these species are key for understanding how mammals were affected by the extinction and blossomed afterwards. *Periptychus* is the archetypal genus of Periptychidae and easily recognised by its distinctive teeth, fossils of which are common in the Paleocene deposits of western North America.

*Periptychus* was first described nearly 140 years ago; following his initial findings, Cope ([1], p.801) wrote, “Its discovery I consider to be an important event in the history of palaeontological science”. Cope did little expand on this statement much beyond a brief description of the material at hand and by hypothesising on the unusual appearance of the animal. Subsequent authors have supplemented our knowledge of *Periptychus*, but a detailed description of this enigmatic taxon has been left wanting. Little is currently known about the cranial and, particularly, the postcranial anatomy of this taxon, which has inhibited a detailed understanding of its paleobiology and evolution. An overview of the literature reveals a surprisingly long and convoluted taxonomic history for *Periptychus* but little discussion regarding its paleobiology; in fleeting sentences it has been described as a medium-sized, terrestrial/generalist, ‘archaic-ungulate’, showing dental specialisations towards an herbivorous to durophagus diet [2,3] and with some superficial postcranial attributes similar to extant tayassuids [4], vombatids, and *Orycteropus* [5]. These descriptions have not been recently updated to place them in context of our modern understanding of mammalian anatomy and evolution.

The necessity of a revision of *Periptychus* is also buoyed by the discovery of a wealth of new fossil material from the lower Paleocene (Torrejonian) deposits in the San Juan Basin of New Mexico, much of it collected over the past two decades by teams led by TEW. By combining these new fossils with restudy of historic collections, we here present a comprehensive anatomical re-description of *Periptychus carinidens*.

### Institutional Abbreviations

AMNH American Museum of Natural History, New York, NY, USA;

MNHN Muséum national d’Histoire naturelle, Paris, Collection de Paléontologie, Paris, France;

NMMNH New Mexico Museum of Natural History and Science, Albuquerque, NM, USA;

SPSM St Paul Science Museum, MN, USA;

TMM Texas Memorial Museum, Austin, TX, USA;

UNM Department of Geology, University of New Mexico, Albuquerque, NM, USA;

USNM National Museum of Natural History, Washington, D. C., USA.

### Historical background

The taxonomy and systematics of *Periptychus*, the Periptychidae and ‘Condylarthra’ have a long and complex history. ‘Condylarths’ have long been recognised as the ancestral stock from which ungulate mammals, including extant artiodactyls and perissodactyls, arose [3,6]. However, over time, ‘Condylarthra’ has come to include an assortment of ungulate-grade Paleogene mammals which do not appear to form a natural group. Periptychidae is an anatomically distinctive ‘condylarth’ subgroup; they are seemingly easy to recognise and were pivotal in the establishment of ‘Condylarthra’.

The historical literature pertaining to *Periptychus* and the Periptychidae is convoluted, which is perhaps surprising given how seemingly distinctive periptychids are. *Periptychus* first appears in Torrejonian aged deposits and is immediately recognisable by its distinctive dentition with enlarged premolars and highly crenulated enamel. *Carsioptychus*, a medium-sized periptychid from the older Puercan deposits of North America, is morphologically similar to *Periptychus*. The relative abundance of dental specimens has allowed workers to observe subtle variations in the dental morphology of these taxa, which some workers have asserted are important enough to warrant species-level recognition (e.g. [5,7–10]). Because of the dental similarities between *Periptychus* and *Carsioptychus* and morphological variation within each genus, there has been, and continues to be, debate over whether *Carsioptychus* should be regarded as a genus distinct from *Periptychus*, and over the validity of the numerous species referred to one genus or the other.

*Periptychus* was first diagnosed by Cope based on a dentary fragment [11]. Not realising that the specimen represented a juvenile individual, Cope named the taxon *Periptychus carinidens* and assigned it to Creodonta. As an aside here, Cope erroneously named *Periptychus carinidens* as a new genus and species twice, in two separate publications [11,12]. In the same year, Cope also described ‘*Catathlaeus rhabdodon’*, based on a partial maxilla preserving the P1-M3 [12]. ‘*Catathlaeus rhabdodon’* was provisionally allied with Phenacodontidae given its highly bunodont molar dentition. In 1882, Cope revised ‘Condylarthra’ with the discovery of *Periptychus* postcrania and several new periptychids from the ‘Puerco beds’ of New Mexico [13]. The new associated dental and postcranial material allowed Cope to recognise his error and synonymize ‘*Catathlaeus rhabdodon’* to *Periptychus* as ‘*Periptychus rhabdodon’*. Cope further observed that the tarsal structure of *Periptychus* bore very little resemblance to that of *Phenacodus*, so he erected Periptychidae as a family within ‘Condylarthra’, which he considered a sub-order of Taxeopoda at the time [13].

Cope advocated that ‘*Periptychus rhabdodon’* represents a separate, larger and more robust species than *Periptychus carinidens* [4]. Matthew noted that intermediate forms exist between *P*. *carinidens* and ‘*P*. *rhabdodon’* and that had Cope not misdiagnosed *P*. *carinidens* based on deciduous teeth, the issue over the validity of ‘*P*. *rhabdodon*’ would probably be a moot point [5]. Nevertheless, Matthew retained ‘*P*. *rhabdodon’* and *P*. *carinidens* as separate valid species. In 1959, Simpson measured and compared an assortment of 37 *Periptychus* dental specimens and found no bimodality in tooth size, so he formally synonymized ‘*P*. *rhabdodon’* with *P*. *carinidens* and noted that the size variation between the different morphotypes could represent intraspecific variation [8].

‘*Periptychus superstes’*, a Tiffanian species of *Periptychus*, was first identified and named by Matthew, but Matthew died before publishing his work. Simpson [14] credited Matthew with the diagnosis of this taxon; consequently, the descriptions in Simpson [14] and Matthew’s posthumous monograph [5] are effectively the same. Matthew [5,14], asserted the validity of ‘*P*. *superstes’* based on the p5 being proportionally smaller relative to the molar series (in comparison to *P*. *carinidens*) and the m3 possessing a more elongate talonid heel. However, Matthew [5] lowered ‘*P*. *superstes’* to subspecies rank within ‘*P*. *rhabdodon’*. Matthew commented that ‘*P*. *superstes’* was likely a progressive form of ‘*P*. *rhabdodon’*, but concluded that it was not known well enough to distinguish it as a separate species [5].

In 1967, Wilson briefly described three *Periptychus* specimens from Black Peaks Formation, exposed at Tornillo Flat in Big Bend National Park, Texas, which he assigned to *Periptychus carinidens* [15]. In 1974, Schiebout provided a more detailed description of the specimens and tentatively referred five new Tiffanian specimens to ‘*Periptychus superstes’* (TMM 41274–1; 40147–4; 40147–17; 40537–59; 41367–8) [16]. Standhardt [17] subsequently also referred TMM 40147–1 to ‘*Periptychus superstes’* (also from Tornillo Flat). Schiebout highlighted similarities between the Big Bend specimens and ‘*P*. *superstes’*, but also concluded that not enough was known about the size variability within *Periptychus carinidens* and ‘*P*. *superstes’* to definitively assign the Big Bend specimens to a species [16].

Williamson [10] found the range of size variation of the first lower molar between *P*. *carinidens* to overlap with specimens of ‘*P*. *superstes’* from the San Juan Basin, with the exception of two particularly large specimens from Big Bend (TMM 40147–17 and 40537–59), and consequently synonymized *‘P*. *superstes’* with *P*. *carinidens*. We agree with Williamson [10] in that the morphological features described by Matthew [5,14] for distinguishing ‘*P*. *superstes’* are present in specimens of *P*. *carinidens*, and thus do not warrant species distinction. However, we do recognize that there is variation in proportions between the premolar and molar dentition in *Periptychus* specimens, which may mark an important ecological, temporal, and/or geographical transition.

*‘Periptychus gilmorei’* was first described by Gazin [18] from the North Horn Formation, Dragon Canyon, Emery County, Utah. Gazin proposed ‘*P*. *gilmorei’* as an intermediate species between *Carsioptychus coarctatus* and *Periptychus carinidens* based on a combination of dental characters present in ‘*P*. *gilmorei’* that are characteristic of both *C*. *coarctatus* or *P*. *carinidens*. The Dragon Fauna was initially thought to represent a distinct North American Land Mammal Age (NALMA; the Dragonian) between the Puercan and Torrejonian NALMAs [19]; however, magnetostratigraphy and correlation of the North Horn Formation with the Nacimiento Formation has shown the Dragonian to be equivalent to the early Torrejonian [20–23]. An early Torrejonian age for ‘*P*. *gilmorei’* supports the hypothesis that it represents a transitional form between *Carsioptychus* and the bulk of *Periptychus* specimens from the Nacimiento Formation, in addition to its purported intermediate morphology. The upper premolars of ‘*P*. *gilmorei’* exhibit a crescentic lingual shoulder as in *P*. *carinidens*; however, the lingual shoulder is somewhat anteroposteriorly constricted, bearing some resemblance to *C*. *coarctatus*. Gazin [18] also noted that the upper molar dentition of ‘*P*. *gilmorei’* resembles *C*. *coarctatus* in being transversely expanded with a moderately elongate lingual slope. Williamson [10] found that the dentition of ‘*P*. *gilmorei’* falls within the size and morphological range exhibited by *P*. *carinidens* and therefore concluded that ‘*P*. *gilmorei’* is a junior synonym of *P*. *carinidens*. We tentatively agree with Williamson [10], given that specimens of *P*. *carinidens* from the San Juan Basin exhibit a range of morphologies which fit the description for ‘*P*. *gilmorei’* and cover the range of difference between ‘*P*. *gilmorei’* and *P*. *carinidens*. We do not think that intermediate dental morphology of ‘*P*. *gilmorei’* warrants specific rank, but it does highlight a subtle morphological shift within *P*. *carinidens* with an apparent prevalence of a *‘P*. *gilmorei’* morphotype in the North Horn Formation, Utah.

The taxonomy for *Carsioptychus* is equally complicated. *Carsioptychus coarctatus* was first described by Cope [24] as a species of *Periptychus* (= *Periptychus coarctatus*). Simpson [14], citing unpublished notes by Matthew, considered ‘*Periptychus coarctatus’* more distinct from *Periptychus carinidens* than the other purported *Periptychus* species, and thus established the subgenus ‘*Plagioptychus’* within *Periptychus* for ‘*Periptychus coarctatus’*. In the same publication, Simpson formally raised ‘*Plagioptychus’* to genus level. Unfortunately, the genus ‘*Plagioptychus’* was occupied so Simpson proposed the new generic name *Carsioptychus*, into which he transferred *Carsioptychus coarctatus* (= *Periptychus coarctatus*) and ‘*Carsioptychus matthewi’* (= *Plagioptychus matthewi*) [25]. Van Valen [9] proposed *Carsioptychus* be treated as a subgenus of *Periptychus* (as previously suggested by Matthew [5]) but provided no further explanation for his decision. Archibald *et al*. [23] asserted that there are enough morphological differences in premolar cusp development and occlusal tooth profile between *Periptychus* and *Carsioptychus* to warrant generic distinction. Given the similarities between *Carsioptychus* and *Periptychus* relative to other periptychids, and the facts that the two are known from the same area and the former is older than the latter, it is possible that *Carsioptychus* is directly ancestral to *Periptychus* [5,23]. Williamson [10] noted the usefulness of retaining *Periptychus* and *Carsioptychus* as separate genera given that the first occurrence of *Periptychus* is used to define the base of the Torrejonian, but was doubtful over whether there is enough morphological dissimilarity between the taxa to warrant generic distinction.

*‘Periptychus brabensis’* was first formally described by Osborn & Earle [26] and credited to Cope (no date). However, subsequent authors have synonymized specimens referred to ‘*P*. *brabensis’* to both *Periptychus carinidens* and *Carsioptychus coarctatus*. Osborn & Earle [26] described ‘*P*. *brabensis’* based on a mandible specimen (AMNH 849), not the type (AMNH 3782) (note that the type was never formally designated by Cope). They asserted that the upper premolars of ‘*P*. *brabensis’* are buccolingually wider than the upper molars, and the upper molar conules are seemingly absent, and noted that this species exhibits some intermediary form between *‘C*. *coarctatus’* and *‘P*. *rhabdodon’*. In 1888, Cope referred twenty individual specimens from the Nacimiento Formation, San Juan Basin, New Mexico to ‘*P*. *brabensis’*. Cope noted the similarities between ‘*P*. *brabensis’* and ‘*C*. *coarctatus’* but retained ‘*P*. *brabensis’* as a separate species [27]. Matthew [5] found that AMNH 849 is a juvenile specimen of *P*. *carinidens* preserving the deciduous dentition, and the morphology displayed by specimens referred to ‘*P*. *brabensis’* by Osborn & Earle [26] fall within the morphological range of *P*. *carinidens*. Matthew [5] also noted that specimens referred to *‘P*. *brabensis’* by Cope [27] exhibit morphologies which fall within the range of *Carsioptychus coarctatus*. Based on the morphology and Puercan age of the type, ‘*P*. *brabensis*’ is a synonym of *C*. *coarctatus*; however, Cope never formally designated the type specimen. Specimens assigned to ‘*P*. *brabensis*’ by Osborn & Earle [26], based on comparison to AMNH 849, are generally referable to *P*. *carinidens*.

*‘Plagioptychus (= Carsioptychus) matthewi’* was first described by Simpson [25] based on a partial dentary preserving p2-m3, recovered from the Nacimiento Formation, San Juan Basin, New Mexico. Simpson described *‘Plagioptychus matthewi’* as a larger and more derived species than *Carsioptychus coarctatus*, but failed to mention any unique characters not found in *C*. *coarctatus*. Van Valen [9] subsequently synonymized ‘*P*. *matthewi’* with *Carsioptychus coarctatus* without providing any justification (note that Van Valen also considered *Carsioptychus* a subgenus of *Periptychus*), although we note there are there no obvious morphological differences between the purported species. Williamson [10] plotted the log of the first lower molar area of *Periptychus carinidens* and *Carsioptychus coarctatus* (into which he included ‘*Carsioptychus matthewi’*). The results showed no statistical differences between first lower molar area of *Carsioptychus coarctatus* and ‘*Carsioptychus matthewi’*, further supporting the synonymy of ‘*Carsioptychus matthewi’* with *Carsioptychus coarctatus*.

*‘Carsioptychus hamaxitus’* was described by Gazin [28] based on a partial maxilla preserving M1-2 from the Wagonroad fauna, North Horn Formation, Dragon County, Utah. Based on two additional specimens from the same area, which preserve the M2-3 and m2-3, Gazin proposed ‘*Carsioptychus hamaxitus’* as a smaller variant of *Carsioptychus coarctatus* with some rudimentary *Periptychus* characteristics, namely a more developed premolar paraconid than *Carsioptychus coarctatus*. Williamson [10] found development of the premolar paraconid to be variable within specimens of *Carsioptychus coarctatus* from the Nacimiento Formation in the San Juan Basin, New Mexico, and the range in morphology overlapped with that of specimens of the Wagonroad fauna, justifying the synonymy of ‘*Carsioptychus hamaxitus’* with *Carsioptychus coarctatus*. Gazin [18] also named a new species of *Periptychus*, ‘*P*. *gilmorei’* (see above), from the Dragon Fauna of the North Horn Formation and other authors have noted that other species from the North Horn Formation are distinct from their Torrejonian congeners [29]. Such findings raise the question of whether North Horn Formation faunas represent a geographically isolated population, which led to the prevalence of different morphotypes compared to their southern and northern counterparts.

To summarize the above discussion, and provide a guide to the reader: in this paper, we recognise *Periptychus carinidens* as a single valid species and agree with Williamson [10] regarding the synonymy of ‘*P*. *gilmorei’* and ‘*P*. *superstes’* with *Periptychus carinidens*, although we note that this conclusion is subject to change if new fossil material documents discrete variation between these different forms. We also choose to retain *Carsioptychus* at generic rank, as we recognize that there are distinct morphological differences with specimens of *Periptychus carinidens* and *Carsioptychus coarctatus* (outlined above and in more detail in the Diagnosis below). Retaining Puercan-aged *Carsioptychus* as a distinct genus-level taxon also provides useful information in determining character polarity within Periptychinae in phylogenetic analyses; its exclusion or assimilation with Torrejonian-aged *Periptychus* has produced erroneous results previously (see [30–32]). However, we note that within genera and species there are subtle changes in dental morphology and tooth proportions, and the differentiation of species based on single tooth measurements may not be the best protocol for distinguishing *Periptychus* from *Carsioptychus* given the large size variation exhibited by these taxa. Instead, the proportional size differences between the premolar and molar dentition better encapsulates the variation based on the descriptions by previous authors; these measurements require further investigation, which may lead future workers to modify the classification scheme we use here.

### Geological setting

*Periptychus* is known solely from the early Paleogene deposits of western North America, with fossil specimens recovered from Colorado, Montana, New Mexico, Texas, Utah and Wyoming. The Paleocene climate was warmer than present, with mean annual temperatures in the San Juan Basin of ~12° ± 4.4° C and mean annual precipitation amounts of ~1,100 mm [33–35]. Mean annual temperatures in mid-latitude continental interiors were warm, with average winter temperatures likely above freezing and a reduced latitudinal temperature gradient [36–41].

*Periptychus* is particularly well represented in the Nacimiento Formation in the San Juan Basin of New Mexico. The Nacimiento Formation contains one of the world’s best records of terrestrial vertebrate succession through the early Paleocene (~64.5 to ~61 million years ago) and includes the type faunas for the first two North American Land Mammal Ages of the Cenozoic: the Puercan (excluding Pu1) and Torrejonian [19,23,42–44] *Periptychus* is one of only a few genera to extend through the entire Torrejonian before going extinct in the Tiffanian, with a total genus duration of approximately three million years. It left no apparent descendants.

The Nacimiento Formation facies are primarily comprised of bentonitic mudstones intercalated with fluvial channel and crevasse sandstones, moderately to well-developed paleosols, and carbonaceous shale units representing a non-marine, fluvial to lacustrine depositional environment [10,42].

The floral and faunal composition of the Nacimiento Formation indicates a frost-free environment [45]. The flora was dominated by an array of angiosperms which were relatively diverse locally while remaining comparatively heterogeneous across the region [46]. Stable isotope analysis of carbon and oxygen indicate an ecosystem dominated by C_3_ vegetation [46]. The predominance of C_3_ vegetation was prevalent up until the Miocene and indicates a paleoenvironment where sunlight intensity was moderate, temperature was moderate, carbon dioxide concentration was relatively high and groundwater was abundant [47]. Isotopic analysis of the enamel of *Periptychus carinidens*, *Claenodon ferox*, *Mioclaenus subtrigonus* and *Tetraclaenodon puercensis* found high carbon values indicative of feeding in comparatively open and relatively drier habitats [46]. Based on these proxies, the paleoenvironment of the San Juan Basin during the early Paleocene can be inferred as being composed of areas with densely vegetated, closed canopy forest interspersed with more open expanses. The abundance of crocodile and turtle fossils is also indicative a warm, humid climate [38,39].

## Materials and methods

### Description and comparison

This study is based largely on new *Periptychus carinidens* specimens collected from the San Juan Basin in New Mexico, curated at the New Mexico Museum of Natural History and Science. Access to precise locality information is restricted to qualified researchers and land management personnel. These new specimens are the primary focus of this study, and allow for a comprehensive re-description of the cranial, dental and postcranial anatomy of *Periptychus carinidens*. Descriptions are supplemented with information from the collection held at the American Museum of Natural History in New York, USA.

Throughout the descriptive text, comparisons are made to numerous medium-sized Paleocene mammals, including other periptychid taxa, known from cranial, dental and postcranial specimens. These include, but are not restricted to: *Carsioptychus coarctatus*, *Ectoconus ditrigonus*, *Mithrandir gillianus*, *Arctocyon primaevus*, *Claenodon ferox*, *Protungulatum* sp., and *Pantolambda bathmodon*. We have observed the comparison taxa first-hand except for *Mithrandir gillianus*, for which a postcranial skeleton (NMMNH P-3083) was not available during the period of study.

*Carsioptychus* and *Ectoconus* are both medium-sized periptychids thought to be closely related to *Periptychus* [2,6,30]. *Ectoconus* is known from a near complete skeleton (AMNH 16500) and shares many morphological similarities with *Periptychus*. *Mithrandir gillianus* is the only small periptychid known from a partial skeleton (NMMNH P-3083), which was described by Rigby [48] (note that in [48] the specimen is referred to as *Gillisonchus* and referenced as UNM-B029). We include *Mithrandir* in our study to assess the morphological features of *Periptychus* associated with larger body size in relation to a smaller, relatively closely related taxon.

*Arctocyon primaevus* and *Claenodon ferox* are members of ‘Arctocyonidae’. The ‘arctocyonids’ are generally considered the ancestral stock from which other ‘condylarth’ groups arose [3], and closely related to the Periptychidae [9], although they have previously been allied with carnivorans due to homoplasic characters of their dentition [49,50]. The morphological similarities between *Arctocyon* and *Claenodon* have resulted in these two taxa often being considered synonymous with one another [3,10,51,52]. We include both *Arctocyon* and *Claenodon* in our comparisons with *Periptychus* as these taxa are seemingly morphologically distinct from each other, but of a similar size to *Periptychus*. This provides useful insight into the functional morphology and ecology of *Periptychus*. We note that future work is required to assess the validity of *Arctocyon* and *Claenodon* as separate genera.

*Protungulatum* is an ungulate-like eutherian mammal known from latest Cretaceous-Paleogene deposits of North America [53]. The phylogenetic position of *Protungulatum* remains contentious. It has previously been considered a basal member of ‘Arctocyonidae’ and plausible ancestor for Periptychidae [9]. More recent studies have excluded *Protungulatum* from ‘Arctocyonidae’ [6], considered it as the oldest undisputed species within crown Placentalia [54], and found it as a non-placental stem eutherian [55–57]. Regardless, of its phylogenetic affinities, *Protungulatum* provides a useful comparison to *Periptychus* and is often considered broadly representative of the primitive eutherian condition [54], albeit with some more ungulate-like features. At the very least, *Protungulatum* is a reasonably well-known taxon documenting a major shift in anatomical specializations between Cretaceous and Paleogene eutherians.

We also make detailed comparisons with *Pantolambda bathmodon*, a medium-sized pantodont also known from the Torrejonian of the San Juan Basin, New Mexico. Previous workers, namely Cope [58], Osborn [59] and Gregory [60], noted the remarkable postcranial similarities between *Periptychus* and *Pantolambda bathmodon*, and proposed a close relationship between these two taxa. The vastly disparate dental anatomy of these two taxa likely means that they are not closely related [5,61,62], but their cranial and postcranial similarities warrant full investigation in order to better understand their functional morphology and paleoecology.

Measurements are provided throughout the text and in tables (S1 Appendix). Measurements were made using digital callipers, in millimetres, to the nearest two decimal places. Digital measurements were taken with ImageJ 1.6.0 [63].

Dental notation follows that of McKenna [64], more recently used by Wible *et al*. [55,56] and O’Leary *et al*. [54], whereby the first and second premolars are P1/p1 and P2/p2, respectively; P3/p3 in basal eutherians is considered to be a retained deciduous tooth (it is not present in *Periptychus*); the penultimate molar is referred to as P4/p4; and the ultimate premolar is referred to as P5/p5. Tooth nomenclature follows the standard eutherian terminology outlined by Szalay [65] where applicable or otherwise specified. Osteological and myological nomenclature and directional references are draw from a range of studies: generally we follow the terminology and protocols outlined in Miller’s Anatomy of the Dog [66] with reference to studies on numerous other Paleogene eutherians [3,5,23,48,52,67–72]. Specimens were imaged in the standard anatomical views unless otherwise specified. Some specimens were dusted with magnesium oxide or ammonium chloride prior to imaging to enhance surface details and contrast (this was not possible for all specimens due to collection restrictions).

Body mass was estimated for *Periptychus* using the long bone scaling equation of Campione and Evans [73] whereby logBM = 2.749*logC_H+F_− 1.104 (BM, body mass; H, humeral minimum midshaft circumference; F, femoral minimum midshaft circumference; whereby C_H+F_, sum of humeral and femoral minimum midshaft circumference). Long bone circumference measurements were taken with a vinyl tape measure, which was marked up and then measured using digital callipers to two decimal places.

## Systematic Paleontology

MAMMALIA Linnaeus 1758 [74]

EUTHERIA Gill 1872 [75]

‘CONDYLARTHRA’ Cope 1881 [76]

PERIPTYCHIDAE Cope 1882 [77]

PERIPTYCHINAE Osborn & Earle 1895 [26]

*Periptychus* Cope 1881 [11]

*Catathlaeus* Cope 1881 [12]

Type species and only known species: *Periptychus carinidens* Cope, 1881 [11]

### Age and locality

Torrejonian to Tiffanian (~63.3- ~61.7Ma BP), Early Paleogene. Best known from the Nacimiento Formation, San Juan Basin, New Mexico, USA. Also known from North Horn Formation, Emery County, Utah, USA; Fort Union Hanna formations, Carbon County, Wyoming, USA; Black Peaks Formation, Big Bend National Park, Texas, USA; Fort Union Formation, Makoshika State Park, Montanta, USA; Animas Formation, San Juan Basin, Colorado, USA.

### Etymology

Cope did not explain the etymology of *Periptychus carinidens*. The generic name *Periptychus* derives from Ancient Greek, *peri* (around/near/surrounding) and the noun *ptych*, (fold/layer), yielding the word 'periptych' which is Latinised with the suffix -us. Thus, *Periptychus* translates as ‘folds around’ or ‘folds surrounding’. The species name *carinidens* derives from the Latin, (keel/prow) and *dens*, (tooth); with *carinidens* translating as ‘keeled-tooth’.

### Emended diagnosis

Upper premolars ovoid in occlusal view and enlarged relative to molars. Premolar paracone forms tall, erect, bulbous centralized cusp, cristae weak, flanked with crescentic lingual shoulder on P1-P5. Crescentic lingual shoulder formed by protocone and cuspules, which develop posteriorly along tooth row. P5 postcingulum is positioned level with protocone. Upper premolar parastyle forms a small but distinct mesially directed lobe. Lower premolars enlarged relative to molars. Premolar protoconid forms tall, erect, bulbous centralized cusp; flanked by paraconid mesiolingually and metaconid lingually on p2-p5. Talonid present on p1-p5, positioned lingually relative to protoconid and increasing in development posteriorly along tooth row. Upper molars near quadrate in occlusal view. Molar protocone flanked by subequally developed hypocone and protostyle. Hypocone and protostyle exhibit tendency to polybuny. Ectocingulum present but reduced. Paraconule and metaconule present and separate, with distinctive wing-like cristae. Lower molar cusps are well-separated. Paraconid distinct and separate, and only slightly smaller than the metaconid. The cristid obliqua is strong and where it intercepts the protocristid notch may be variably marked by an obliconid. The hypoconid, hypoconulid and entoconid form discrete, well separated cusps, and approach the trigonid cusps in height. The hypoconid and entoconid are subequal in size, the hypoconulid is slightly smaller.

### Differential diagnosis

The dentition of *Periptychus* differs from *Carsioptychus* in that the enamel is more strongly crenulated with distinct apicobasally aligned ridges. The upper postcanine dentition of *Periptychus* is not as transversely expanded as *Carsioptychus*, with the latter possessing a shallower lingual slope on all postcanine teeth. In *Periptychus*, the premolar paracone/protocone forms an erect cusp whereas in *Carsioptychus* the premolar paracone/protocone is distinctly posteriorly pitched. The premolar protocone of *Periptychus* forms a crescentic shoulder on all upper premolars, whereas in *Carsioptychus* the lingual shoulder is present on only P4 and P5 and more transversely expanded, with strong anteroposterior constriction and weak to indistinct cuspules. The lower premolar paraconid of *Periptychus* is more developed on p2-5, whereas the paraconid is only weakly developed on p4-5 and absent on p1-2 in *Carsioptychus*. The premolar metaconid is absent in *Carsioptychus*. The premolar trigonid is relatively more developed in *Periptychus* and positioned on the lingual side of the protoconid. Upper molars of *Periptychus* are near quadrate in occlusal view whereas they are more rectangular in *Carsioptychus* due to a longer lingual slope. The protostyle and hypocone of *Periptychus* are both well developed, subequal in size with a tendency to polybuny; in *Carsioptychus*, the hypocone is proportionally larger than the protostyle. A molar ectocingulum is present but reduced in *Periptychus*, and continuous and more prominent in *Carsioptychus*. The lower molar cusps of *Periptychus* are more widely separated and more subequal in size. The talonid of *Carsioptychus* is proportionally shorter and not as tall as that in *Periptychus* and lacks a strong cristid obliqua and obliconid. The ectocingulid is stronger in *Carsioptychus*.

### Species

*Periptychus carinidens* Cope 1881 p.337 [11]

*Catathlaeus rhabdodon* Cope 1881 p.487 [12]

*Periptychus rhabdodon* Cope 1882 p.465 [13]

*Periptychus brabensis* Osborn & Earle 1895 p.55 [26]

*Periptychus superstes* Matthew (in Simpson) 1935 p.25 [14]

*Periptychus rhabdodon superstes* Matthew 1937 p.121 [5]

*Periptychus gilmorei* Gazin 1938 p.275 [18]

#### Lectotype

**Fig 1:**
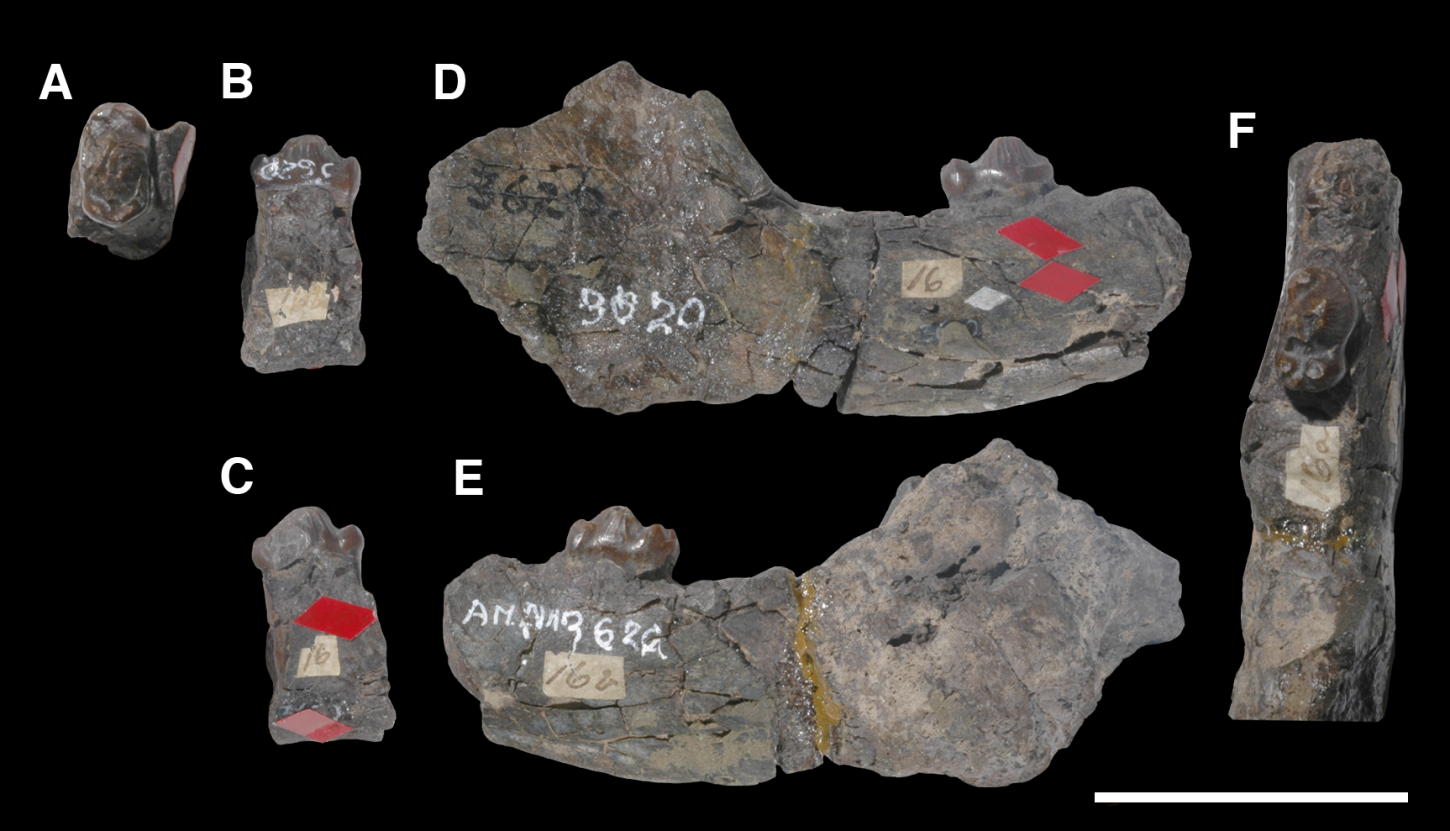
Lectotype of *Periptychus carinidens* (AMNH 3620). Left partial dentary with dp4 in (A) occlusal view; (B) buccal view; (C) lingual view. Right partial dentary with dp5 in (D) buccal view; (E) lingual view; (F) occlusal view. Scale bar: 30mm.

AMNH 3620, left partial dentary with dp3; right partial dentary with dp4 (Fig 1).

#### Lectotype locality

Nacimiento Formation, San Juan Basin, NM, USA.

#### Designation of lectotype

Herein we formally designate AMNH 3620 as the lectotype of *Periptychus carinidens* in accordance of Article 74 of the International Code on Zoological Nomenclature Code, specifically Article 74.6. Cope (1881) did not designate a holotype specimen in his original description of *Periptychus carinidens*. A left partial dentary with dp3 and right partial dentary with dp4, identified as *Periptychus carinidens* and described by Cope (1881), was first formally identified by a specimen number (AMNH 3620) in Matthew [5]. Matthew’s [5] inference that this specimen represents the type specimen is deemed to constitute lectotype fixation (International Commission on Zoological Nomenclature Article 74.6.1).

#### Hypodigm

**Fig 2:**
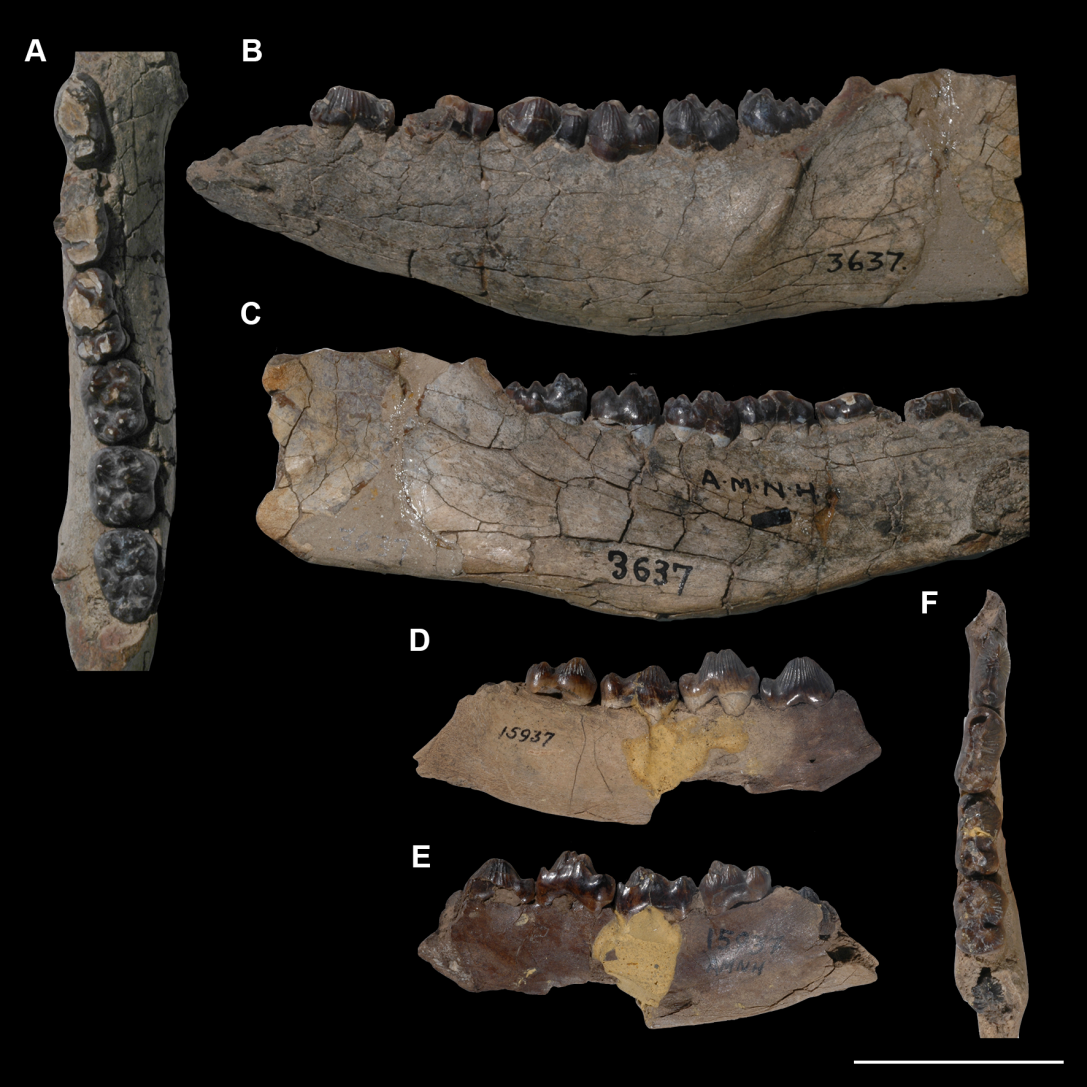
Hypodigm specimens of *Periptychus carinidens*. Left dentary with dp2-5, m1-3 (AMNH 3637) in (A) occlusal view; (B) buccal view; (C) lingual view. Right dentary with dp2-5, m1 and a partially erupted m2 (AMNH 15937) in (D) buccal view; (E) lingual view; (F) occlusal view. Scale bar: 30mm.

AMNH 3637, right mandibular fragment with dp2-5, m1-3; AMNH 15937, right dentary with dp2-5, m1 and a partially erupted m2 (Fig 2).

#### Diagnosis

Same as for genus.

#### Comments

We treat ‘*Periptychus gilmorei’* and ‘*Periptychus superstes’* as junior synonyms of *Periptychus carinidens* following Williamson [10], contra Archibald [2]. The morphological features which have been previously been stated as diagnostic of ‘*P*. *gilmorei’* [18,28] and ‘*P*. *superstes’* [5,14] are widely observed in specimens assigned to *P*. *carinidens*. A more convincing rationale for distinguishing between the purported species is the proportional sizes differences between the premolar and molar teeth. However, the size difference between Puercan ‘*P*. *gilmorei’* and older Torrejonian (To1) *P*. *carinidens*, and ‘*P*. *superstes’* and younger Torrejonian (To3) *P*. *carinidens* is less than the difference between older Torrejonian (To1) and younger Torrejonian (To3) *P*. *carinidens*, and therefore is not robust enough to warrant specific recognition.

## Comparative description

### Skull

The skull of *Periptychus* is reasonably well known from numerous specimens preserving various features of the cranial anatomy; however, a single complete skull remains unknown. The following observations are based on a selection of *Periptychus* specimens covering most of the cranial anatomy, including the auditory region. The alisphenoid and infraorbital regions of *Periptychus* remain poorly known at present.

**Fig 3:**
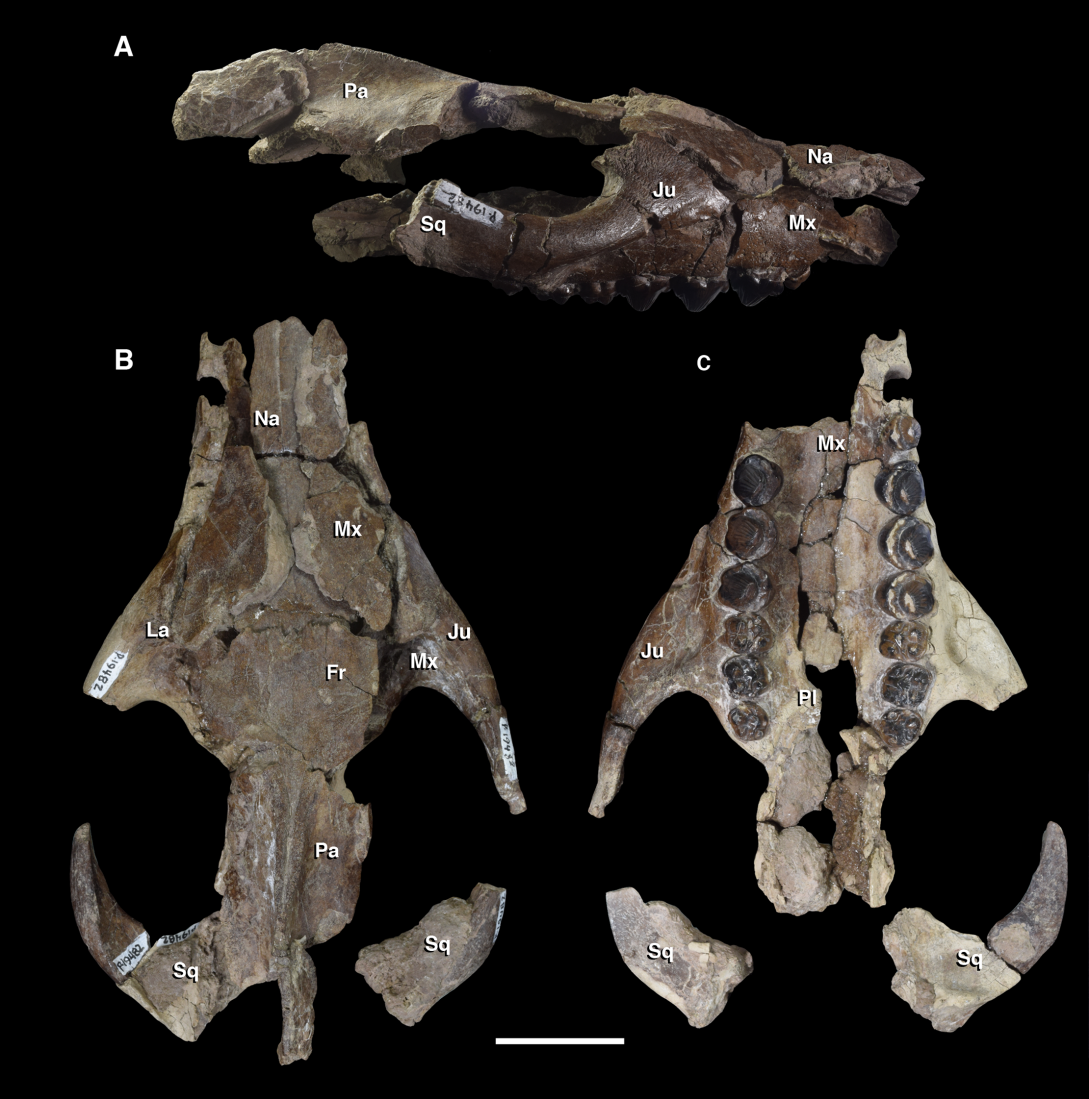
Skull of *Periptychus carinidens* (NMMNH P-19482). (A) lateral view; (B) dorsal view, (C) ventral view. Abbreviations:; Fr, frontal; Ju, jugal; La, lacrimal; Mx, maxilla; Na, nasal; Pa, parietal; Pl, palatine; Sq, squamosal. Scale bar: 30mm.

**Fig 4:**
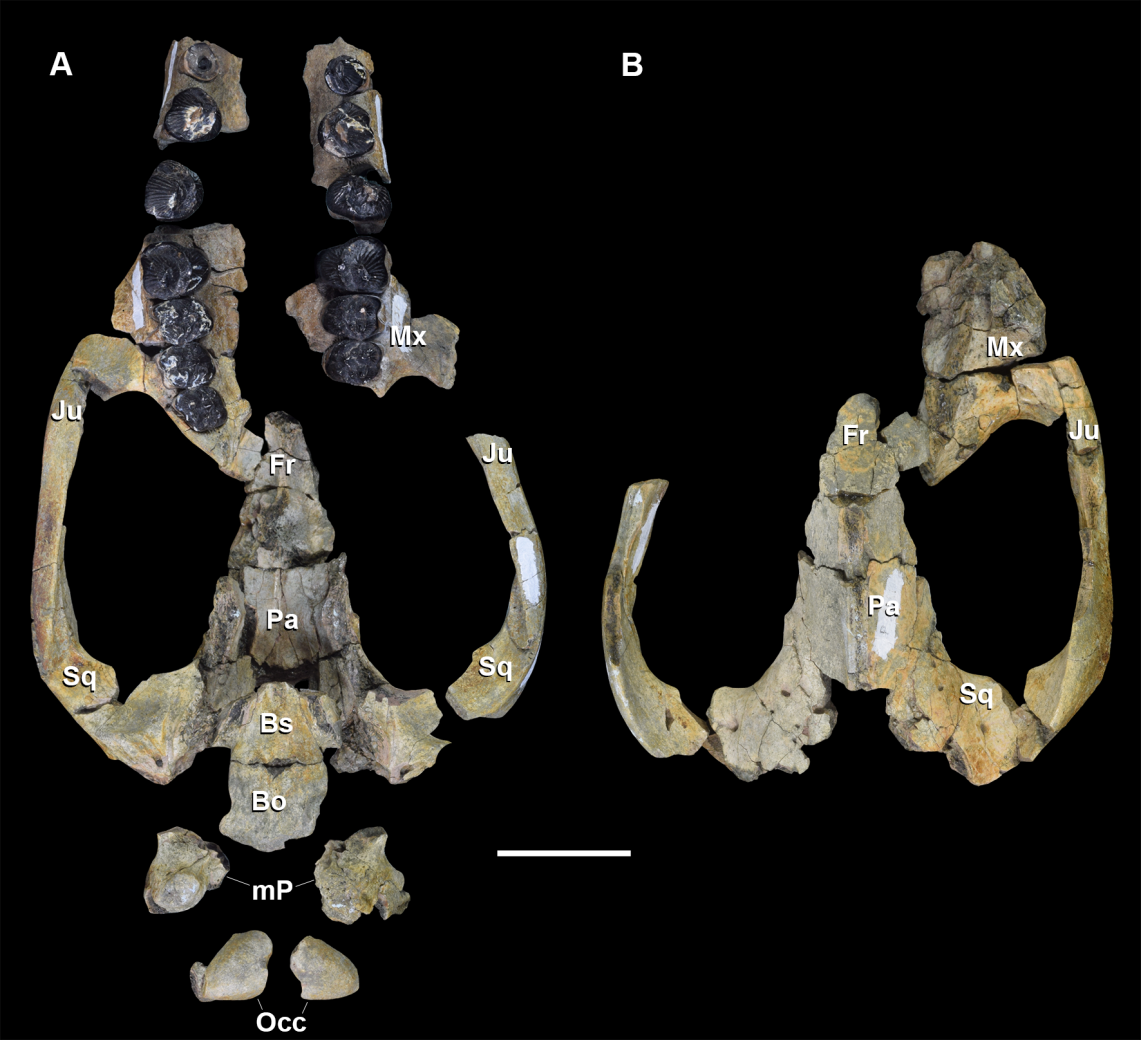
Skull of *Periptychus carinidens* (NMMNH P-36631). (A) ventral view, (B) dorsal view. Abbreviations: Bo, basioccipital; Bs, basisphenoid; Fr, frontal; Ju, jugal; mp, mastoid protuberance; Mx, maxilla; Pa, parietal; Occ, occipital; Sq, squamosal. Scale bar: 30mm.

Comparative taxa used in the following description of the skull bones include: *Carsioptychus coarctatus* (AMNH 27601, figured in [5]), *Ectoconus ditrigonus* (AMNH 16500, figured in [5]), *Arctocyon primaevus* (MNHN.F.CR700 figured in [52]), *Claenodon ferox* (NMMNH P-8627 figured in [78]) and *Pantolambda bathmodon* (AMNH 16663, figured in [5]). Detailed comparisons of the auditory region are made to *Protungulatum* (AMNH 118359, figured in [79]), *Arctocyon primaevus* (MNHN BR. L9) and *Pantolambda bathmodon* (AMNH 16663, figured in [69]). Comparisons of the dentition are primarily made to *Carsioptychus coarctatus*, *Ectoconus ditrigonus*, and other periptychid taxa where necessary. The overall construction and morphology of the skull of *Periptychus* is typical of a medium sized ‘condylarth’, albeit somewhat stouter in form (Figs 3 and 4). The rostrum of *Periptychus* is moderately elongate, dorsoventrally deep and tapers anteriorly with no rostral constriction. The rostral morphology of *Periptychus* is broadly similar to that in *Carsioptychus* and *Ectoconus*, all of which possess a dorsoventrally deep and anteriorly tapering snout. The rostrum of *Periptychus* is not as elongate as that of *Arctocyon*, but is longer than the comparatively short rostrum of *Pantolambda*. Further to this, both *Arctocyon* and *Pantolambda* exhibit a degree of rostral constriction between the upper canine and ultimate upper premolar.

In lateral view, the dorsal surface of skull of *Periptychus* is relatively flat in comparison to the more domed morphology exhibited by *Pantolambda*. The anterior portion of the skull of *Periptychus* (rostrum and frontal region) gently slopes anteroventrally and is mediolaterally broad across the facial region. The posterior portion (braincase) is somewhat taller than the anterior skull in lateral aspect, due to the moderate expansion of the sagittal crest rather than an expanded braincase.

The zygomatic arches of *Periptychus* are broad and laterally spreading. In dorsal view, the zygomatic arches exhibit a sub-rectilinear profile. The anterior portion of the arch rapidly spreads away from the facial region before extending posteriorly along a parasagittal plane. Posteriorly, the arch forms a rounded angle, anterior of the glenoid fossa. The dorsal profile of the zygomatic arches of *Periptychus* is more rectilinear than that observed in *Carsioptychus* and *Ectoconus*, where the arches are more convex in profile. The arch morphology in *Periptychus* is not as angled as that in *Arctocyon* and *Pantolambda*. In *Arctocyon* and *Pantolambda*, the posterior angle of the zygomatic arch is positioned anterior of the glenoid fossa as in *Periptychus*, but is considerably sharper, forming a near right angle.

**Fig 5:**
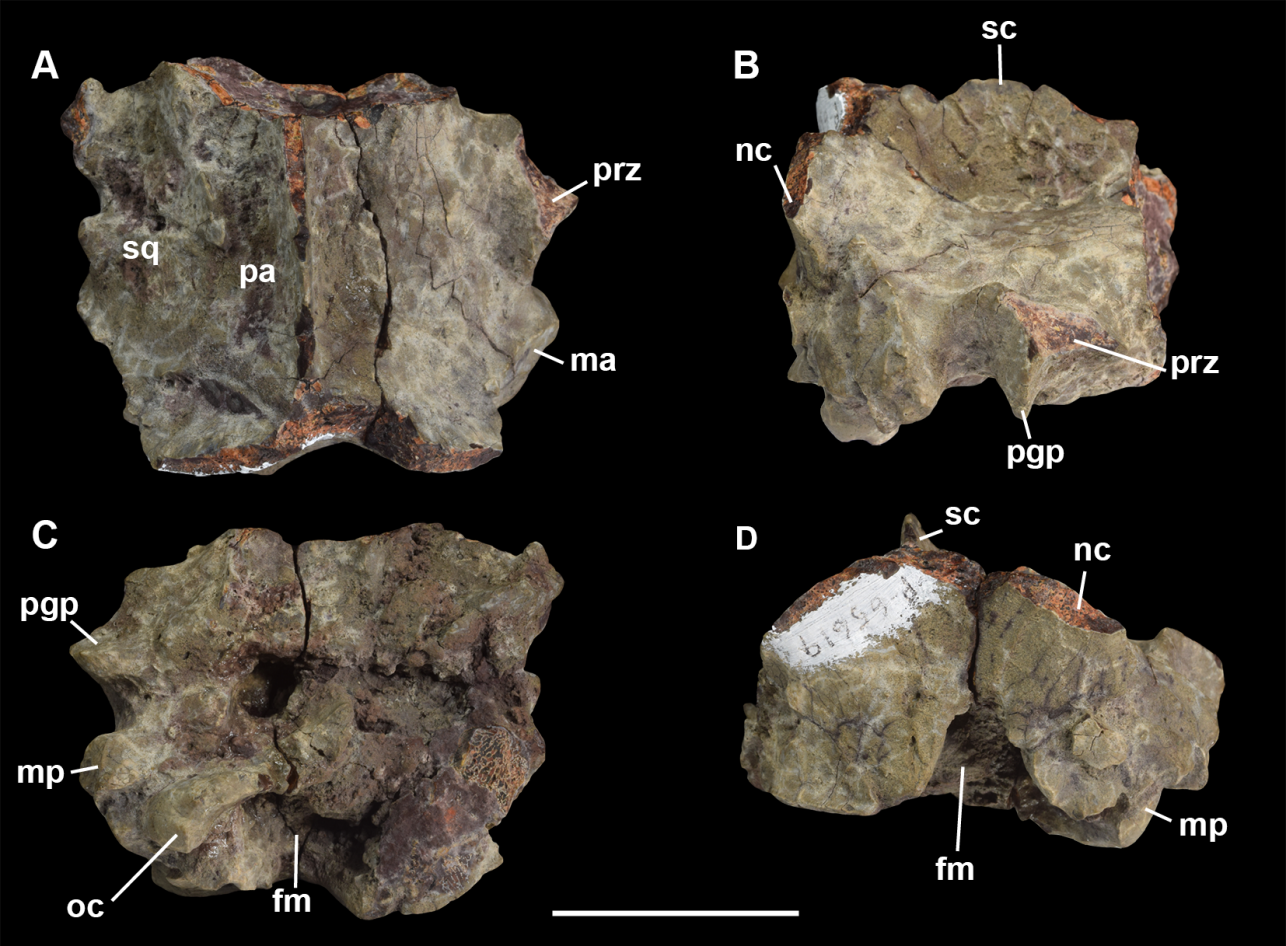
Braincase of *Periptychus carinidens* (NMMNH P-65619). (A) dorsal view; (B) lateral (right) view; (C) ventral view; (D) posterior view. Abbreviations: fm, foramen magnum; pgp, glenoid process; ma, mastoid; mp, mastoid protuberance; nc, nuchal crest; oc, occipital condyle; pa, parietal; prz, posterior root of the zygomatic arch; sc, sagittal crest; sq, squamosal. Scale bar: 30mm.

**Fig 6:**
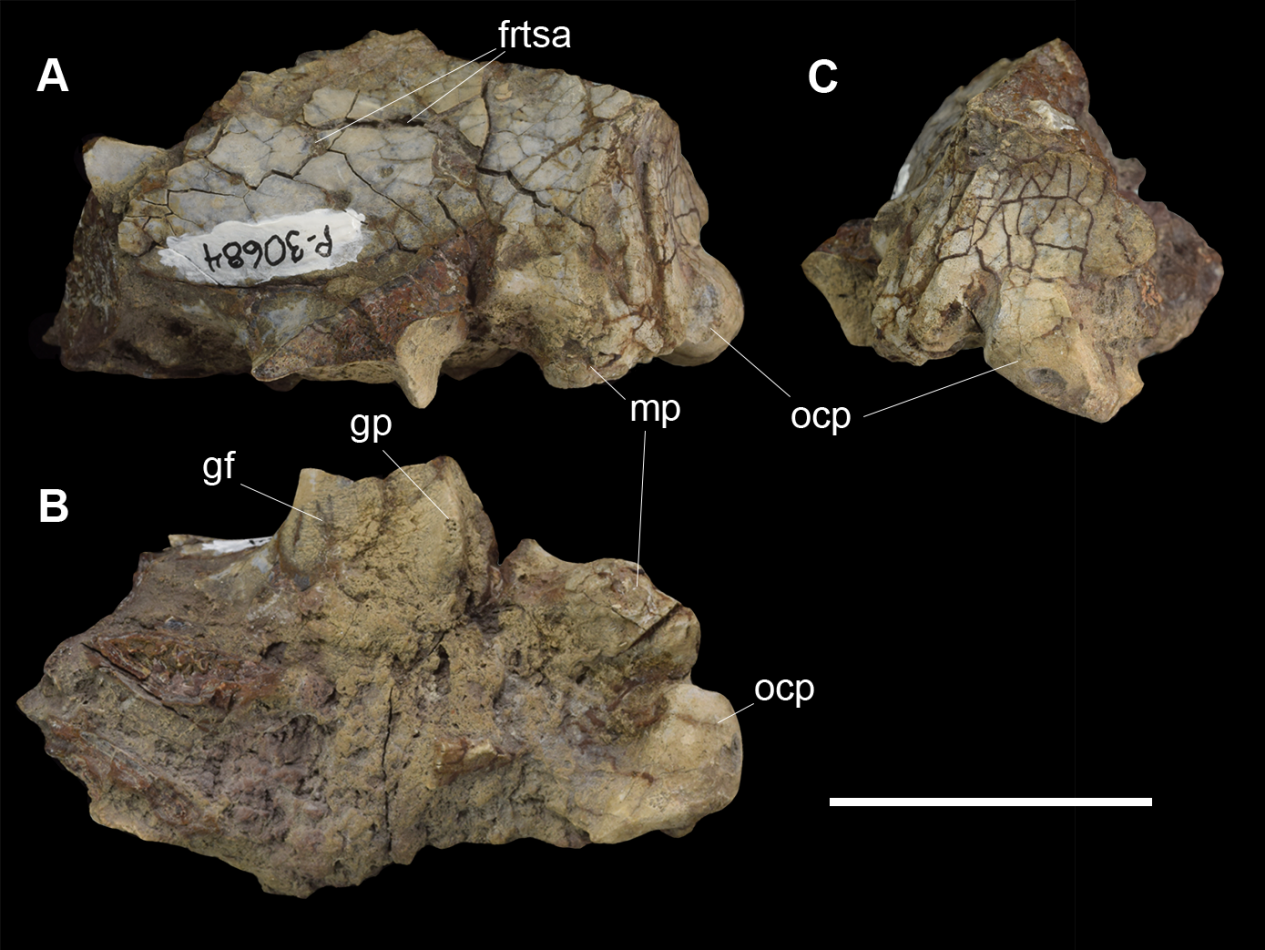
Partial left braincase of *Periptychus carinidens* (NMMNH P-30684). (A) lateral view; (B) ventral view; (C) posterior view. Abbreviations: frtsa, foramen for the rami temporalis of the stapedial artery; gf, glenoid fossa; gp, glenoid process; mp, mastoid process; ocp, occipital condyle. Scale bar: 30mm.

The braincase of *Periptychus* is small and low, with well-developed sagittal and nuchal crests (Figs 5 and 6). In dorsal view, the braincase is mediolaterally broadest at the level of the temporomandibular joint. Anteriorly from this point, the braincase tapers and is strongly constricted where it contacts the frontal region. Posteriorly, the braincase tapers slightly but remains relatively broad due, in part, to the relatively large exposure of the mastoid on the lateroventral surface of the braincase. The braincase of *Periptychus* is proportionally mediolaterally broader but not as anteroposteriorly elongate as that in *Arctocyon*. The braincase morphology is notably different to that observed in *Pantolambda* where the braincase continues to increase in width posteriorly beyond the temporomandibular joint.

### Nasal

The delicate paired nasal bones of *Periptychus* are not well known. The following description is based on NMMNH P-19482, a portion of the skull roof including the nasal bones. In this specimen, the nasals are incomplete, the lateral edges are highly damaged and the anterior nasal aperture is absent. Comparisons will be made to *Arctocyon primaevus* (MNHN.F.CR700, figured in [52] and MNHN BR L9), *Claenodon ferox* (NMMNH P-8627 figured in [78]), and *Pantolambda bathmodon* (AMNH 16663, figured in [5]).

The paired nasal bones of *Periptychus* are anteroposteriorly long and together form a broad, flat roof over the rostrum. Based on NMMNH P-19482 (Fig 3), the anteroposterior length of the nasal is equal to approximately 50% of the anteroposterior length of the skull (note that on this specimen the anterior tip of the nasal is missing and the posterior end of the sagittal crest is damaged). The nasals of *Periptychus* are proportionally slender and elongate, forming a flat roof over the rostrum rather than a transversely arched roof as seen in *Pantolambda*, *Arctocyon* and *Claenodon*. The anterior-most portion of the nasals is unknown for *Periptychus*, and it is not possible to determine the nature of the contact with the premaxilla or the morphology of the nasal aperture from the specimens available.

Posteriorly, the nasals extend past the anterior edge of the anterior root of the zygomatic arch to terminate at a point well within the transverse level of the orbit and approximately level with M3. Based on the shape of the bone present on NMMNH P-19482, the nasals are highly expanded posteriorly and appear to form a broad contact with the paired frontals. The frontals of NMMNH P-19482 overlie the nasals and are broken along a transverse line that could be interpreted as the nasofrontal suture. However, the ventral surface of the specimen shows the nasal bones tapering to point posteriorly along the sagittal axis of the skull that extends well under the frontals.

As such, there are several plausible interpretations for the nasofrontal suture in *Periptychus*. It is possible that the nasals projected posteriorly into the frontal region on the dorsal surface of the skull, as observed in *Arctocyon*, so that in NMMNH P-19482 the frontal has been displaced over the nasal and the maxillary processes of the frontals are missing. Alternatively, the nasals may have formed a mediolaterally broad nasofrontal contact on the dorsal surface of the skull with a posterior projection of the nasals on the ventral surface of the frontals. It is also plausible that the condition of *Periptychus* is an intermediary between the two aforementioned states, in which the nasals form a small dorsal projection into the frontal with a larger ventral extension underlying the frontal.

Comparisons with *Ectoconus* help elucidate the nasal morphology in *Periptychus*. In *Ectoconus*, the dorsal surface of the nasal shows a small, mediolaterally narrow posterior projection into the frontal region, which tapers to a posterior point which approximates the transverse plane of the mesial surface of the ultimate upper molar. Given the similarity of preserved portions of the nasal, *Periptychus* likely possessed a similar morphology to that observed in *Ectoconus*. In *Claenodon* and *Arctocyon*, the nasals form a more distinct posterior projection into the frontal region on the dorsal surface of the skull than that observed in *Ectoconus*. The condition in *Pantolambda* is harder to describe as the nasals have been displaced over the frontals, which artificially shortens the anteroposterior length of the frontal region. Regardless, the nasals are anteroposteriorly short, barely extending past the plane of the anterior root of the zygomatic arch, with a broadly rounded posterior border.

In dorsal aspect, the midsection of the nasals of *Periptychus* is mediolaterally constricted and forms a concave suture with the maxillae. The anterior portion of the nasals are not as broad as the posterior region, with the posterior region expanding abruptly from above P2 to the frontal region. The nasal foramina are not observable along the nasofrontal suture on NMMNH P-19482. The constriction of the nasals in *Periptychus* is not as extreme as in *Arctocyon*, but is more marked than the very slight constriction seen in *Claenodon* and *Pantolambda*. Posteriorly, the nasal is excluded from contacting the lacrimal by the maxilla and frontals.

In *Periptychus*, the ventral surface of the nasal exhibits a dorsal nasal meatus. Two grooved nasal fossae are separated by a distinct nasal septum along their entire length, and laterally delimited by a curved ridge for the attachment of the nasoturbinals. The nasal fossae are mediolaterally broad anteriorly and taper to a posterior point which underlies the frontals. It is not clear if the lateral surfaces of the nasals are damaged and extended to the same posterior point as the dorsal nasal meatus or diverged laterally prior to the posterior projection of the meatus. On each side, posterolateral to the dorsal nasal meatus, there are two open ended-ovoid fossae. The nasals appear to contribute to the anterior border of the fossae, with the frontal contributing to the posterior border. Based on their posterodorsal position, we tentatively infer these depressions to be a pair of fossae for the frontotubinals.

#### Premaxilla

The premaxilla of *Periptychus* is very poorly known. Of the specimens observed, only three preserve portions of the premaxilla: NMMNH P-19482, a subcomplete skull, (Fig 3); NMMNH P-35194, a highly concreted specimen preserving a small portion of the right premaxilla above the third incisor; and AMNH 3665, a subcomplete skull which preserves a similar portion of the premaxilla as NMMNH P-35194, but lacks an associated incisor. Comparisons are with *Claenodon ferox* (NMMNH P-8627 figured in [78]); *Arctocyon primaevus* (MNHN.F.CR700, figured in [52] and MNHN BR L9) and *Pantolambda bathmodon* (AMNH 16663, figured in [5]).

The upper canine and upper third incisor of NMMNH P-35194 are subequal in size and separated by a narrow diastema. This condition to appears to be unique to *Periptychus* compared to *Ectoconus*, *Arctocyon*, *Claenodon* and *Pantolambda*, which exhibit a much greater size disparity between the upper canine and upper third incisor, with a much larger diastema between the teeth. The third incisor of *Periptychus* is situated within the premaxilla whilst the canine is situated within the maxilla and possesses a deep root (subequal in size to the crown). Given the proximity of the teeth, there is no room for the posterior migration of the premaxilla, suggesting a deep, dorsoventrally aligned contact between the premaxilla and maxilla which restricted the posterodorsal process of the premaxilla to the dorsal-most edge of the rostrum.

#### Maxilla

The following maxilla description is based on: NMMNH P-19482, a subcomplete skull preserving the lateral and palatal components of the left and right maxillae separately (Fig 3); NMMNH P-36631, a fragmentary skull preserving parts of the left and right maxilla above the dentition (Fig 4); and NMMNH P-35194, a concreted specimen preserving the upper dentition in the maxilla. Comparisons will be made to *Carsioptychus coarctatus* (AMNH 27601 figured in [5]); *Claenodon ferox* (NMMNH P-8627 figured in [78]); *Arctocyon primaevus* (MNHN.F.CR700, figured in [52] and MNHN BR L9) and *Pantolambda bathmodon* (AMNH 16663, figured in [5]).

The surface texture of the maxilla of *Periptychus* is unusual and features a distinct network of fine pits just above the tooth row; this texturing is particularly concentrated above the premolars and around the infraorbital foramen. The pits are numerous and variable in size and shape: some are circular while others are more ovoid, but not exceeding 0.7 mm in diameter. In life, they most likely housed a network of capillaries supplying blood to the external maxilla and possibly innervated the vibrissae.

The paired maxillae form the lateral walls of the rostrum. They extend posteriorly and contribute to the anterior root of the zygomatic arches. Ventrally they form the lateral and anterior components of the hard palate and house the upper canine-postcanine dentition. Anteriorly, the maxilla contacts the premaxilla. Given that the anterior-most maxilla and premaxilla of *Periptychus* are poorly known, very little can be deduced about the anterior rostrum and nasal aperture. Based on the size and depth of the known maxillae and the position of the anterior dentition, the maxilla-premaxilla contact must have been deep and dorsoventrally orientated.

Dorsally the lateral walls of the maxillae overlay the nasals. The external suture between the maxilla and nasal extends anteroposteriorly to form a concave arc, with no distinct projections. The vertical walls of the maxillae are deep and form the lateral walls of the rostrum. Dorsoventrally, the maxillae walls are slightly convex so that with the nasals they form a rounded rostrum in anterior aspect. Anteroposteriorly, the maxillae are long and positioned subparallel to one another; there is no constriction along the rostrum. The lack of rostral constriction in *Periptychus* is evident in all specimens and is notably different from the conditions in *Ectoconus*, *Claenodon*, *Arctocyon* and *Pantolambda* in which the maxillae constrict above the second upper premolar. This condition is most exaggerated in *Claenodon* and *Arctocyon*, but still evident in *Ectoconus* and is more subdued in *Pantolambda*. Curiously, the maxillae of *Carsioptychus* exhibit a lateral expansion above the ultimate and penultimate upper premolars that is echoed in the arc of the postcanine dentition. This lateral expansion is somewhat apparent in *Periptychus* but is reduced to a very subtle expansion.

The anterior opening of the infraorbital canal is demarcated by a single infraorbital foramen. The foramen is positioned on the lateral wall of the maxilla directly above the midpoint of the penultimate premolar at the same level as (but anterior to) the ventral edge of the anterior root of the zygomatic arch. It forms an anteriorly directed opening for the infraorbital canal, which transmitted the infraorbital artery and infraorbital nerve (CN V_2_). The position of the infraorbital foramen in *Periptychus* is comparable to *Arctocyon* and *Claenodon* but differs from *Ectoconus* and *Pantolambda*, both of which exhibit a more anteriorly placed opening above the second upper premolar, but which is positioned closer to the anterior root of the zygomatic arch. In *Periptychus*, the anterior and posterior infraorbital foramina are smaller than the lacrimal foramen and proportionally much smaller than the infraorbital foramina of any of the comparison taxa. Furthermore, in *Ectoconus*, *Arctocyon*, and *Pantolambda* the anterior infraorbital foramen opens into a shallow, anteriorly directed ovoid groove, which is not observable on *Periptychus* (it is also indistinct on *Claenodon*, but this may be due to damage). In NMMNH P-36631 the infraorbital canal is exposed and it almost twice the width as that of NMMNH P-19482. The length of the infraorbital canal in all observed specimens is far greater than one upper molar length; in NMMNH P-19482 it is approximately 2.5 times the length of M1 (which is equivalent to the length of the ultimate and penultimate premolars.

The posterodorsal region of the maxilla is preserved in NMMNH P-19482 and AMNH 3665, but the inferred life position of the maxilla relative to other bones in this vicinity is somewhat tentative. In NMMNH P-19482 the maxillae are displaced ventrally over the nasals, both lacrimals are incomplete and the nature of the contact between frontals and nasals is somewhat ambiguous; in AMNH 3665 the rostrum has been heavily reconstructed. Based on the size and configuration of the known bones from both specimens, we tentatively infer that posterodorsally the maxilla contacts the frontal at the base of the rostrum and in doing so excludes the nasals from contacting the lacrimal on the dorsal skull surface. The maxilla contacts the lacrimal on the edge of the orbit, with the suture between the maxilla and lacrimal following the contour of the orbital rim and inhibiting the facial process of the lacrimal from making any contribution to the rostral region of the skull.

The maxillae contribute a large portion of bone to the anterior root of the zygomatic arches. In lateral aspect, the maxilla does not extend far onto the zygomatic arch; it contacts the jugal at a point just dorsal of the lacrimal foramen, forming a concave contact. The maxilla and jugal do not interdigitate on the lateral surface of the zygomatic arch, unlike in *Arctocyon* where the maxilla bifurcates into a short process on the ventral edge of the zygomatic and a long process in between the short and long processes of the jugal. In *Periptychus* the long process of the jugal occupies the entire lateral surface of the zygomatic arch, displacing the reduced long and short processes of the maxilla to the ventral surface of the anterior root of the zygomatic arch.

The maxilla extends underneath the jugal to form an anteroposteriorly deep orbital floor. The orbital floor of *Periptychus* forms a large, horizontal shelf in the anterior corner of the orbit and is completely composed of maxillary bone. On the ventral surface, the orbital floor houses the entire molar row. In dorsal aspect, the maxillary bone of the orbital floor forms a sweeping convex suture which abuts the jugal laterally on the medial wall of the zygomatic arch; it extends into the anterior corner of the orbit and onto the anterior wall of the orbit, where it contacts the transversely concave ventral border of the lacrimal above the posterior infraorbital foramen. The posterior infraorbital foramen marks the orbital opening of the infraorbital canal; it forms a small posteriorly directed opening within the maxilla at the anterior corner of the orbit. The medial contacts of the maxilla are more ambiguous. A sharp groove is positioned near the medial limit of the maxillary component of the orbital floor, but the medial wall of the orbit is poorly preserved, making observations on the contact between the maxilla, lacrimal, palatine and frontal difficult. The labial roots of the ultimate upper molar roots are just visible on the dorsal surface of the orbital floor and provide some contour to the surface of the bone. The bone texture on the dorsal surface of the orbital floor displays a distinct pitting texture where the inferior oblique muscle attached. The pitting is evident in *Carsioptychus* and *Ectoconus*, but to a lesser extent than in *Periptychus*.

The condition in *Periptychus* is most like that in *Ectoconus*, both of which possess a larger orbital floor than *Carsioptychus*. The orbital floor of *Arctocyon* is not as large as *Periptychus*, and in *Claenodon* it is highly reduced. *Pantolambda* possesses a highly expanded orbital floor which occupies almost 50% of the orbital space in ventral view, with the single lingual root of the ultimate upper molar (M3) visible on the dorsal surface of the orbital floor.

On the ventral surface of the zygomatic arch of *Periptychus*, the contact between the maxilla and jugal shallowly interdigitates. The maxilla on the ventral surface of the anterior root of the zygomatic arch is expansive, with the jugal restricted to forming a small short process which projects slightly into the maxillary region. On the medial surface of the zygomatic arch the maxilla contribution to the orbital floor closely abuts the anterior jugal; it extends from the orbital floor to the dorsal rim of the zygomatic arch. Posteriorly, the zygomatic process of the maxilla extends along the medial surface of the jugal. Based on NMMNH P-36631 it appears that the zygomatic process of the maxilla extends posteriorly along the medial wall of the zygomatic arch to a point just posterior to where the squamous process of the zygomatic terminates dorsally over the jugal.

The ventral surface of the anterior root of the zygomatic arch forms an anteroposteriorly broad shelf which extends as far as the mesial border of the ultimate upper premolar (P5). The ventral surface exhibits two well-defined ovoid fossae, which we interpret as particularly well-developed attachment sites for the zygomaticus major and minor muscles. The posterior border of the ventral surface of the anterior root of the zygomatic arch is demarcated by a thick ridge which is continuous with the ventral edge of the zygomatic arch. A small tubercle is positioned on the ridge level with the second upper molar (M2); this is part of the attachment area of the masseter. The ventral surface of the maxilla on the anterior root of the zygomatic arch is very well developed in *Periptychus*. None of the comparison taxa exhibit such a well-defined shelf, although the zygomatic arches of *Pantolambda* exhibit a similar level of mediolateral expansion (but the ventral surface is predominantly occupied by the dentition).

On the ventral surface of the skull the maxillae contribute to the hard palate, forming the anterior and lateral components of the palate in conjunction with the premaxilla anteriorly and the palatines posteromedially. The maxilla contribution surrounds the lateral edges of the palatines and supports the canine and post-canine dentition.

The alveolar processes of the maxillae house the dentition and are dorsoventrally deep, giving the palate a highly arched profile in anterior view. Posteriorly the alveolar processes project past the posterior edge of the palatines to form a pronounced maxillary tuberosity above the ultimate upper molar. A maxillary tuberosity housing the ultimate upper molar is present in all comparison taxa. In *Periptychus* the dorsal surface of the tuberosity contributes to the medial half of the orbital floor with the portion of the orbital floor lateral to the tuberosity forming a notch, the apex of which is level with the distal edge of the penultimate upper molar. In *Carsioptychus* the lateral notch is even deeper, extending to the level of the ectoflexus of the penultimate upper molar; in *Ectoconus*, *Arctocyon* and *Claenodon* the morphology of the notch is comparable to *Periptychus*. *Pantolambda* exhibits an unusual condition in this respect: the maxillary tuberosity is very well developed and the lateral notch is distinct, but the tuberosity does not extend past the mesial border of the ultimate molar.

In *Periptychus* the maxillary tuberosity is medially defined by the minor palatine notch. The notch is variably developed in different specimens. In NMMNH P-19482 the notch is present and reasonably distinct, so it forms a rounded apex that is directed anteromedially. In NMMNH P-36631, however, the medial notch is indistinct. In *Carsioptychus*, *Ectoconus*, *Arctocyon* and *Claenodon* the medial notch is highly reduced so that the maxillary tuberosity is not well defined from the palatine; in *Pantolambda* the notch is relatively well developed so that the notch is transversely aligned and its apex is directed anteriorly. In *Periptychus* and all the comparison taxa except *Pantolambda*, the maxillary tuberosity surrounds the ultimate upper molar, forming a rim around its distal edge. In *Pantolambda* the ultimate upper molar is positioned on the very edge of the tuberosity. The development of the maxillary tuberosity and the associated lateral and medial notches of *Periptychus* are likely related to the mesial migration of the molars during ontogeny [69]. A similar morphology is observed in the South American pantodont, *Alcidedorbignya inopinata* [69], and as such, comparisons of the development of the maxillary tuberosity between taxa are here made with caution.

The palatine process of the maxilla surrounds the horizontal processes of the paired palatine bones. The hard palate of *Periptychus* is best observed on NMMNH P-19482. Here, the palatines overlay the maxillae but the maxilla-palatine suture is not well defined. The maxillary contribution to the posterior hard palate is restricted and provides more dorsoventral height to the palate than mediolateral width, as opposed to the condition in *Pantolambda* where the maxilla provides a mediolaterally broad shelf between the dentition and lateral edges of the horizontal process of the palatine. Posteriorly the maxilla-palatine suture in *Periptychus* is marked by a short, deep sulcus on the palatine, which extends around the mesial and lingual border of the ultimate upper molar. On the left side of NMMNH P-19482 the maxilla-palatine suture is visible and extends dorsoventrally underneath the alveolar line of the molar row, and then at the level of the first upper molar protostyle (= pericone) the suture turns medially towards the inferred position of the major palatine foramen. On the same specimen, we infer a small broken notch on the left side of the palate at the level of the hypocone on the first upper molar as the greater palatine foramen. The maxilla-palatine suture is less well-defined on the left side but clearly shows an anterior projection of the palatine lateral to the major palatine foramen. A shallow sulcus extends anteriorly from the major palatine foramen to a point on the palate level with the medial border of the ultimate upper premolar. The incisive foramen is not preserved on any of the specimens observed and it is not possible to deduce the presence or absence of a maxillary fossa. On the ventral surface of the palate the nasal septum marks the midline suture between the paired maxillae and palatines. The mesial roots of the last three premolars form prominent bony protuberances along the lateral edge of the dorsal surface of the hard palate.

#### Palatine

The delicate paired palatine bones are not well known for *Periptychus*. The following description is based on NMMNH P-19482, which preserves fragments of the palatines within the hard palate (Fig 3); NMMNH P-36631, which preserves fragments of the posterior parts of the palatines (Fig 4); and AMNH 3669, which also preserves fragments of the posterior palatine.

The horizontal processes of the palatines form the posterior quarter of the hard palate. They form a mediolaterally broad plate of thin bone which overlays the maxillae. The maxilla-palatine suture extends along the palate adjacent to the alveolar line of the molars and turns medial at the point level with the protostyle on the first upper molar. Posteriorly, the contact between the maxilla and palatine is marked by the minor palatine canal which runs from the posterolateral edge of the hard palate along the lingual edge of the ultimate upper molar towards the posterior most accessory palatine foramen.

At least four pairs of foramina pierce the horizontal process of the palatine. The most anterior opening is likely the major palatine foramen, which forms a large anteriorly directed opening level with the hypocone of the first upper molar. In life, the major palatine foramen conveyed the major palatine nerve and vessels [80]. Three pairs of accessory foramina are preserved within the palatines on NMMNH P-19482. The first is positioned immediately posterolateral to the major palatine foramen and opens ventrally. Immediately adjacent to the first accessory foramen is a small depression which could be interpreted as a second accessory foramen. The third accessory foramen is positioned posteriorly and slightly medial to the second accessory palatine foramen and is level with the hypocone of the penultimate upper molar. The minor palatine foramen forms a shallow notch rather than a discrete foramen on the posterior border of the palatines, posteromedial to the hypocone of the ultimate molar and in life conveyed the minor palatine nerve and associated vessels [80].

The horizontal processes of the palatines in *Periptychus* are mediolaterally broader but anteroposteriorly shorter than those of *Pantolambda*. In *Pantolambda* the palatines form a narrower finger-like projection to the mesial edge of the penultimate molar and the mediolateral width is approximately subequal to the buccolingual width of the penultimate upper molar. *Ectoconus*, *Arctocyon* and *Pantolambda* all possesses a minor palatine notch rather than a discrete foramen. In *Pantolambda* and *Arctocyon* the notch is more developed than in *Periptychus* and *Ectoconus*; however, the associated minor palatine canal in *Pantolambda* and *Arctocyon* are not as well-defined and are composed of both the palatine and maxilla rather than just the palatine as in *Periptychus* and *Ectoconus*.

The posterior margin of the hard palate is delimited by the postpalatine torus, which form the ventral surface of the choanae. The choanae open at the level of the protocone of the ultimate upper molar. The postpalatine torus of *Periptychus* is known from NMMNH P-19482, which preserves the lateral portions of the torus and suggests a biconcave profile as seen in *Carsioptychus*, *Arctocyon* and *Pantolambda*. However, a transverse torus cannot be ruled out completely at this point. The edge of the torus forms a thick rim, with its anterior border edged by a shallow but defined sulcus for the attachment of the tensor veli palatine muscles laterally and the musculus uvalae medially. The nasal surface of the palatine is exposed on NMMNH P-19482 and displays a raised median palatine suture which would have articulated with the vomer.

The perpendicular processes of the palatines are not well-known for *Periptychus*. NMMNH P-19482 preserves a small fragment of the perpendicular processes on both the left and right sides. The palatines extend dorsoanteriorly towards the anterior orbit; the ventral suture of the palatine with the maxilla along the medial edge of the orbital floor is not evident. On the right side of NMMNH P-19482 we tentatively infer the presence of the sphenopalatine foramen preserved within the palatine. The foramen is positioned in a deep fossa at a point level with the paracone on the ultimate upper molar, and posteriorly it opens into a posteriorly shallowing sphenopalatine groove. The posterior contact between the perpendicular process of the palatine with the pterygoid is not defined.

#### Lacrimal

The lacrimal of *Periptychus* is incompletely known. The following description is based on NMMNH P-19482, which preserves parts of the lacrimal over the anterior root of the zygomatic arch on both the left and right sides (Fig 3); NMMNH P-36631, which preserves a small portion of the right lacrimal in the anterior-most corner of the orbit (Fig 4); and AMNH 3665, which preserves fragments of the anterior orbital region. The extent of the contribution made by the lacrimal to the anterior wall of the orbit is unknown. Comparisons are made to *Carsioptychus coarctatus* (AMNH 27601 figured in [5]); *Ectoconus ditrigonus* (AMNH 16500, figured in [5]); *Arctocyon primaevus* (MNHN.F.CR700, figured in [52] and MNHN BR L9) and *Pantolambda bathmodon* (AMNH 16663, figured in [5]).

The lacrimal of *Periptychus* forms a small plate situated in the anterior corner of the orbit. Anteriorly the lacrimal contacts the maxilla via a simple convex suture situated on the rim of the orbit. As such, the facial process of the lacrimal in *Periptychus* is effectively absent and the lacrimal is completely contained within the orbit. *Carsioptychus* and *Ectoconus* appear to display a similar condition to *Periptychus* in which the lacrimal is contained within the orbit. However, in both specimens the anterior orbit is poorly preserved. In *Arctocyon* the lacrimal forms a prominent triangular facial process which contacts the frontal dorsally, maxilla anteriorly (extends anterior relative to jugal) and the jugal ventrally. In *Pantolambda* a small but defined facial process of the lacrimal is evident as a narrow rim on the external surface of the anterior root of the zygomatic arch; it is more developed than in *Periptychus* but not as extensive as in *Arctocyon*. In both *Arctocyon* and *Pantolambda* the facial process of the lacrimal is positioned high on the rostrum, above the anterodorsal long process of the jugal.

In *Periptychus*, the lacrimal contacts the jugal along its ventral border on the dorsal surface of the anterior root of the zygomatic arch. The lacrimal exhibits a small contact with the jugal on its anterior border, which is continuous with the lacrimomaxillary suture anterodorsally. In posterior view, the orbital process of the lacrimal of *Periptychus* (or at least what is preserved of it) is roughly quadrilateral in shape and positioned in the anterior-most corner of the orbit. The medial portion of the lacrimal and associated sutures are poorly preserved; however, it appears that the orbital process of the lacrimal contacts the jugal ventrolaterally, the maxilla anterodorsally (on the orbital rim) and ventrally (along the orbital floor), and the frontal medially, with apparently little or no contact with the palatine. The enlarged orbital floor formed by the maxilla and the apparent expansion of the facial process of the frontal (based on AMNH 3665) appears to impede the palatine from forming a broad contact with the relatively reduced lacrimal. However, it should be recognised that this region of the skull is very poorly known for *Periptychus* and our observations are based on a composite of numerous specimens that each preserve slightly different portions of the anatomy, and as such they are tentative.

The ventral border of the lacrimal contacts the maxilla via a transversely concave suture above the posterior opening of the infraorbital canal. The lacrimal does not make any contribution to the orbital floor. An ovoid lacrimal foramen forms a large posterolaterally directed opening situated just inside the orbital rim and fully within the lacrimal, albeit only just within the lacrimal. The lacrimal foramen in *Periptychus* is relatively small in comparison to *Arctocyon*, in which it forms a much larger opening in the anterior orbit. In *Periptychus* a groove runs posteriorly from the ventral edge of the lacrimal foramen along the dorsal surface of the zygomatic arch, terminating at approximately the same mediolateral level as the orbital floor. A second lacrimal foramen is not evident on the specimens observed; however, given the poor preservation of the lacrimal region and second foramen cannot be ruled out at present. A small lacrimal tubercle is positioned on the antorbital rim dorsal to the lacrimal foramen.

#### Jugal

The jugal forms the robust mid-section of the zygomatic arch and is well known for *Periptychus*. The following description is based on NMMNH P-36631, which preserves almost the entire right jugal and the posterior half of the left jugal (Fig 4); NMMNH P-19482, which preserves the anterior portion of the left and right jugals where they contact the maxillae (the sutures on this specimen are particularly well preserved) (Fig 4); and AMNH 3665, which preserves part of the left jugal which has been reconstructed to form a complete zygomatic arch. Comparisons are made to *Carsioptychus coarctatus* (AMNH 27601 figured in [5]); *Ectoconus ditrigonus* (AMNH 16500, figured in [5]); *Arctocyon primaevus* (MNHN.F.CR700, figured in [52] and MNHN BR L9) and *Pantolambda bathmodon* (AMNH 16663, figured in [5]).

In *Periptychus* the jugal extends from the anterior border of the zygomatic arch, where it contacts the maxilla and lacrimal, posteriorly to where it terminates on the ventral surface of the squamosal. The anterior portion of the jugal is dorsoventrally thick and mediolaterally compressed. The medial surface of the anterior portion of the jugal is shallowly excavated, giving the zygomatic arch a slightly bowed profile in cross section. The anterior portion of the jugal bifurcates into an anterodorsal long process and a ventrally displaced short process. The anterodorsal long process of the jugal is massively expanded so that is occupies the entire lateral side of the anterior root of the zygomatic arch; anteriorly it extends to the same level as the apex of the paracone on the ultimate upper premolar. At its anterior-most border it forms a convex contact with the maxilla. On the ventral surface of the anterior root of the zygomatic arch the jugal shallowly interdigitates with the maxilla. The short process of the jugal is completely contained on the ventral surface of the arch and forms a stunted protrusion into the maxilla directed towards the first upper molar. The morphology of the anterior portion of the jugal of *Periptychus*, and its relationship with the maxilla, is notably different from the condition exhibited by *Arctocyon* and *Pantolambda*. In *Arctocyon* and *Pantolambda* the jugal is bifurcated; however, the long process is not as massively expanded on the lateral surface of the anterior root of the zygomatic arch and the short process is not as ventrally displaced as the condition observed in *Periptychus*.

In *Periptychus* the jugal contacts the maxilla along the dorsal surface of the zygomatic arch and delimits the lateral edge of the orbital floor. The maxilla extends posteroventrally along the medial surface of the jugal to a point just posterior to where the squamosal overlies the jugal dorsally. On the anterodorsal surface of the zygomatic arch (within the orbit) the jugal maintains a small transverse contact with the lacrimal. In *Arctocyon*, and to a lesser extent in *Pantolambda*, the jugal maintains a larger contact with the lacrimal, which extends onto the facial region due to the expansion of facial process of the lacrimal.

Posteriorly, the jugal of *Periptychus* does not bifurcate; it exhibits a long tetrahedral shaped posteroventral process that contacts the ventral surface of the squamosal on of the zygomatic arch. The jugal-squamosal contact runs horizontally from the dorsal edge of the mid-zygomatic arch to the ventral edge of the squamosal. The posterior portion of the jugal of *Periptychus* is not as robust as that in *Ectoconus* but does appear to form a relatively more elongate contact with the squamosal. In *Periptychus* the posterodorsal-most portion of the jugal is distinctly flattened to form a mediolaterally broad contact with the squamosal. This feature is also present in *Carsioptychus* and *Ectoconus*, but absent in *Arctocyon* and *Pantolambda*. In *Periptychus*, the posterior-most end of the jugal extends to the anteromedial edge of the glenoid fossa, the point of which is marked by a small eminentia articularis. The medial surface of the posterior jugal is deeply excavated, forming a glenoid process on the jugal that is continuous with the glenoid fossa on the squamosal. A similar glenoid process on the jugal is observed in *Carsioptychus* and *Ectoconus*, but absent in *Arctocyon* and *Pantolambda*.

In *Periptychus*, the lateral surface of the jugal is highly sculpted so that in cross section it forms an equilateral trapezium-shaped profile. The lateroventral surface of the jugal tapers into a sharp crest ventrally and provides attachment for the superficial and deep masseter muscles. The lateroventral surface tapers anteriorly and is separated from the deep ovoid fossa on the ventral surface of the anterior root of the zygomatic arch by a small ridge. *Pantolambda* exhibits a similarly sculpted jugal surface to that observed in *Periptychus*, whereas in *Carsioptychus*, *Ectoconus* and *Arctocyon* the lateral surface of the jugal is more rounded with less distinct muscle attachment areas.

#### Squamosal

The paired squamosals of *Periptychus* are extensive: they form a large plate on the posteroventral wall of the braincase, laterally they extend out to form the posterior arm and articular region of the zygomatic arch, and posteriorly they contribute to the auditory region on the posteroventral surface of the basicranium. This description is based on several specimens preserving different portions of the squamosal, as a complete squamosal is unknown for *Periptychus*. NMMNH P-36631 preserves the zygomatic process of the squamosal in several fragments, including the right mandibular articulation surface and the squama (Fig 4). NMMNH P-35194 is a near complete but highly concreted braincase preserving the lateral walls of the squamosals with rudimentary features of the tympanic process of the squamosal discernible on the left side of the braincase. NMMNH P-30684 is a portion of the left side of the braincase preserving a section of the lateral wall and the tympanic process (Fig 6). NMMNH P-30731 is a small piece of the right braincase preserving the region around the external acoustic meatus, including part of the squama and tympanic process of the squamosal. NMMNH P-65619 is portion of the posterior braincase preserving the left and right lateral walls of the squamosal and the right tympanic process of the squamosal (Fig 5). AMNH 3669 is a crushed braincase; the dorsal surface is poorly preserved and retains only fragments of the lateral braincase, but the medial most region of the zygomatic and tympanic processes of both the left and right squamosals are well preserved. Comparisons are made to *Carsioptychus coarctatus* (AMNH 27601 figured in [5]); *Ectoconus ditrigonus* (AMNH 16500, figured in [5]); *Arctocyon primaevus* (MNHN.F.CR700, figured in [52] and MNHN BR L9) and *Pantolambda bathmodon* (AMNH 16663, figured in [5]).

The lateral portion of the squamosal in *Periptychus* is expansive and can be divided into two gross portions: the squama contributes to the lateral wall of the braincase anterior to the glenoid fossa, and the posterior process is located posterior to the glenoid fossa. In lateral aspect, the squama and posterior process form an elongate subquadrilateral plate on the lateral wall of the posterior braincase. The squama of *Periptychus* is poorly known. In the specimens observed, it is apparent that it extends well into the orbital region and appears to overlay the alisphenoid. The posterior process of the squamosal extends to the posterior edge of the braincase where it contacts the pars mastoidea of the petrosal and contributes to the ventral portion of the nuchal crest. The squamosal is inhibited from contacting the supraoccipital on the posteroventral border of the braincase due to the dorsal expansion of the pars mastoidea of the petrosal on the posterolateral corner of the braincase. The condition of *Periptychus* differs from that observed in *Carsioptychus*, *Ectoconus*, *Arctocyon* and *Pantolambda*, in which the squamosal maintains contact with the supraoccipital on the dorsal surface of the braincase due to the comparatively limited expansion of the pars mastoidea (or in the case of *Pantolambda*, the dorsal expansion of the squamosal).

Dorsally, the squama and posterior process of the squamosal of *Periptychus* form an arcuate suture which overlies the parietal and forms the ventral half of the lateral wall of the braincase. The condition in *Periptychus* closely resembles that of *Pantolambda*, whereby the squamosal is expansive and subquadrilateral; however, in *Pantolambda* the squamosal is more dorsally expanded, covering three quarters of the of the lateral braincase. This is markedly different from the condition in *Arctocyon* where the squama and posteriorprocess (= caudal-process) barely extend over a third of the dorsoventral height of the braincase (the posteriorprocess is highly reduced) and the dorsal border of the bone forms a sigmoidal profile where it overlays the parietals (which are massively expanded).

The surface of the posterior process of the squamosal of *Periptychus* is punctured by numerous foramina of varying size, with the number of foramina variable across individuals. NMMNH P-30684 and NMMNH P-35194 exhibit six large foramina above the posterior root of the zygomatic arch and anterodorsal to the external acoustic meatus. The two largest foramina open anteriorly and are positioned on the posterodorsal border of the squamosal. The body of the foramina are within the parietal, with the dorsal edge of the posterior process of the squamosal contributing to the ventral border of the openings. These two openings are interpreted as the rami temporalis of the stapedial artery [81]. Numerous smaller foramina are positioned ventral to the rami temporalis foramina and are interpreted as emissary foramina, most likely for draining the intracranial squamosal sinus, which connects to the superior petrosal sinus [82], rather than nutrient foramina. The nutrient foramina are considerably smaller, forming small, pin-prick sized holes, scattered irregularly over the surface of squamosal and extending onto the posterior root of the zygomatic arch.

The zygomatic component of the squamosal forms the posterior root of the zygomatic arch and the zygomatic processes of the squamosal. The posterior root of the zygomatic arch is massive, with the dorsal surface forming a large, shallowly excavated postorbital shelf dorsally and a large articular surface for the mandible ventrally. The postorbital shelf is triangular in ventral profile and subequal in mediolateral width and anteroposterior depth at its widest points. The condition in *Periptychus* is proportionally similar to that in *Carsioptychus* but differs from *Ectoconus*, in which the dorsal shelf is anteroposteriorly deeper but mediolaterally narrower. The periptychids differ greatly from the condition seen in *Arctocyon* and *Pantolambda*, in which the postorbital shelf is proportionally more robust and anteroposteriorly much deeper. In *Periptychus* the anterior border of the postorbital shelf is shallowly concave, forming a smooth, near continuous surface with the midportion of the zygomatic arch. *Carsioptychus* and *Ectoconus* differ from *Periptychus* as their anterior borders form a near straight margin that is perpendicular to the sagittal axis of the skull. *Arctocyon* and *Pantolambda* also possess a straight anterior border, but it is set at an oblique angle to the sagittal axis of the skull, forming an obtuse angle posteriorly (100–110°). In *Periptychus* the posterolateral border of the shelf is delimited by the crista supramastoideus, which forms the dorsal ridge of the zygomatic process of the squamosal. The crest is thin but prominent, forming a high wall around the posterior border of the dorsal shelf. It is well developed in *Periptychus* but not as salient as in *Arctocyon* and *Pantolambda*, where it thickens at the posterolateral corner of the zygomatic arch.

A large foramen is positioned on the dorsum of the posterior root of the zygomatic arch; it opens anterodorsolaterally and appears to communicate with a large internal opening visible on NMMNH P-36631. The internal opening is positioned anteromedial to the postglenoid foramen and opens onto a ventromedially directed surface at an approximately 90° angle to the surface of the basicranium. We interpret this foramen as being homologous with the ‘supraglenoid foramen’ as identified by Cope [83], which appears to be an ungulate feature found in both artiodactyls and perissodactyls. A similarly positioned foramen, albeit somewhat smaller, is observed in *Carsioptychus* and *Arctocyon*. A supraglenoid foramen is not visible in *Ectoconus* (AMNH 16500) or *Pantolambda* (AMNH 16663), but this could be due to poor preservation rather than true absence.

The zygomatic process of the squamosal is anteriorly expanded, contributing a large portion of bone to the zygomatic arch, which forms a long horizontal contact along 70% of the dorsal surface of the jugal. In dorsal aspect, the dorsal edge of the zygomatic process of the squamosal forms a sharp crista supramastoidea. A small notch-like shelf separates the crista supramastoidea from the lateral wall of the braincase, so the crista supramastoidea is not quite continuous with the post-temporal crest [66] (= postzygomatic crest; [82]). The post-temporal crest is salient, forming an anteriorly tilted shelf over the external acoustic meatus. Posteriorly the shelf is not quite continuous with the squamosal-petrosal suture. A similar condition is observed in *Carsioptychus* and *Ectoconus*, although the shelf is not pitched at an oblique angle as seen in *Periptychus*. A post-temporal crest is present in *Arctocyon* and *Pantolambda* but is not as prominent as in the periptychids and forms only a small, horizontal shelf above the external acoustic meatus.

The ventral surface of the zygomatic process of the squamosal forms the articular surface of the temporomandibular joint. The articular surface of the glenoid fossa is positioned on the squamosal; laterally the fossa is continuous with the glenoid process of the jugal. This condition is also evident in *Carsioptychus* and *Ectoconus* and differs from that in *Arctocyon* and *Pantolambda*, in which the glenoid fossa is positioned entirely on the squamosal. The glenoid fossa of *Periptychus* is shallowly excavated, forming a relatively flat, pyriform shaped fossa that is mediolaterally broader than anteroposteriorly long. At its widest, the mediolateral width of the fossa is approximately twice the anteroposterior depth. The condition of *Periptychus* (and also *Carsioptychus* and *Ectoconus*, which are broadly similar) is notably different from that of *Arctocyon* and *Pantolambda*. In *Arctocyon* the glenoid fossa is massive, forming a deep, saddle-shaped articular surface for the mandibular condyle, the ventral profile of the fossa is ovoid and the anterior border of the fossa is demarcated by a thick rim so that the articular surface is raised relative to the surrounding squamosal. *Pantolambda* differs from both *Periptychus* and *Arctocyon* in that the glenoid fossa extends medially onto the lateral edge of the basicranium, with the medial portion of the fossa forming a deeply excavated depression that is subtly differentiated from its lateral counterpart (as opposed to forming a smooth continuous surface as seen in *Periptychus* and *Arctocyon*).

In *Periptychus* a small eminentia articularis marks the lateral border of the glenoid fossa on the squamosal where it meets the jugal. The posterior border of the glenoid fossa is delimited by a small and thin, but distinct, postglenoid process. The postglenoid process is positioned on the posteromedial edge of the fossa and forms a pyramidal-shaped ventral projection with no anterior inclination. The anterior surface of the process is subvertical and continuous with the glenoid fossa and served to stabilize the mandible. In posterior aspect, the postglenoid process is roughly ‘U” shaped and near symmetrical. The overall morphology of the postglenoid process of *Periptychus* (as well as *Carsioptychus* and *Ectoconus*) is broadly similar to that of *Pantolambda*. However, *Pantolambda* differs in being anteroposteriorly more robust, and the entire process has shifted medially so that it is situated on the basicranium, anterior to the external acoustic meatus as opposed to on the zygoma as in *Periptychus* and *Arctocyon*. The postglenoid process of *Arctocyon* is distinctly different from that of *Periptychus*; it is massive, forming a mediolaterally expanded wall along the entire posterior margin of the glenoid fossa.

The postglenoid foramen of *Periptychus* forms a small, ventrally directed opening just posteromedial to the apex of the postglenoid process; a shallow sulcus runs from near the apex of the postglenoid process to the lateral edge of the foramen. In life, the postglenoid foramen would have transmitted the capsuloparietal vein; in *Periptychus* this opening is somewhat variable in size, but is consistently proportionally smaller than the corresponding foramen in *Carsioptychus*, *Ectoconus*, *Arctocyon* and *Pantolambda*. The posteromedial margin of the postglenoid foramen is marked by a small postglenoid eminence [69]. The eminence in *Periptychus* is formed by a prominent right angle of bone positioned on the posteromedial edge of the postglenoid foramen. It also contributes to the posterolateral corner of the Glaserian fissure (= petrotympanic fissure) and the dorsal roof of the external acoustic meatus. The condition of *Periptychus* is more pronounced than in *Pantolambda*, where the eminence is less salient, but still distinct, and *Arctocyon*, where the eminence is indistinguishable relative to the surrounding bone.

The medial edge of the glenoid fossa is delimited by a well-developed Glaserian fissure which transmitted the chorda tympani. The fissure forms a discrete anteroposteriorly directed sulcus that is continuous with the epitympanic recess posteriorly; anteriorly the fissure provides communication with the temporomandibular joint. The walls of the sulcus are high; the lateral wall is formed by the medial boundary of the glenoid fossa and the medial wall is formed by the entoglenoid process of the squamosal. A well-defined Glaserian fissure is present in *Carsioptychus* and *Ectoconus* as a deep high-walled sulcus. In *Pantolambda*, the Glaserian fissure is present in the same position as *Periptychus*, but is much shallower with poorly defined, low lying walls. In *Arctocyon*, the fissure is present but distinctly different from the condition exhibited by periptychids and *Pantolambda*. Anteriorly the fissure is represented by a mediolaterally broad notch between the postglenoid process laterally and the entoglenoid processes medially. Posteriorly the fissure becomes less distinct; the medial boundary between the fissure and entoglenoid process and the lateral boundary formed by the postglenoid eminence are indistinct, resulting in an almost smooth plate anterior to the auditory region, with only a shallow contour on the surface of the bone marking the placement of the Glaserian fissure.

In *Periptychus*, the entoglenoid process of the squamosal marks the medial extent of the squamosal on the basicranium. The process is well developed, forming an elevated plateau on the posterolateral margin of the glenoid fossa, with the long axis orientated obliquely to the sagittal plane from anteromedial to posterolateral. The process is separated from the glenoid fossa by the Glaserian fissure and positioned medial to the parasagittal plane of the postglenoid foramen. The process is large and ventrally prominent so that it is roughly subequal in the size to the postglenoid process, forming a high wall on the posteromedial border of the glenoid fossa. In life, this would have provided support to the mandibular condyle when fully occluded in the glenoid fossa. In ventral aspect, the entoglenoid process is sub-triangular; the lateral edge forms a sharp wall along the medial edge of the Glaserian fissure, and the medial edge contacts the alisphenoid within a narrow but deep channel. The channel extends anteriorly from the auditory region to a large ovoid foramen, which we infer to be the foramen ovale. The medial wall of the channel is formed by the preotic crest on the alisphenoid. The entoglenoid process of *Periptychus* is similar in morphology to that of *Pantolambda*; however, the condition in *Periptychus* is substantially more developed than the inconspicuous process of *Pantolambda*. The process of *Periptychus* is mediolaterally broader and ventrally elevated relative to the surrounding bone, whereas the process of *Pantolambda* is not elevated relative to the surrounding bone and is much smaller relative to the size of its postglenoid process. The entoglenoid morphology of *Arctocyon* is notably different from that of *Periptychus* and *Pantolambda*, as previously described, in relation to the Glaserian fissure. Anteriorly the process is prominent, but it does not form an elevated plateau as in *Periptychus*; posteriorly, the borders between the entoglenoid process, the Glaserian fissure laterally and alisphenoid medially are indistinct.

Anterior to the entoglenoid process, the squamosal contacts the alisphenoid to form an anteroposteriorly aligned suture along the margin of the posterior root of the zygomatic arch, lateral to the foramen ovale. Anterolateral to the foramen ovale, the suture turns and ascends the braincase dorsally to form the squama of the squamosal.

Posteriorly, the squamosal contributes to the lateral-most portion of the auditory region. Anteriorly the squamosal-petrosal contact is clearly delimited between the Glaserian fissure and the epitympanic recess (on AMNH 3669 the bone appears to have fractured along this suture). From the posterolateral corner of the Glaserian fissure, the squamosal-petrosal suture runs posteriorly within the epitympanic recess. In *Periptychus* the suture runs anteroposteriorly along (but not quite touching) the lateral edge of the recess rather than running anteromedially to posterolaterally, cutting through the middle of the recess, as in *Pantolambda*. Lateral to the squamosal-petrosal suture, the squamosal forms a high, rounded lateral wall for the epitympanic recess, which corresponds to the dorsal edge of the external acoustic meatus.

The dorsal edge of the external acoustic meatus forms a thick, blunt ridge positioned ventrally relative to the floor of the epitympanic recess, rather than a low lying, sharp crest as in *Pantolambda*. Lateral to this point the squamosal forms the arcuate roof of the external acoustic meatus. The external acoustic meatus of *Periptychus* is mediolaterally deep, so that the petrosal bone is situated well within the basicranium. The condition of *Periptychus* is like *Pantolambda*, in which the external acoustic meatus is even longer due to the medial invasion of the glenoid fossa onto the basicranium. The broadly similar conditions of *Periptychus* and *Pantolambda* differ greatly from that of *Arctocyon*, where the petrosal is situated near the lateral border of the basicranium and possesses a mediolaterally short external acoustic meatus.

In lateral aspect, the roof of the external acoustic meatus is highly arched, with the postglenoid process forming the anterior wall and the post-tympanic process of the squamosal forming the posterior wall. Anteriorly, the external rim of the acoustic meatus is formed by the post-temporal crest (which extends posterodorsally from the zygomatic arch), whereas the posterior portion of the external rim of meatus is formed by a less salient suprameatal crest, which diverges from the post-temporal crest midway over the arch of the meatus. The development of the suprameatal crest in *Periptychus* is variable across specimens and tends to be more salient in smaller individuals.

In *Periptychus*, the suprameatal surface of the external acoustic meatus faces ventrolaterally and is excavated by a moderately deep suprameatal fossa, so that the dorsal roof of the external acoustic meatus is set at a 35° oblique angle relative to the basicranium, with the medial portion positioned ventrally relative to the more dorsal lateral portion. There is no evidence of a suprameatal foramen. The roof of the external acoustic meatus of *Periptychus* features a stronger vertical component and is more dorsally excavated than the condition in *Pantolambda*, where the dorsal surface is set at approximately a 15° oblique angle relative to the basicranium and is not excavated by a suprameatal fossa. The vertical component of *Periptychus* is not as strong as in *Arctocyon*, in which the roof forms a steep vertical slope over a much shorter mediolateral distance.

The posterior wall of the external acoustic meatus is formed by the post-tympanic process of the squamosal. The process forms a small protuberance which is tightly appressed against the anterior surface of the mastoid process. In lateral view, the post-tympanic process closely approximates the postglenoid process in size and projects to the same ventral extent. In *Periptychus*, the contribution of the squamosal to the posterolateral corner of the basicranium is limited to a small portion on the anterior surface of the mastoid process. This is contrary to the condition in *Arctocyon* and *Pantolambda*, in which the post-tympanic process forms a thin plate that wraps around the mastoid process. In the case of *Pantolambda*, the post-tympanic process surrounds the lateral surface of the mastoid process and contacts the paraoccipital. In *Arctocyon*, the post-tympanic process is more restricted and only extends on to the lateral surface of the mastoid process.

#### Frontal

The paired frontal bones of *Periptychus* form the midsection of the skull roof and contribute to the walls of the braincase within the orbital region. The following description is based on a collection of fragmentary specimens; a complete specimen preserving the orbital process of the frontal is still unknown. NMMNH P-19482 preserves the dorsal-most portion of the frontal on the skull roof, but not any part of the orbital processes (Fig 3). NMMNH P-35194 and NMMNH P-36631 (Fig 4) preserve equivalent portions of the posterior frontal on the dorsal surface of the skull. AMNH 3665 preserves fragments of the dorsal and lateral processes of the frontal which have been reconstructed. Comparisons are made to *Carsioptychus coarctatus* (AMNH 27601 figured in [5]); *Ectoconus ditrigonus* (AMNH 16500, figured in [5]); *Arctocyon primaevus* (MNHN.F.CR700, figured in [52] and MNHN BR L9); *Claenodon ferox* (NMMNH P8627 figured in [78]) and *Pantolambda bathmodon* (AMNH 16663, figured in [5]).

The paired frontal bones of *Periptychus* are mediolaterally expanded and form a broad, flat roof over the anterior orbit. Anteriorly the frontals contact the nasals along the sagittal midline of the skull, anterolaterally they contact the maxillae and the lacrimals and posteriorly they contact the parietals. Based on comparisons to other taxa, we can assume that, within the anterior orbit, the frontals likely contacted the palatine anteroventrally, the orbitosphenoid ventrally and the alisphenoid posteroventrally; however, the nature of these contacts remains unknown.

In dorsal aspect, the paired frontal bones exhibit a faint metopic suture visible along the sagittal midline of the skull. The anterior frontal region is greatly expanded and forms a mediolaterally transverse contact over the nasals; as discussed in the nasal section, it is not possible to definitively determine the nature of the nasofrontal suture, but we tentatively postulate that *Periptychus* exhibited a short posterior projection of the nasals into the frontal region. The maxillary process of the frontal is not preserved, but the surrounding elements allow for tentative inferences about the morphology. Based on the size and configuration of the known elements, we infer that anteriorly maxillary process of the frontal form a substantial contact with the maxilla due to the robust depth of the posterior region of the maxilla and the highly reduced facial process of the lacrimal (the posterior expansion of the nasals limits the dorsal extent of the contact). The maxilla-frontal contact is positioned at the base of the rostrum, but is restricted from extending into the orbital region by the lacrimal. A similar condition is seen in *Ectoconus*, providing further support to our interpretation. In *Arctocyon*, the maxillary process of the frontal is large and mediolaterally broad due to the relatively narrow nasals.

The supraorbital region of *Periptychus* is transversely inflated, forming a broad flat surface at the base of the rostrum. The condition of *Periptychus* is accentuated by the narrowness of the posterior portion of skull, but is not as swollen as the condition in *Arctocyon* and *Claenodon*. In these taxa, the combination of the transverse anterior frontal region and extensive facial process of the lacrimal results in a very broad, flat face across the supraorbital region. This is somewhat mediated by, when viewing the entire skull, the anteroposterior length of the skull and elongation of the cranium, but is still proportionally more inflated and bulbous in comparison to *Periptychus*. The postorbital process of *Periptychus* is highly reduced, forming little more than an angular protuberance on the dorsal rim of the orbit. In AMNH 3665, the postorbital protuberance has been reconstructed in a position that is far too posterior. Based on NMMNH P-19482, the process should be placed dorsal to the orbital floor, as seen in all the other comparison taxa. There is no evidence of a supraorbital foramen or notch in *Periptychus*. The postorbital processes of *Arctocyon* and *Claenodon* are larger than in *Periptychus*, but in both taxa, form a rounded swelling that is most prominent just posterior and dorsal to the orbital floor.

The frontals narrow posteriorly but remain relatively broad where they contact the parietals. The supraorbital ridges (= temporal lines) are moderately high but remain broadly rounded, and extend from the postorbital processes to the posterior margin of the frontal. Posteriorly they form a continuous ridge with the anterior processes of the parietals, which then converge on the parietals to form the sagittal crest. The temporal lines of *Periptychus* are not as sharp in *Pantolambda*, where they form a distinct line rather than a broadly rounded ridge. The temporal lines of *Periptychus* are also widely spaced on the frontal, more widely than in *Ectoconus* and *Pantolambda*, so that they bear more resemblance to *Arctocyon*. The paired posterior processes of the frontals project into the parietals along the sagittal midline between the anterior processes of the parietal. The posterior processes of *Periptychus* form a narrow, but elongate triangular projection extending approximately 25% of the length of the sagittal crest. In *Arctocyon* and *Pantolambda*, the posterior processes are mediolaterally broad but do not form an elongate projection into the parietal region as in *Periptychus*, *Carsioptychus* and *Ectoconus*. The orbital processes of the frontal that lie ventrolateral to the temporal line remain unknown and no foramina are visible on the specimens observed.

#### Parietal

The parietals form the bulk of the posterolateral and dorsal braincase and house the cerebral hemispheres. The following description is based on an assortment of specimens preserving the entire parietal region. NMMNH P-19482 preserves the dorsal half of both parietals and the sagittal crest, which is near complete and undistorted (Fig 3). NMMNH P-36631 preserves the anterior portion of the dorsal parietals, but most of the dorsal region and the sagittal crest are missing; however, the specimen does preserve the contact between the squamosal and parietal in good detail (Fig 4). NMMNH P-35194 is an almost complete posterior braincase preserving both parietal bones; however, the specimen has been crushed and is somewhat concreted obscuring the finer anatomical details. NMMNH P-65619 is a posterior portion of the braincase; it is somewhat dorsoventrally crushed and concreted but preserves the entire posterior cranium and general anatomical details reasonably well (Fig 5).

The paired parietal bones form the dorsolateral walls of the braincase, and the dorsal suture between the two parietal bones is marked by a prominent sagittal crest. Anteriorly the parietals overlie the frontal, laterally they underlie the squama and posterior processes of the squamosal and posteriorly they contact the occipital complex.

Anteriorly the parietals contact the frontals. Based on NMMNH P-19482 and AMNH 3665, it appears that the anterior contact between the parietals and frontals remains posterior to the coronal plane set by the distal surface of the ultimate upper molar. The lateral sutures between the parietals and frontals are not extensively preserved nor well defined, but it appears the parietals slightly overlie the frontals on the lateral surface of the braincase. On the dorsal surface, the anterior processes of the parietals diverge within the parietal region, separated by the posterior processes of the frontal. Anteriorly, the anterior processes of the parietal become mediolaterally broader and more rounded so that they are near continuous with the supraorbital ridges on the frontal. There is some variability in the anterior extent of the sagittal crest and the extent of the posterior processes of the frontal. On NMMNH P19482 the anterior processes of the parietal are separated by an elongate posterior frontal process so that they do not fully converge into a sagittal crest until nearly half way into the parietal region, whereas in NMMNH P-35194 the anterior processes converge within the anterior third of the parietal.

The sagittal crest of *Periptychus* is robust and provides an anteroposteriorly extensive and dorsoventrally deep attachment area along the length of the braincase for the temporalis muscles. In lateral aspect, the dorsal border of the sagittal crest remains relatively low in relation to the dorsal surface of the facial and rostral processes of the skull. *Carsioptychus* and *Ectoconus* display a similar condition whereby the sagittal crest is well developed but projects only just dorsal to the dorsal surface of the facial and rostral processes. In *Pantolambda* the sagittal crest is proportionally comparable to the periptychids; however, it sits on a higher braincase and as such forms a more prominent crest on the dorsal surface of the skull. In *Arctocyon* the sagittal crest is massive, forming a prominent crest on the dorsal surface of the skull which combined with a small, low lying braincase amounts to a massive crest with a dorsoventrally extensive attachment area for the temporalis muscles. In *Periptychus* the sagittal crest is present and well developed on all specimens observed; however, relative development across individuals is variable, suggesting an ontogenetic or sexually dimorphic control on its development.

In lateral aspect, the parietals form the lateral walls of the posterior braincase in conjunction with the squama and posterior processes of the squamosal. The braincase is small and dorsoventrally compressed so that, when excluding the sagittal component, the braincase lies lower than the frontal region of the skull. In dorsal aspect, the walls of the braincase are subtly convex and mediolaterally broadest at the posterior root of the zygomatic arch. *Carsioptychus*, *Ectoconus* and *Arctocyon* all exhibit a similar morphology; however, *Pantolambda* differs drastically, in that the braincase increases in mediolateral width posteriorly giving the skull an unusual hourglass profile in dorsal aspect. In *Periptychus* the braincase is mediolaterally narrower than the facial process of the skull and shows a marked constriction along the coronal plane where the frontal contacts the parietal. This constriction is evident to roughly the same degree in *Carsioptychus*, *Ectoconus* and *Pantolambda* but is noticeably more marked in *Arctocyon*. Further to this, in *Ectoconus* and *Arctocyon* the mediolateral width of the braincase is subequal to the mediolateral width of the facial process, whereas in *Pantolambda* the braincase is mediolaterally broader than the facial process (*Carsioptychus* exhibits a morphology similar to *Periptychus* in that the facial process is mediolaterally broader than the braincase). Posteriorly, the braincase of *Periptychus* forms a substantial protrusion beyond the posterior edge of posterior root of the zygomatic arch. *Carsioptychus* and *Ectoconus* exhibit a similar condition. In *Arctocyon* the protrusion is more elongate than in the periptychids, but not as mediolaterally broad, and in MNHN.F.CR700 the posterior braincase shows a marked tapering towards the supraoccipital. *Pantolambda* exhibits a very different morphology in that the braincase does not protrude particularly far beyond the posterior edge of the posterior root of the zygomatic arch, but instead forms a broad contact with the supraoccipital, which is almost vertical in lateral view.

In *Periptychus* the parietal is exposed on the dorsal half of the lateral wall of the braincase and is punctured by at least two large foramina for the rami temporalis of the stapedial artery [81]. The foramina are situated along the arcuate squamosal-parietal suture with the dorsal border of the squamosal contributing to the ventral border of the opening. The lateral contribution of the parietal to the braincase of *Periptychus* is more extensive than in *Pantolambda*, where the squamosal is dorsally expanded, and *Arctocyon*, where the braincase is reduced due to the extreme development of the sagittal crest. Anteroventrally the parietals contact the alisphenoid, but the nature of this suture remains unknown at present. Posteriorly the parietal contacts the supraoccipital on the lateral walls of the braincase. The posteroventral portion of the parietal underlies the supraoccipital along the lambdoid suture, anterior to the nuchal crest. The dorsoventral expansion of the posterior portion of the parietal excludes the squamosal from contacting the supraoccipital (in conjunction with the expansion of the pars mastoidea on the posterolateral surface of the braincase). The posterodorsal portion of the parietal contacts the supraoccipital where the sagittal crest converges on the nuchal crest. This condition is also evident in *Carsioptychus*, *Ectoconus* and *Arctocyon*; however, it appears that in all three taxa the squamosal maintains a small contact with the supraoccipital on the dorsal surface of the braincase due to the limited expansion of the pars mastoidea on the posterolateral corner of the braincase. The condition of *Pantolambda* is broadly similar in that the parietals contact the supraoccipital along the lambdoid suture anterior to the nuchal crest; however, the contact between the parietal and supraoccipital is limited by the dorsal expansion of the squamosal, resulting in a dorsoventrally extended contact between the squamosal and supraoccipital.

#### Pterygoid

**Fig 7:**
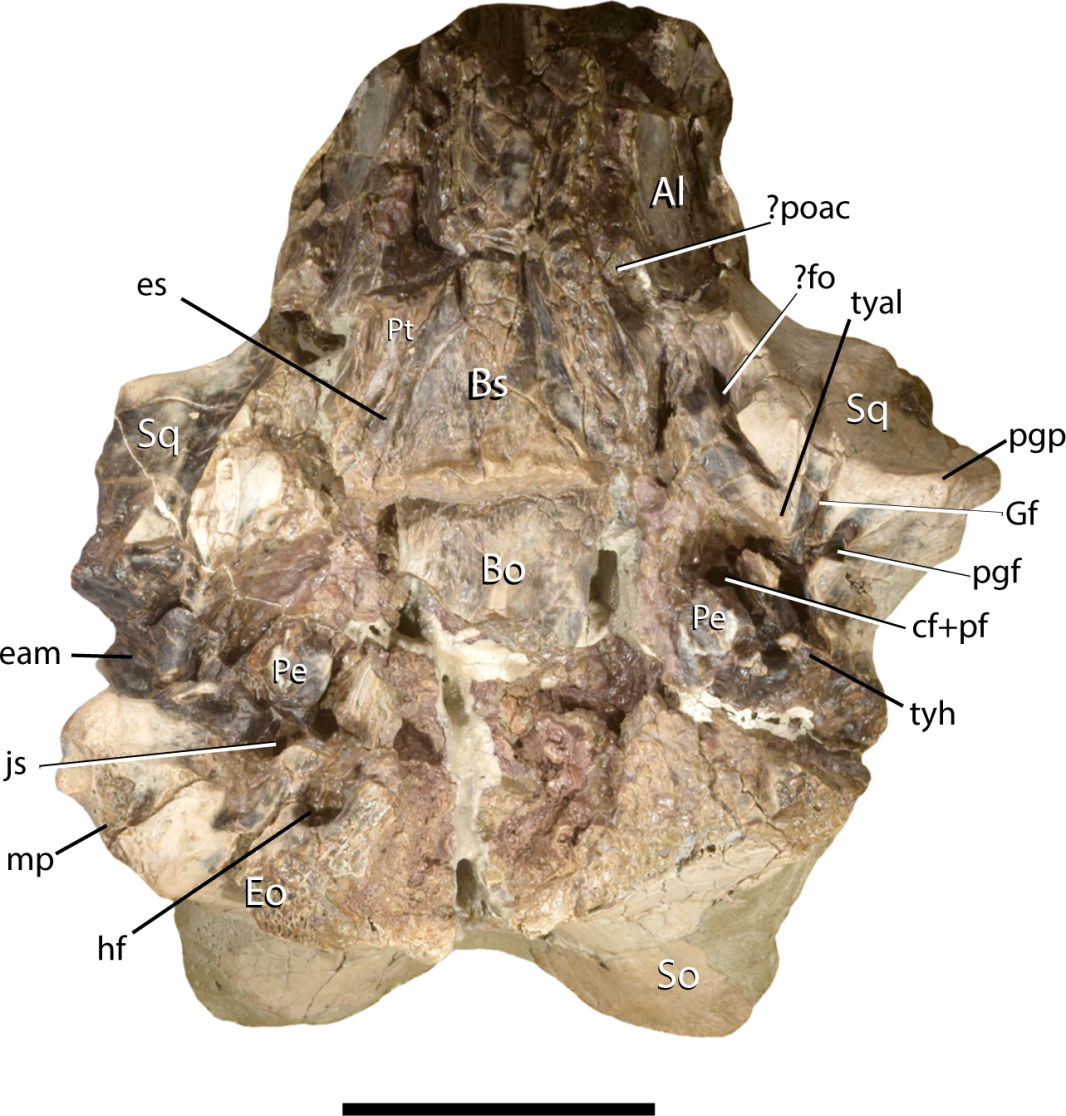
Basicranium of *Periptychus carinidens* (AMNH 3669) in dorsal view. Abbreviations: Al, alisphenoid; Bo, basioccipital, Bs, basisphenoid; cf, carotid foramen; eam, external acoustic meatus; Eo, exoccipital; es, Eustachian sulcus; fo, foramen ovale; Gf, Glaserian fissure; hf, hypoglossal foramen; js, jugular sulcus; mp, mastoid process; pe, petrosal; pf, pyriform fenestra; pgf, postglenoid foramen; pgp, postglenoid process; poac, posterior opening of the alisphenoid canal; pt, pterygoid; Sq, squamosal; tyal, tympanic process of the alisphenoid; tyh, tympanohyal; ?, denotes uncertainty of label. Scale bar: 30mm.

The fragile paired pterygoid bones are not well known for *Periptychus*. The following description is predominantly based on AMNH 3669, a posterior braincase preserving the basicranium. Portions of pterygoid are visible on this specimen, but the preservation is poor and the sutures between the bone elements are not clearly delimited (Fig 7). NMMNH P-19482 is subcomplete skull preserving fragmentary pieces of the entopterygoid processes (Fig 3). NMMNH P-36631 is a partial skull preserving a small piece of the basicranium which includes posterior fragments of the entopterygoid processes in association with the basisphenoid, alisphenoid and basioccipital (Fig 4). Reference is also made to the braincase of NMMNH P-35194; the basicranium of this specimen is highly concreted, but the position of the paired entopterygoid processes is observable. Comparisons are made to *Carsioptychus coarctatus* (AMNH 27601 figured in [5]); *Arctocyon primaevus* (MNHN.F.CR700, figured in [52] and MNHN BR L9) and *Pantolambda bathmodon* (AMNH 16663, figured in [5]).

The pterygoid is a long, narrow, element positioned on the ventral surface of the skull between the rostrum anteriorly and the basicranium posteriorly. The paired pterygoids are separated by the basicranial elements of the sphenoid complex and contribute to the roof of the basipharyngeal canal. The lateral entopterygoid processes of the pterygoid form the parasagittal walls of the basipharyngeal canal and extend posterolaterally towards the auditory region, terminating at the approximate transverse level of the foramen ovale.

The anterior portion of the pterygoid is poorly known for *Periptychus*. NMMNH P-19482 preserves fragments of the pterygoid and perpendicular process of the palatine, but the suture is obliterated and inhibits any meaningful interpretation. It is not possible to comment on the relationship between pterygoid and presphenoid bone. Posteriorly the basisphenoid separates the pterygoids.

The lateral component of the pterygoid is formed by the entopterygoid process. The entopterygoid processes of *Periptychus* are damaged on all the specimens observed, with the dorsal base of the processes preserved to varying degrees of completeness and the ventral projecting plates, which form the walls of the basipharyngeal canal, only fragmentarily preserved. Based on what is known, it appears the entopterygoid process of the pterygoid is anteroposteriorly extensive, forming a laterally concave plate which extends from the perpendicular process of the palatine to a point that is just medial to the foramen ovale. A small, posteriorly projecting hamular process is evident on the left entopterygoid process on AMNH 3669, but it is incompletely preserved. The lateral edge of the entopterygoid contacts the ectopterygoid process of the alisphenoid anteriorly. It is not possible to compare the relative sizes of the entopterygoid process of the pterygoid with ectopterygoid process of the alisphenoid, but based on anteroposterior length, the entopterygoid process appears to be larger. Mediodorsally, the entopterygoid process forms an anteroposteriorly elongate contact with the basisphenoid along the lateral border of the Eustachian sulcus (= Eustachian tube). Any foramina perforating the pterygoid have been obliterated on the specimens observed.

The lack of an intact pterygoid limits comparisons to other taxa. The condition of *Periptychus* appears to be broadly like that of *Carsioptychus* and *Arctocyon*, whereby the entopterygoid process is anteroposteriorly elongate, extending to the posterior transverse level of the foramen ovale. The process of *Periptychus* is proportionally more elongate than in *Pantolambda;* note that the midsection of AMNH 16663 is damaged and artificially truncated, but even so it does not appear to not appear to be as elongate or transversely narrow as the condition in *Periptychus*.

#### Sphenoid complex

The sphenoid complex of *Periptychus* is composed of four elements which form a significant portion of the basicranium and braincase between the palatines anteriorly and the basioccipital posteriorly. The sphenoid complex includes the presphenoid and basisphenoid on the basicranium; the alisphenoid, which extends from the lateral basicranium into the orbit; and the orbitosphenoid, positioned wholly within the orbit. The sphenoid complex of *Periptychus* is incompletely known; the presphenoid is very poorly known and orbitosphenoid is unknown/not identifiable, the alisphenoid is only partially known and the basisphenoid is known from several fragmentary specimens. The following description is based several cranial specimens preserving various portions of the sphenoid complex. NMMNH P-35194 is a highly concreted braincase preserving the basisphenoid, the alisphenoid and possibly the orbitosphenoid; however, the alisphenoid and orbitosphenoid are highly concreted preventing any meaningful observation. AMNH 3669 is a posterior braincase preserving the bulk of the basisphenoid, part of the presphenoid and the basicranial portion of the alisphenoid (Fig 7). NMMNH P-36631 is a partial skull preserving a small piece of the basicranium, which includes a posterior portion of the basisphenoid with fragments of the alisphenoid in association with the pterygoids and basioccipital (Fig 4). Comparisons will be made to *Carsioptychus* (AMNH 27061, figured in [5]) and *Pantolambda bathmodon* (AMNH 16663, figured in [5]).

The presphenoid is not well known for *Periptychus*, and on AMNH 3669 the borders are poorly delimited. As such, it is not explicitly clear where the posterior border of the presphenoid is positioned, but two potential morphologies can be inferred. The first interpretation suggests a relatively elongate sub-rectangular presphenoid extending along the sagittal axis of the roof of the basipharyngeal canal similar to the condition in *Leptictis* [82]. The second interpretation suggests a relatively short presphenoid due to the anterior extension of the basisphenoid well into the basipharyngeal canal, resulting in a more anteriorly placed presphenoid similar to the condition observed in *Alcidedorbignya* [69] and likely present in *Pantolambda*. In *Periptychus*, the posterior border of the presphenoid could be interpreted to form a near transverse contact with the basisphenoid at a level just anterior to the posterior opening of the alisphenoid canal laterally (contact fractured on AMNH 3669). This would support a *Leptictis-*like morphology. Alternatively, the fracture on AMNH 3669 may not represent the presphenoid-basisphenoid suture and said suture is not observable, implying a more anterior *Alcidedorbignya*-like morphology. Taking into consideration the morphology observed in *Carsioptychus*, it seems that the first interpretation is most likely as both *Periptychus* and *Carsioptychus* exhibit a posteriorly placed, transverse fracture which we infer as the presphenoid-basisphenoid contact.

In *Periptychus*, a longitudinal keel is evident along the anteroposterior midline of the presphenoid. The presphenoid clearly inhibits the paired bones of the lateral walls of the canal (composed from the palatines anteriorly and the entopterygoid processes posteriorly) from contacting each other on the sagittal axis of the skull. The full anterior extent of the presphenoid of is not known. The known morphology would imply a mediolaterally broad presphenoid, in which the lateral contacts are close to the base of the lateral walls of the basipharyngeal canal.

The basisphenoid of *Periptychus* is an unpaired bone situated on the midline of the basicranium between the inferred position of the presphenoid anteriorly and the basioccipital posteriorly. The lateral border of the basisphenoid contacts the pterygoids anteriorly and the alisphenoid posteriorly. In ventral aspect, the basisphenoid is broadly trapezoidal in shape, tapering anteriorly. The posterior suture between the basisphenoid and basioccipital is transverse and does not excavate the basioccipital. The posterolateral corners of the basisphenoid are expanded into flanges which delimit the anterior border of the anterior foramen lacerum. This foramen is a large vacuity containing the carotid notch (= carotid foramen) and appears to be contiguous with the middle foramen lacerum which contains the pyriform fenestra. A similar morphology is observed in *Carsioptychus* and *Pantolambda*, in which the basisphenoid does not completely enclose the carotid notch. In *Periptychus*, the lateral border of the ventral surface of the basisphenoid exhibits a shallowly grooved Eustachian tube which conveyed the pharyngeal vein, artery and nerve anteriorly into the basipharyngeal canal [82].

The orbitosphenoid is not definitively known for *Periptychus*. It may be present on NMMNH P-35194, but the specimen is too concreted to make any meaningful observation. The basicranial portion of the alisphenoid of *Periptychus* is known from fragments. The following description is based on AMNH 3669, which preserves portions of the alisphenoid, although it should be noted that the cranial bones on the specimen have been displaced. In *Periptychus*, the tympanic process of the alisphenoid is present where it contacts the medial edge of the entoglenoid of the squamosal, the lateral edge of which is marked by a distinct preotic crest. The preotic crest is well defined with the medial edge of the entoglenoid overhanging the tympanic process of the alisphenoid to form a distinct sulcus. The sulcus is defined along its entire length and extends anteromedially from the auditory region towards the foramen ovale and posterior opening of the alisphenoid canal. A preotic crest and associated sulcus is present in both *Arctocyon* and *Pantolambda* although it is not as prominent in either taxon as that in *Periptychus* due to the less salient development of the entoglenoid process. The anteroposterior length of the preotic crest of *Arctocyon* is proportionally longer than in *Periptychus* and *Pantolambda*. The posterior tip of the crest extends to the lateral midsection of the auditory region (approximately level with the fenestra cochleae on the promontorium) rather than terminating anterior to the auditory region as in *Periptychus* and *Pantolambda*.

#### Petrosal

**Fig 8:**
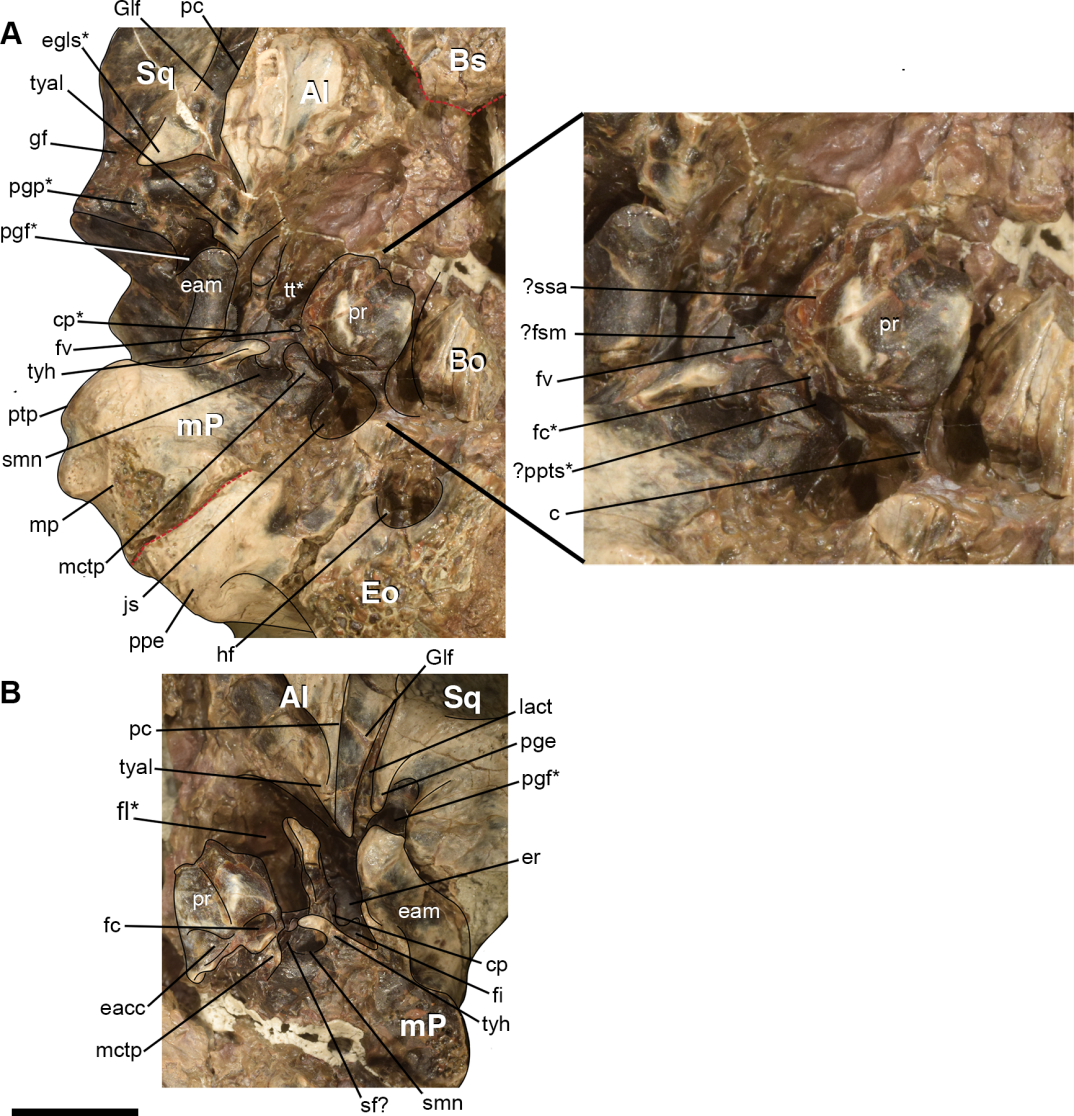
Auditory region of *Periptychus carinidens* (AMNH 3669). (A) The right auditory region of AMNH 3669 with a cut out to annotate finer anatomical details. Major anatomical boundaries are lined. (B) The left auditory region of AMNH 3669. Abbreviations: Al, alisphenoid; Bo, basioccipital, Bs, basisphenoid; c, commissure, cp, crista parotica; eam, external acoustic meatus; egls, entoglenoid process of the squamosal; Eo, exoccipital; er, epitympanic recess; fc, fenestra cochlearis; fi, fossa incudis; fl, foramen lacerum; fv, fenestra vestibuli; gf, glenoid fossa; Glf, Glaserian fissure; hf, hypoglossal foramen; lact, lateral articulation for the anterior crus of the ectotympanic; mctp, medial posterior tympanic process; m(P) mastoid bone of the petrosal; mp, mastoid process; pc, preotic crest; pge, postglenoid eminence; pgf, postglenoid foramen; pgp, postglenoid process; ppe, paroccipital process of the exoccipital; ppts, postpromontorial tympanic sinus, pr, promontorium; ptp, post-tympanic process of the squamosal; sf, sulcus facialis; smn, stylomastoid notch; Sq, squamosal; ssa, sulcus for the stapedial artery; tyal, tympanic process of the alisphenoid; tyh, tympanohyal; *, denotes damage; ?, uncertainty of label. Scale bar: 10mm.

The petrosal of *Periptychus* is preserved on AMNH 3669, a braincase with associated dentition and which includes the left and right auditory apparatus (Figs 7 and 8). The preservation of this specimen is excellent for a larger bodied Paleocene mammal (as petrosals are infrequently well-preserved for such taxa), but there are some significant areas of distortion and notable discrepancies between the placement of features on opposite sides of the basicranium. That said, the specimen allows for the first detailed description of the tympanic region of *Periptychus*.

The skull has been crushed so that very little of the dorsal surface of the braincase is preserved and the left side of the skull has shifted anteriorly relative to the right. The medial components of the basicranium, including the basisphenoid and occipital complex, have shifted relative to the more lateral elements (exposing internal matrix on the surface of the basicranium), and both petrosals have rotated medially by approximately 20°. Fortunately, the left petrosal is reasonably intact; the promontorium is evident (although its posterior-most portion and the mastoid region are damaged), the pars canalicularis is very well preserved and includes the tympanohyal, but the shelf-like portion of the tegmen tympani is broken. The petrosal has shifted counter clockwise relative to the squamosal and alisphenoid, creating an artificially enlarged cavity anteriorly. The basioccipital and exoccipital are present, but poorly defined, creating an artificial cavity on the medial border of the left petrosal. The right petrosal is not as well preserved as the left, but it does preserve features which are not preserved on the left-hand side. The right petrosal has rotated clockwise relative to the squamosal and alisphenoid; the promontorium is somewhat crushed, but its posterior portion and the tympanic processes are intact and the posterior lacerate foramen is clearly defined. The posterior portion of the pars canalicularis and the mastoid region are better preserved than on the left petrosal. However, the anterior portion, including the shelf-like portion of the tegmen tympani, is missing and the anterior lacerate foramen and middle lacerate foramen are very poorly defined.

The description of the petrosals of AMNH 3669 is supplemented by two additional specimens preserving portions of the pars mastoidea which are absent on AMNH 3669: NMMNH P-30684 and NMNH P-65619. Both are concreted braincases but display the mastoid process in reasonably good detail.

The petrosal is subdivided into two gross parts: the pars cochlearis and the pars canalicularis. The tympanic exposure of the pars cochlearis is primarily composed of the promontorium, which houses the cochlea internally. The pars canalicularis is formed by a reversed ‘L’ shaped piece of bone surrounding the lateral and posterior edges of the pars cochlearis; internally the pars canalicularis houses the vestibular system. The pars mastoidea forms part of the pars canalicularis and is composed of the region posterior to the jugular sulcus and the stylomastoid notch of the pars canalicularis, including part of the external acoustic meatus and the mastoid process. Relative to the overall size of the skull, the tympanic exposure of the pars cochlearis of *Periptychus* is relatively small. The proportion of the pars cochlearis relative to the basicranium of *Periptychus* is substantially smaller than that in *Pantolambda*. In contrast, the pars canalicularis (particularly the pars mastoidea portion) of *Periptychus* is proportionally large, not only in comparison to the pars cochlearis, but the skull as a whole, with the mastoid region forming a prominent projection on the posterolateral corner of the basicranium.

The auditory region of *Periptychus* is positioned in the posterolateral corner of the basicranium and is not as recessed relative to the surrounding basicranium as in *Arctocyon*; instead the ventral protrusion of the petrosal is more like that of *Pantolambda*, forming a small protrusion on the ventral surface of the basicranium. Anterolaterally, the petrosal is bordered by the squamosal. The squamosal borders the pars canalicularis anteriorly and exhibits a broad posterior expansion which forms the roof of the external acoustic meatus and the lateral wall of the epitympanic recess. Posteriorly, the squamosal forms a small post-tympanic process tightly appressed against the anterior surface of the mastoid process.

The anterior, medial and posteromedial border of the petrosal is delimited by a well-defined anterior foramen lacerum and middle foramen lacerum, which are contiguous and together form a deep vacuity around the anterior, medial and posteromedial edges of the promontorium. The foramen lacerum is delimited by the tympanic process of the alisphenoid anteriorly, the basisphenoid anteromedially, and the basioccipital posteromedially. The basicranial region surrounding the petrosal is poorly preserved on AMNH 3669. However, it does not appear that there was any contact between the tympanic process of the alisphenoid and the anterior most tip of the promontorium. It is therefore likely that the carotid opening and pyriform fenestra were continuous and located within a large sub-triangular shaped void which are within the anterior foramen lacerum and middle foramen lacerum. The opening for the carotid artery was most likely expressed by an open notch on the edge of the basisphenoid, rather than a discrete foramen within the bone. The tegmen tympani is not preserved on either side of AMNH 3669, so the size and shape of the pyriform fenestra can only be tentatively inferred as being reasonably developed by the placement of the surrounding elements. The morphology of the anterior foramen lacerum of *Periptychus* is like the condition of *Pantolambda*, in which the anterior margin of the pars cochlearis is demarcated by a large vacuity, with the carotid foramen forming a notch (it is also likely the tegmen tympani of *Periptychus* exhibited a similar morphology and size to *Pantolambda* based on where the breakages occur in AMNH 3669). Both *Periptychus* and *Pantolambda* exhibit a different condition to that of *Arctocyon* where the opening for the carotid forms a discrete foramen between the basisphenoid and pars cochlearis, so that the anterior foramen lacerum is greatly reduced in size.

The medial edge of the petrosal of *Periptychus* is separated from the basioccipital by a deep slit-like basicochlear fissure, which is continuous with the anterior foramen lacerum. The slit is narrow along its length but clearly separates the basioccipital from contacting the promontorium on the petrosal. Posteriorly, the basicochlear fissure is continuous with the posterior foramen lacerum, which is composed of two large, slightly recessed foramina separated by a commissure. The anterior most foramen in the posterior foramen lacerum is the slightly smaller of the two and marks the opening for the inferior petrosal sinus, which is adjacent to the opening of the external aperture of the cochlear canal. The inferior petrosal sinus would have entered the tympanic cavity, traversed the promontorium medial to the internal carotid artery (most likely close to or within the basicochlear fissure) and re-entered the cranial cavity via the carotid notch in the anterior foramen lacerum. The second larger foramen is the jugular foramen; it is positioned on the posteromedial border of the promontorium and is subequal in size to the hypoglossal foramen (located posteromedial to the jugular opening). The posterior foramen lacerum of *Periptychus* is notably different from that of *Pantolambda*, in that the ventral exposure of the two openings of the posterior foramen lacerum is much smaller, with a much wider basicapsular commissure and a narrow fissure extending posteriorly along the petrosal-occipital suture from the opening of the jugular foramen. It should be noted that the large ventral exposure of the posterior foramen lacerum in *Periptychus* may be due, in part, to damage to the basioccipital. In *Arctocyon* the foramen lacerum is much less distinct; the anterior portion is reduced to a discrete opening for the carotid, the basicochlear fissure is not open for its entire length and the posterior foramen lacerum forms an elongate slit-like fissure.

The promontorium of *Periptychus* forms a small but prominent hemi-ellipsoidal protuberance on the anteromedial corner of the petrosal. It projects ventrally from the surface of the basicranium and is more highly domed than in *Pantolambda* and *Arctocyon*. The longitudinal midline of the promontorium is set at an oblique angle to the sagittal axis of the skull so that the anterior apex of the promontorium points at a 30° angle towards to the sagittal midline. The promontorium of *Periptychus* (and also *Pantolambda*) is anteroposteriorly squat in comparison to *Arctocyon*. In ventral aspect, the shape of the promontorium is ovoid and the multi-peaked anterior apex tapers somewhat, but it does not form the elongate sub-pyriform ventral shape as seen in *Arctocyon*. The most anterior portion of the promontorium is formed by a ‘peak’ above the fenestra cochleae, which corresponds to the pole of the promontorium as defined by Luo and Gingerich [84]; O’Leary [79] synonymizes the pole of the promontorium with the anterior epitympanic wing. This area in *Periptychus* is slightly expanded (considerably more so than in *Protungulatum* or *Pantolambda*), but does not form a prominent anterior epitympanic wing. Posteriorly the promontorium is mediolaterally broad and bulbous. Overall, the promontorium of *Periptychus* is more comparable to *Pantolambda* than either to *Protungulatum* or *Arctocyon*.

The posterior portion of the promontorium is dominated by three fossulae. The posterolateral most fossula contains the fenestra vestibuli; it is a small ovoid opening in the posterolateral region of the promontorium, which in life would have been closed by the footplate of the stapes. Due to the preservation of the specimen, the exact size and position of the opening is hard to determine; it is best viewed on the right petrosal but is poorly preserved. The fenestra vestibuli of *Periptychus* appears most comparable to the condition in *Pantolambda* (and *Phenacodus*) whereby the fenestra is a shallow opening that appears to extend onto the tympanic recess. In *Pantolambda* this gives the lateral border of the pars cochlearis a sigmoidal profile, whereas in *Protungulatum* and *Arctocyon* the fenestra vestibuli is positioned on the lateral wall of the promontorium (which is much more domed relative to the tympanic recess).

Medial to the fenestra vestibuli is a second, larger fossula which contains the fenestra cochleae. The fenestra cochleae is formed by a mediolaterally broad ovoid depression within a large vacuity on the posteroventral surface of the promontorium. In life, this would have been sealed by the secondary tympanic membrane, which served to relieve rapid changes in hydraulic pressure caused by the vibrating stapes. The fenestra cochleae of *Periptychus* is proportionally much larger relative to the size of the promontorium than in *Pantolambda*, and in this regard *Periptychus* bears more resemblance to *Arctocyon* and *Protungulatum* in that the fossula is large and is posteriorly delimited by a thick border. The fenestra vestibuli and fenestra cochleae are separated by the interfenestralis region, which is broadly rounded rather than crested.

A third fossula is positioned medial to the fenestra cochleae on the ventral surface of the promontorium. We interpret this depression as the external aperture of the cochlear canaliculus (= cochlear aqueduct). It forms a large triangular depression that is subequal-to-larger than the fenestra cochleae in size. In life, the cochlear canal connected the perilymphatic space and the subarachnoid space, which served to adjust long term pressure changes in the perilymphatic space. It also transmitted the cochlear vein from the cochlea to the internal jugular. A large ventral external aperture for the cochlear canal is unusual in extant taxa. Among Paleocene mammals, a ventral aperture is much more prevalent, although notably absent on the ventral surface of *Protungulatum*. *Pantolambda*, *Arctocyon*, *Phenacodus*, *Hyopsodus*, and *Eoconodon* all exhibit a proportionally enlarged ventral aperture; however, the shape of the fossula varies. The condition in *Pantolambda* most closely resembles the triangular fossula of *Periptychus*, but does not attain as much ventral exposure. The aperture of *Arctocyon* is large but more rounded. In *Phenacodus* the opening is particularly large, forming an anteroposteriorly elongate ovoid fossula, whereas in *Hyopsodus* it is smaller, forming a mediolaterally elongate, ovoid opening.

On the left side of AMNH 3669, the surface of the promontorium of *Periptychus* is bulbous and highly sculpted. In ventral view, the pars cochlearis forms a strong medial protrusion which is separated from the main body of the promontorium by a relatively deep anteroposteriorly directed groove, which extends the length of the promontorium to the medial border of the fenestra cochleae. The medial protrusion appears to give rise to the rostral tympanic process, with a distinct ventrally directed apex that is confluent with the anterior apex of the external aperture of the cochlear canaliculus. We tentatively interpret the structure on the left side of AMNH 3669 as a rostral tympanic process rather than a medial epitympanic wing given that it projects ventrally. However, we note that on the right side of AMNH 3669 there is no indication of a rostral tympanic process. A medial tympanic wing is absent in *Periptychus*.

Several very faint sulci appear to traverse the main body of the promontorium of *Periptychus* (best observed on the right side of AMNH 3669). A small, indistinct sulcus extends mediolaterally across the promontorium just anterior to the promontorial fenestrae, which we interpret as the sulcus of the common carotid artery. The common carotid artery enters the tympanic cavity from the neck; above the fenestra cochleae the common carotid bifurcates into the internal carotid artery. In Placentalia, the path of this artery is variably demarcated by a shallow median transpromontorial sulcus [85]. In *Periptychus*, a faint sulcus is seemingly discernible along sections traversing the length of promontorium anteriorly, with the anterior end of the sulcus positioned in the ‘valley’ on the lateral edge of the pole of the promontorium. The mediolateral sulcus is also faint, but discernible along mediolateral sections above the fenestra cochleae before traversing the promontorium anteriorly almost parallel to the sulcus for the internal carotid artery. We interpret the continuation of the mediolateral sulcus as the sulcus for the stapedial arteries [86]. The proximal stem of the stapedial artery occupies the mediolateral component of the sulcus. A proximal stapedial artery is absent in extant artiodactyls [79,87,88], perissodactyls [88] and cetaceans [87], except as a possible ontogenetic feature in a few artiodactyl taxa where it is present in early growth stages (*Sus*) [79]. In extinct taxa, a sulcus for the proximal stapedial artery is seemingly common and present in *Protungulatum* [79], *Arctocyon*, *Hyopsodus* [89], *Phenacodus* [89] and *Pantolambda* [69], where it is shallow but evident in low light. In most eutherian mammals the proximal stapedial artery (when present) typically passes through the obturator foramen of the stapes before bifurcating into the superior and inferior rami [88].

There is no osteological evidence for the posterior ramus of the stapedial artery in *Periptychus*. The lack of osteological evidence is not unusual and based on observations of extant eutherian taxa it seems likely that if the artery was present it would have branched from the proximal stapedial artery and traversed the petrosal posteriorly, possibly alongside the facial nerve [88]. The proximal stapedial artery of *Periptychus* bifurcated into the superior and inferior rami ventral to the tympanic roof, as is typical of most eutherian taxa rather than within the cranial cavity [88]. This is deduced based on several osteological features on the ventral surface of the tympanic region for the rami inferior. The life position of the superior ramus of the stapedial artery in *Periptychus* is highly speculative due to the absence of the tegmen tympani; however, it is still possible to deduce some aspects of the anatomy of this artery. In eutherian taxa, the ramus superior enters the cranial cavity via one of three possible openings: either through a specific stapedial foramen in the petrosal or squamosal, through a foramen within the tegmen tympani or through the pyriform fenestra [90] anterior to the tegmen tympani [88]. An unambiguous foramen for the exit of the superior ramus of the stapedial artery has been observed in *Arctocyon primaevus*, where it is present and positioned lateral to the epitympanic recess [51] (visible on MNHN BR L9). In the mesonychians *Hapalodectes* [91], *Mesonyx* [91] and *Dissacus* [84], a foramen is present in the anterior vicinity of the epitympanic recess. There is no evidence of a foramen in either of these positions in *Periptychus* though the area appears to be reasonably well preserved. A plausible opening is not preserved, and presumed absent, in *Phenacodus* [91] and *Protungulatum* [79] (uncertain if absent in life or due to preservational damage). However, in *Pantolambda*, Muizon [69] describes an opening as present positioned lateral to tegmen tympani and medial to the epitympanic recess, but the authors do not annotate it in their associated figures. Based on our own observations of *Pantolambda* (AMNH 16663), we observe a small fissure lateral to the tegmen tympani which is anteriorly continuous with the pyriform fenestra, but postulate whether a larger opening in the pyriform fenestra (anterior to the tegmen tympani) is more likely.

In *Periptychus*, a dorsal opening in visible on the right petrosal of AMNH 3669; it is positioned on the anteromedial edge of the ventral process of the tegmen tympani. Considering the distortion of the petrosal relative to the surrounding bone, this foramen would have been positioned within the petrosal and near the tegmen tympani and the contact between the petrosal, squamosal and alisphenoid which is concordant with the position of the stapedial foramen in *Pantolambda*. It is not possible to state whether it is a specific foramen or falls within the pyriform fenestra without the tegmen tympani. Regardless of the nature of the opening it occupies a similar position to that of *Pantolambda* rather than the more anterior/lateral positions seen in *Arctocyon* and the mesonychids.

In eutherian taxa, the superior ramus of the stapedial may bifurcate to produce the inferior ramus; then, if present, the inferior ramus will invariably bifurcate into the infraorbital and mandibular rami [88]. There is no osteological evidence of superior-inferior bifurcation in *Periptychus*; however, a well-defined preotic crest indicates the presence of the ramus infraorbitalis, and thus the presence of the inferior ramus with a bifurcation from the superior ramus somewhere within the tympanic cavity can be inferred. The ramus mandibularis may extend to the mandible via the Glaserian fissure on the squamosal [82,92]; however, a Glaserian fissure path for the ramus mandibularis is somewhat contentious and not common among extant taxa [88].

The pars canalicularis surrounds the lateral and posterior edges of the pars cochlearis forming a reverse ‘L’ shaped bone which internally housed the semi-circular canals. The pars canalicularis of *Periptychus* is recessed relative to the pars cochlearis and takes up a proportionally larger area of the petrosal compared to the other comparative taxa observed in this study. The shelf of the tegmen tympani is not preserved on either petrosal of AMNH 3669. However, its presence is indicated from the broken surfaces surrounding the exposed cavum supracochleae for the geniculate ganglion. In life, the geniculate ganglion would have given rise to the greater petrosal nerve, which in turn would have exited the cavum supracochleae via the hiatus fallopii, and the posterior continuation of the facial nerve, which would have exited the cavum supracochleae via the secondary facial foramen at the posterior edge of the tegmen tympani. The location of the hiatus fallopii is unknown for *Periptychus* due to the missing tegmen tympani. The hiatus fallopii of *Protungulatum* is known to open ventrally into the tympanic cavity given that the foramen is positioned within the anterior region of the tegmen tympani [79,86]. In *Pantolambda*, it also opens ventrally into the tympanic cavity as the foramen is positioned on the anteromedial corner, within the tegmen tympani. Cifelli [89] described the hiatus fallopii of *Hyopsodus* and *Phenacodus* as positioned on the anterior edge of the tegmen tympani and opening anteriorly into the pyriform fenestra so that the greater petrosal nerve exits the cranial cavity ventrally, but external to the tympanic cavity. It therefore seems likely that the hiatus fallopii of *Periptychus* opened ventrally (as opposed to dorsally), but it is not clear whether it opened into, or external to, the tympanic cavity.

In *Periptychus*, the space for the tegmen tympani is mediolaterally broad (subequal to half the mediolateral width of the promontorium) and relatively uniform in mediolateral width. Posteriorly the tegmen tympani would have overlain the facial sulcus and formed the ventral border of the secondary facial foramen. The secondary facial foramen is not preserved on the left petrosal and barely visible on the right petrosal of AMNH 3669, but the facial sulcus is well defined and it appears that the secondary facial foramen is positioned anterior relative to the fenestra vestibuli. The sulcus is mediolaterally-broad and ventrally-open along its entire length, as opposed to being enclosed within a canal. The facial canal of *Periptychus* is proportionally broader than in *Pantolambda* and better defined than in *Protungulatum*. It extends posteriorly and curves along the lateral side of the tympanic recess, passing around the ovoid fossa for the stapedius muscle (fossa muscularis minor) and through the stylomastoid notch; the lateral border of its anterior path is delimited by the crista parotica. The stylomastoid notch of *Periptychus* is large and formed by the tympanohyal laterally and the caudal tympanic processes medially. In *Pantolambda*, the lateral caudal tympanic process delimits the medial edge of the stylomastoid notch to form a much smaller aperture than in *Periptychus* (where the lateral caudal tympanic process is positioned ventral to the stylomastoid notch and does not interrupt its margin).

In *Periptychus*, the tympanohyal is wholly-preserved on both the left and right petrosals of AMNH 3669. It forms a robust ventromedially-projecting hook-like process over the stylomastoid notch, with a slightly expanded, pointed apex. The tympanic process of *Periptychus* is well developed and subequal in size to the caudal tympanic process, but it is not as robust as the tympanohyal of *Pantolambda*, in which the apex is squared off and considerably larger than the caudal tympanic process.

In *Periptychus*, the caudal tympanic process is massive with no contribution from the exoccipital. The medial process forms a transverse strut which extends from the jugular opening at the posteromedial edge of the promontorium to a point on the posterior edge of the promontorium that is level with the lateral edge of the fenestra cochleae. There is some disparity between the morphology of the left and right caudal tympanic processes on AMNH 3669. The caudal tympanic process on right petrosal of AMNH 3669 is more complete than on the left, but is not as well preserved. It forms a robust process and shows the full mediolateral extent of the process. However, the separation between the lateral and medial processes appears indistinct due to damage. The left petrosal preserves only the lateral-most part of the process, which is formed by a transverse medial process with a mediolaterally-aligned crest along its preserved length (which constitutes the medial border of the stylomastoid notch) and a smaller lateral process. The two processes are separated by a small groove, which we tentatively interpret as the sulcus for the posterior auricular nerve (CNX) [69]. The medial process is separated from the fenestra cochleae anteriorly by the postpromontorial sinus [69,91], which is expressed as a shallow depression on the shelf just posterior to the fenestra cochleae.

In *Periptychus*, and also *Pantolambda*, the postpromontorial sinus is confined to a narrow shelf, whereas in *Arctocyon* it forms a much larger shelf-like channel directed towards the stylomastoid notch. The caudal tympanic process of *Arctocyon* is not as robust as in *Periptychus*. In *Arctocyon*, the medial process forms a small protuberance instead of a transverse strut like that seen in *Periptychus* and *Pantolambda*. Additionally, in *Arctocyon*, the region posterior to the promontorium (occupied by the caudal tympanic process and postpromontorial sinus) is anteroposteriorly elongate, whereas in *Periptychus* (and *Pantolambda*) it is much more compressed, giving the petrosal a squatter appearance. The caudal tympanic processes on the left petrosal of *Periptychus* (AMNH 3669) are comparable to *Pantolambda* in that the transverse medial process is crested. However, in *Periptychus* the strut is positioned at a more oblique angle and is much less robust. The lateral process of the left petrosal of *Periptychus* (AMNH 3669) is proportionally larger than in *Pantolambda* and positioned ventrally over the stylomastoid notch (rather than interrupting the margin of the stylomastoid notch as in *Pantolambda*).

The anterolateral portion of the pars canalicularis of *Periptychus* is formed by the epitympanic recess, which is separated from the medial portion of the pars canalicularis by the crista parotica. The crista parotica extends posteriorly along the medial edge of the epitympanic recess to the tympanohyal and continues onto the pars mastoidea. Anteriorly, the crista parotica forms a prominent anteroventromedially-directed flange. The projection is not as recessed as the roof of the tympanic cavity of the medial portion of the pars canaliculus. In *Periptychus*, it forms an enlarged ventrally projecting flange with a flat and slightly depressed ventral surface that was most likely level with the tegmen tympani. The large triangular depression seats the anterior crus of the ectotympanic. In *Pantolambda*, the depression is subequal in size to that of *Periptychus* but lenticular in shape, whereas in *Arctocyon* it forms little more than a ridge. Interestingly, in *Phenacodus* it is massively expanded relative to the rest of petrosal [89]. A ventrally-projecting flange is absent in *Protungulatum* [79]. Posteriorly, the crista parotica becomes nearly flush with the tympanic surface at approximately the same mediolateral level as the secondary facial foramen. Here it forms a well-defined ridge that separates the facial sulcus on its medial side from the more deeply excavated epitympanic recess on its lateral side. Where the crista parotica meets the tympanohyal, it forms an articular facet for the posterior crus of the ectotympanic. The facet forms the posteromedial edge of the fossa incudis and extends up the anterior edge of the tympanohyal. Posterior to the tympanohyal, the crista parotica is less defined but can be seen extending laterally to the squamosal-mastoid suture and the mastoid process.

Positioned lateral to the crista parotica is the epitympanic recess, which housed the incudomalleolar articulation. In *Periptychus* the epitympanic recess forms a large, elongate depression that is larger in size than in *Protungulatum*, *Arctocyon*, or *Pantolambda* (even when considering the displacement of the petrosal), but it is not as proportionally deep. Based on AMNH 3669 it appears that the entire epitympanic recess is situated within the petrosal, with the petrosal-squamosal suture demarcating the lateral border between the epitympanic recess and the external acoustic meatus. Anteriorly the epitympanic recess is nearly continuous with the Glaserian fissure on the squamosal. On the left petrosal of AMNH 3669 the anterior border of the recess is clearly defined by a fracture along the petrosal-squamosal suture. The long axis of the recess is orientated anteroposteriorly and the recess deepens posteriorly. The anterior two-thirds of the recess are formed by the malleolar fossa, which is a shallow depression that tapers anteriorly to a medially directed apex. In life, this fossa would have housed the malleus. The posterior third is formed by a deeper ovoid fossa incudis, which in life would have housed the short crus of the incus. The fossa incudis lies posterior to the fenestra vestibuli.

The external acoustic meatus is primarily composed by the squamosal, with a small contribution from the petrosal at the posterior wall. The arcuate channel is created by an anteroposteriorly wide and smooth arch which broadens externally. The anterior border is formed by the near vertical wall of the squamous root of the zygomatic arch and the postglenoid process, and the posterior border is formed by the posttympanic process, which is appressed against the mastoid process of the petrosal to form a single protuberance. The auditory bulla and ectotympanic are not preserved. The external acoustic meatus of *Periptychus* is like that of *Pantolambda*, in that the suprameatal fossa forms a broad roof both anteroposteriorly and mediolaterally, so that the inner ear is placed well within the basicranium. This is very different from the condition in *Arctocyon*, in which the external acoustic meatus is very short (both mediolaterally and anteroposteriorly) and the auditory region is positioned near the external edge of the basicranium.

The pars mastoidea of *Periptychus* is massive, forming a prominent angle on the posterolateral corner of the braincase. It makes a large contribution to the lateral surface of the braincase between the squamosal and occipital complex. The expanded mastoid condition of *Periptychus* is broadly like the morphology in *Pantolambda*, in which the pars mastoidea forms a broad corner on the posterior edge of the braincase. *Periptychus* and *Pantolambda* both exhibit a different morphology to *Arctocyon*, in which the pars mastoidea is much reduced and the posterior portion of the braincase tapers mediolaterally relative to the anterior portion.

The mastoid process of *Periptychus* is positioned adjacent to the jugular sulcus and posterolateral to the hypoglossal foramen. The post-tympanic process is tightly appressed against the mastoid process anteriorly and the paraoccipital process is tightly appressed against the mastoid posteriorly, resulting in a particularly robust ventral protuberance at the site of the mastoid process. For the purpose of this description, the mastoid process refers to the central ridge on the protuberance and the term ‘mastoid protuberance’ refers to the entire protrusion formed by the post-tympanic, mastoid and paraoccipital processes. The lateral placement of the mastoid protuberance relative to the auditory apparatus in *Periptychus* differs from the condition in *Pantolambda*, in which the mastoid process is positioned posterolateral to the auditory region and inset from the edge of the basicranium, with the post-tympanic and paraoccipital processes more widely spaced and less well-defined.

The ventral surface of the mastoid protuberance features three distinct ridges; from each of these ridges a crest extends dorsally and converges with the nuchal crest dorsally on the posterior border of the cranium. The lateral exposure of the mastoid forms a broad surface that tapers dorsally and faces posterolaterally. A distinct mastoid foramen is present on the anterolateral side of the mastoid protuberance adjacent to the anterior-most crest formed by the squamosal-petrosal suture. *Pantolambda* exhibits a similar morphology of the pars mastoidea to *Periptychus*; however, in *Pantolambda* the mastoid process ridges are not appressed and their associated crests are much less distinct, forming a more posteriorly-directed surface that only extends a third of the way up the dorsoventral height of the skull.

Numerous muscles attach to the lateral surface of the pars mastoidea. The anterior surface of the mastoid protuberance (post-tympanic process and mastoid process) and its associated lateral ridges provide attachment for muscles with the principal purpose of stabilising and moving the head and neck. The posterior surface of mastoid protuberance (paraoccipital process and posterior surface of the mastoid process) also provides attachment for muscles that stabilise the head and neck, as well as for muscles associated with the mandible.

The post-tympanic process is a small rounded protuberance on the anterior surface of the mastoid process. It does not make any contribution to the lateral surface of the mastoid process. Ventrally it forms the posterior wall of the external acoustic meatus and is subequal to the postglenoid process in ventral projection. Dorsally, it gives rise to a sharp crest formed by the suture between the squamosal and pars mastoidea of the petrosal, which is near continuous with the nuchal crest dorsally.

The mastoid process forms a sharp ridge on the ventral surface of the protuberance, which marks the most ventral point of the process with a thick crest that ascends to the nuchal crest. This crest would have provided attachment for the sternocleidomastoid muscle ventrally, and for the splenius and longissimus capitis muscles that attached midway along its length. The splenius would have attached anterior to the longissimus capitis, and both would have attached posterior to the sternocleidomastoid and extended onto the superior nuchal line of the occipital [66].

The paraoccipital process of the exoccipital forms a small, erect, trihedral-shaped eminence pushed up against the posterior surface of the mastoid process. A well-expressed ridge, formed by the suture between the lateral exposure of the pars mastoidea and the occipital complex, extends ventrally to the nuchal crest. The digastric muscle attached to the ventral apex of the paraoccipital [93], with the rectus capitis lateralis attaching to the ventral portion of the ridge and the obliquus capitis anterior attaching to the more dorsal portion of the ridge as well as the mastoid ridge [86].

#### Occipital complex

The occipital complex is formed of four components: the basioccipital, the paired exoccipitals and the supraoccipital. In mature animals, these bones fuse and in the specimens observed it is difficult to structurally delimit the borders of the individual components. Therefore, in the following description we will treat the occipital complex as a single element and note individual component boundaries where possible. The following description is based on NMMNH P-65619, a small fragment of the posterior braincase preserving the exoccipital and supraoccipital (the basioccipital is mostly damaged) (Fig 5); NMMNH P-30684, a portion of the right braincase preserving the left exoccipital and part of the supraoccipital (Fig 6); NMMNH P-35194, a concreted braincase preserving most of the occipital complex; NMMNH P-36631, a skull fragment preserving the occipital condyles (Fig 4); and AMNH 3669, a posterior braincase preserving fragments of the occipital complex which have been set in plaster (Fig 8).

The unpaired basioccipital of *Periptychus* forms the basilar portion of the occipital complex. Anteriorly, it exhibits a mediolaterally-broad contact with the basisphenoid as previously described. Posteriorly, it remains broad to form a sub-quadrate plate, as opposed to a posteriorly-broadening trapezoidal bone like that in *Pantolambda*. Anteriorly and laterally, the border of the basioccipital of *Periptychus* is delimited by the foramen lacerum, which inhibits any contact with the petrosal promontorium. The lack of contact between the basioccipital and promontorium observed in *Periptychus* is generally like the condition observed in *Pantolambda*, in which a continuous foramen lacerum prevents contact between the basioccipital and petrosal. However, in *Pantolambda* the ventral exposure of the posterior foramen lacerum appears to be much smaller, with a narrow fissure extending posteriorly along the petrosal-exoccipital suture from the opening of the jugular foramen. It should be noted that the large ventral exposure of the posterior foramen lacerum in *Periptychus* may be enhanced by preservational distortion. In *Arctocyon*, the basioccipital is positioned much closer to the petrosal, with a comparatively reduced foramen lacerum. In *Periptychus*, the ventral surface of the basioccipital features two ovoid fossae located bilaterally to a weak medial crest; the two fossae are relatively well-excavated and provide large attachment areas for the insertion of the ventral rectus capitis muscles. Similar fossae are observed in *Carsioptychus* and *Pantolambda*, but are seemingly absent in *Arctocyon*.

The paired exoccipital bones of *Periptychus* are poorly differentiated from the basioccipital and supraoccipital, and known only from fragments. The anterolateral border of the exoccipital forms the posteromedial border of the posterior foramen lacerum (jugular sulcus + inferior petrosal sinus = jugular foramen = posterior foramen lacerum). The hypoglossal foramen is positioned posterior to the posterior foramen lacerum. The hypoglossal foramen itself sits within a large, circular, ventrally directed opening and appears to have two separate divisions within the foramen, based on AMNH 3669. The hypoglossal foramen of *Periptychus*, and also *Arctocyon*, is relatively large in comparison to that of *Pantolambda*, in which the foramen forms a comparatively small, single opening. In *Periptychus*, the hypoglossal foramen is positioned approximately midway between the posterior foramen lacerum and occipital condyle, whereas in *Pantolambda* it is within close proximity to the jugular sulcus, and in *Arctocyon* it is positioned well posterior to the posterior foramen lacerum.

In *Periptychus*, the paraoccipital process of the exoccipital forms a small, ventral eminence tightly appressed against the posterior surface of the mastoid process. A well-expressed ridge is formed at the contact between the paraoccipital complex and mastoid process, which extends ventrally to the nuchal crest. The digastric muscle originates at the ventral apex of the paraoccipital process [93], with the rectus capitis lateralis attaching to the ventral portion of the ridge, and the obliquus capitis anterior attaching to the more dorsal portion of the ridge as well as the mastoid ridge [86]. *Periptychus* exhibits a different, more appressed morphology of the pars mastoidea compared to that observed in *Pantolambda*. In *Pantolambda*, the mastoid process ridges are not as tightly appressed and their associated crests are much less distinct, forming a more posteriorly-directed surface that only extends a third of the way up the dorsoventral height of the skull.

In *Periptychus*, the occipital condyles demarcate the posteroventral-most exposure of the exoccipital and form the ventral edge of the foramen magnum. The posteroventral position of the occipital condyles in *Periptychus* differs from the more posterior position observed in *Pantolambda*, where the condyles form prominent posterior projections relative to the braincase. In ventral aspect, the occipital condyles of *Periptychus* form two discrete, sub-conical protuberances on either side of the cranial midline, which are separated by a relatively deep odontoid notch. The separated condition exhibited by *Periptychus* (also observed in *Carsioptychus*) differs from *Pantolambda*, in which the occipital condyles form a continuous articular surface around the ventral border of the foramen magnum. In *Periptychus*, the transverse axis of each condyle lies oblique to the mediolateral axis of the skull, so that the condyles are positioned posterolateral-anteromedially.

The occipital condyles articulate with the atlas via the atlantal articular facet. In *Periptychus*, the atlantal articular facet is subdivided into ventral and dorsal portions. The dorsal portion is shallowly convex dorsoventrally, but not mediolaterally, with the articular surface facing posterodorsally. The dorsal facet in *Periptychus* is proportionally smaller and is not as transversely convex as the dorsal facet in *Pantolambda*. The ventral portion of the atlantal articular facet of *Periptychus* is strongly convex anteroposteriorly and faces ventrolaterally. In ventral aspect, the ventral facet is subconical in shape and increases in diameter distally. The ventral atlantal articular facet of *Periptychus* is proportionally larger along its transverse axis and more conical than the condition in *Pantolambda*. In *Periptychus*, the dorsal and ventral facets of the atlantal articular facet are variably distinguished from one another: in NMMNH P-35194 the facets are delimited from one another by a distinct ridge, whereas in NMMNH P-30684 the two facets are poorly delimited and near continuous.

The medial and lateral edges of the condyle are marked by sharp, sub-vertical walls with no mediolateral convexity. The medial wall of the occipital condyle of *Periptychus* exhibits a small, but deep, circular fossa which we interpret as the insertion site of the alar ligament. Laterally, the occipital condyle is separated from the paraoccipital process by a deep ventral condyloid fossa. Dorsally, the ventral condyloid fossa is continuous with the dorsal condyloid fossa, which is formed by a shallow depression within the exoccipital just dorsal to the occipital condyle. The foramen magnum is positioned in the ventral half of the posterior wall of the braincase. The exoccipital contains most of the foramen magnum, with a small contribution from the supraoccipital ventrally. The foramen is bilaterally symmetrical and opens posteriorly.

The supraoccipital is large and forms the dorsal portion of the posterior wall of the braincase, the nuchal crest and a small portion of the posterolateral walls of the braincase. The posterior portion of the supraoccipital is dorsoventrally-tall and lacks a vertical median crest; however, a deep concave depression is present dorsal to the foramen magnum for the insertion of nuchal muscles. Individual, well-defined muscle attachment sites for the various nuchal muscles are not present in *Periptychus*. The nuchal crest is large and composed entirely of the supraoccipital. It projects posterodorsally relative to the posterior wall of the braincase. In ventral aspect, the nuchal crest is deeply notched at its midpoint, where it meets the sagittal crest. Lateral to its midpoint the crest expands posterodorsally. At its most expanded point, the nuchal crest projects just posterior to the foramen magnum and lateral to the lateral edge of the occipital condyle. *Periptychus* exhibits a similar nuchal crest morphology to *Carsioptychus*, *Ectoconus*, and *Arctocyon* in that the crest forms bilobate posterodorsal projections which meet at a notched midpoint. *Pantolambda* exhibits a starkly different condition to *Periptychus* and the other ‘condylarth’ taxa. The posterior supraoccipital and nuchal crest form a near vertical wall on the posterior end of the braincase, with no posterodorsal projection of the nuchal crest. Further to this, the nuchal crest of *Pantolambda* is mediolaterally broad, much more so than in *Periptychus*, and extends well beyond the lateral edge of the occipital condyles.

In *Periptychus*, the supraoccipital extends onto the dorsolateral sides of the posterior braincase. Anteriorly, the supraoccipital overlays the posteroventrolateral region of the parietal along the lambdoid suture and anterior to the nuchal crest. The supraoccipital does not contact the squamosal on the lateral sides of the braincase due to the dorsoventral expansion of the parietal. This condition is also evident in *Carsioptychus*, *Ectoconus*, and *Arctocyon*. However, it appears that in *Carsioptychus*, *Ectoconus*, and *Arctocyon* the squamosal maintains a small contact with the supraoccipital on the dorsal surface of the braincase due to the limited expansion of the pars mastoidea on the posterolateral corner of the braincase. The condition of *Pantolambda* is broadly similar in that the supraoccipital contacts the parietals along the lambdoid suture anterior to the nuchal crest; however, the supraoccipital-parietal contact is limited by the dorsal expansion of the squamosal, resulting in a dorsoventrally extended contact between the supraoccipital and squamosal.

#### Mandible

**Fig 9:**
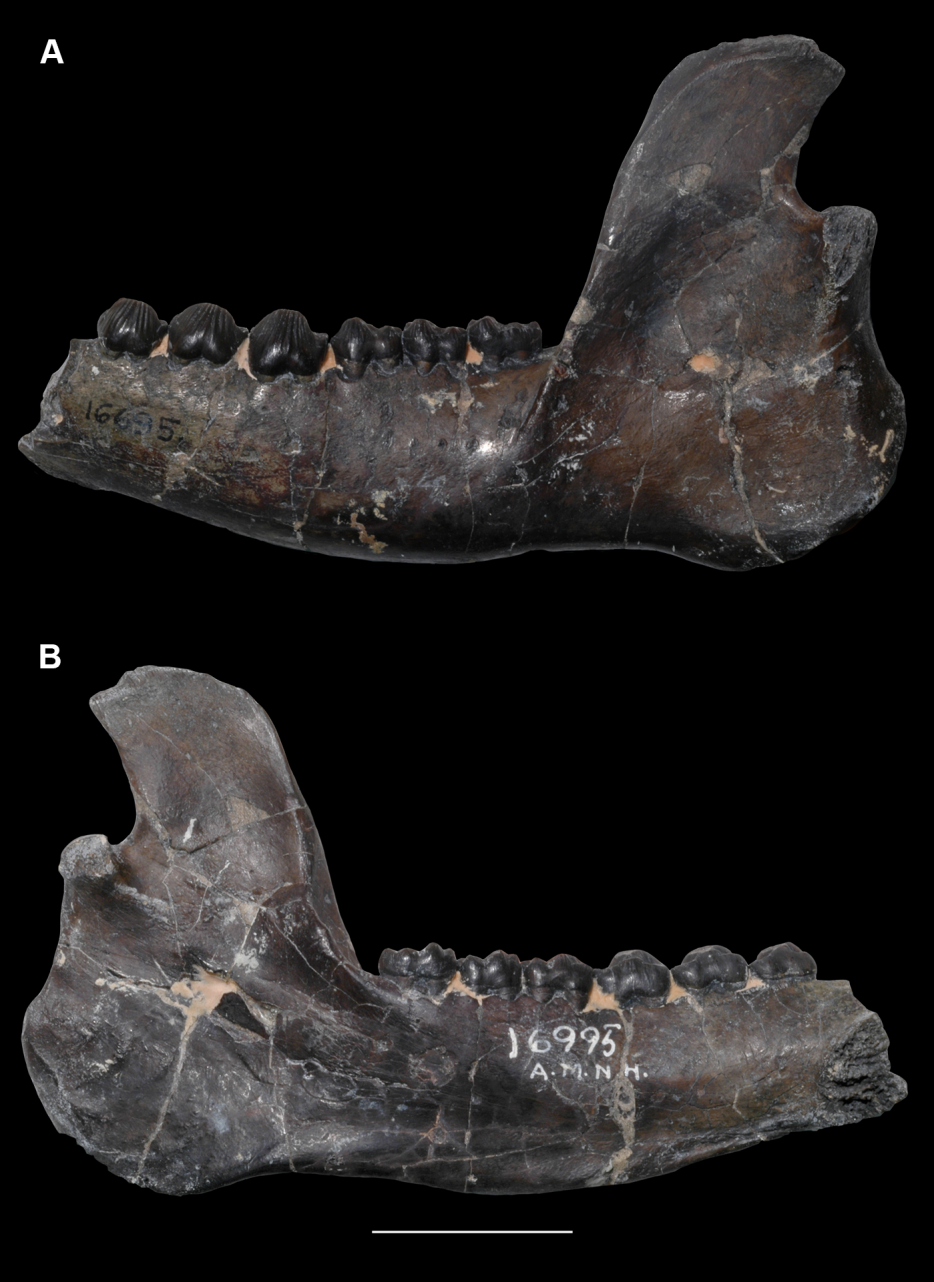
Left dentary of *Periptychus carinidens* (AMNH 16995). (A) lateral view; (B) medial view. Scale bar: 30mm.

**Fig 10:**
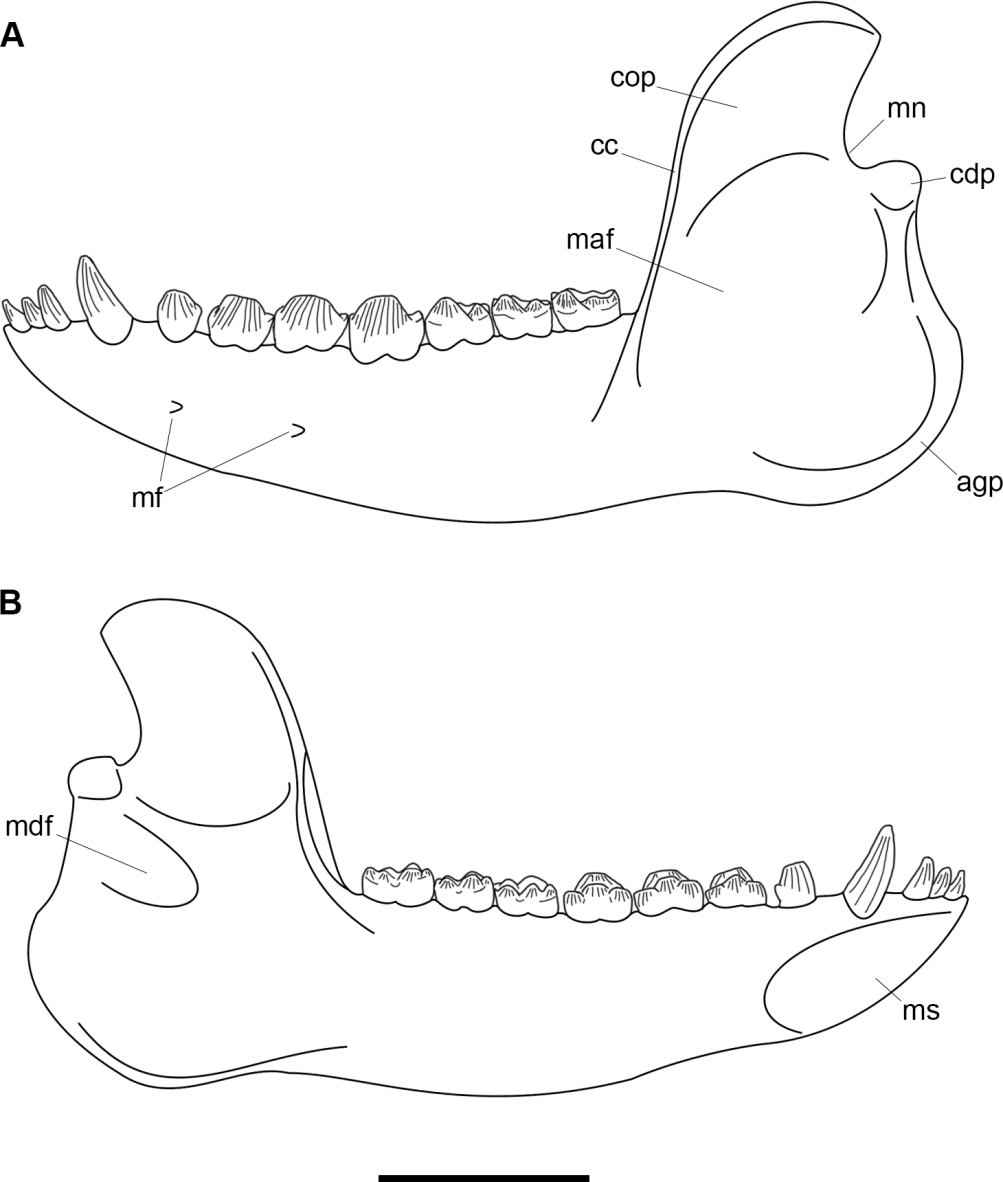
Annotated line drawing of the left dentary of *Periptychus carinidens*. (A) lateral view; (B) medial view. Abbreviations: agp, angular process; cc, coronoid crest; cdp, condyloid process; cop, coronoid process; maf, masseteric fossa; mdf, mandibular foramen; mf, mandibular foramina; mn, mandibular notch; ms, mandibular symphysis. Scale bar: 30mm.

The mandible is reasonably well known for *Periptychus*, with several near complete dentary specimens. AMNH 3665 is a sub-complete specimen preserving a large portion of the right mandibular body, the ventral portion of the ramus, and the symphysis with the anterior portion of the left dentary. AMNH 3690 is a slightly more complete specimen preserving the mandibular body and ramus of the left dentary. The coronoid process is reasonably well intact, but the condyloid process is damaged. AMNH 16695 is a near complete and well-preserved left dentary (Figs 9 and 10); however, the condyloid process is damaged and the anterior end of the mandibular body is missing. Comparisons are made to *Ectoconus ditrigonus* (AMNH 16495, figured in [5]), *Protungulatum donnae* (SPSM 62–2028, figured in [94]), *Arctocyon primaevus* (MNHN.F.CR2, figured in [52]), *Claenodon ferox* (AMNH 2459 figured in [5] and AMNH 16541), and *Pantolambda bathmodon* (AMNH 16663, figured in [5]).

The mandible of *Periptychus* is characteristically stout. The robusticity of the mandible is quantified by calculating the maximum dorsoventral height of the dentary divided by the maximum anteroposterior length. *Periptychus* (AMNH 3690) has a value of 0.57, meaning it is more robust than *Arctocyon* (0.44) but less than *Pantolambda* (0.67).

The paired dentaries of *Periptychus* are co-ossified to form a deep mandibular symphysis, which extends posteroventrally from the incisors to the ventral edge of the mandible at the level of the anterior margin of p2. The anteroventral corner of the symphysis forms an angle of ~135°. The mandibular symphysis of *Periptychus* is broadly similar to the condition in *Ectoconus*, but not as elongate as the condition in *Arctocyon* or *Claenodon*. In *Arctocyon* and *Claenodon*, the expansion of the canines permits a longer symphysis; however, the posteroventral border only extends to the level of the anterior margin of p2 as in *Periptychus*. The angle of the anteroventral corner of the symphysis in *Arctocyon* is larger than in *Periptychus* (~160°). The mandibular symphysis of *Periptychus* is not as deep as in *Pantolambda* due to the increased depth of the mandibular body in this latter. On the medial surface of the mandibular body of *Periptychus*, the mandibular symphysis terminates in a small, anteroposteriorly-orientated ventral mental spine for the attachment of the geniohyoideus muscle. The ventral mental spine is separated from the dorsal mental spine by a relatively deep fossa. The dorsal mental spine is not as well-defined as the ventral spine, and provided the origin of the genioglossus muscle.

In lateral aspect, the alveolar plane of the *Periptychus* dentary is horizontal and the ventral border is convex. The mandible achieves maximum depth between p5 and m1 and tapers anteriorly. The mandibular body of *Periptychus* is moderately deep. The maximum depth of the mandibular body divided by the anteroposterior length of the body is equal to 0.33. As such, the mandibular body of *Periptychus* is proportionally more robust than that of *Arctocyon* (0.29) and *Pantolambda* (0.31). Two mental foramina pierce the lateral surface of the mandibular body of *Periptychus* and served to transmit blood vessels and the terminal branches of the inferior alveolar nerve. The foramina are situated towards the ventral border of the mandibular body. The anterior-most foramen is slightly larger and positioned on the longitudinal axis of the posterior edge of p2; the posterior-most foramen is positioned on the longitudinal axis of the midline of p4. Both foramina open into anteriorly-directed sulci. Two foramina are also present in *Protungulatum*, positioned beneath the anterior margin of p2 and anterior margin of p5. At least three foramina are observed in *Arctocyon*: one beneath p1 and two smaller openings beneath p5 (a possible fourth foramen is observed on MNHN.F.CR2 beneath p4).

In dorsal aspect, the dentary of *Periptychus* is mediolaterally thick and tapers anteriorly. The alveolar process is broad, forming a wide shelf where it borders the lateral side of the molars for the origin of the buccinator muscle. In addition to this, the posterior tooth row (molar region) appears to shift medially relative to the anterior dentition. A similar medial shift of the molar teeth and lateral expansion of the alveolar margin is also observed in *Protungulatum* and *Arctocyon*. It is less marked than in *Ectoconus* and *Claenodon*. The lateral expansion of the posterior portion of the alveolar process may well represent an ontogenetically-variable morphology, as on AMNH 3720, a juvenile specimen of *Periptychus* preserving the deciduous dentition, the lateral margin of the alveolar process is barely expanded. In *Periptychus*, a retromolar gap is evident between the m3 and the ascending mandibular ramus. It is approximately one molar width deep, although this appears to vary across specimens and may also be an ontogenetic feature that becomes larger with age.

In medial aspect, the surface of the mandibular body of *Periptychus* is contoured with a shallow fossa extending the entire length of the body, which housed the submaxillary gland. The anteroventral border of the fossa is demarcated by the mylohyoid line, which forms a broad ridge anteriorly and extends from the dorsoventral midpoint of the mandibular symphysis posteriorly to the level of m2. Posterior to m2 the mylohyoid line is continuous with the mylohyoid groove, which extends to the mandibular foramen. The ventral border of the mandibular body is smooth and mediolaterally rounded, providing attachment for the digastric muscle. Meckel’s sulcus is absent in *Periptychus*.

The ascending mandibular ramus of *Periptychus* is relatively tall in comparison to the mandibular body. The maximum dorsoventral height of the mandibular body divided by the maximum dorsoventral height of the mandibular ramus equals 0.37. As such, the mandibular ramus of *Periptychus* is shorter (relative to the body) than the ramus of *Arctocyon* (0.42), but taller than the ramus of *Pantolambda*.

The mandibular ramus of *Periptychus* ascends posterodorsally from the mandibular body to form an enlarged and rounded coronoid process that projects ventral to the mandibular condyle. In lateral aspect, the mandibular ramus of *Periptychus* forms a ~110° angle with the mandibular body. In *Arctocyon* this angle is slightly wider (~120°), whereas in *Pantolambda* the mandibular ramus forms an acute angle with the mandibular body. The anterior border of the mandibular ramus of *Periptychus* is formed by a massive and mediolaterally-broad coronoid crest. The crest extends onto the mandibular body, terminating ventral to the anterior border of the ultimate molar (m3) in a salient and rounded tuberosity. In anterior aspect, the medial border of the coronoid crest is strongly sigmoidal and greatly thickened just above the ultimate molar. The coronoid crest of *Periptychus* is broadly like the morphology exhibited in *Arctocyon*, with both taxa possessing salient crests that terminate below the third molar. The morphology exhibited by *Periptychus* and *Arctocyon* is notably different from that of *Pantolambda*, where the crest descends vertically to terminate posterior to the level of the third molar.

The coronoid process of *Periptychus* is tall and anteroposteriorly broad along its entire height. Dorsally it tapers to a posteriorly-directed, almost hook-like, apex. The anterior border of the process is demarcated by the coronoid crest and in lateral aspect the anterior border is shallowly convex. The lateral and medial surfaces of the process are shallowly-excavated and provide large attachment areas for the insertion of the temporalis muscle. The posterior border of the coronoid process is subtly concave and dorsoventrally short, forming a small mandibular notch where it meets the mandibular condyle. The coronoid process of *Periptychus* is more like the condition exhibited by *Pantolambda* and notably different from the condition in *Arctocyon*. In *Periptychus* and *Pantolambda*, the process is anteroposteriorly deep along its entire height, whereas in *Arctocyon* it is anteroposteriorly deeper ventrally. However, the anterior border of the process of *Periptychus* is not strongly convex and more closely resembles the condition in *Arctocyon*. In this respect, *Periptychus* differs from *Pantolambda* in that the anterior border of the coronoid process of *Pantolambda* is moderately convex dorsally and distinctly concave ventrally. In *Periptychus*, the lateral and medial surfaces of the coronoid process are not as deeply excavated as in *Arctocyon*. Further to this, in *Arctocyon*, the coronoid crest forms a more salient rim around the anterior border of the process. The mandibular notch of *Periptychus* is more concave than in *Arctocyon* and *Pantolambda* and excavates the posterior border of the coronoid process.

The mandibular condyle of *Periptychus* is positioned high on the mandibular ramus, approximately two and half times as high as the length of the ultimate lower molar above the occlusal plane and approximately two thirds of the way up the ramus. The mandibular condyle of *Periptychus* is positioned higher on the ramus than the condyle in *Arctocyon*, which is barely one molar length above the occlusal plane, and in *Protungulatum*, where the condyle is approximately one and a half molar lengths above the occlusal plane. However, the condyle of *Periptychus* is not as highly positioned as that of *Pantolambda*, which is approximately three and a half ultimate molar lengths above the occlusal place. The mandibular condyle of *Periptychus* is ovoid and symmetrical, with tapered medial and lateral ends. The condyle morphology of *Periptychus* differs from *Arctocyon*, in which the condyle is proportionally more massive and strongly asymmetrical (the lateral portion is expanded relative to the medial portion). In *Periptychus*, the peduncle is short, robust and projects posteriorly to barely form a neck between the mandibular ramus and condyle. Both *Arctocyon* and *Protungulatum* exhibit a more developed and elongate condylar neck; however, in *Protungulatum* the neck and condyle project posteroventrally rather than posteriorly as in *Periptychus* and *Arctocyon*.

The angular process of *Periptychus* is greatly expanded to form a rounded, posteriorly-directed process rather than a pointed projection. The lateral surface of the angular process provided a large attachment area for a large portion of the masseter muscle. The medial surface of the angular process exhibits a broad posteroventral-anterodorsally-directed crest for the insertion of the medial pterygoid muscle. The angular process of *Periptychus* is dorsoventrally-deep and provides massive attachment areas, but it is not posteriorly-expanded and barely extends beyond the level of the posterior border of the mandibular condyle. The angular process of *Periptychus* is distinctly different form the hook-like projection in *Protungulatum* and *Arctocyon*. Other larger bodied Palaeocene ungulates such as *Phenacodus* [95], *Meniscotherium* [72], *Tetraclaenodon* [68] and *Pantolambda* [96] have a similarly expanded and rounded angular processes to *Periptychus*; however, in these taxa the process is even more posteriorly- and ventrally-expanded than the condition exhibited by *Periptychus*.

In *Periptychus*, the lateral surface of the mandibular ramus is excavated by a deep, subtriangular fossa for the insertion of the masseter muscle. The masseter fossa is subtriangular in shape and deep, but relatively small in terms of surface area due to the massive expansion of the angular process (the lateral surface of which also provides attachment to the masseter muscle). The ventral border of the masseter fossa is positioned above the alveolar plane along most of its length and is delimited from the angular process by a rounded crest extending anteroventrally from the mandibular condyle to a point on the coronoid crest just below the alveolar line. There is no anteroventral expansion of the masseter fossa onto the mandibular body. The masseter fossa of *Periptychus* is smaller and not as deeply excavated as the fossa in *Arctocyon*. Both *Periptychus* and *Arctocyon* possess a much smaller masseter fossa than *Pantolambda*. In *Pantolambda* the fossa is massive and dorsoventrally expanded, so that it nearly reaches the ventral border of the mandibular ramus.

A small, posteriorly directed mandibular foramen is present in *Periptychus*. The opening is positioned towards the centre of the medial surface of the mandibular ramus, posterodorsal to the mylohyoid groove, and on the same horizontal axis as the occlusal plane. The position of the mandibular foramen of *Periptychus* differs from *Arctocyon*, in which the foramen is positioned below the horizontal axis of the occlusal plane.

### Dentition

The dentition of *Periptychus* is distinctive. The premolars are enlarged relative to the molars and have massively expanded central cusps. The molars are highly bunodont and possess apically compressed bulbous cusps. The enamel is strongly crenulated with apicobasally-aligned ridges that are characteristic of the Periptychidae and highly developed within *Periptychus*. This immediately caught the attention of Cope, who wrote that: ‘This sculpture [in reference to *Periptychus*] is unparalleled in the class Mammalia…’ ([4], p797).

**Fig 11:**
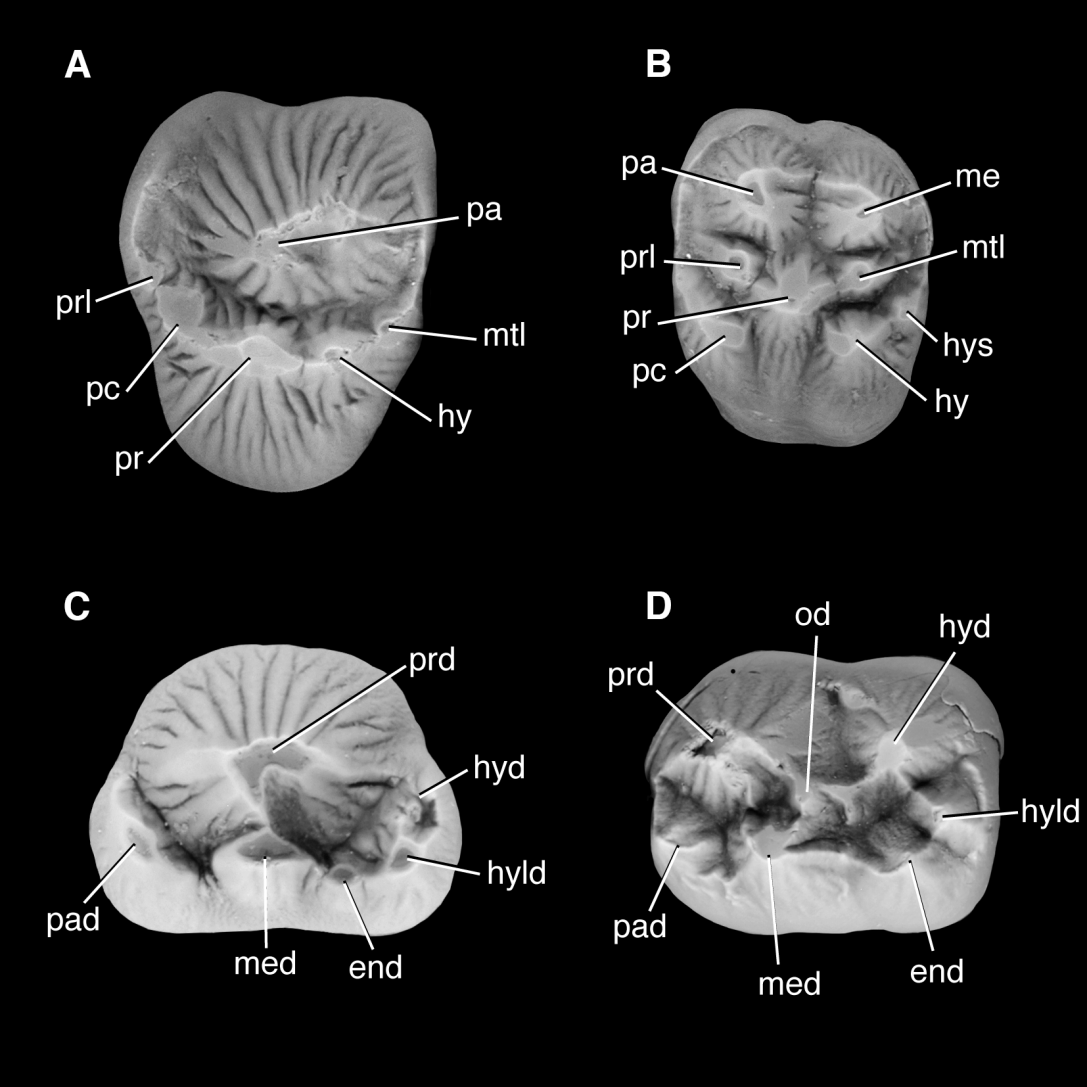
Tooth nomenclature for *Periptychus carinidens*. (A) upper premolar–P4 of NMMNH P-36631; (B) upper molar–M2 of NMMNH P-36631, (C) lower premolar—p4 of NMMNH P-36631; (D) lower molar—P4 of NMMNH P-19363. Abbreviations: end, entoconid; hy, hypocone; hyld, hypoconulid; hys, hypostyle; me, metacone; med, metaconid; mtl, metaconule; od, obliconid; pa, paracone; pad, paraconid; pc, pericone/protostyle; pr, protocone; prd, protoconid; prl, paraconule. Tooth images serve to identify cusps and are not to exact scale.

*Periptychus* possessed 44 permanent teeth with a dental formula of 3.1.4.3/3.1.4.3. The dental notation for *Periptychus* is: I1/i1, I2/i2, I3/i3, C1/c1, P1/p1, P2/p2, P4/p4, P5/p5, M1/m1, M2/m2, M3/m3. Cusp nomenclature follows the standard terminology where possible or is otherwise described (Fig 11).

*Periptychus* is well sampled throughout the early Paleocene deposits of the San Juan Basin, New Mexico, with dental specimens more common than any other part of the anatomy. Examination of a wealth of new specimens from the San Juan Basin shows a remarkable amount of size variation within *Periptychus*, with less variation in cusp arrangement. Here we describe the dental morphology of *Periptychus carinidens* from a selection of associated dental specimens covering as much of the tooth row as possible. NMMNH P-35194 is a partial skeleton with fragmentary skull and associated dentition that includes left P1-P5, M2-3, right I3-M3, left p4-5, m2-3 and right c1, p2-m3. NMMNH P-36631 is a partial skull with associated dentition that includes the left P2-M3, right P1-M3, left i1-p1 and right i1 and p1. NMMNH P-19482 is partial skull that includes the left C1-M3 and right P2-M3.

#### Incisors

**Fig 12:**
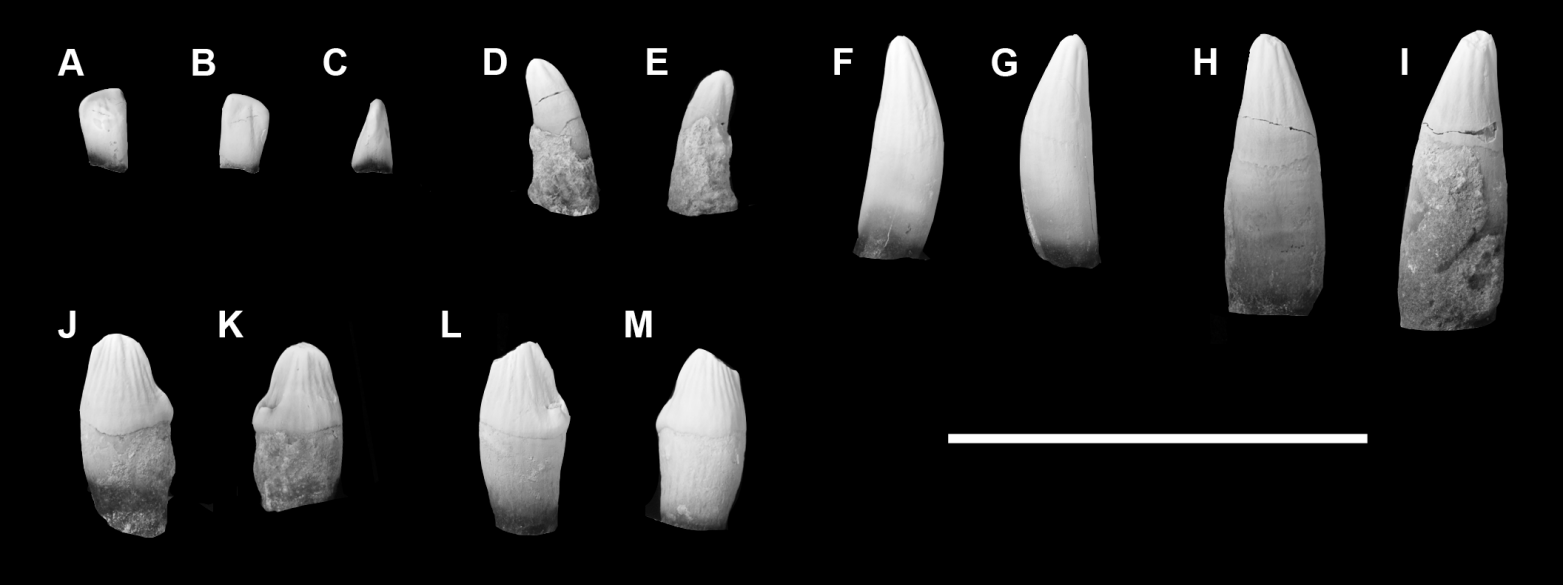
Anterior dentition of *Periptychus carinidens* (NMMNH P-36631) with right i1-3, c, p1 and left p1. Ri1 (A-C) (A) mesial/buccal view; (B) distal/lingual view; (C) lateral view. Ri2 (D-E): (D) buccal view; (E) lingual view. Ri3 (F-G): (F) buccal view; (G) lingual view. Rc (H-I): (H) buccal view; (I) lingual view. Lp1 (J-K): (J) buccal view; (K) lingual view. Rp1 (L-M): (L) buccal view; (M) lingual view. Scale bar: 30mm.

The anterior dentition of *Periptychus* is poorly known, with only a handful of specimens preserving the incisors and canines (Fig 12). The incisors are relatively simple in their form, although not completely ‘characterless’ as described by Matthew ([5], p114). The incisor morphology is not homogeneous along the tooth row, as the posterior-most incisors more closely resemble the canines than the anterior-most incisors.

The first lower incisor is a small, blunt, single-rooted, monocuspid tooth. The tooth is mesiodistally-compressed so that it is spatulate in shape, and in occlusal view the apex of the tooth is lenticular with a smooth (non-cuspidate) surface. The labial surface is relatively flat and the enamel surface of the tooth is very subtly crenulated. The lingual surface bears a distinct lingual ridge along the vertical midline of the tooth flanked on both sides by a shallow depression. The tooth is determinate and the enamel surrounds the entire tooth.

The second lower incisor is more caniniform than the first lower incisor. It is marginally larger than the first lower incisor and single-rooted. It is monocuspid and somewhat mesiobuccally-compressed, so that the base of the crown is ovoid rather than circular. The crown tapers to a distally displaced, rounded apex, which gives the tooth a curved profile. The lingual ridge is less well developed than that in i1 and the mesial and distal edges of the tooth exhibit moderately well-developed carinae. The enamel on the labial surface is faintly crenulated.

The third lower incisor is substantially enlarged relative to the preceding incisors and more closely approximates the lower canine in size and shape. It is monocuspid and single-rooted; the root is continuous with the crown and bulges lingually to anchor the tooth. The base of the crown is ovoid in cross-section, and the crown tapers to form a distally-positioned apex. The tooth is not mesiodistally-compressed like the preceding incisors, and as such it does not feature a lingual ridge. The distal carina is moderately-developed and the mesial carina is weak. The enamel is strongly crenulated around the entire crown of the tooth, forming apicobasally-aligned ridges.

The anterior-most upper dentition (first and second upper incisors) of *Periptychus* is poorly known. The third upper incisor is almost identical to the third lower incisor in shape and size. It is a single-rooted tooth that closely approximates the canine in size and shape. NMMNH P-35194 preserves the third upper incisor in situ, which demonstrates that there is a diastema between I3 and CI that is approximately the same length as I3.

#### Canines

The upper and lower canines of *Periptychus* are relatively small, single-rooted, monocuspid teeth that are subequal to the premolars in height (Fig 12). The upper and lower canines are nearly identical; however, the base of the crown of the lower canine is slightly more buccolingually-compressed in comparison to the upper canine. The crown itself is erect, with rounded apices positioned slightly distal to the midline of the tooth. The mesial and distal carinae are indistinct. The enamel surrounds the tooth and exhibits distinct apicobasally aligned crenulations. The shape of the canines of *Periptychus* is consistent across all known specimens. However, there is some marked variation in size that is most noticeable in specimens affiliated with the larger *P*. *rhabdodon* morphotype. The canine dentition of *Periptychus* is not known from enough specimens to be able to comment on whether canine size is linked to sexual dimorphism.

#### Premolars

The premolars are more homogenous in their morphology than the anterior teeth, except for the first premolar that is noticeably simpler in form and monophyodont. Therefore, the following description begins with the first premolar (P1/p1) followed by description of the general morphology of the succeeding premolars (P2/p2, P4/p4, P5/p5), with a final section outlining differences between the succeeding premolars.

**Fig 13:**
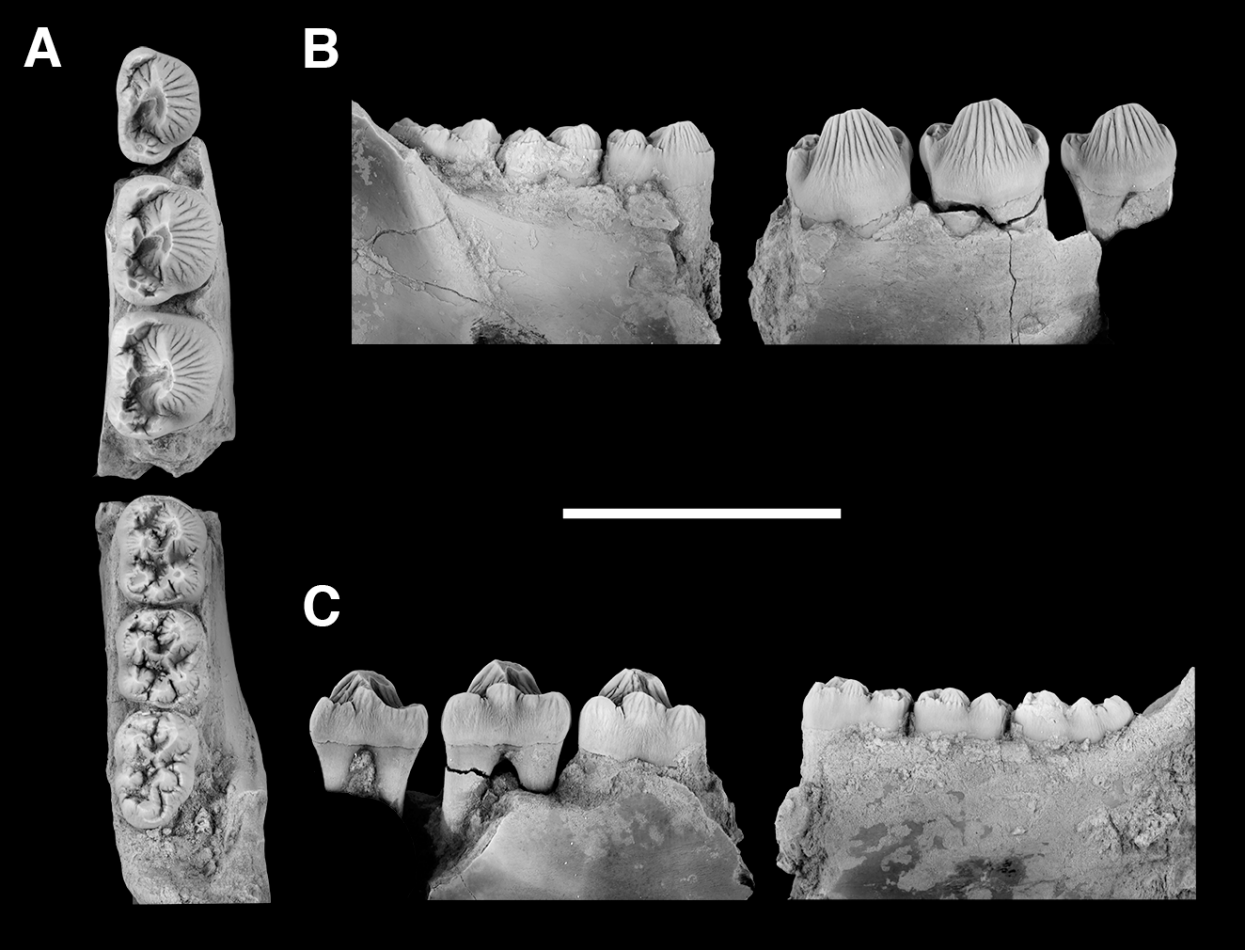
Lower dentition of *Periptychus carinidens* (NMMNH P-35194) with right p2-5, m1-3. (A) occlusal view; (B) buccal view; (C) lingual view. Scale bar: 30mm.

The lower premolars of *P*. *carinidens* are massive and distinctive (Figs 11C & 13). The lower premolars are larger than the molars in terms of surface area, and are considerably taller apicobasally. A large, centrally positioned protoconid dominates the tooth surface; the protoconid is lingually-flanked by a number of smaller cusps. The enamel surrounding the premolars is distinctly crenulated with well-developed apicobasally-aligned ridges on both the buccal and lingual surfaces of the teeth.

The first lower premolar is noticeably distinct from the more posterior premolars. It is a small single-rooted tooth, separated from the lower canine and second lower premolar by a small, but distinct diastema (less than the width of the p1). The tooth is dominated by an enlarged protoconid; the protoconid is roughly conical and buccolingually compressed so that the apex is somewhat spatulate, with distinct mesial and distal cristae. The mesial surface of the tooth exhibits a very faint cingulum with no evidence of a vestigial paraconid or metaconid (which develops in the succeeding lower premolars). There is a small cuspidate swelling on the distal edge of the tooth, which we interpret as a rudimentary talonid with two cuspules, which we identify as a vestigial hypoconid and hypoconulid, respectively.

The p2, p4 and p5 of *Periptychus* are double-rooted and massive, increasing slightly in size posteriorly along the tooth row. The proportional increase in successive premolar size for *Periptychus* (and also *Carsioptychus*) is not as great as that seen in *Ectoconus*; however, the premolars of *Ectoconus* are considerably smaller relative to the lower molars. As with the first premolar, the protoconid on p2-5 of *Periptychus* is enlarged relative to the other cusps on the tooth and occupies the entire buccal edge of the tooth. The protoconid is erect and somewhat buccolingually compressed. The lower premolars of *Carsioptychus* also possess an enlarged protoconid, which is strikingly posteriorly-pitched rather than erect as seen in *Periptychus*. There is some variation in the erection of the premolar protoconid of *Periptychus*. Older specimens from the early Torrejonian show a slight posterior pitch of the protoconid (albeit it not as extreme as that observed in *Carsioptychus*), whereas younger specimens are more vertically erect. The lower premolar protoconid morphology of both *Periptychus* and *Carsioptychus* differs from the lower premolar morphology in *Ectoconus*, in which the protoconid is somewhat enlarged relative to the other trigonid cusps, but is much more molariform in overall construction.

The mesial surface of the protoconid of *Periptychus* features a distinct mesiodistally-aligned cristid that we interpret as the paracristid; it descends the protoconid and forms a sharp notch before ascending to the much smaller paraconid. A second buccolingually-aligned cristid descends from the midline of the lingual surface of the protoconid to the metaconid. This feature is absent in *Carsioptychus* due to the lack of a distinct metaconid on the lower premolars. It is also absent in *Ectoconus* despite the presence of a large well-developed metaconid. A similar type of cristid morphology to that seen in *Periptychus* is present in the smaller, early Paleocene periptychid, *Haploconus*. A third mesiodistally-aligned cristid descends from the distal surface of the protoconid to close off the buccal edge of the talonid. We interpret this as the postprotocristid. The region of the protoconid between the postprotocristid and the protocristid forms an occlusal surface for the enlarged upper premolar paracone.

The lower premolar paraconid of *Periptychus* is positioned mesiolingual to the protoconid on the lingual margin of the tooth. It is the second largest cusp on the trigonid, although still substantially smaller than the protoconid (approximately half the height). The cusp is appressed against the protoconid, but still provides some mesiolingual expansion to the occlusal profile of the tooth. The paraconid in *Periptychus* is much more developed than that seen in both *Carsioptychus* and *Ectoconus*. In *Carsioptychus*, the paraconid is similarly positioned and appressed against the protoconid, but is greatly reduced; it is absent on p1 and p2 but forms a small circular cusp on a shallow mesiolingual cingulid on p4 and p5. In *Ectoconus* the paraconid increases in development posteriorly along the tooth row: on p1 and p2 it is absent, on p4 it is present as a small swelling and on p5 it forms a distinct circular cusp that is somewhat smaller than the metaconid, but that occupies a distinct area on the trigonid, giving the tooth a distinct molariform appearance.

The lower premolar metaconid of *Periptychus* is the smallest cusp on the trigonid, although it is positioned vertically higher than the paraconid and talonid cusps. It is situated directly lingual to the apex of the protoconid, with the protocristid traversing the two cusps to close off the trigonid. The metaconid region is much less lingually expanded than the paraconid and talonid, and as such, the paraconid and talonid encroach into the metaconid region. However, the metaconid does maintain lingual exposure. The metaconid is absent in *Carsioptychus*, giving the protoconid complete lingual exposure. In *Ectoconus*, the metaconid is present as a well-developed, distinct cusp positioned lingually relative to the protoconid; it is not as reduced relative to the protoconid as in *Periptychus*.

The premolar talonid of *Periptychus* is positioned distolingual from the protoconid and is offset from the mesiodistal midline of the tooth. The talonid region approximates the paraconid in size and position, so that in occlusal view the premolars are roughly symmetrical along a buccolingual line of symmetry that passes through the apices of the protoconid and metaconid. The talonid of *Carsioptychus* is somewhat lingually-shifted but does not possess the same symmetry as seen in *Periptychus* due to the lack of development of the paraconid region. In *Ectoconus*, the talonid forms a heel on the mesiodistal midline of the tooth.

The talonid of *Periptychus* is cuspidate, with small hypoconid, hypoconulid, and entoconid cuspids evident in varying degrees of development along the tooth row. The premolar hypoconid of *Periptychus* is small cusp positioned directly distal to the apex of the protoconid. In *Ectoconus* the hypoconid is also positioned directly distal of the protoconid, but is the largest cusp on the talonid (on p5 it is subequal in size to the paraconid). The hypoconulid of *Periptychus* is situated closer to the hypoconid than the entoconid on the distolingual margin of the tooth, rather than closer to the mesiodistal midline as seen *Ectoconus*. In *Ectoconus*, the hypoconulid is highly reduced so that hypoconid is the largest cusp on the talonid followed by the hypoconulid, and then the entoconid is the smallest. The entoconid of *Periptychus* is positioned mesial to the hypoconid on the lingual margin of the tooth. A sharp entocristid connects it to the metaconid via a postmetacristid. The relative size of the talonid cusps is variable along the tooth row and is described at the end of this section. In *Periptychus*, the cristid obliqua is very short and continuous with the postprotocrista. It borders a rudimentary shallow hypoflexid buccodistally. In *Ectoconus*, the cristid obliqua is absent due to the presence of the enlarged hypoconid. No cingulids are evident on the lower premolars of *Periptychus*. However, in *Carsioptychus* a narrow and sharp mesiolingual cingulid borders the paraconid region and in *Ectoconus* well-developed cingulids surround the lower premolar teeth. These are particularly deep and well developed on p5 in *Ectoconus*.

The lower premolars increase in size posteriorly along the tooth row. The protoconid becomes progressively more bulbous over the mesial root and the metaconid becomes more mesiodistally-expanded in succeeding teeth. The occlusal profile of the lower premolars develops posteriorly. On p2, the paraconid and talonid are small and appressed against the protoconid, but still form bulbous heels on the lingual margin of the tooth. More posteriorly along the tooth row, the paraconid and talonid are larger and less appressed against the protoconid (in comparison to p2); however, the protoconid has also expanded so that the profile of the posterior premolars appears less bulbous. The p5 is often shorter or subequal in length to the p4, but is larger in overall area as it is buccolingually wider.

The talonid becomes more developed posteriorly along the tooth row and the cuspules increase in definition. On p1 only a cuspidate talonid is evident, on p2 the hypoconid and hypoconulid are distinct cuspulids and are accompanied by a small rudimentary entoconid, and on p4 the entoconid is more developed and subequal in size to the hypoconid and hypoconulid. On p5, however, the entoconid is greatly reduced to little more than a cuspulid on the cristid between the hypoconulid and metaconid. The hypoconulid is positioned on the distolingual margin of the tooth rather than the distal midline. It is the largest cusp on the talonid and increases in size posteriorly along the tooth row.

**Fig 14:**
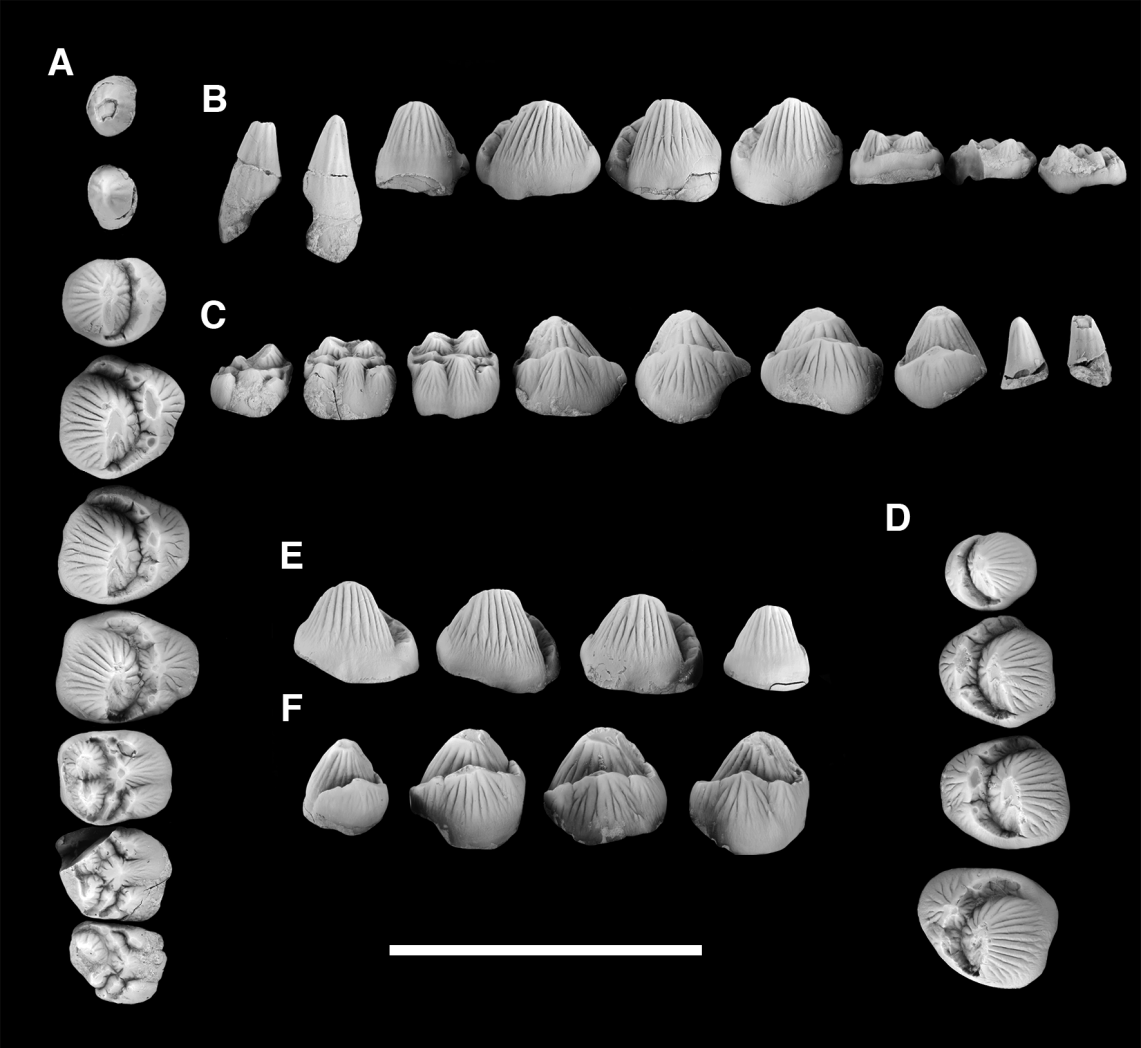
Upper dentition of *Periptychus carinidens* (NMMNH P-36631) with right I3, C, P1-5, M1-3 and left P1-5. **A-C, right I3, C, P1-5, M1-3.** (A) occlusal view; (B) buccal view; (C) lingual view; D-F left P1-5: (D) occlusal view; (E) buccal view; (F) lingual view. Scale bar: 30mm.

The upper premolars of *Periptychus* closely approximate the lower premolars in that they are massive and distinctive (Figs 11A & 14). The premolars are proportionally larger than the molars and bulbous. The paracone is conical, enlarged, and positioned centrally on the tooth. The protocone is reduced relative to the paracone, with the pre- and postprotocristae forming a mesiodistally-elongate crescentic cusp around the lingual surface of the paracone. In occlusal view, the teeth are approximately circular, nearly as wide as they are long, and in buccal aspect they are ‘plumb-bob’ shaped with a distinctly swollen base. The enamel is strongly crenulated with apicobasally-aligned, inverted ‘Y’ shaped plications surrounding the tooth.

The first upper premolar of *Periptychus* is noticeably distinct from the succeeding premolars. It is a small single-rooted tooth, separated from the upper canine and second upper premolar by diastema (less than half the mesiodistal length of p1). The paracone is the largest cusp on the tooth; it is erect and positioned centrally so that the buccal surface of the cusp appears slightly bulbous. The apex of the paracone is shifted lingually. However, it is not positioned as far lingually as in the succeeding premolars, and so the buccal slope of the paracone is not as long and the cusp appears more erect than in P2-P5. A moderately well-developed preparacrista descends from the paracone mesially and intercepts the preprotocristae on the mesial surface of the tooth. A more distinct postparacrista descends from the paracone distally (and more buccally relative to the preparacrista) to intercept the postprotocristae. Positioned lingually and slightly distal to the paracone is a small protocone (= deuterocone). The protocone is separated from the paracone by a distinct crescentic trough formed by the pre- and postprotocristae. The pre- and postprotocristae extend mesiodistally from the protocone, around the base of the paracone, and terminate where they intercept the pre- and postparacristae. The pre- and postprotocrista are not symmetrical; the mesial-most terminal portion of the preprotocrista is not as expanded as the terminal portion of the postprotocrista. Furthermore, the postprotocrista terminates more buccally than the preprotocrista and is demarcated by a small cuspule that is interpreted to be a vestigial metacone. Both the pre- and postprotocrista are slightly rugose, but do not feature any distinct cuspules.

The succeeding premolars (P2, P4 and P5) of *Periptychus* each possess three roots, with two roots supporting the paracone and a third supporting the protocone region. As with the first premolar, the paracone is enlarged, conical, and centrally placed. However, the apex of the paracone is positioned more lingully, increasing the buccal slope of the paracone. The paracone is somewhat distally-pitched, although not to the extremely high degree as in *Carsioptychus*. This subtle distal pitch of the upper premolars is present in specimens with erect lower premolar protoconids and is accentuated by the skewed morphology of the tooth. As with the lower premolars, the subtle posterior pitch is more pronounced in specimens from lower Torrejonian deposits. The apex of the paracone is blunted to form a wear facet with a distinct raised border from which several cristae descend. The paracone wear facet in *Periptychus* is rarely as highly worn as in specimens of either *Ectoconus* or *Carsioptychus*. In *Ectoconus* and *Carsioptychus*, the apical wear facet often encompasses the protocone.

In *Periptychus*, the preparacrista originates at the rounded mesial end of the wear facet and descends directly mesial to the parastylar region. The distal end of the facet is more unusual. The buccal edge of the facet is continuous with the postparacrista, which is well developed and descends distally. The lingual edge of the occlusal wear facet does not meet the buccal edge of the facet posteriorly; instead the lingual edge of the facet is continuous with a third crista which descends from the lingual surface of the paracone parallel to the postparacrista, leaving the facet distally open ended. The distinctive enamel crenulations are absent, or significantly less pronounced, between the postparacrista and secondary lingual crista. The preparacrista descends from the paracone mesiobuccally. It is broadly rounded and weaker than the postprotocrista and it terminates in a small parastyle (or on the parastylar region if parastyle absent/vestigial as on the anterior premolars) where it intercepts the preprotocrista. The postparacrista descends distally and terminates where it meets the postprotocrista. This confluence is demarcated by a small swelling, or mesiodistally-appressed cusp, that we interpret as a vestigial metacone.

The upper premolars of *Periptychus* bear some superficial resemblances to those of *Ectoconus* in their overall structure and size, with an enlarged and centrally placed and erect paracone positioned buccal to a much more-developed protocone (subequal in mesiodistal width to the paracone). However, the posterior-most premolars of *Ectoconus* are much less bulbous than *Periptychus*, with an almost vertical buccal slope and an overall molariform appearance that is accentuated by the presence of a large paraconule and slightly smaller metaconule. Furthermore, a vestigial postcingulum extends to the hypocone region on P5. The overall molariform morphology of the upper premolars of *Ectoconus* is more like that of *Hemithlaeus kowalevskianus* than of *Periptychus*.

The upper premolars of *Periptychus* possess a distinctive crescentic shoulder that surrounds the lingual surface of the paracone. The composition and nomenclature of the shoulder morphology depends on the cusp developed. On P1 it is formed by the pre- and postprotocristae. On P2-5 it is formed by the pre- and postprotocristae, the pre- and post cingulae, and the preparaconule crista and postmetaconule. *Carsioptychus* exhibits a similar morphology; however, in this taxon the pre- and postprotocristae form a buccolingually-deep, semi-circular shoulder that is more lingually-expanded and mesiodistally-constricted than in *Periptychus*, so that its mesiodistal length is less than that of the paracone (in *Periptychus* the shoulder is longer than the paracone, giving the tooth an almost circular occlusal profile). Premolar pre- and post cingulae are present in *Periptychus* (contra to [18,28]) and contribute to forming the crescentic shoulder. Premolar pre- and post-cingulae are also present in *Carsioptychus*, but do not contribute to the semi-circular shoulder. The distinctive crescentic shoulder present in *Periptychus* and *Carsioptychus* also bears notable similarity to the premolar morphology of *Anisonchus*, which also possesses a similar crescentic configuration, although in this taxon the cingulae are rather more developed on the mesial and distal faces of the talonid.

In *Periptychus*, the protocone is the largest of seven cuspules on the crescentic shoulder surrounding the paracone. It is positioned slightly off-centre (mesially) from the paracone, to give the tooth a skewed appearance. The base of the protocone is somewhat lingually expanded, giving the tooth a circular profile in occlusal aspect. However, it does not form as prominent a lingual bulge as in *Carsioptychus* or *Ectoconus*. Considering the progressive development of the dental morphology posteriorly along the tooth row, the cusps can be interpreted as follows: parastyle, paraconule, protostyle, protocone, hypocone, metaconule and vestigial metacone (from mesial to distal end). The cristae/cingulae between the cusps are interpreted as follows: parastyle-preparaconulecrista-paraconule-precingulum-protostyle-preprotocrista-protocone-postprotocrista-hypocone-postcingulum-metaconule-postmetaconule crista-metacone. The postparaconulecrista and premetaconule cristae are greatly reduced relative to the preparaconule and postmetaconule cristae.

The cuspule interpreted as a metaconule is very weakly developed and often absent or reduced to a small swelling. Its development appears to be dependent on the development of the metacone. The protostyle and hypocone are subequal in size and slightly smaller than the protocone. The paraconule is smaller than the protostyle and hypocone but larger than the metaconule. Contrary to the morphology seen on P1, the preprotocrista is more mesially expanded than the postprotocrista, forming a deeper shelf that extends farther buccally and descends further ventrally.

The upper premolars of *Periptychus* increase in size and development posteriorly but otherwise remain homogenous in their overall morphology. It should be noted that P5 is larger than P4 in terms of overall occlusal area, but P4 is slightly mesiodistally longer. The apex of the paracone shifts lingually so that the buccal slope of the paracone increases in length from P1 to P5. The base of the protocone also becomes more buccally expanded, but the apex does not shift its position. The pre- and postprotocristae become increasingly cuspidate posteriorly along the tooth row as previously described.

#### Molars

The molars are comparatively homogenous in their morphology. The following description will describe the general morphology of the lower and upper molars, with a concluding section after each to differentiate between the individual teeth.

The lower molars of *Periptychus carinidens* are large and bunodont (Figs 11D & 13). The trigonid is composed of three principal cusps. The protoconid is the largest molar cusp and is positioned on the mesiobuccal border of the tooth, opposite and between the paraconid and metaconid. It exhibits a buccally-expanded base so that the cusp apex is situated lingually from the buccal tooth perimeter, creating a long buccal slope to the protoconid. In *Carsioptychus*, the protoconid is proportionally larger relative to the other trigonid cusps and is positioned opposite the metaconid, and the paraconid is mesially-positioned. The protoconid of *P*. *carinidens* exhibits the bizarre ‘skewed’ morphology seen on the other postcanine teeth where four cristids descend from the cusps, creating a distolingual occlusal facet. This feature is not evident in *Ectoconus* or *Carsioptychus*. The paracristid descends mesiolingually, creating a sharp notch between the protoconid and paraconid on the mesial edge of the tooth. The protocristid mirrors the paracristid and descends from the protoconid to the metaconid distolingually, creating a notch. The cristid obliqua intercepts the protocristid at the notch. A distinct postprotocristid descends from the distobuccal side of the protoconid to the ectocingulid, and the point of confluence between the postprotocristid and ectocingulid is sometimes marked by a small cuspid (best seen on m3). The cuspid is situated on the ectocingulid, not on the cristid obliqua, and therefore it cannot be interpreted as a mesoconid. This cuspid is also distinctly present in *Carsioptychus* and to a lesser extent in *Ectoconus*. A fourth cristid descends from the lingual surface of the protoconid; it is positioned mesial to the protocristid and runs parallel to it, forming a mesial border for the occlusal facet.

In *Periptychus*, the paraconid is a small, erect cusp on the mesiolingual margin of the tooth. It is the smallest cusp on the trigonid. In *Carsioptychus*, the paraconid is positioned closer to the mesiodistal midline of the tooth and is much more closely appressed against the other trigonid cusps. The metaconid of *Periptychus* is positioned distal and separate from the paraconid and distolingual to the protoconid, forming a deep valley between the two aforementioned cusps. In contrast, in *Carsioptychus*, the metaconid and protoconid are much more closely appressed with only a shallow valley separating them. The metaconid in *Periptychus* is taller and more lingually-expanded in comparison to the paraconid, but remains small relative to the protoconid. There is no evidence of a cusp on the mesiobuccal cingulid (mesiobuccal cingular cusp f).

The position of the trigonid cusps and their cristids creates a mesiodistally-compressed trigonid configuration, whereby the angle between the paracristid and protocristid is less than 50°. This is most pronounced on m2, where the angle is less than 35°. The trigonid cusp configuration of *Carsioptychus* appears relatively consistent along the tooth row, with the angle between the paracristid and protocristid set at 35°. However, it should be noted that the molars of *Carsioptychus* are typically highly worn, making measurements somewhat subjective. The trigonid configuration of *Ectoconus* is also consistent along the tooth row, although the trigonid is relatively much wider than in either *Periptychus* or *Carsioptychus*, with the angle between the paracristid and protocristid set at 50°. In *Periptychus*, a sharp postmetacristid extends distally from the metaconid and converges with the entocristid on the talonid to form a notch and partially close off the talonid basin. In both *Periptychus* and *Ectoconus*, the base of the talonid is positioned slightly lower than the base of the trigonid. However, the talonid cusps approximates the paraconid and metaconid in height, providing a relatively level occlusal surface, so that the trigonid cusps are twice the height of the talonid cusps. In *Carsioptychus*, the trigonid is positioned considerably higher relative to the talonid than in *Periptychus* (and *Ectoconus*).

The trigonid of *Periptychus* is mesiodistally compressed relative to the length of the talonid on m1-2. The talonid of m3 is not compressed and forms an elongate heel. In *Ectoconus*, the trigonid and talonid are subequal in mesiodistal length and on m3 the talonid forms a heel that is only marginally longer than the trigonid, so that the occlusal profile of the m3 of *Ectoconus* is much less elongate than in *Periptychus*.

The talonid of *Periptychus* forms a multicuspidate basin bordered by three well-developed cusps that approximate the trigonid in width and height. The hypoconid is a prominent and distinct cusp on the buccal border of the talonid that is subequal, to slightly larger than, the entoconid in size (both cusps are larger than the hypoconulid). The hypoconid is positioned distally on the trigonid, closer to the hypoconulid than protoconid, and opposite the entoconid. This is somewhat like the hypoconid in *Carsioptychus* and notably different in *Ectoconus*. In *Carsioptychus*, the hypoconid is consistently the largest cusp on the talonid and is substantially larger than the entoconid, positioned distally on the trigonid, but is marginally medial to the entoconid (particularly on the m3) rather than opposite. In *Ectoconus*, the hypoconid is the largest cusp on the trigonid, and in some cases, it approximates the protoconid in size (e.g., AMNH 16500). It is positioned midway between the protoconid and hypoconulid, mesial to the entoconid, and set in from the buccal border of the tooth so that the cusps are much more closely-appressed, forming a smaller talonid basin than in *Periptychus*.

The hypoconulid of *Periptychus* is a short and erect cusp positioned on the distal midline of the tooth. It is the smallest cusp on the trigonid and is nearly the same size as the paraconid. The hypoconulids of *Carsioptychus* and *Ectoconus* are both like *Periptychus* in that they also form a small, erect cusp on the distomedial border of the molar teeth. In *Periptychus*, a sharp cristid extends from the hypoconulid mesiobuccally towards the hypoconid, where it variably terminates at the hypoconid or slightly mesiolingual to the hypoconid, on the cristid obliqua. In *Carsioptychus*, this cristid is much shorter and only extends to the distolingual surface of the hypoconid (which is much larger than in *Periptychus*); in the specimens observed, it never contacts the cristid obliqua.

The entoconid of *Periptychus* is a large cusp on the talonid. On m1 and m2 it is variably subequal to, to very slightly larger than, the hypoconid. It is subequal to the metaconid on m2, and on m3 it is the largest cusp on the trigonid. It is situated on the lingual border of the talonid immediately opposite the hypoconid. In *Carsioptychus*, the entoconid is consistently larger than the hypoconid in size, whereas in *Ectoconus* it is consistently smaller among all molars.

The molar cristids of *Periptychus*, and also *Carsioptychus*, are well defined although are not as strong as those seen on the molars of *Ectoconus*. In *Periptychus*, a distinct cristid obliqua extends from the hypoconid mesiolingually to intercept the protocristid notch on the mesiodistal tooth midline where it variably forms a discrete cusp which is termed an obliconid [97]. The presence and development of an obliconid is variable in *Periptychus* but consistently absent in *Carsioptychus*. The cristid obliqua of *Periptychus* is relatively elongate in comparison to *Carsioptychus*, with a more defined junction with the protocristid (whether marked by an obliconid or not). A second de novo crest extends mesioventrally from the hypoconid towards the buccal cingulid on *Periptychus* and is best observed on m2. A homologous hypoconid de novo crest is absent in all other periptychids including *Carsioptychus*. In *Periptychus*, a deep hypoflexid is present in the space between the cristid obliqua, postprotocristid and buccal cingulid. The hypoflexid of *Periptychus* is proportionally more developed than those seen in either *Carsioptychus* or *Ectoconus* (particularly on m1 and m2) and forms a deep pit.

The entocristid of *Periptychus* is weakly developed, extending ventromesially from the entoconid. A second, more developed de novo cristid extends mesially, and slightly buccally relative to the entocristid, from the entoconid to intercept the distally directed postmetacristid. The exact homology of these crests is somewhat ambiguous. For now, we interpret the lingual most crest extending from the entoconid as the entocristid and the more internal crest as an entoconid de novo cristid. An entocristid and entoconid de novo crest are present in numerous periptychids including *Carsioptychus* (variable and weak), *Ectoconus*, and *Conacodon*, whereas it is notably less developed to absent in others, such as *Hemithlaeus*, *Oxyacodon*, *Mithrandir* and *Anisonchus*.

The talonid cusps of *Periptychus* form an open configuration. The angle formed by vectors from the hypoconulid to the entoconid and hypoconid is greater than 130° on m1 and m2, and it reduces to 85° on m3. The mesiodistal position of the entoconid and hypoconid relative to the protoconid and metaconid is somewhat variable, but is always more distally positioned in comparison to *Carsioptychus* with a more open talonid basin. The cusp configuration of *Carsioptychus* is notably tighter and less variable along the tooth row; it measures 100° on m1 and m2 and 90° on m3. *Ectoconus* displays a similar configuration to *Carsioptychus*; it measures 100° on m1 and m2 and 75° on m3. The differences in cusp configuration appear to be associated with the variability in hypoconid size and position, as the entoconid position remains relatively consistent along the tooth row.

The buccal cingulid of *Periptychus* varies in development along the tooth row, becoming increasingly well developed and cuspidate distally. On m1 it is little more than a shallow shoulder on the mesiobuccal portion of the protoconid; on m2 it forms a narrow shelf that is continuous along the buccal edge of the tooth; and on m3 it forms a deep cuspidate shelf that is continuous around the buccal edge of the tooth. The cuspidate buccal cingulid on the m3 exhibits two distinct cuspules: one between the hypoconulid and entoconid and a second between the entoconid and metaconid. These cusps are present in *Periptychus* but noticeably more developed in *Carsioptychus*. A buccal cingulid is also present in *Ectoconus*, but appears less sharp. In *Periptychus*, the lingual cingulid is much less developed and forms little more than a shoulder on all the molars, except between the paraconid and metaconid, where it forms a narrow shelf. *Carsioptychus* displays a very similar trend to *Periptychus*, with the buccal cingulid becoming progressively more developed on the molars posteriorly and the lingual cingulid being only weakly developed. *Ectoconus* is somewhat similar in that the cingulids are increasingly developed posteriorly. However, they form deeper cuspidate shelves on both the buccal and lingual margins of the molars, giving the molar cusps a highly transversely appressed appearance.

In *Periptychus* the ultimate lower molar is the largest of the lower molars, and it is slightly larger than m1; both m1 and m3 are considerably larger (approximately 15%) than m2. The trigonid becomes progressively more compressed posteriorly: on m1 the angle formed between the paracristid and protocristid is approximately 50°, and on m2 and m3 this decreases to 35°. The talonid does not follow this trend. It is shorter on m1 and m2 (making up less than 50% of the overall tooth length), but is relatively longer on m3, where it is approximately 75% of overall tooth length. The changes in relative trigonid and talonid proportions cause the hypoflexid to become shallower posteriorly. Cusp appression increases posteriorly along the tooth row; the cusps move inwards from the tooth perimeter and the tooth base appears more bulbous as a result. On all the molar teeth, the buccal cusps form a relatively long buccal slope, whereas the lingual cusps are much more erect and form a steep wall rather than a slope.

Despite the changing size of teeth along the tooth row (m1>m2<m3), the cusps progressively decrease in size posteriorly due to the increasing cusp appression. The portion of space occupied by the protoconid reduces in size posteriorly along the tooth row, although the height of the protoconid remains relatively subequal. The paraconid sits on the lingual margin of the tooth, but shifts more towards the mesiodistal midline of the tooth from m1-3. The hypoconulid increases in size and development posteriorly along the tooth row so that on m3 it is subequal in size to the other cusps on the talonid, situated on a distinct heel with a cuspidate postcristid between the hypoconulid and entoconid.

The upper molars are bunodont and roughly quadrate in occlusal profile (Figs 11B & 14). Although they are smaller than the upper premolars, they are still relatively massive with inflated, conical cusps that converge apically. Each tooth possesses three roots, two supporting the trigon and one supporting the talon. The upper molars of *Periptychus* are buccolingually compressed in occlusal profile in comparison to those of *Carsioptychus*, which are more transversely expanded, particularly in the talon region, to give a subtriangular occlusal profile.

In *Periptychus*, the paracone and metacone are positioned on the buccal border of the upper molar teeth. The paracone is marginally taller than the metacone and more buccally expanded, so that in occlusal aspect the tooth profile exhibits a shallow ectoflexus. *Carsioptychus* displays a somewhat similar condition; however, the size difference between the paracone and metacone is less marked and the buccal border exhibits only a very shallow ectoflexus. In contrast, in *Ectoconus*, the ectoflexus is strongly developed, particularly on M2. The paracone of *Periptychus* is conical and features a similar morphology to the premolar paracone; the preparacrista and centrocrista are mesiodistally aligned with a third crista descending from the paracone, lingually towards the protocone. The third crista is not as well developed as the preparacrista and centrocrista, but demarcates an excavated occlusal facet on the distolingual surface of the paracone as also seen on the premolars. A similar but consistently weaker crista is variably present on the metacone, extending to the protocone. The paracone-protocone crista is more defined than the metacone-protocone crista and appears to become more prominent posteriorly along the molar series. The metacone-protocone crista is more variable in its presence and tends to become more prominent posteriorly along the molar series. The presence of either crista does not appear to depend on the other. A preparacrista descends from the paracone mesially and intercepts the paracingulum to variably form a small parastyle. The crista itself is not cuspidate. *Carsioptychus* exhibits a similar paracone morphology to *Periptychus*, but the cristae are much less salient and the parastyle is highly reduced or absent.

The upper molar stylar shelf of *Periptychus* is sharp and narrow. The presence of stylar cusps is variable. A parastyle (= stylar cusp A) and the metastyle (= stylar cusp E) are generally present; a mesostyle (= stylar cusp C) may also be observed, particularly on M1 or M2, but is less common than a parastyle or metastyle. The stylar shelf of *Periptychus* is relatively less-developed as that in *Carsioptychus*, which tends to be deeper. Both *Periptychus* and *Carsioptychus* possess a much weaker and shallower stylar shelf in comparison to *Ectoconus*. In *Periptychus*, the metacone is situated very slightly lingual of the paracone and is slightly lower and less buccally expanded despite occupying roughly the same surface area of the tooth (the same arrangement is observed in *Carsioptychus*). The postmetacrista of *Periptychus* is smooth and descends from the metacone distally to intercept the metacingulum, where it forms a small metastyle. A highly reduced metastyle is observed in *Carsioptychus* at the base of a comparatively less salient postmetacrisa. In *Periptychus*, the paracone and metacone are joined at their bases to form a ‘V’ shaped valley as viewed buccally. In occlusal view, a mesiodistally-straight centrocrista traverses the aforementioned valley. A similar centrocrista is observed in *Carsioptychus*, but it is not as deeply incised as the condition in *Periptychus*.

In *Periptychus*, the paraconule and metaconule are small (occupying less than one third of the tooth width), but are well developed on all three molars. In *Carsioptychus* the paraconule and metaconule are reduced in comparison to *Periptychus*, but positioned in the same general arrangement. In contrast, in *Ectoconus*, the conules are relatively more developed and nearly subequal in size to the protocone. The paraconule of *Periptychus* is formed by a single prominent cusp positioned slightly closer to the protocone than the paracone. The pre- and postparaconule cristae are relatively weak (in comparison to the other molar cristae), forming wing-like extensions from the conule. The metaconule is slightly larger than the paraconule, forming a prominent cusp, positioned midway between the metacone and protocone with wing-like pre- and postmetaconule cristae. The preparaconule and postmetaconule cristae are stronger than their internal counterparts (the postparaconule and premetaconule cristae respectively) and intercept the pre-/postcingulae lingual to the paracone/metacone. Both conules are positioned on the same mesiodistal axis of the tooth but are externally offset (paraconule mesially, metaconule distally) from the geometrical segment between the associated buccal cusp and protocone, so that the paraconule is positioned mesial to the paracone-protocone segment and the metaconule is positioned distal to the metacone-protocone segment.

The upper molar protocone of *Periptychus* is a small erect cusp that is lower than both the paracone and metacone, and only slightly higher than the conules. The apex of the protocone is shifted buccally and possesses an elongate lingual protocone slope. The protocone of *Periptychus* shows a small degree of variation in its position relative to the hypocone and protostyle: it may be aligned or positioned more buccal relative the hypocone and protostyle. In *Carsioptychus*, the protocone is generally nearly in line with the hypocone and protostyle, whereas in *Ectoconus* the protocone is nearly in line with the para- and metaconule, and the hypocone and protostyle are positioned well lingual of the protocone. Despite the more lingual position of the protocone in *Carsioptychus*, the lingual protocone slope is proportionally broader than that in *Periptychus*. In *Periptychus*, the pre- and postprotocristae are well defined and extend to the conules, creating a small trigon basin. In *Carsioptychus*, the trigon basin is proportionally larger due to the more lingual position of the protocone.

In *Periptychus*, the protocone is flanked by a distinct protostyle on its linguomesial side. The protostyle forms a circular cusp on the precingulum. A small secondary cuspule is evident on some specimens, particularly on M2-3 (see NMMNH P-36631 [Fig 14]). It is roughly subequal in size to the conules, with the base of the protostyle extending lingually beyond the base of the protocone (although the protocone retains lingual exposure). In *Carsioptychus*, the protostyle is positioned mesial to the protocone and is slightly reduced in size in comparison to *Periptychus*. Unlike *Periptychus*, the base of the protostyle in *Carsioptychus* does not extend lingually beyond the base of the protocone.

In *Periptychus*, the protocone is distally flanked by a very well-developed hypocone. The hypocone is formed by a distinct circular cusp situated on the postcingulum; in unworn teeth, a second slightly smaller cusp is variably present on the postcingulum just buccal of the hypocone. The base of the hypocone extends lingually beyond the base of the protocone and protostyle, but does not envelop it. In *Carsioptychus*, the hypocone forms a single cusp which is relatively more developed in comparison to the protostyle (whereas in *Periptychus* the hypocone and protostyle are subequal in development). As in *Periptychus*, the base of the hypocone of *Carsioptychus* extends lingually beyond the base of the protocone and protostyle, but does not envelop it. In *Ectoconus*, the bases of the hypocone and protostyle are strongly reduced in comparison to both *Periptychus* and *Carsioptychus*, with the protocone base retaining a proportionally larger area of lingual exposure. In *Periptychus*, the pre- and postcingulae extend from the protostyle and hypocone to the parastyle and metastyle, respectively, forming a broad shelf along the mesial and distal borders of the tooth. The pre- and postcingulae in *Carsioptychus* are relatively less developed than in *Periptychus*, forming a weaker shelf which extends to the position of the parastyle and metastyle. In both *Periptychus* and *Carsioptychus*, the upper molars lack an endocingulum.

The upper molars are homogenous in their morphology along the tooth row. However, there are a few distinct differences and trends. The second molar is the largest tooth and is slightly larger than M1; M3 is the smallest molar and it is considerably smaller than both M1 and M2. The paracone region becomes progressively more buccally-expanded along the tooth row, although it never forms a distinct lobe. On the other hand, the metacone region becomes progressively more reduced, particularly on M3, in which the metacone is significantly smaller than, and is positioned lingual to, the paracone. The hypocone and protostyle are present and well developed on all three upper molars. The secondary hypocone cusp becomes more distinct distally from M1, where it is a small swelling, to M3, where it is a distinct cusp that is subequal in size to the true hypocone. The protocone gains more lingual exposure posteriorly as the hypocone and protostyle expand and diverge to give the upper molars a more quadrate occlusal profile.

#### Deciduous dentition

**Fig 15:**
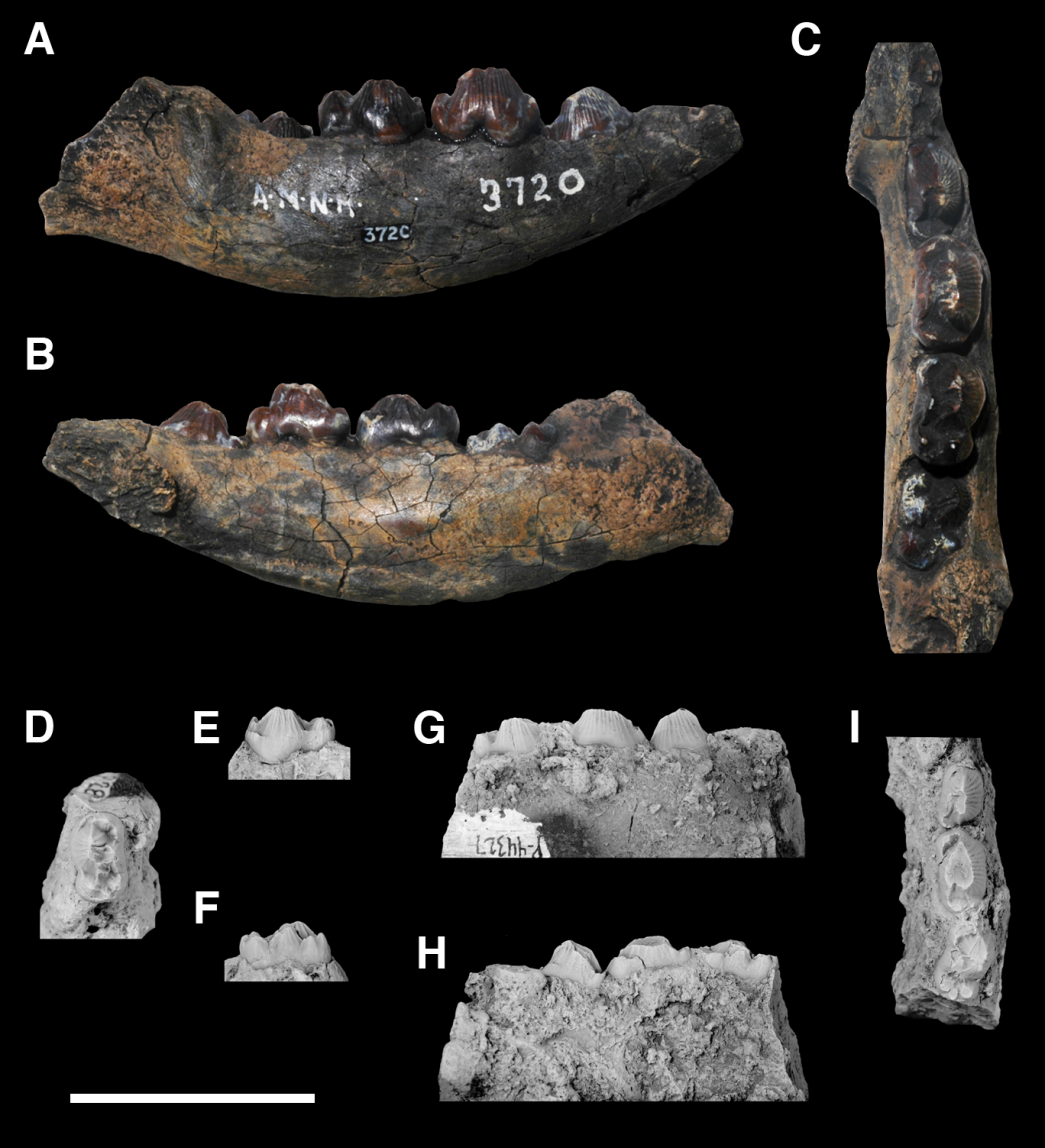
Lower deciduous dentition of *Periptychus carinidens*. AMNH 3720, a left dentary with dp2, dp4-5 and a partially erupted m1 (A-C): (A) buccal view, (B) lingual view; (C) occlusal view. NMMNH P-2870, an isolated left dp5 (D-F): (D) occlusal view; (E) buccal view; (F) lingual view. NMMNH P-44327, a partial right dentary with dp2-5 (G-I): (G) buccal view; (H) lingual view; (I) occlusal view. Scale bar: 30mm.

*Periptychus* exhibits a diphyodont condition characterised by the single replacement of deciduous dentition. Only the deciduous premolar dentition is known for *Periptychus*. The deciduous incisors and canines have yet to be positively identified, and their relative lack of distinguishing characters relative to the adult dentition inhibits identification of isolated deciduous anterior teeth. The molars are part of the permanent dentition and are never replaced [98,99]. The first premolar of *Periptychus* does not appear to have a deciduous precursor, as is common in some placental groups [98] and dP3/dp3 is absent in *Periptychus*; therefore, the deciduous premolars present and described are dP2/dp2, dP4/dp4, dP5/dp5. The following description of the lower deciduous dentition is based on an assortment of mandibular fragments. NMMNH P-19224 consists of left dp2 and dp4. NMMNH P-44327 is a right mandibular specimen preserving dp2-5 (Fig 15G–15I). AMNH 3720 is a relatively unworn left mandibular specimen preserving a partially erupted dp2, fully erupted dp4-5, and an m1 that is only slightly erupted (Fig 15A–15C). AMNH 15937 consists of a right mandibular fragment with dp2-5, a fully erupted m1 and a partially erupted m2 (Fig 2D–2F). AMNH 3637 is a right mandibular fragment with highly worn dp2-5 and fully erupted m1-3 (Fig 2A–2C). AMNH 3620 includes the right dp5 (Fig 1) and left dp4. NMMNH P-2870 is an isolated left dp5 (Fig 15D–15F).

The second deciduous lower premolar of *Periptychus* is only slightly dissimilar from its permanent replacement, but differs in several important characteristics. The tooth is strongly buccolingually-compressed (more so than any of the permanent postcanine dentition). The crown is dominated by a central and enlarged protoconid, with the remaining cusps either highly reduced or absent. The protoconid is not as swollen as in the permanent p2, and exhibits a reduced buccal slope. The cusp exhibits a subtly twinned apex with a strong mesial paracristid and distal postprotocristid. The protocristid is relatively weak due to a reduced metaconid. The paraconid is highly reduced, forming little more than a cingulid on the mesiolingual surface of the protoconid. The metaconid is also highly reduced; it often forms a vestigial cingulid, rather than a discrete cusp, lingual to the protoconid, but in some specimens, is completely absent (e.g., AMNH 15937).

The talonid heel of dp2 is proportionally larger than on the p2, so as to form a buccolingually-broader heel positioned distal to the protoconid rather than distolingual as on p2, giving the tooth a submolariform appearance. Due to the reduction of the paraconid and lingual shift of the talonid, the occlusal profile of dp2 lacks the symmetry seen in p2. The talonid of dp2 is cuspidate, but a discrete hypoconid, hypoconulid, or entoconid are not discernible.

The penultimate deciduous lower premolar is larger and more developed, but generally like the second, in its morphology. The protoconid is large, but remains strongly buccolingually-compressed, with a twinned apex. The paracristid, postprotocristid, and protocristid are stronger than on dp2 due to the development of the paraconid and metaconid. The paraconid forms a small, separate cusp on the mesiolingual border of the tooth. It is relatively more developed than the cingulid expansion observed on dp2, but is not as developed or mesiolingually expanded as the paraconid on its permanent replacement. The paraconid is strongly appressed against the protoconid, but still provides some mesiolingual expansion to the occlusal profile of the tooth. The metaconid forms a small distinct cusp on the lingual surface of the protoconid, and is positioned vertically higher than the paraconid and talonid cusps, as also is seen on the permanent premolar dentition.

The talonid heel of dp4 is positioned distally from the protoconid, but is slightly more lingually-expanded than the condition exhibited on dp2. The lingual expansion and paraconid region and the talonid provides an occlusal profile that bears some resemblance to p4, but still lacks the symmetry observed on the permanent premolars. The talonid region of dp4 is mesiodistally reduced relative to the mesiodistal length of the protoconid, and is buccolingually broad to form a broad heel.

The talonid cusps are more developed than the cuspidate condition observed on dp2, with a small hypoconid and entoconid and a larger hypoconulid. The hypoconid is slightly larger than the entoconid and positioned distal to the apex of the protoconid and buccodistal to the entoconid. The hypoconulid is larger (subequal to the paraconid in development) and is positioned slightly lingual to the mesiodistal midline of the tooth, closer to the hypoconid than the entoconid. The entoconid is small, forming a small cuspule on the entocristid, which extends to the metaconid via a postmetacristid. A short cristid obliqua is observed on the dp4. It is continuous with the postprotocrista and borders a rudimentary hypoflexid.

The ultimate deciduous lower premolar is distinctly molariform, and so it more closely resembles m1 than dp4 or its permanent replacement. Although dp5 is molariform, it is distinctly buccolingually compressed as is characteristic of the deciduous dentition of *Periptychus*. The protoconid is still the largest cusp with a twinned apex, but it is considerably reduced to that in dp4 and p5. The cristid obliqua intercepts the protocristid to form an obliconid, which is also observed on the molars of *Periptychus*. The postprotocristid descends from the protoconid, on the buccodistal surface, to the ectocingulid. In the molar teeth, the confluence of the postprotocristid and ectocingulid is variably marked by a small cuspule, which is not present on any of the dp5 specimens we observed. A fourth cristid descends from the protoconid on its lingual surface. It is positioned mesial to the protocristid and extends parallel to it, forming a mesial border for the occlusal facet as observed on the permanent dentition.

The paraconid is relatively well developed on dp5 compared to the preceding deciduous premolars. On NMMNH P-2870, it forms a large, twinned cusp on the mesiolingual margin of the tooth. The metaconid is also relatively well developed. It forms a discrete cusp positioned lingual and separate from the protoconid in a characteristically molar configuration, rather than tightly appressed against the molars as observed on the preceding deciduous premolars and on the permanent premolars. The metaconid is higher and more lingually-expanded in comparison to the paraconid, but is still small relative to the protoconid.

The talonid of dp5 forms a multicuspidate basin bordered by three well-developed cusps. The talonid is approximately half the height of the trigonid. The hypoconid is a well-developed, distinct cusp on the buccal border of the talonid and is subequal to slightly larger than the entoconid in size (both cusps are larger than the hypoconulid). The hypoconulid is short and erect, and positioned on the distal border of the talonid, slightly lingual to the mesiodistal midline of tooth. The entoconid forms a large, discrete cusp on the lingual border of the talonid immediately opposite the hypoconid. A deep hypoflexid is present in the space between the cristid obliqua, postprotocristid and buccal cingulid.

**Fig 16:**
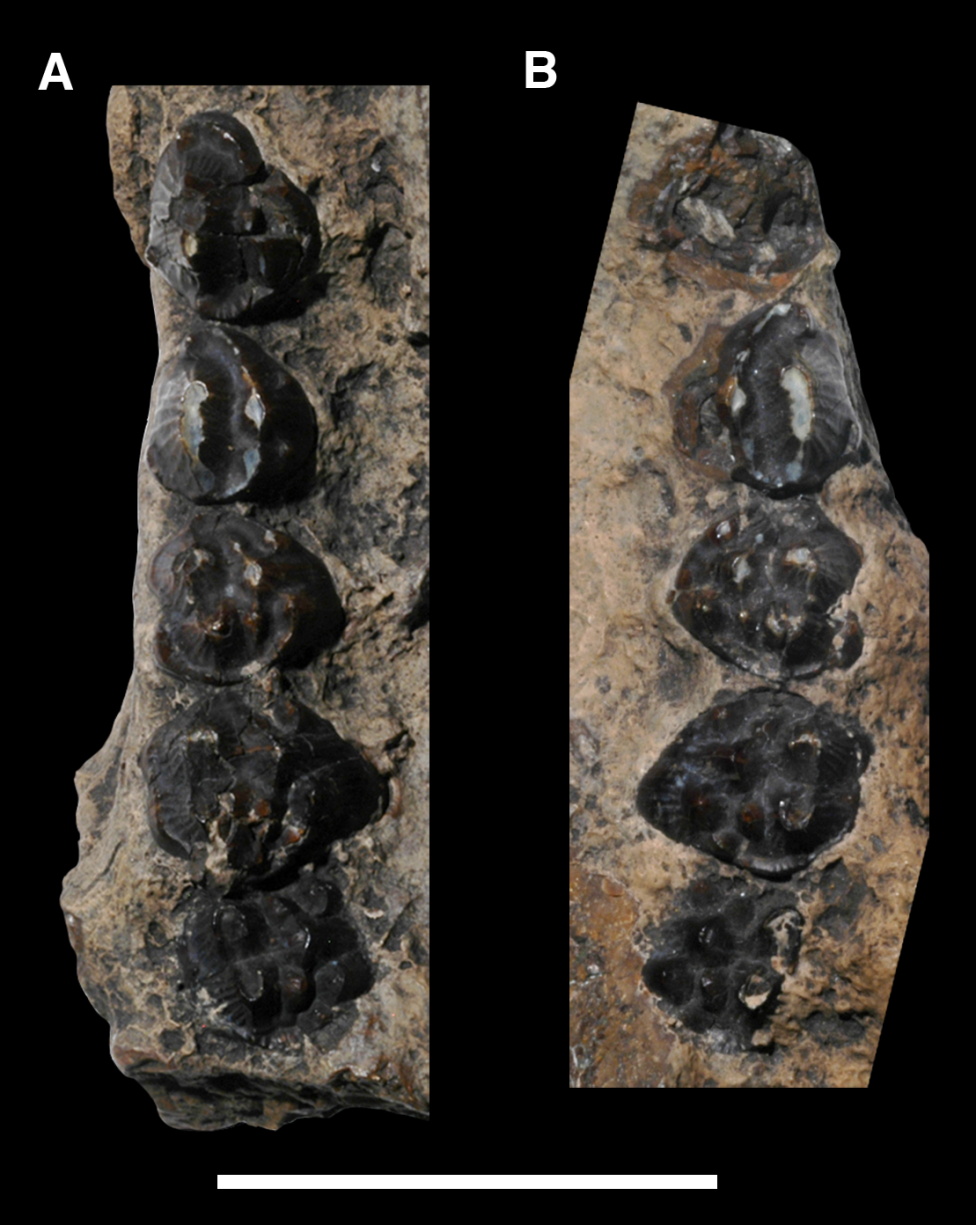
Upper deciduous dentition of *Periptychus carinidens* (AMNH 3794). (A) right dP2-5, M-2 in occlusal view; (B) left dP4-5, M1 and a partially erupted M2 in occlusal view. Scale bar: 30mm.

The deciduous upper premolars are described based on AMNH 3794, a palate preserving the right dP2-5, M1-2 and the left dP4-5, M1 and partially erupted M2 (Fig 16).

The second deciduous upper premolar is generally comparable to its permanent replacement, but exhibits several deviations from the characteristic premolar configuration of *Periptychus*, which sheds some light on the development of these unusual teeth. The paracone is typically enlarged relative to the other cusps on the tooth, but is buccolingually compressed in comparison to P2, and possesses a twinned apex. The preparacrista extends from the paracone mesially to an expanded parastylar region. The parastylar region forms a mesiolingual cuspidate shelf, but appears to lack a discrete parastyle. The postparacrista is well developed and descends distally to terminate in a small metacone (the metastylar region is not as expanded as the parastylar region). On dP2, the preparacrista and postparacrista are mesiodistally aligned, whereas on P2 both crista extend mesio-/disto- buccally, respectively, due to the increased expansion and lingual shifting of the paracone. On dP2, a third, substantially weaker crista descends the lingual surface of the paracone parallel to the postparacrista, forming the distinctive occlusal facet that is also present on the permanent upper premolar. However, on dp2, this facet is not as strongly defined as on its permanent replacement.

The pre- and postprotocristae form a distinctive lingual crescentic shoulder around the paracone, as is typical of *Periptychus* premolars. However, the shoulder is not continuous with the parastylar and metastylar regions as observed on the permanent premolars. On AMNH 3794, the shoulder is abruptly interrupted where it meets the parastylar region. This appears to be a genuine feature of the tooth rather than damage, as a much weaker interruption is observed on dP4 and in the permanent premolars.

The crescentic pre-/postprotocrista shoulder exhibits two discrete cusps, but lacks the continuous cuspidate configuration observed on the permanent premolars. The mesial-most cusp is the smaller of the two and is positioned towards the lingual base of the paracone. The second, more distal cusp is larger (approximately twice the size of the more mesial cusp) and is positioned more lingually. Considering the progressive development of the dental morphology along the tooth row, we interpret these cusps as the paraconule and protocone, respectively.

The dp4 exhibits a more molariform morphology compared to dP2. The paracone is enlarged, buccolingually compressed, and possesses a twinned apex as seen on dP2. The cristae are much the same as on dP2. The parastyle forms a distinct cusp where the preparacrista meets the preparaconule crista. The pre- and postprotocristae form a distinctive lingual crescentic shoulder around the paracone in conjunction with the preparaconule crista and postmetaconule crista, in a manner that is most typical of the permanent premolar teeth of *Periptychus*. The paraconule, protocone, and metaconule and their cristae form a continuous arc (from mesial to distal end) on the shoulder. The interruption between the preprotocrista/preparaconule crista and parastylar region (which was observed on dP2) is substantially reduced on dP4. The hypocone and protostyle are present as small cusps on dP4, and are positioned lingual to the crescentic shoulder on the lingual slope of the protocone. The pre- and postcingulae are absent.

The dp5 premolar is completely molariform in its construction. The enlarged, centralised paracone seen on the preceding deciduous premolars is replaced by a paracone and metacone positioned on the buccal border of the tooth. The paracone is positioned slightly higher than the metacone, and is more buccally-expanded. In occlusal aspect, the tooth profile exhibits a distinct ectoflexus which is more pronounced than that seen on the permanent molars. The preparacrista descends the paracone mesially and intercepts the preparaconule crista and precingulum to form a vestigial parastyle. The metacone is situated very slightly lingual of the paracone and is less buccally expanded, despite occupying roughly the same surface area of the tooth. The postmetacrista descends the metacone distally to intercept the metacingulum, where it forms a small metastyle. The tooth exhibits a narrow but sharp stylar shelf which supports two stylar cusps (the parastyle [= stylar cusp A] and the metastyle [= stylar cusp E]).

The distinctive lingual crescentic shoulder that is characteristic of the permanent premolar dentition is lost on dP5 and replaced by a more molariform configuration. The talon of dP5 is lingually expanded in comparison to the preceding deciduous premolars, but is not as buccolingually or mesiodistally expanded as in the molars. The reduction of the talon causes dP5 to lack the quadrate occlusal profile, which is typical of the molar dentition. Instead it exhibits a subtriangular occlusal profile. The paraconule and metaconule are small, but well developed. The preparaconule crista and postmetaconule crista are well developed, but do not extend beyond the lingual border paracone and metacone, respectively. The protocone forms a small erect cusp that is positioned lower than both the paracone and metacone and only slightly higher than the conules. The apex of the protocone is shifted buccally and possesses an elongate lingual protocone slope. The pre- and postprotocristae are well defined and extend to the conules, creating a small trigon basin. A distinct protostyle is present on the mesiolingual flank of the protocone, possessing a single circular cusp on a well-developed precingulum. The cusp is roughly subequal in size to the conules. A large hypocone is present on the postcingulum on the buccodistal flank of the protocone. A second slightly smaller cusp is observed in AMNH 3794 on the postcingulum just buccal of the hypocone. The base of the hypocone is massively-expanded and extends lingually beyond the base of the protocone and protostyle, nearly enveloping the base of the protocone. The pre- and postcingulae are extremely well developed and form broad shelves which extend from the protostyle and hypocone to the parastyle and metastyle, respectively.

The eruption sequence of the postcanine dentition of *Periptychus* can be inferred based on: AMNH 3720, a relatively unworn left mandibular specimen preserving a partially erupted dp2, fully erupted dp4-5 and a partially erupted m1; AMNH 15937, a right mandibular fragment with dp2-5, a fully erupted m1 and a partially erupted m2; AMNH 3637, a right mandibular fragment with highly worn dp2-5 and fully erupted m1-3; and AMNH 3794, a palate preserving the right dP2-5, M1-2 and the left dP4-5, M1 with a partially erupted M2. We assume that the eruption sequence of the upper and lower dentition correspond to one another

The dental replacement in *Periptychus* follows the typical mammalian diphyodont condition in which antemolars are replaced by one generation, but molars are not replaced. We infer the following sequence from the dental specimens observed: dp4 + dp5 → dp2 → m1 → m2 → m3 → p2 + p4 + p5. From the specimens observed, it is not evident whether the sequential replacement of premolars in *Periptychus* occurs anteroposteriorly or posteroanteriorly or at what point p1/P1 erupts. Based on the wear on AMNH 3637, it is clear there is a substantial hiatus between the complete eruption of the molar series and the replacement of the deciduous premolars.

### Postcranial skeleton

We calculated the body mass of *Periptychus carinidens* using the scaling equation of Campione and Evans [73] based on minimum midshaft circumference measurements of the humerus and femur. We found the body mass of one particularly well preserved partial skeleton of *Periptychus carinidens*, NMMNH P-35194, to be 23.1kg. Further to this, we also estimated the body mass of *Ectoconus* (AMNH 16500) to be 99.8kg, *Arctocyon* (MNHM F. CR. 17, 72) to be 49.6kg and *Pantolambda* (AMNH 16663) to be 42.3kg.

#### Vertebral column

**Fig 17:**
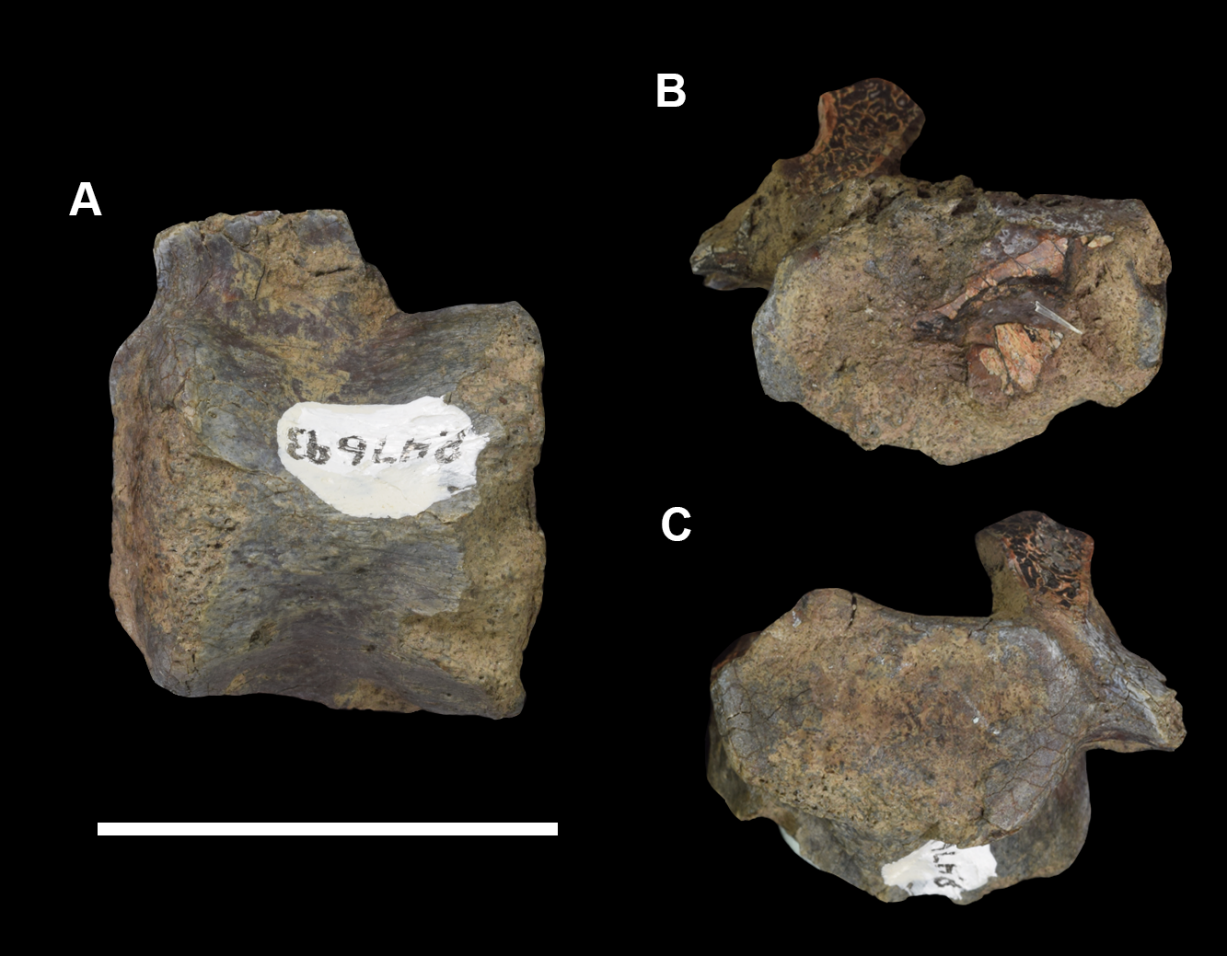
Lumbar vertebra of *Periptychus carinidens* (NMMNH P-47693). (A) dorsal view; (B) anterior view; (C) posterior view. Scale bar: 30mm.

The vertebral skeleton of *Periptychus* is poorly known, with very few associated or referred specimens. NMMNH P-47693 includes two partial vertebrae, which we interpret as lumbar vertebrae rather than proximal caudal vertebrae (our rationale is outlined below) (Fig 17). Comparisons are made to *Arctocyon primaevus* (MNHN.F.CR100 and CR101 figured in [52]) and *Pantolambda bathmodon* (AMNH 16663, figured in [5]).

Cope figured an atlas, axis (AMNH 3637) and four cervical vertebrae (unreferenced specimens in [4]) for *Periptychus*. However, these specimens were not available for observation during the period of study, as they could not be located in the AMNH collection. Cope described the cervical vertebrae of *Periptychus* as short, being dorsoventrally deeper than anteroposteriorly long and mediolaterally wider than dorsoventrally deep [4]. The anterior and posterior surfaces of the cervical body are described as ‘very slightly’ opisthocoelous (the posterior surface of the body is concave and the anterior surface is either flat or convex) [4]. Comparisons are made to *Arctocyon primaevus* (MNHN.F.CR100 and CR101 figured in [52]) and *Pantolambda bathmodon* (AMNH 16663, figured in [5]).

The body of the lumbar vertebra of *Periptychus* is robust and dorsoventrally compressed. The anterior and posterior ends are opisthocoelous. The transverse process is blade-like and protrudes anterolaterally; it does not appear to project ventrally. The anterolateral projection of the transverse process is indicative of a lumbar vertebra based on observations of *Arctocyon* and *Pantolambda*, rather than a caudal vertebra. In *Arctocyon* and *Pantolambda*, the transverse processes of the lumbar vertebrae project anterolaterally, whereas on the proximal caudals of these taxa the transverse processes project posterolaterally. The transverse process observed for *Periptychus* is not as robust as those observed in *Pantolambda*. In *Periptychus* (and also *Arctocyon*), the anteroposterior length of the base of the process is approximately half the anteroposterior length of the body, whereas in *Pantolambda* the base of the transverse process is over half as long as the anteroposterior length of the body. The vertebral foramen is present and moderately large in relation to the size of the vertebral body.

**Fig 18:**
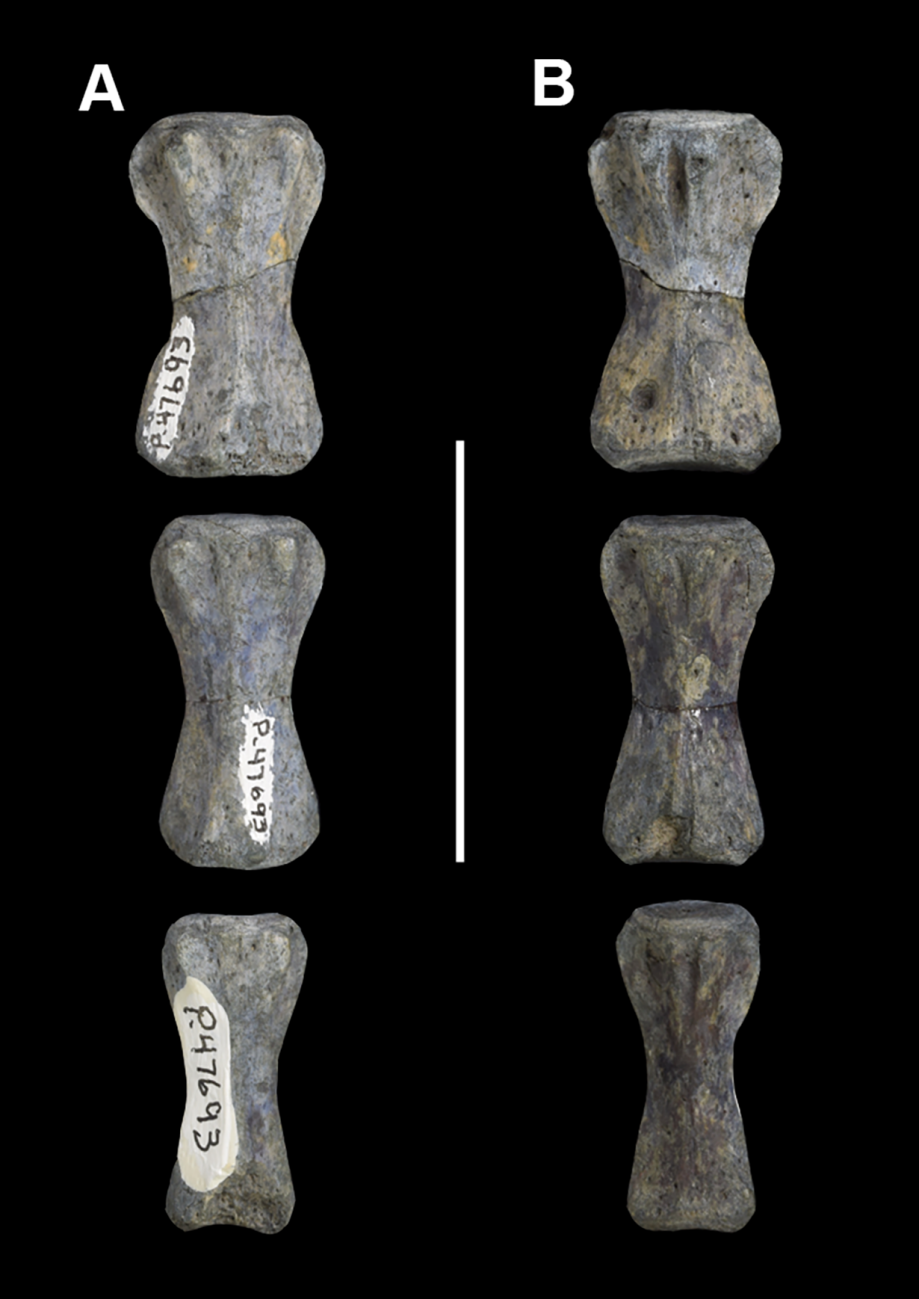
Caudal vertebrae of *Periptychus carinidens* (NMMNH P-47693). (A) dorsal view; (B) ventral view. Scale bar: 30mm.

NMMNH P-47693 also includes three consecutive distal caudal vertebrae of *Periptychus* (Fig 18). The caudal vertebrae known for *Periptychus* are robust, rod-like bones. The distal caudal vertebrae of *Periptychus* are broadly similar to *Arctocyon*, and more elongate that those known for *Pantolambda*. In *Periptychus*, the largest of the three distal caudals is subequal in anteroposterior length to the lumbar vertebrae described above, with the subsequent caudals showing a small decrease in size and robustness posteriorly. The transverse processes are elongate and subdivided into two parts, forming a pair of anterior processes and a pair of posterior processes, both of which project laterally from the vertebral body. The prezygapophyses are present on all three caudals of *Periptychus*, forming prominent, dorsally projecting spines. The caudal prezygapophyses of *Periptychus* are not as well developed as those of *Arctocyon*, but are considerably more developed than those of *Pantolambda*. The postzygapopysis are present on the three caudal vertebrae of *Periptychus*; they become progressively weaker posteriorly and highly reduced in comparison to the prezygapophyses. The anterior and posterior surfaces of the body are circular in profile, with no dorsoventral compression, and they are moderately opisthocoelous.

#### Scapula

**Fig 19:**
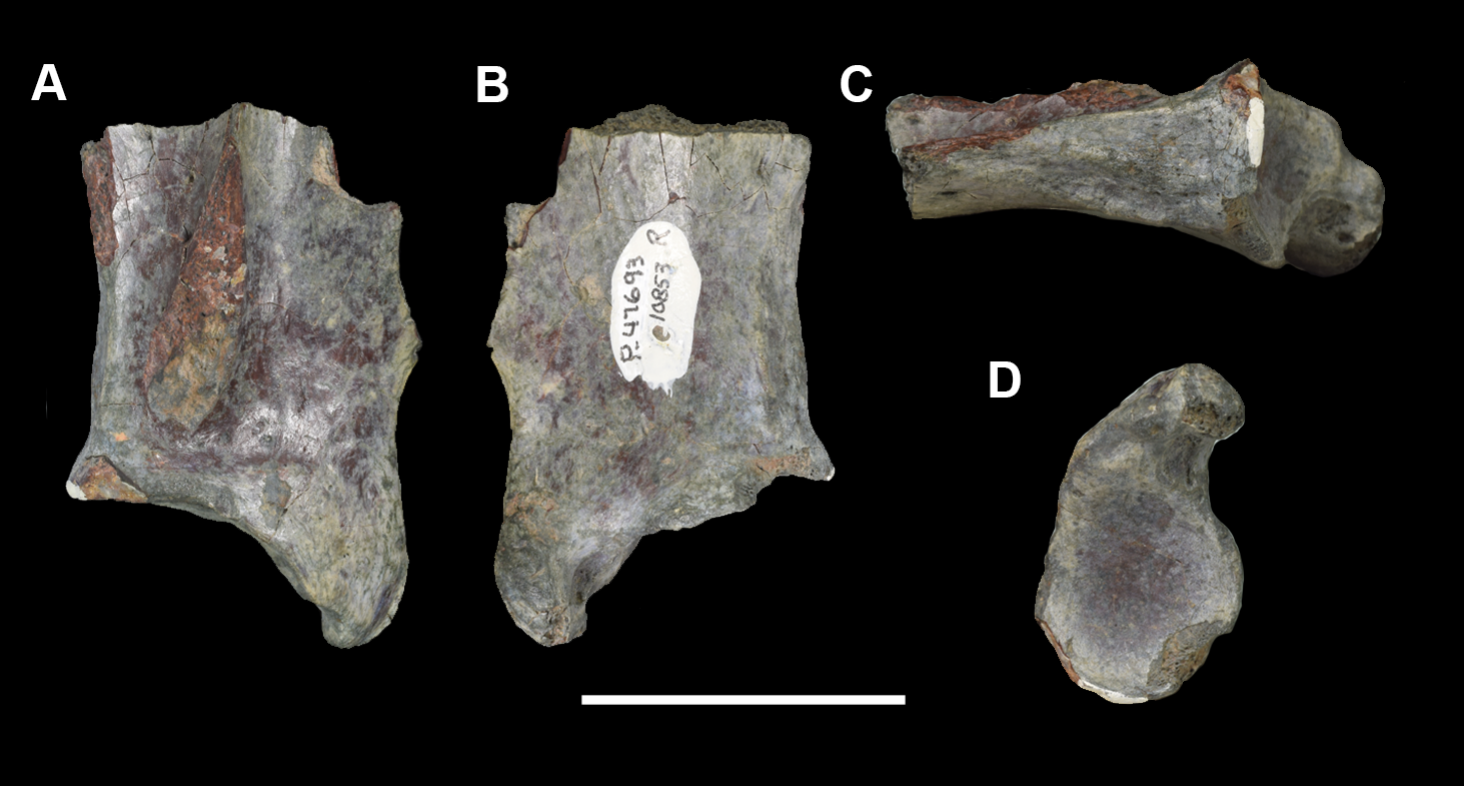
Right scapula of *Periptychus carinidens* (NMMNH P-47693). (A) lateral view; (B) medial view; (C) posterior view; (D) distal view. Scale bar: 30mm.

**Fig 20:**
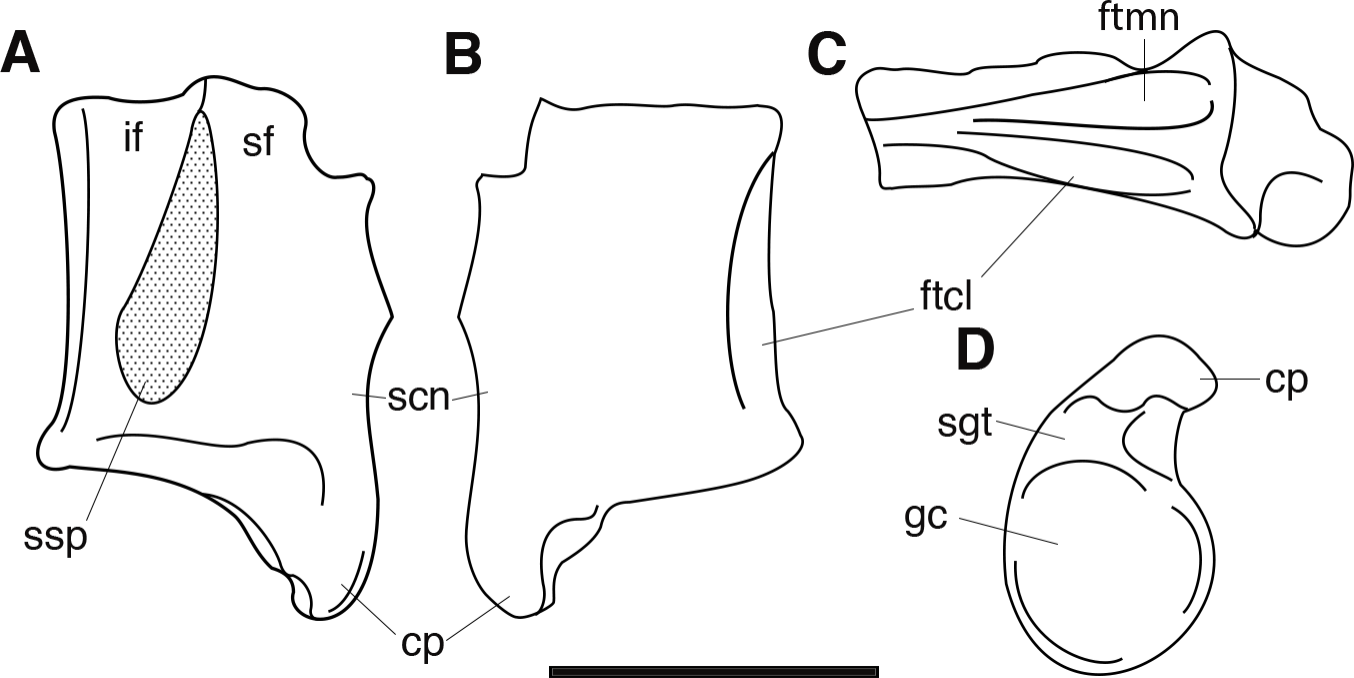
Annotated line drawing of the right scapula of *Periptychus carinidens* (NMMNH P-47693). (A) lateral view; (B) medial view; (C) posterior view; (D) distal view. Abbreviations: cp, coracoid process; ftcl, fossa for the origin of the caput longum of the triceps; ftmn, fossa for the origin of the teres minor; gc, glenoid cavity; if, infraspinatus fossa; scn, scapular notch; sf, supraspinatus fossa; sgt, supraglenoid tubercle; ssp, scapular spine (damaged). Scale bar: 30mm.

The scapula is poorly known for *Periptychus*, with no complete specimens recognised. The following description is based on NMMNH P-47693, which includes a partial scapula including the glenoid region (Figs 19 and 20), and AMNH 3636 (figured in [4]), which includes partial scapula including the glenoid region and scapula spine but not the lateral portion or acromion. Comparisons are made to *Ectoconus ditrigonus* (AMNH 16500, figured in [5]), *Mithrandir gillianus* (NMMNH P-3083, figured in [48]), *Arctocyon primaevus* (MNHN F.967, MNHN F.CR.15 figured in [52]).

The partial scapular body of AMNH 3636 is poorly preserved. The sections of the supraspinatus fossa and subscapular fossa that are present do not include any portions of the true margins of the bone, so it is not possible to comment on the shape of the scapular body or the relative sizes of the supraspinatus and infraspinatus fossae. However, based on the portion of neck known on NMMNH P-47693, it is possible to conclude that the supraspinatus fossa and infraspinatus fossa form a relatively flat plate, so that in distal aspect the proximal border of the infraspinatus fossa is in line with the anteroposterior long axis of the distal epiphysis. This contrasts with the supraspinatus and infraspinatus fossae of *Arctocyon*, in which the posteroproximal border of the infraspinatus fossa curves laterally so that in dorsal aspect the posterior edge of the infraspinatus fossa projects at approximately 30° from the anteroposterior long axis of the distal epiphysis.

The scapular spine on AMNH 3636 is damaged. Nevertheless, the base of the spine is preserved and extends the length of the scapula. It is completely straight, with no curvature along its length. Both *Ectoconus* and *Arctocyon* exhibit a slightly posteriorly directed curvature towards the midsection of the spine. The scapular spine of *Periptychus* terminates just short of the proximal and distal borders of the bone. The base of the spine is relatively thin (more so than in *Arctocyon*) but broadens at the distal end, suggesting a high placement of the acromion. However, we cannot comment on whether the acromion projects distally over the glenoid fossa, or on the presence or absence of any posterior inclination in the spine.

The scapular body joins the distal glenoid portion of the scapula via an anteroposteriorly broad neck. The neck appears to be proportionally broader than that of *Ectoconus* and more closely comparable to that of *Arctocyon*, but the damaged supraspinatus fossa makes direct comparison difficult. The scapular notch in *Periptychus* is shallow, and as such the anteroposterior length of the scapula neck is subequal in anteroposterior length to the distal epiphysis, but remains longer than the anteroposterior length of the glenoid fossa. *Arctocyon* possesses a similar shallow scapular notch to *Periptychus*. In contrast, in *Ectoconus*, the notch is near semi-circular and the scapular neck does not exceed the glenoid fossa in anteroposterior length. Two deep grooves are present on the posterior most edge of the scapula neck. The medial-most groove is interpreted as the origin of the triceps caput longum. The lateral-most groove is interpreted as the origin of the teres minor muscle (which likely continued proximally along the posterior border of the scapula). These muscle attachment areas are large and well developed, much more so than those in *Arctocyon*.

In *Periptychus*, the glenoid fossa is broad and pyriform in shape, with a shallow central concavity. In distal view, the long axis of the distal epiphysis (the glenoid portion) of the scapula is angled at 90° to the plane of the scapula body (formed by the supraspinatus and infraspinatus fossae). The medial margin of the glenoid is slightly expanded relative to the lateral margin, creating an asymmetrical distal profile. The overall shape of the glenoid fossa of *Periptychus* is near identical to that in *Ectoconus*, just smaller in size; in both taxa, the glenoid is broader and more robust in comparison to *Mithrandir gillianus*. The glenoid fossa of *Periptychus* is substantially more anteroposteriorly elongate than that of *Arctocyon*, in which the glenoid is almost circular in profile. In lateral view, the glenoid of *Periptychus* is concave and is very similar in outline to both *Ectoconus* and *Arctocyon*. The lateral profile of the glenoid of *Mithrandir* is more anteroposteriorly elongate and concave than in *Periptychus*, but not as mediolaterally broad.

In *Periptychus*, the supraglenoid tubercle is a smooth tuberosity located on the anterolateral edge of the glenoid fossa. It provides a mediolaterally broad and slightly raised area of attachment for the origin of the long head of the biceps brachii muscle. The tuberosity is distinct from the glenoid fossa and interrupts the curvature of the rim of the glenoid fossa, where it borders the coracoid process. In *Ectoconus*, the supraglenoid tubercle is less salient and near continuous with the glenoid fossa posteriorly. In contrast, in *Arctocyon*, the tuberosity is more prominent relative to the glenoid fossa, but is positioned further within the perimeter of the glenoid fossa.

The coracoid process of *Periptychus* projects medially and anterodistally, with a well-developed, thick, hook shaped process which serves to stabilise the shoulder joint. The coracoid process of *Periptychus* is robust, but not as robust as the morphology exhibited by *Ectoconus* and *Arctocyon* (particularly when viewed in lateral/medial aspect). However, the coracoid of *Periptychus* is still comparatively stout (and very different in shape) in comparison to the elongate coracoid of *Mithrandir*. The head of the coracoid of *Mithrandir* is curved medially but does not form the distinctive hook shape seen in *Periptychus*, *Ectoconus* and *Arctocyon*. In *Periptychus*, the medial surface of the neck of the coracoid process exhibits a distinct ovoid fossa positioned adjacent to the supraglenoid tubercle for the insertion of the pectoralis minor muscle. In *Periptychus*, the anterodistal apex of the coracoid process is marked by a small ridge and associated sulcus for the origin of the coracobrachialis muscle and the short head of the biceps brachii muscle.

The clavicle is unknown for *Periptychus* and the lack of a known acromion process impedes any possible inferences about the nature of articulation between the scapula and clavicle. A clavicle is known for *Carsioptychus* (AMNH 27601) and *Ectoconus* (AMNH 16500) suggesting it was indeed present in *Periptychus* and not lost as in more derived ungulates.

#### Humerus

**Fig 21:**
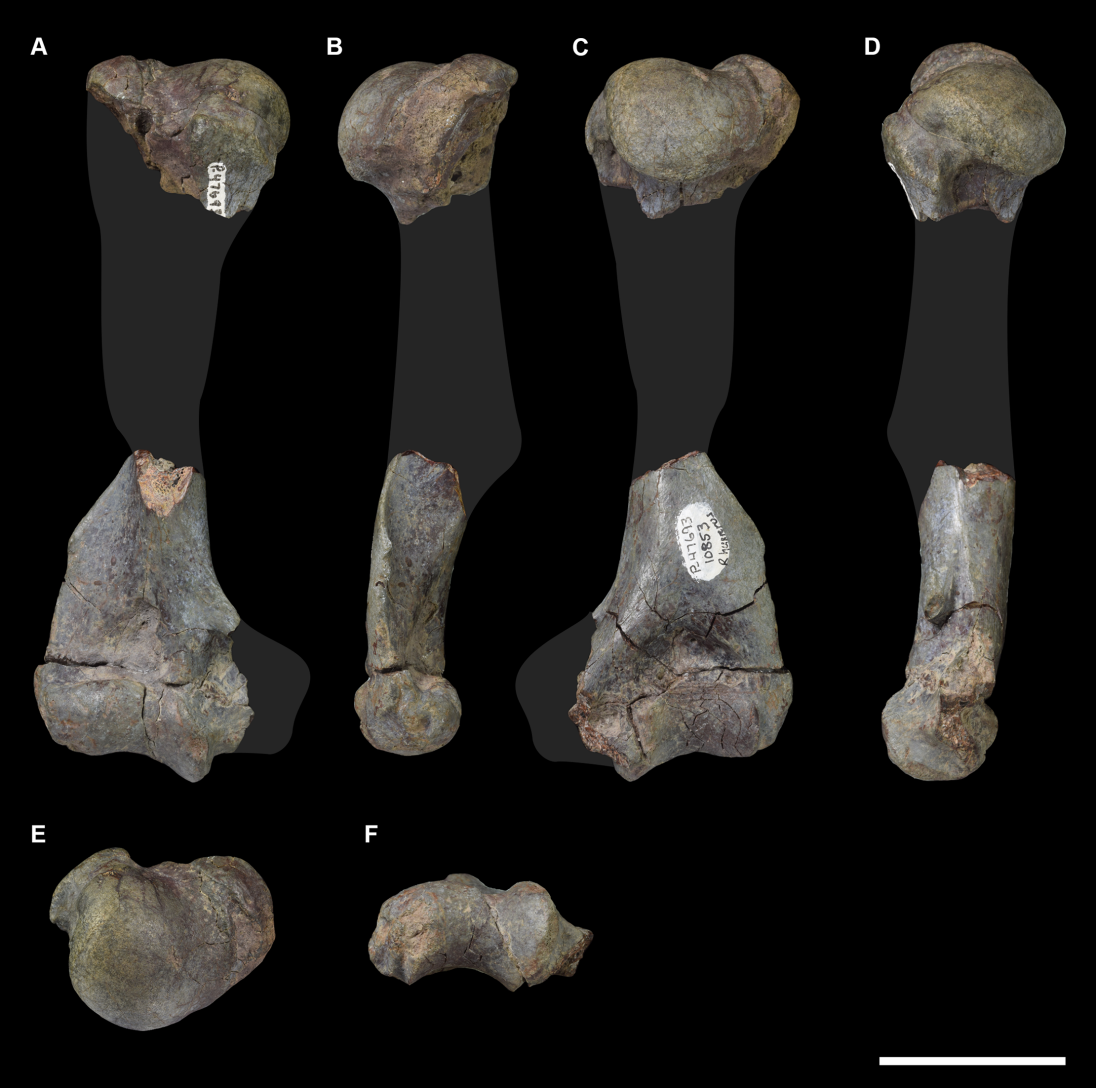
Right humerus of *Periptychus carinidens* (NMMNH P-47693). (A) anterior view; (B) lateral view; (C) posterior view; (D) medial view; (E) proximal view, (F) distal view. Scale bar: 30mm.

**Fig 22:**
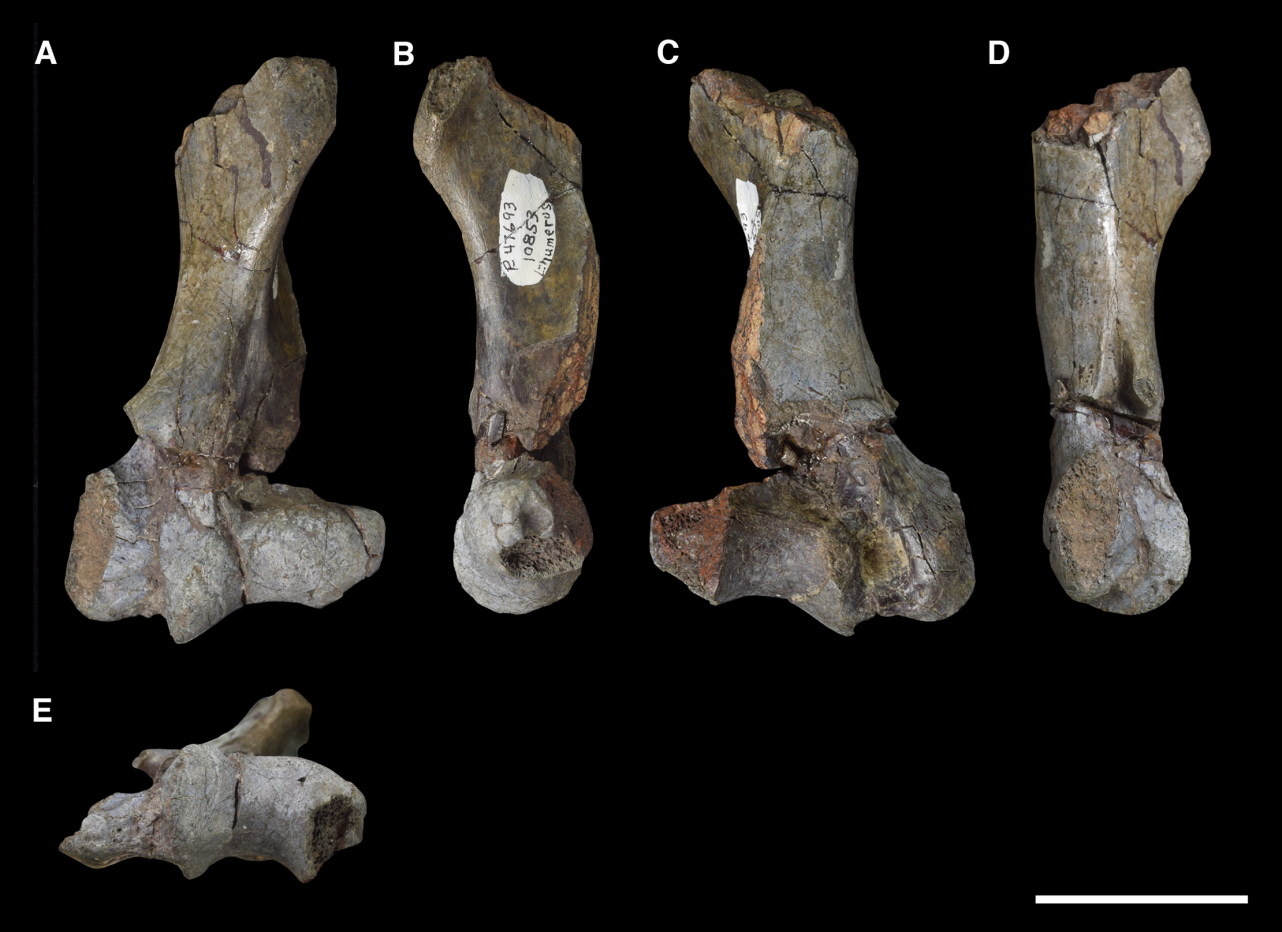
Left humerus of *Periptychus carinidens* (NMMNH P-47693). (A) anterior view; (B) lateral view; (C) posterior view; (D) medial view; (E) distal view. Scale bar: 30mm.

The humerus of *Periptychus* is known from numerous specimens. This description is based on an assortment of specimens, including several complete humeri that allow for a comprehensive description of the anatomy. NMMNH P-35194 includes the diaphysis of the right humerus and the distal portion of the left humerus. NMMNH P-47693 includes the proximal and distal portions of the right humerus and the distal portion of the left humerus (Figs 21 and 22). NMMNH P-53998 includes an incomplete right humerus. AMNH 3636 includes a complete right humerus (figured in [5]), AMNH 17075 includes a complete right humerus (figured in [5]), and AMNH 3637 includes a partially complete left humerus.

**Fig 23:**
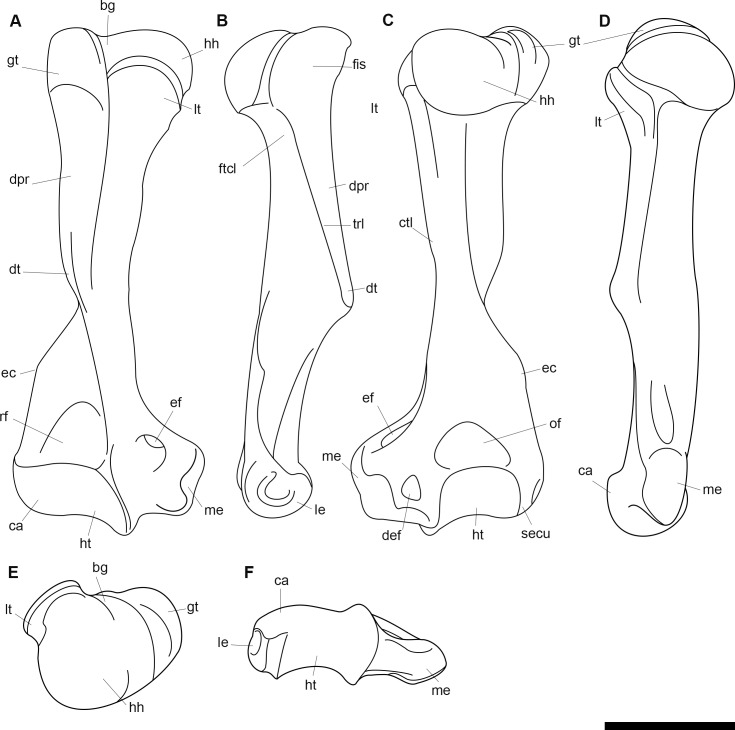
Annotated line drawing of the right humerus of *Periptychus carinidens*. (A) anterior view; (B) lateral view; (C) posterior view; (D) medial view; (E) proximal view; (F) distal view. Abbreviations: bg, bicipital groove; ca, capitulum; ctl, crest for insertion of the teres major and latissimus dorsi; def, dorsoepitrochelar fossa; dt, deltoid tuberosity; dpr, deltopectoral region; ec, epicondylar crest; ef, entepicondylar foramen; ftcl, fossa for the caput longum of the triceps brachii; fis, fossa for insertion of the infraspinatus; gt, greater tubercle; hh, humeral head; ht, humeral trochlea; le, lateral epicondyle; lt, lesser tubercle; me medial epicondyle; of, olecranon fossa; rf, radial fossa; secu, sulcus for the extensor carpi ulnaris; trl, tricipital line. Scale bar: 30mm.

Comparisons are made to *Ectoconus ditrigonus* (AMNH 16500, figured in [5]), *Mithrandir gillianus* (NMMNH P-3083, figured in [48]), *Pantolambda bathmodon* (AMNH 16663, figured in [5]), *Claenodon ferox* (AMNH 16542) and *Arctocyon primaevus* (MNHN.F.CR17 & CR16, figured in [52]). Comparisons are also drawn to a referred distal humerus of *Protungulatum* sp. (AMNH 118460, figured in [100]).The overall morphology of the humerus of *Periptychus* is that of a robust, stout limbed animal (Fig 23). In specimens preserving the entire humerus, the bone is short, with a relatively straight diaphysis and broad epiphyses that do not exhibit any torsion. The proximal epiphysis is sub-spherical, and is mediolaterally wider than anteroposterior deep.

The greater tubercle is large but remains subequal to slightly shorter than the humeral head. *Mithrandir* and *Pantolambda* display a similar condition, with both taxa possessing a well-developed greater tubercle, but one that does not surpass the humeral head in height. In contrast, *Arctocyon* and *Claenodon* possess a massive greater tubercle that projects proximally beyond the humeral head. *Ectoconus* exhibits an intermediate state between *Periptychus*, and *Arctocyon* and *Claenodon* in which the greater tubercle is positioned just proximal of the humeral head, but not as proximal as in *Arctocyon* and *Claenodon*. In proximal view, the greater tubercle of *Periptychus* is positioned on the anterolateral surface of the humeral head, forming a crescentic tuberosity that is tightly appressed against the sub-hemispherical dome of the head. This is notably different from *Arctocyon*, in which the greater tubercle, although still appressed, is positioned further towards the anterior surface of the humeral head. Although the greater tubercle of *Periptychus* is present as a structure that is set off from the head of the humerus, it is not nearly so well defined as the condition in *Mithrandir*. In *Mithrandir*, the sulci that separate both the greater and lesser tuberosities from the head are proportionately much deeper than the shallow grooves exhibited on the proximal epiphysis of *Periptychus*. The lateral surface of the greater tubercle of *Periptychus* possesses a well-defined fossa for the insertion of the infraspinatus muscle. In *Pantolambda bathmodon* this muscle scar is orientated towards the posterolateral axis.

The proximal surface of the greater tubercle in *Periptychus* is separated from the head of the humerus by a broad, shallow sulcus that is a continuation of the bicipital groove. The bicipital groove extends onto the anterior surface of the proximal epiphysis at an oblique angle, where it becomes somewhat deeper and served to channel the tendons of the pectoralis major, the long tendon of the biceps brachii and the teres minor. The lateral ridge of the bicipital groove is proximally defined by the medial boundary of the greater tubercle, which descends distally to form a strong tricipital line. This also demarcates the medial boundary of the deltopectoral crest. The bicipital groove of *Periptychus* is not overhung by the greater tubercle. The tricipital line of *Periptychus* is well expressed and provided an insertion site for the teres minor and latissimus dorsi muscles approximately half way down the proximodistal length of the deltopectoral region.

In *Periptychus*, the lesser tubercle is small and appressed against the anteromedial surface of the humeral head, barely reaching the neck of the humeral head in height. On its posterior surface, it possesses a deep scar for the insertion of the subscapularis muscle. The lesser tubercle of *Mithrandir gillianus* is comparatively more prominent than that in *Periptychus*. In contrast, in *Ectoconus*, the lesser tubercle appears to be proportionally reduced in relation to the humeral head.

Distal to the lesser tubercle in *Periptychus*, a small, poorly defined ridge marks the insertion site of the teres major and latissimus dorsi muscles. This feature is weakly present in *Periptychus*, but noticeably more salient in *Arctocyon* and *Claenodon*. In *Pantolambda*, it is particularly well developed, to form a small tuberosity which is delimited by a small, shallow sulcus on the posterior surface. This feature does not appear to be particularly well developed in *Ectoconus*, despite the considerable robusticity of the taxon.

The humeral head of *Periptychus* is sub-hemispherical in shape and orientated proximoposteriorly. It does not project strongly posteriorly and has a short humeral neck which overhangs the diaphysis by approximately 10% of the overall anteroposterior depth of the humeral head. In contrast, the humeral heads of *Arctocyon*, *Claenodon* and *Pantolambda* all overhang the diaphysis by up to approximately 35%. Overall, the humeral head of *Periptychus* is broadly comparable to that of *Pantolambda* in terms of shape, although *Periptychus* possesses a shorter neck. The head of *Periptychus* is not as rounded as in *Mithrandir*, but is more rounded than the humeral head of *Ectoconus*. Overall, the tubercles of the proximal epiphysis of *Periptychus*, although not particularly salient in comparison to *Arctocyon* and *Claenodon*, are better defined than those seen in other taxa of a similar body size and suggested mode of life, namely *Ectoconus* and *Pantolambda*.

The humeral diaphysis of *Periptychus* is short and straight with several crests and flanges along its length. The shaft proper is subcircular in cross-section and not particularly robust; it is the addition of the crests and their associated flanges that give the humerus its apparent robusticity. Despite the considerable similarities in morphology between the two taxa, the diaphysis of *Ectoconus* is considerably more robust and somewhat anteroposteriorly flattened compared to *Periptychus*.

On the proximal portion of the diaphysis, the tricipital crest and deltopectoral crest demarcate an enlarged ‘V’-shaped deltopectoral region. This region is well expressed, although it is not as prominent or elevated relative to the shaft as in *Arctocyon* and *Pantolambda*. *Arctocyon* and *Pantolambda* both possess extremely well-developed, but distinctly different, deltopectoral regions. The deltopectoral crest and tricipital line of *Periptychus* descends approximately 50% of the shaft length to terminate in a small and low deltoid tuberosity. The surface of the deltopectoral region is formed from a single smooth, anteriorly facing shelf of bone. The edges of the deltopectoral crest are well defined, although they do not form a distinct overhanging lip as seen in *Arctocyon*. *Arctocyon* is further characterised (and distinguished from *Claenodon*) by the presence of a third crest which runs along the proximodistal midline of the deltopectoral region, dividing the shelf into two distinctly separate muscle attachment areas (see [52], Fig 7A). In posterior view, the deltopectoral region of *Periptychus* forms a moderately well-developed, laterally projecting flange. The deltopectoral crest of *Periptychus* is not as salient as that observed in *Ectoconus* and *Pantolambda*. In *Pantolambda*, the crest and tricipital line are positioned high above the bone shaft, along with an enlarged deltoid tuberosity, which forms a thick laterally projecting flange. The flange of *Periptychus* extends laterally, approximately half the mediolateral width of the lateral epicondyle. However, in *Pantolambda* and *Ectoconus*, the flange projects laterally to the same extent as the lateral epicondyle.

In *Periptychus*, the lateral epicondylar crest descends the distal portion (approximately 40%) of the humeral diaphysis to the lateral epicondyle, and provided attachment for the anconeus and the extensor carpi radialis muscles. Proximally it extends just past the horizontal level of the deltoid tuberosity. Laterally, it does not expand beyond the tangential lateral boundary set by the capitulum in any of the observed specimens. With that said, however, the trajectory of the epicondylar crest is variable among specimens. In AMNH 3636, it forms a straight line between the diaphysis and capitulum, whereas in a smaller specimen, NMMNH P-47693, it ascends proximally from the capitulum for half its length before inflecting and traversing the space to the diaphysis at a proximomedial gradient. This is similar to the condition in *Arctocyon*, although Argot [52] notes variation within this taxon as well. In *Periptychus*, the inflection of the crest creates a small tuberosity at the vertex on the crest. The lateral epicondylar crest of *Periptychus* (as well as *Arctocyon*, *Claenodon* and *Protungulatum*) is slightly convex, so that the crest flares anteriorly. In *Pantolambda* the lateral epicondyle is so extremely convex that the epicondylar crest projects posterolaterally. *Ectoconus* exhibits an intermediate condition between *Periptychus* and *Pantolambda*, with the epicondyle crest projecting somewhat posterolaterally proximally before curving anteriorly towards the distal epiphysis.

In *Periptychus*, the distal epiphysis of the humerus is mediolaterally expanded and anteroposteriorly flattened so that it is approximately 30% broader than the proximal epiphysis. The medial epicondyle is massive. It accounts for almost 50% of the mediolateral width of the distal epiphysis and forms a robust rectangular shaped tuberosity (in anterior aspect) that projects medially beyond the tangential medial boundary set by the proximal epiphysis. The medial epicondyle of *Periptychus* is very similar in form and overall proportions to those of *Ectoconus* and *Pantolambda*. It is more robust and projects to a higher proximomedial height than in *Arctocyon*, but it is not as proportionately robust as the medial epicondyles of *Protungulatum* or *Mithrandir*, which are expanded to a higher proximomedial level than in any of the other larger-bodied taxa observed.

The medial apex of the medial epicondyle of *Periptychus* is broadly rounded, with a well-defined muscle scar that extends onto the anterior surface of the condyle to provide an attachment site for the origin of the flexor carpi radialis muscle. *Claenodon* also exhibits this scar, but it is proportionally smaller and positioned lower on the medial epicondyle. None of the other comparison taxa exhibit this feature, although in *Pantolambda* this could be due to damage.

The proximal border of the medial epicondyle of *Periptychus* is marked by a large entepicondylar foramen. The foramen is mediolaterally wide, with a robust entepicondylar crest. However, it is small relative to the size of the medial epicondyle, as it is less than 50% of the mediolateral width of the epicondyle. A proportionally similar foramen is present in *Ectoconus*. This is small in comparison to *Protungulatum*, *Arctocyon* and *Claenodon*, in which the foramen is much larger and extends over 50% of the mediolateral width of length of the medial epicondyle. However, the entepicondylar foramen of *Periptychus* is proportionally much larger than that in *Pantolambda*, where the foramen is only 25% of the mediolateral length of the medial epicondyle. In anterior aspect, the entepicondylar foramen of *Periptychus* (and *Ectoconus*) opens onto the medial epicondyle at a 45° anteriorly projecting angle from the distolateral axis, whereas in *Protungulatum* it opens up closer to the vertical distolateral axis, and in *Arctocyon*, *Claenodon* and *Pantolambda* it opens up closer to the distal axis.

The posterior surface of the medial epicondyle of *Periptychus* exhibits a deep, subtriangular notch positioned adjacent to the medial wall of the humeral trochlea, for origin of the ulnar collateral ligament. This feature is well developed in *Ectoconus* and *Pantolambda*, in which it parallels the morphology of *Periptychus*. It is less developed in *Mithrandir* and *Protungulatum*, in which the fossa is present, but much shallower. It is also present in *Arctocyon*, but as shallow and elongate depression rather than a distinct ovoid fossa.

The radial fossa of *Periptychus* is relatively shallow, with gently sloping walls that form a triangular border. Relative to the size of the distal epiphysis, it is larger than the radial fossa of *Ectoconus* and *Arctocyon*, although not as deep as in *Arctocyon*. The olecranon fossa of *Periptychus* is deeper than the radial fossa with steep, near vertical walls. It is proportionally larger and deeper than in *Ectoconus* but not as deep as in *Arctocyon*. The mediolateral width of the fossa is equal to the mediolateral width of the humeral trochlea. This contrasts with the condition exhibited by *Protungulatum* and *Mithrandir*, where the mediolateral width of the humeral trochlea is narrower than the olecranon fossa. There is no separate coronoid fossa and the humerus does not possess a supratrochlear foramen like that seen in *Protungulatum*.

The lateral epicondyle of *Periptychus* is considerably smaller than the medial epicondyle, with no lateral expansion beyond the capitulum. The posterior surface of the lateral epicondyle exhibits a deep sulcus positioned adjacent to the lateral wall of the humeral trochlea. This spool shaped sulcus is the posterior continuation of the capitulum for the origin of the extensor carpi ulnaris. The sulcus is well developed in *Ectoconus*, *Arctocyon* and *Pantolambda*, where it is like the morphology of *Periptychus*, and to a lesser extent in *Mithrandir* and *Protungulatum*, where it is present, but much shallower.

The lateral apex of the lateral epicondyle is flattened and exhibits three muscle scars. Two shallow sulci positioned on the posterior portion of the lateral apex are for the origin of the digit flexors. A third deeper, ovoid fossa positioned on the anterior border of the lateral apex is for the origin of the supinator muscle.

In anterior aspect, the humeral trochlea and ovoid capitulum form a near continuous articular surface. In *Periptychus*, the articular surface accounts for 56% of the mediolateral width of the distal epiphysis. This is broader than in *Ectoconus* (51%) and *Protungulatum* (53%), comparable to *Pantolambda* (55%) and smaller than *Arctocyon* (61%) and *Claenodon* (59%). There is no clear development of the zona conoidea in *Periptychus*. The lack of a definitive zona conoidea makes distinguishing the boundary between the capitulum and trochlea difficult. When considering the articulation between the humerus, ulna and radius, the capitulum of *Periptychus* is mediolaterally broader than the trochlea, accounting for 34% of the mediolateral width of the distal epiphysis. This condition is prevalent in all the comparison taxa observed, with the capitular region accounting for somewhere between 30–35% of the mediolateral width of the distal epiphysis.

The humeral trochlea of *Periptychus* is asymmetrical. The medial keel of the trochlea is strongly flared and projects mediodistally beyond the horizontal distal boundary set by the capitulum and the medial epicondyle. This condition is seen in all the comparison taxa, but in varying degrees of development. In *Protungulatum*, the medial trochlea keel is somewhat distally expanded but barely projects beyond the distal boundary of the medial epicondyle. The medial keel of *Mithrandir* is more developed than in *Protungulatum*, but does not protrude to the same level as in *Periptychus* (or any of the other remaining comparison taxa). In *Pantolambda*, the medial trochlea wall is both mediolaterally and distally expanded so that it looks more robust and does not give the appearance of flaring as far medially as in *Periptychus*. The medial keel of *Ectoconus* is subequal to that of *Periptychus* in terms of mediodistal protrusion, but it is not as mediolaterally robust. In *Arctocyon* and *Claenodon*, the medial trochlea keel is the most developed of any of the taxa observed, projecting farther distally relative to the capitulum and medial epicondyle. In *Periptychus* the anterior articular surface of the trochlea is not as mediolaterally wide as the posterior trochlea surface, and it is displaced medially relative to the position of the posterior articular surface due to displacement by the capitulum.

In posterior aspect, the trochlea of *Periptychus* exhibits sharp, asymmetrical walls that define a moderately deep articular surface. As described above, the medial keel flares mediodistally and protrudes distally beyond the horizontal distal boundary of the medial epicondyle and capitulum. The lateral rim is reduced relative to the medial rim, so that it does not project distally beyond the horizontal distal level of the capitulum and medial epicondyle. The lateral keel is orientated at an oblique angle to the long axis of the humerus, such that it is approximately parallel to the medial trochlea keel (this is also the condition in *Protungulatum* and *Ectoconus*). In *Arctocyon* and *Claenodon*, the lateral keel is more prominent and is orientated parallel to the vertical long axis of the humerus. This is in stark contrast to the condition exhibited by *Mithrandir*, in which the lateral keel of the trochlea is almost symmetrical to the medial keel, so that in posterior aspect the trochlea has an isosceles trapezium shaped profile.

The capitulum of *Periptychus* forms an elongate ovoid articular surface in anterior view. It is more elongate in shape than the more convex capitulum of *Mithrandir* and *Protungulatum*. Laterally, the capitulum of *Periptychus* constricts and tapers continuously to form a short and narrow capitular tail. The constriction of the capitulum in *Periptychus* is less pronounced than in *Arctocyon* and *Pantolambda*, in which a ridge is present towards the lateral edge of the capitulum where the bone starts to taper. The capitular tail of *Periptychus* (and *Ectoconus*) is reasonably well defined, but not as distinguished as that of *Protungulatum* or *Arctocyon*, nor as broad as that of *Pantolambda*. The border between the proximal boundary of the capitulum and the distal border of the radial fossa is demarcated by a well-defined, mediolaterally aligned sulcus that extends along the length of the distal border of the radial fossa.

#### Ulna

**Fig 24:**
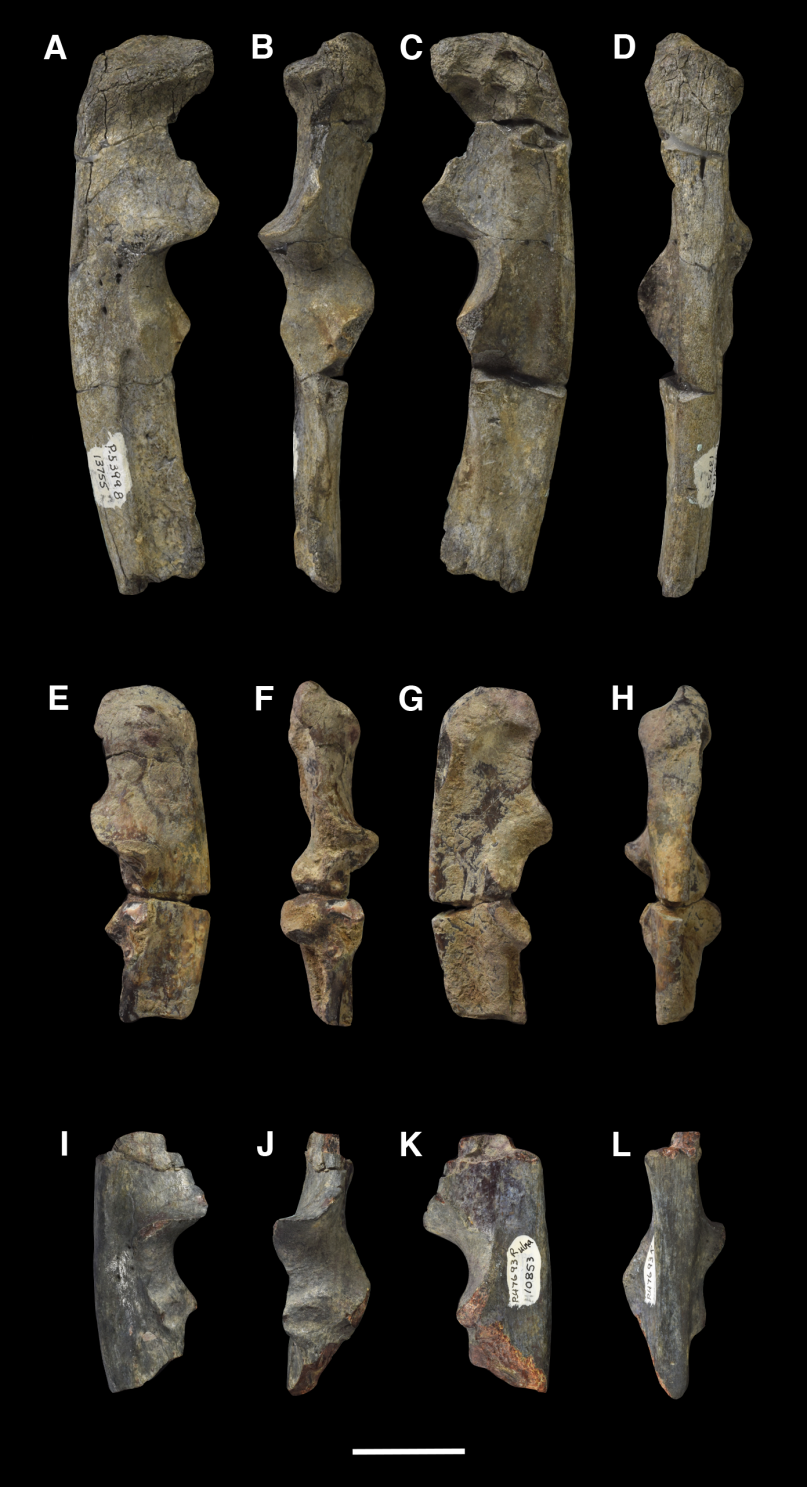
Ulnae of *Periptychus carinidens*. A-D: Right ulna (NMMNH P-53998): (A) lateral view; (B) anterior view; (C) medial view; (D) posterior view. E-H: Left ulna (NMMNH P35194): (E) lateral view; (F) anterior view; (G) medial view; (H) posterior view. I-L: Right ulna (NMMNH P-47693): (I) lateral view; (J) anterior view; (K) medial view; (L) posterior view. Scale bar: 30mm.

**Fig 25:**
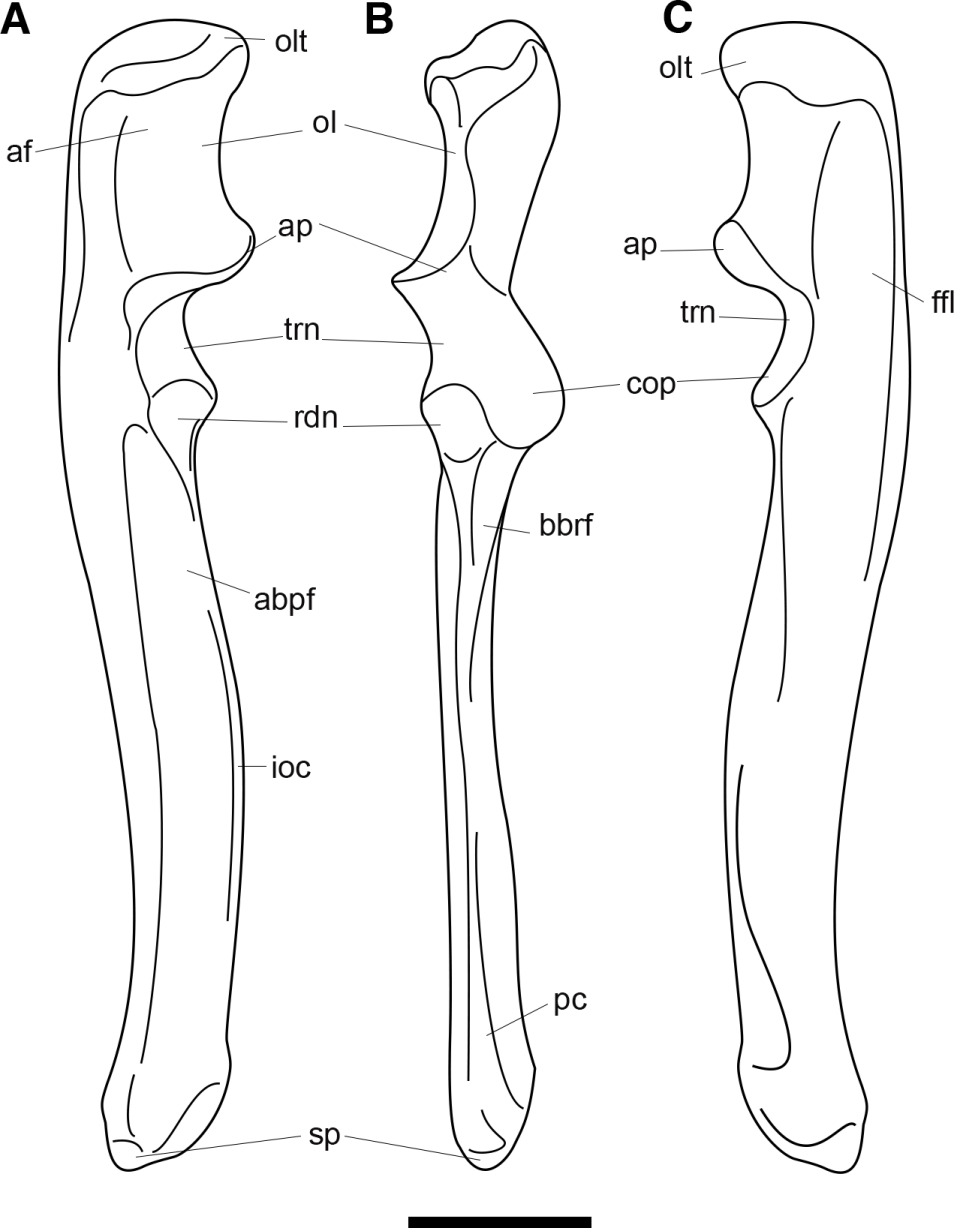
Annotated line drawing of the right ulna of *Periptychus carinidens*. (A) lateral view; (B) anterior view; (C) medial view. Abbreviations abpf, abductor pollicis longus fossa; af, anconeal fossa; ap, anconeal process; bbrf, biceps and brachialis fossa; cop, coracoid process; ffl, flexor fossa; ioc, interosseous crest; ol, olecranon; olt, olecranon tuber; pc, pronator quadratus + teres crest; rdn, radial notch; sp, styloid process; trn, trochlear notch. Scale bar: 30mm.

The following description is based on a selection of partial specimens that together allow for a complete description of the anatomy of the ulna (Figs 24 and 25). NMMNH P-35194 includes a partial left ulna (Fig 24E–24H). The specimen preserves the proximal epiphysis, including the articular region, and a small portion of the proximal diaphysis. The bone itself is well preserved, but somewhat concreted. NMMNH P-53998 includes the proximal portion of the right ulna (Fig 24A–24D). The specimen preserves the proximal epiphysis, including the articular region, and partial diaphysis, but the bone surface is weathered in areas and the olecranon process and the trochlear notch exhibit some damage. NMMNH P-47693 includes the proximal portion of the right ulna (Fig 24I–24L). The specimen preserves the articular region of the proximal epiphysis, but not the entire olecranon. AMNH 17075 includes a near complete left ulna (figured in [5]). AMNH 3636 includes the left and right ulnae. Comparisons are made to *Ectoconus ditrigonus* (AMNH 16500, figured in [5]), *Arctocyon primaevus* (MNHN.F.CR18, CR19 figured in [52]) and *Pantolambda bathmodon* (AMNH 16663, figured in [5])

The ulna of *Periptychus* is conspicuously gracile in comparison to the humerus. This observation is corroborated when taking into consideration the forelimb anatomy of other similarly sized Paleocene taxa such as *Ectoconus*, *Pantolambda* and *Arctocyon*, all of which possess distinctly more robust ulnae. The ulna of *Periptychus* is elongate, approximately 10% longer than the humerus, and mediolaterally compressed in anterior view. In lateral aspect, complete and near complete specimens show the diaphysis to exhibit a slight posterior bowing on the posterior border approximately level to the articular region. The distal epiphysis is also somewhat posteriorly directed, giving the ulna a subtle sigmoidal profile in lateral view. This is exaggerated by the slight anterior bow of the proximal portion of the ulna (olecranon and articular region). The curvature of the ulna is not as extreme as the curvature in *Arctocyon*, but more curved than the lateral profile of the ulna of *Pantolambda*, which is near perfectly straight. In anterior aspect, the ulna of *Periptychus* is somewhat laterally bowed along the central portion of the diaphysis, distal to the articular region.

The olecranon of *Periptychus* is massive, forming a robust sub-rectangular eminence on the proximal end of the ulna. It is approximately 10% proximodistally shorter than the articular region and forms 20% of the overall ulna length. *Pantolambda* and *Arctocyon* display a similar condition, whereby the olecranon is approximately 20% and 10% shorter than the articular region, respectively, and constitutes 20% of the overall ulna length in both taxa. This is unlike *Ectoconus*, in which the olecranon is 20% proximodistally longer than the articular region and constitutes 25% of the overall ulna length. In lateral aspect, the olecranon forms the mediolaterally deepest portion of the ulna, with the posterior border curving continuously onto the proximal border rather than forming an obtuse angle as seen in *Arctocyon*, or a near right angle as in *Ectoconus* and *Pantolambda*. In anterior aspect, the olecranon of *Periptychus* is medially excavated, but it is not curved medially as in *Arctocyon*. The proximal-most portion of the olecranon is flattened to form a mediolaterally broad tuber with a rugose surface and numerous muscle attachment fossae and scars.

In proximal aspect, a broad sulcus is positioned on the lateral side of the proximal surface of the tuber of the olecranon. The sulcus is formed from two interconnected shallow fossae for the insertion of part of the medial head of the triceps brachii (anteriorly) and the long head of the triceps brachii (posteriorly), with the lateral head of the triceps brachii attaching to the lateral rim of the olecranon tuber. In *Ectoconus*, the attachment area for the triceps brachii forms a large concave oval facet, suggesting the insertion of a much larger muscle body. The lateral surface of the olecranon process of *Periptychus* exhibits a large fossa proximally bordered by a steep ridge formed by the olecranon tuber. The posterior border of the fossae is demarcated by a salient ridge, which extends distally towards the articular region of the ulna. This anterior-most portion of the fossa is interpreted as the insertion site for the anconeus muscle, with the lateral flexor digitorum profundis originating in the posterior portion of the fossa and the lateral head of the triceps brachii inserting on the longitudinal ridge on the posterior border of the lateral surface of the olecranon process. In *Arctocyon* and *Ectoconus*, the fossa is not as well expressed and lacks a distinctive subdividing ridge. In *Pantolambda*, the posterior border of the fossa is well defined and a shallow subdividing ridge is present, but the overall morphology is not as pronounced as in *Periptychus*.

The medial surface of the olecranon process of *Periptychus* is excavated for the attachment of several muscle bodies. The proximal edge of the medial surface is raised and provides attachment for the medial head of the triceps brachii anteroproximally and the tensor fasciae antebrachii posteroproximally. A large, deep fossa excavates the body of the medial surface of the olecranon process, which is where the flexor carpi ulnaris muscle would have originated. A large longitudinal sulcus extends distally along the posterior border of the olecranon process and provided a large attachment area for the origin and passage of the medial flexor digitorum profundus muscle along approximately 50% of the ulna diaphysis. The medial surface of the olecranon of *Periptychus* exhibits a more deeply excavated morphology that that in *Pantolambda*. The morphology observed in *Periptychus* is somewhat more comparable to *Arctocyon*. However, in *Arctocyon*, the fossa for the origin of the flexor carpi ulnaris is relatively reduced in comparison to *Periptychus*, whereas the longitudinal groove for the medial flexor digitorum profundus muscle in *Arctocyon* is relatively more developed, forming a deeper and anteroposteriorly broader groove along the medial surface of the ulna.

The articular region of the ulna of *Periptychus* is prominent, forming a mediolaterally broad joint surface for the humerus and radius. In medial aspect, the proximal boundary of the trochlear notch is confined by a well-developed anconeal process, whilst the distal boundary is widely open due to the size and positioning of the coronoid process. In anterior aspect, the lateral border of the trochlea forms a shallow ‘w’-shaped profile where the radial notch protrudes. The surface of the trochlear notch is saddle shaped, with a mediolateral convexity and a subtle crest defining the proximodistal midline of the trochlea. This morphology is developed in *Periptychus*, but much more defined in *Pantolambda*. It is much less distinct in *Arctocyon* and *Ectoconus* than in either *Periptychus* or *Pantolambda*.

The anconeal process of *Periptychus* is strongly asymmetrical and prominent, protruding anteriorly relative to the anterior border of the olecranon process. In anterior aspect, the medial portion of the anconeal process faces medially to articulate with the expanded medial rim of the humeral trochlea. In *Periptychus* (and in *Ectoconus*), it lacks a medial protrusion beyond the proximodistal midline of the ulna. This is unlike the case in *Arctocyon* and *Pantolambda*, in which there is a small medial projection at the proximal-most border of the facet. The lateral portion of the anconeal process is comparatively larger than the medial portion, and protrudes laterally to form a lip, which projects at almost a right angle to the medial portion of the anconeal process so that the articular surface faces distally. In *Arctocyon* and *Pantolambda*, the lateral edge of the anconeal process flares proximally as well as laterally to articulate with the steep lateral wall of the humeral trochlea.

The coronoid process of *Periptychus* is large in terms of its surface area, but is low (not prominently offset from the bone), with a semi-rounded apex which does not project to the same anterior extent as the anconeal process. However, the proximal flank of the coronoid process forms a salient articular facet on the medial portion of the trochlear notch. In *Ectoconus*, *Pantolambda*, and *Arctocyon*, the coronoid process is comparatively taller relative to the height of the anconeal process than that observed in *Periptychus*, but still does not protrude beyond the anterior boundary of the anconeal process. In *Periptychus*, the proximal flank of the coronoid process is subtly concave, forming a shallow facet that faces proximoanteriorly and is continuous with the trochlear notch.

The lateral flank, or radial notch, of the coronoid process of *Periptychus* is relatively large and subequal to the proximal flank surface area in size. In *Arctocyon* and *Pantolambda*, the radial notch is relatively smaller and approximately half the size of the proximal flank of the coronoid process. In *Periptychus*, the radial notch is positioned on the lateral portion of the trochlear notch, adjacent to the medial flank of the coronoid process, with the lateral border of the radial notch forming a lip that overhangs the ulna shaft. The articular surface forms a concave inverted subtriangular profile that is orientated anterolaterally. *Pantolambda* and *Ectoconus* both possess a similarly shaped sub-triangular facet, but in *Ectoconus* the facet is orientated closer to the lateral axis. In *Arctocyon*, the facet is orientated anterolaterally, but is much more circular in shape with a slightly raised border. In *Periptychus*, the medial lateral flanks of the coronoid process converge at a point just medial of the proximodistal midline of the ulna to form the blunt apex of the coronoid process. From a distal perspective, the angle formed by the two flanks is approximately 100°. In *Pantolambda*, this angle is approximately 90°, whereas in *Ectoconus* and *Arctocyon* the coronoid process flank angle exceeds 100°. On the anterior surface of the ulna of *Periptychus*, distal to the apex of the coronoid process (which is medially offset from the proximodistal midline of the ulna), is a small proximodistally aligned sulcus for the insertion of the brachialis. The condition in *Periptychus* is broadly comparable to that of *Arctocyon*, but not as developed as in *Pantolambda*, where the sulcus is both deeper and more pronounced.

The diaphysis of the ulna of *Periptychus* bears several prominent furrows along its length for the attachment of numerous muscles. The lateral surface of the diaphysis exhibits a shallow longitudinal groove extending along the posterior edge of the diaphysis from the olecranon to just distal of the radial notch. This fossa is for the lateral origin of the flexor digitorum profundis. A sharp crest demarcates the posterior border of the groove for approximately half of its length and provides attachment for the lateral triceps brachii. Five foramina perforate the lateral surface of the ulna, just posterior to the trochlear notch. A second longitudinal groove on the lateral ulna surface extends along the anterior border of the ulna from the coronoid process to the styloid process. This groove was for the abductor pollicis longus and is posteriorly delimited by a sharp crest for the attachment for the extensor pollicis longus and the indicis proprius. Anteriorly, the groove for the abductor pollicis longis it is demarcated by the interosseous crest, which forms a sharp medially inflected ridge. The groove for the abductor pollicis longis fossa is also well defined in *Arctocyon*, but it is not as deep as in *Periptychus*, and its distal border is much less defined. In *Pantolambda*, the borders of the fossa are not as strongly defined as in *Periptychus* and *Arctocyon*, but the surface area of the fossa is larger and mediolaterally broader.

On the medial surface of the ulna diaphysis, a long groove extends distally along approximately 50% of the ulna shaft length for the origin of the medial flexor digitorum profundus, and is particularly deep in the portion which runs posterior to the articular region of the ulna. The groove in *Periptychus* is deep and clearly defined by high walls, particularly along its proximal portion. In *Arctocyon*, the borders of the fossa are not as sharply expressed, but the fossa is much larger due to the broader mediolateral depth of the ulna. In *Pantolambda* the fossa is poorly expressed and much shorter, only extending about a third of the way down the bone shaft. Towards the distal end of the radial diaphysis of *Periptychus* there is a small tubercle positioned on the medial surface, just proximal to the styloid process, for the origin of the short radial collateral ligament.

**Fig 26:**
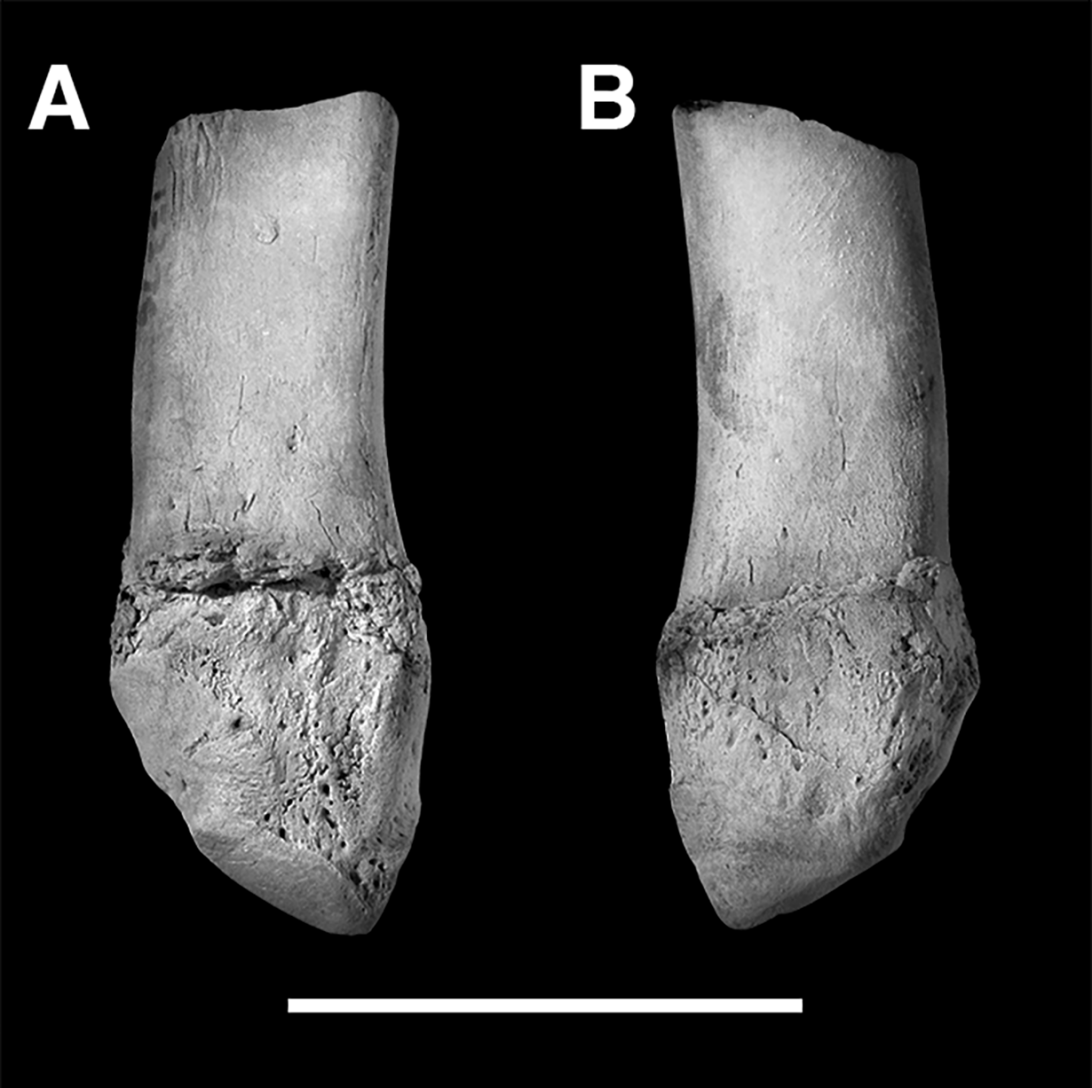
Left distal ulna of *Periptychus carinidens* (USNM 15376). (A) anterior view; (B) posterior view. (specimen has been coated in ammonium chloride prior to photographing). Scale bar: 30mm.

In *Periptychus*, the distal epiphysis of the ulna is not fused with the radius, but together the two bones form a closely articulated and broad distal antebrachium that is mediolaterally wider than dorsopalmarly deep (Fig 26). The distal ulnar head and styloid process are nearly continuous and separated by only a shallow groove for the tendon of the extensor carpi ulnaris muscle. The distal head of the ulna is broadly rounded and larger than that of *Arctocyon*, but not as robust as in *Pantolambda*, where the distal epiphysis is mediolaterally expanded. In *Periptychus*, the styloid process is present, but short and positioned posteriorly as seen in *Arctocyon*, rather than centrally positioned as in *Pantolambda*. It is roughly conical and forms a broadly rounded distal projection.

#### Radius

**Fig 27:**
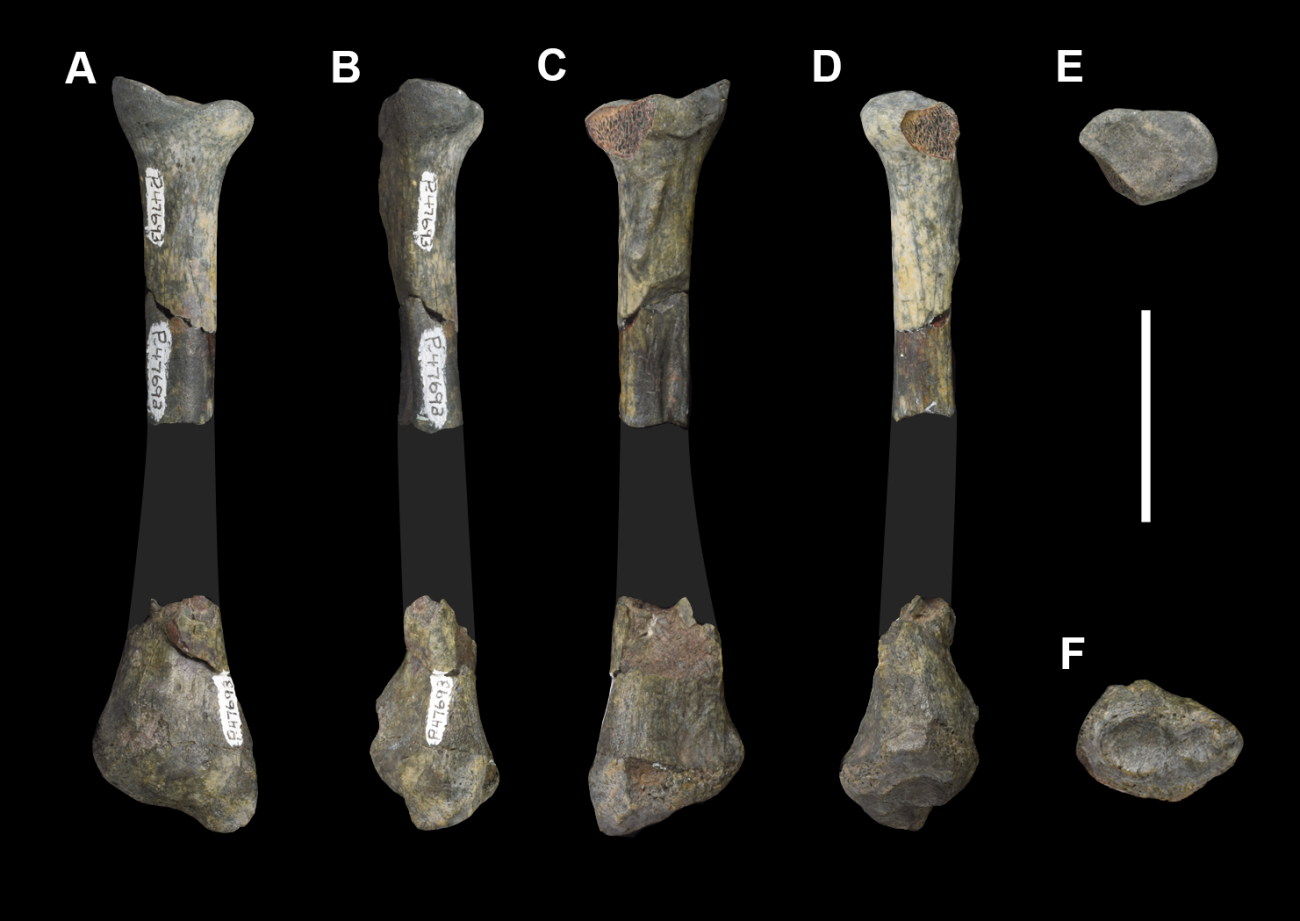
Right radius of *Periptychus carinidens* (NMMNH P-47693). (A) anterior view; (B) lateral view; (C) posterior view; (D) medial view; (E) proximal view, (F) distal view. Scale bar: 30mm.

The following description of the radius of *Periptychus* is based on several specimens. NMMNH P-47693 includes the proximal and distal epiphyses of a right radius (Fig 27). The proximal epiphysis includes part of the articular surface and the proximal portion of the diaphysis, with some damage to the medial facet of the articular surface. The distal fragment preserves the entire epiphysis. NMMNH P-53998 includes a proximal fragment of a right radius, preserving part of the articular surface and the proximal portion of the diaphysis. The specimen is weathered and exhibits some damage to the lateral facet of the articular surface. AMNH 17075 is a nearly complete left radius (figured in [5]). Comparisons are made to *Ectoconus ditrigonus* (AMNH 16500, figured in [5]), *Mithrandir gillianus* (NMMNH P-3038, figured in [48]), *Arctocyon primaevus* (MNHN.F.CR20, CR21 figured in [52]) and *Pantolambda bathmodon* (AMNH 16663, figured in [5]).

**Fig 28:**
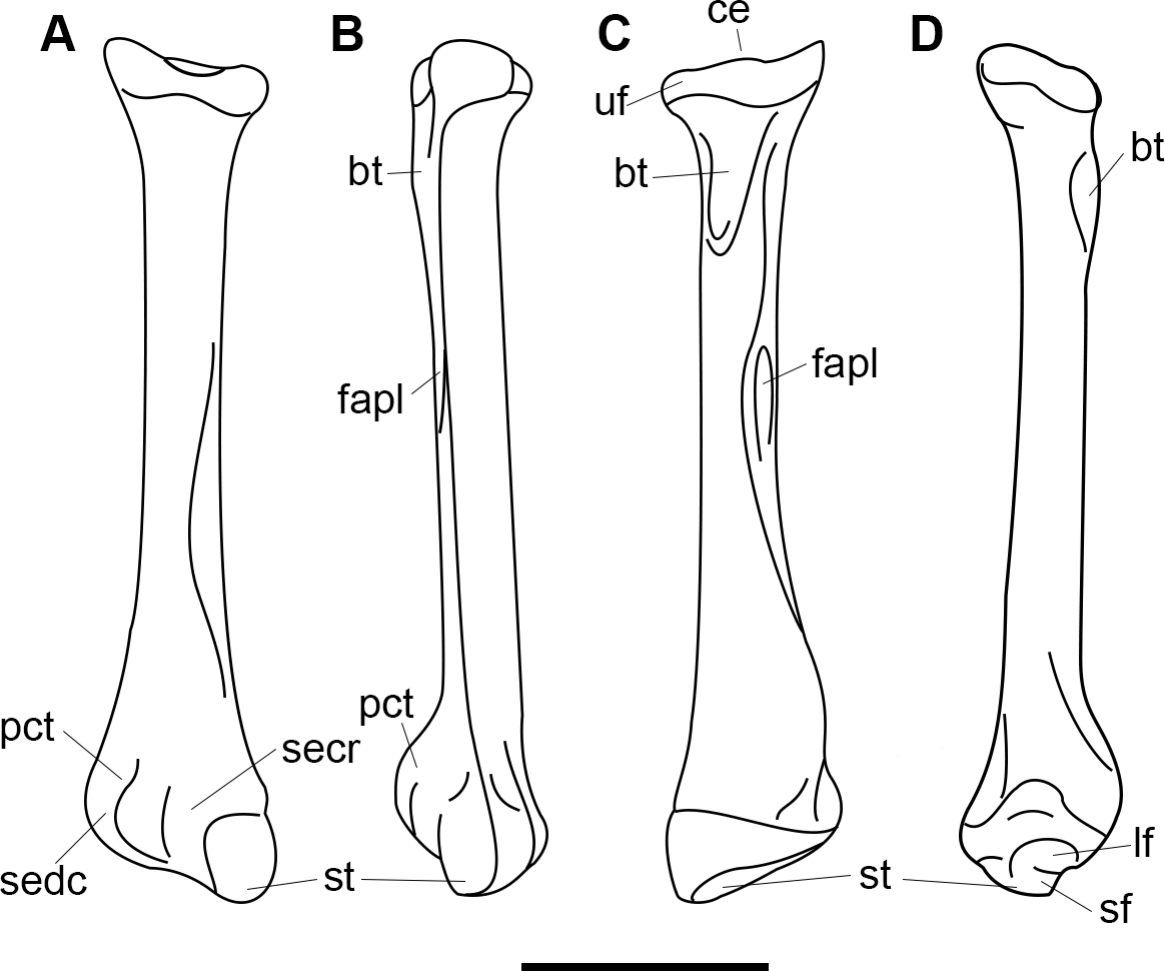
Annotated line drawing of the right radius of *Periptychus carinidens*. (A) anterior view; (B) lateral view; (C) posterior view; (D) medial view. Abbreviations: bt, bicipital tuberosity; ce, capitular eminence; fapl, fossa for the abductor pollicis longus; lf, lunate facet; secr, sulcus for the extensor carpi radialis; sedc, sulcus for the extensor digitorum communis; sf, scaphoid facet; st, styloid process; uf, ulnar facet. Scale bar: 30mm.

The radius of *Periptychus* is a robust element, particularly in comparison to the relatively gracile ulna. It is short and stout, and possesses mediolaterally broad proximal and distal epiphyses (Figs 27 and 28). This contrasts with the condition in *Arctocyon*, in which the radius is relatively gracile in comparison to the robust ulna and the proximal and distal epiphyses are not as proportionally broad. The brachial index (radial length/humeral length x 100) [101] for *Periptychus* (based on AMNH 3636) is 82, indicating that *Periptychus* has a comparatively long radius relative to its humerus. The brachial index for *Periptychus* is higher than that in any of the other comparative taxa; in *Ectoconus* (AMNH 16500) it is 70, *Pantolambda* (AMNH 16663) 73 and *Arctocyon* (MNHN.F.CR16, MNHN.F.CR20) 77.

In anterior aspect, the proximal end of the radius of *Periptychus* is mediolaterally broad and strongly asymmetrical. The degree of asymmetry among observed specimens is variable. However, all specimens display the same general morphology. Observed differences can likely be attributed to post-burial deformation. In proximal aspect, the proximal articular surface is elliptical in profile and almost twice as wide mediolaterally as anteroposteriorly. The radial fovea is broadly concave and dominated by an axisymmetrical central depression. The shallow radial fovea of *Periptychus* is comparable to that of *Ectoconus*, *Pantolambda* and *Arctocyon* in its morphology. However, it is proportionally shallower and more mediolaterally elongate than in *Mithrandir gillianus*. The medial border of the fovea in *Periptychus* is convex to form a distally directed rim, which articulates with the lateral rim of the trochlea.

Laterally, the radial fovea is flanked by a highly expanded crescent shaped flange, which protrudes proximally and acts to stabilise the radius against the mediolaterally broad capitulum. The morphology of *Periptychus* is also seen in *Pantolambda*, which has a tall and greatly expanded lateral flange. In *Ectoconus* and *Arctocyon*, the flange is expanded, but does not project to the same degree as in *Periptychus* or *Pantolambda*. In *Periptychus*, the capitular eminence is small and broadly rounded. It forms a distinct proximal protuberance on the anterior rim of the proximal epiphysis, but it does not project above the lateral flange. The ulnar facet on the posterior surface of the proximal epiphysis forms a gently convex articular surface, which is mediolaterally wide and proximodistally short. Distal to the ulna facet, on the posterior surface of the proximal portion of the diaphysis, is a bicipital tuberosity. The tuberosity forms a raised, proximodistally elongate, inverted triangular area for the insertion of the tendon of the biceps brachii. Argot [52] described the bicipital (= radial) tuberosity of *Arctocyon* as ‘weak’, and although the tuberosity is more prominent in *Arctocyon*, it is not as proximodistally elongate as in *Periptychus*. In *Periptychus*, the tuberosity extends along approximately 18% of the radial diaphysis, whereas in *Arctocyon* is extends along 13%. *Periptychus* is comparable to *Pantolambda*, where the tuberosity extends along 19% of the radial diaphysis.

The radial diaphysis of *Periptychus* is straight. This is also the case in *Ectoconus* and likely in *Mithrandir* based on the known portions of NMMNH P-3038. This is in stark contrast to the highly sigmoidal radius of *Pantolambda* and the anteriorly bowed radius of *Arctocyon*. In cross section, the proximal radial diaphysis of *Periptychus* is roughly circular. Distally, it becomes subtriangular as the posterior surface flattens and a well-defined, but low, longitudinal crest develops for the insertion of interosseous ligaments. A small sulcus is positioned at the proximal end of the crest. Distally, the crest descends along two thirds of the posterior surface of the radius to the distal epiphysis, where it does not protrude strongly from the diaphysis.

A weak longitudinal pronator crest, with a slight lateral curve, is positioned two thirds of the way along the proximodistal length of the ulna for the insertion of the pronator teres (at the apex) and the pronator quadratus (distal to the pronator teres insertion site). In *Ectoconus*, *Arctocyon* and *Pantolambda* this crest is proximodistally longer and sharper, with the apex positioned higher (approximately halfway) on the diaphysis. In *Periptychus*, the pronator crest terminates in a small rounded tuberosity positioned close to the proximodistal midline of the long axis of the bone, on the anterior border of the distal epiphysis. This tuberosity separates two shallow, but mediolaterally broad, grooves for the tendon of the extensor carpi radialis medially and both the extensor digitalis communis and digitorum lateralis laterally. The condition in *Periptychus* is like that in *Ectoconus* and *Pantolambda*, none of which possess a morphology as pronounced as in *Arctocyon*, in which the tuberosity forms a very prominent protuberance positioned near the medial border of the anterior surface of the distal epiphysis, separating two comparatively deeper grooves.

A small styloid process is positioned on the anteromedial border of the distal epiphysis of *Periptychus*. It forms a small, but broad, protuberance with gently sloping walls. A small, round, slightly concave facet for articulation with the scaphoid extends onto the gently sloping distal surface of the styloid process. A second, larger facet is located on the lateral half of the distal epiphysis for articulation with the lunate. The scaphoid and lunate facets are distinctly separate in *Periptychus*. The condition of *Periptychus* is distinct from that in *Arctocyon*, in which the scaphoid and lunate facets converge and the scaphoid facet forms a flat surface rather than a concave depression.

#### Manus

**Fig 29:**
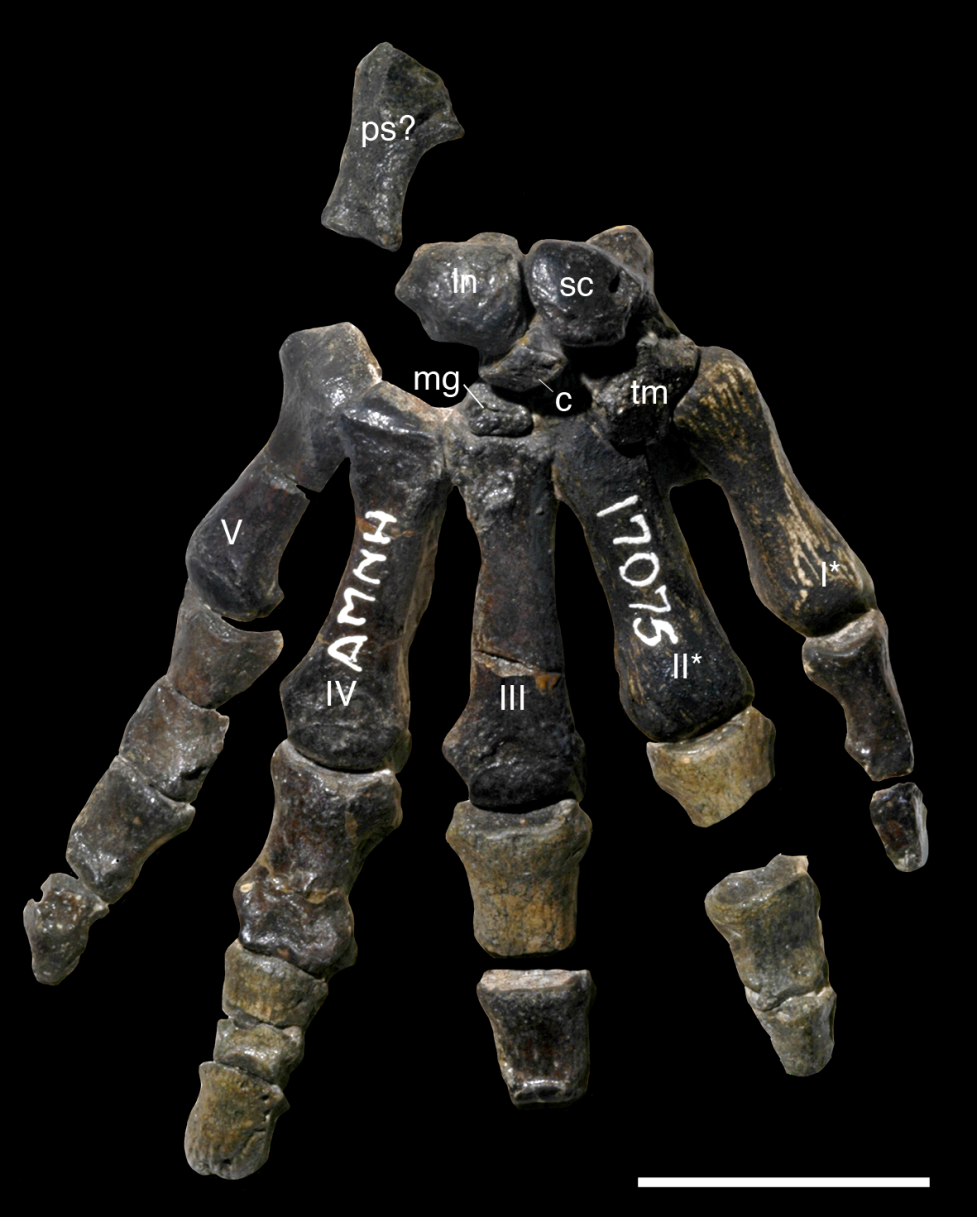
Right manus of *Periptychus carinidens* (AMNH 17075) in dorsal view. Abbreviations: c, centrale; ln, lunate, mg, magnum, ps, pisiform, sc, scaphoid, tm, trapezium; I-V refer to digit number; * denotes reconstructed elements. Scale bar: 30mm. Note that this specimen is reconstructed and mounted on a resin base. Photographs of the individual bones are illustrated and photographed in [5].

The following description of the manus of *Periptychus* is based on AMNH 17075, a well preserved partial right manus consisting of an associated scaphoid, lunate, centrale, magnum, trapezium and pisiform, with metacarpals III, IV, V and all of the proximal phalanges (although the distal ends of proximal phalanges II and III are damaged), all the intermediate phalanges (except on digit I) and four (I, II, IV and V) of the distal phalanges (Fig 29).

The specimen is mounted in articulation on a solid base mount with some dubious positioning of the carpal elements that do not accurately represent the true articulated position of the bones. As a result, the palmar surfaces of the bones and the details of the articulations cannot be observed. It should also be noted that there is some discrepancy in the literature over which metacarpals are present. Matthew [5] writes that II, IV and V are preserved (p.118), but in his Fig 23 ([5] p.120) only III, IV and V are drawn, and in the appending photographic plates ([5], p.412, plate XX) four metacarpals are shown, but not identified in the legend (we tentatively identify them as I, V, IV and III, from left to right). Based on our observations of AMNH 17075 as it is currently curated, it appears that only three metacarpals are present (III, IV and V, with I and II being reconstructed on the mount).

Several bones of the manus are associated in another new specimen: NMMNH P-47693, which includes a nearly complete pisiform with some damage to the distal articulation surface, a metacarpal diaphysis, a proximal portion of a phalanx which we interpret as the third proximal phalanx, a phalanx which we interpret as the second intermediate phalanx, and a phalanx which we interpret as the third intermediate phalanx. Due to the lack of any other manus material associated with NMMNH P-47693 and the inability to compare the specimens with the palmar surface of AMNH 17075, the assignment of the bones to specific digits is somewhat tentative. The aforementioned digital bones of NMMNH P-47693 could be interpreted as tarsal bones given that the distal manual and tarsal elements are near identical.

The unciform, cuneiform, and trapezoid are not definitively known for *Periptychus*, but are inferred to be present in the manus during life based on the shape and configuration of the known carpal elements. In the following text, the manus is described along the three main axes: proximodistal, lateromedial and dorsopalmar. The dorsopalmar axis corresponds to dorsoventral axis because the manus of *Periptychus* is plantigrade. Comparisons of the manual elements are made with *Ectoconus ditrigonus* (AMNH 16500, figured in [5]), *Arctocyon primaevus* (MNHN.F.CR34, CR38, CR39, CR17474, CR36, CR57, 1307, CR35, CR823, CR40, CR66, CR44, CR43, CR45, CR46, CR54; figured in [52]), *Claenodon corrugatus* (AMNH 16543, figured in [5]) and *Pantolambda bathmodon* (AMNH 16663, figured in [5]).

The manus of *Periptychus* is plantigrade and pentadactyl, with broad, widely spaced digits. The carpus is composed of at least eight bony elements: the scaphoid, lunate, and cuneiform form the proximal carpal row, and the unciform, magnum, centrale, trapezoid and trapezium form the distal carpal row, with at least one known sesamoid (the pisiform). Furthermore, the prepollex sesamoid was mostly likely present [102], but has yet to be identified from fossils. The carpal arrangement forms a nearly alternating sequence in which the scaphoid does not contact the magnum, but the lunate has a broad contact with the unciform. The digits of *Periptychus* are robust and the pollex is well developed. The phalangeal formula conforms to the generalized mammalian condition (2-3-3-3-3).

#### Proximal carpal joint

The proximal carpal joint is formed by the articulation between the ulna and radius proximally with the scaphoid, lunate and cuneiform distally. The pisiform is also considered part of the proximal carpal joint. The ulna and radius are not fused, but together form a closely articulated and broad antebrachium that is mediolaterally wider than dorsopalmarly deep. The proximal articular surface formed by the scaphoid, lunate and cuneiform mirrors the antebrachium in that the mediolateral width of each element is longer than its dorsopalmar depth. The scaphoid and lunate are separate bony elements, but their combined mediolateral width would have been greater than the width of the cuneiform (based on the assumed minimum width of cuneiform inferred from metacarpal width). The transition between the proximal surface of the scaphoid and lunate of *Periptychus* does not form as tight an articulation as in *Arctocyon*. This relationship also manifests itself in the distal articular surface of the radius. In *Periptychus* the scaphoid and lunate facets are distinctly separate, whereas in *Arctocyon* they form a continuous articular surface. The proximal surface formed by the scaphoid and lunate is reasonably flat, although when considering the medial articular surface of the scaphoid (and omitting the scaphoid tubercle), the actual articular surface is convex. It is much more convex in *Periptychus* than in *Ectoconus* or *Pantolambda*.

#### Scaphoid

The scaphoid is a large element in the carpus (Fig 29), positioned on the medial side of the proximal row of carpal bones. It is subequal in size to, and separate from, the lunate. Proximally it articulates with the distal radius. In dorsal aspect, the bone is subquadrilateral (when considering the scaphoid tubercle), with a mediolateral axis longer than the proximodistal axis. It is dorsopalmarly shallow. The scaphoid of *Periptychus* most closely resembles those of *Ectoconus* or *Pantolambda* and is not as mediolaterally broad as the scaphoid in *Claenodon*, in which the mediolateral width of the bone is greater than twice the proximodistal depth. The condition of *Claenodon* is notably different from that in *Arctocyon*, in which the scaphoid is anteroposteriorly deeper than mediolaterally broad.

In *Periptychus*, the dorsal-most surface of the scaphoid (excluding the palmar process) is convex and exhibits a small fovea on its medial edge. This feature is most likely caused by damage to the bone, particularly as no other observed taxa exhibit such a condition. However, the condition of the specimen makes it hard to be certain of this and the fovea may potentially be a ligament attachment site.

In *Periptychus*, the distal border of the proximal surface of the scaphoid overhangs the distal articular surface to limit dorsiflexion of the carpus. The proximal surface of the scaphoid exhibits a facet for articulation with the styloid process of the radius. In proximal view, the facet tapers medially to form a triangular articular surface that is dorsopalmarly convex. The lateral border of the facet is dorsal relative to the tapered medial border, with the mediolateral axis of the facet exhibiting some convexity. The medial edge of the facet descends to the distal border of the scaphoid, creating a deep support for the styloid process of the radius. *Ectoconus* exhibits a similarly deep facet, although it is not as pronounced as in *Periptychus*. In *Claenodon* and *Arctocyon*, the facet descends only approximately half of the proximodistal height of the scaphoid.

The scaphoid tubercle of *Periptychus*, or palmar process, forms a small, but robust, hook-like process, which projects medially and somewhat posteriorly from the main body of the scaphoid. Laterally, the scaphoid articulates with the lunate and centrale. The contact between the scaphoid and centrale in *Periptychus* is highly expanded and much broader than in *Ectoconus*, despite the centrale of *Ectoconus* being of comparable size. Distally, the scaphoid of *Periptychus* articulates with the trapezium and trapezoid. The nature of the medial and distal contacts of the scaphoid are unobservable due to the specimen being mounted in articulation; however, brief descriptions were provided by Matthew ([5] pp.137-138).

#### Lunate

In *Periptychus*, the lunate is a large, separate element on the proximal row of carpal bones (Fig 29). It is large, and subequal in size to the scaphoid. These two bones have similar dimensions in that they are mediolaterally wide and proximodistally long, but dorsopalmarly shallow. The dorsal surface of the lunate is broadly convex and forms a rounded 6-sided polygonal profile, with the mediolateral width subequal to the proximodistal length. The proximal surface is somewhat continuous with the smooth dorsal surface. The radius articulates with a small protuberance on the mediopalmar edge of the proximal surface of the lunate.

The distal surface of the lunate is formed by two articular facets that converge into a blunt, mediodistally directed apex. The medial facet provides a large articular area for the centrale. The contact between the lunate and centrale is greatly expanded in *Periptychus*. The centrale is at least double the size of that in any of the other comparison taxa, and consequently, the lunate has a 6-sided polygonal dorsal profile rather than the more typical 5-sided profile. The interpretation of the contact of the lunate with the magnum and the unciform is somewhat subjective. Matthew asserts that the articulation between the lunate and magnum does not reach the dorsal surface due to the enlargement of the centrale [5]. While this is plausible, it is also possible to observe two facets on the lateral portion of the distal surface. The small, sub-rectangular medial-most facet could be interpreted as the contact with the magnum and the larger sub-triangular lateral facet for contact with the unciform. The lack of a known unciform for *Periptychus* and the inability to manipulate the known carpal bones in the mount leads to ambiguity.

The medial-most facet on the distal surface of the lunate is mediolaterally narrow (forming the shortest side in the dorsal 6-sided polygon profile) and proximodistally deep, giving the facet a concave rectangular profile that is orientated to face distally. The lateral facet on the distal surface for the unciform is broader at its dorsal border, and tapers palmarly to form a concave facet that is broadly orientated laterodistal relative to the dorsal surface of the lunate. The proximal border of this facet is demarcated by a dorsopalmarly aligned protuberance that would underlay the cuneiform in life. Laterally, the lunate articulates with the cuneiform via a large, rounded, concave facet.

The overall morphology and dimensions of the lunate of *Periptychus* most closely resemble those of *Ectoconus* and *Pantolambda*, rather than *Claenodon*. The lunate of *Claenodon* is proportionally smaller relative to the rest of the carpus and its dorsal profile forms an angular, 5-sided polygon with a distally directed apex. In *Ectoconus*, the lunate is proportionally similar in size to *Periptychus*, relative to the other carpal bones, with a mediodistally directed apex. However, the contact between the lunate and centrale is much smaller than in *Periptychus*, while the contact with the magnum is much broader. The condition in *Pantolambda* bears the most resemblance to *Periptychus* in that the centrale is somewhat expanded, although the magnum retains a relatively large contact with the lunate. The lunate of *Pantolambda* differs from that of *Periptychus* in that the bone is much more rounded in *Pantolambda*, so that its dorsal profile is more ovoid than polygonal and the articular surface with the radius is much broader and less prominent.

#### Pisiform

**Fig 30:**
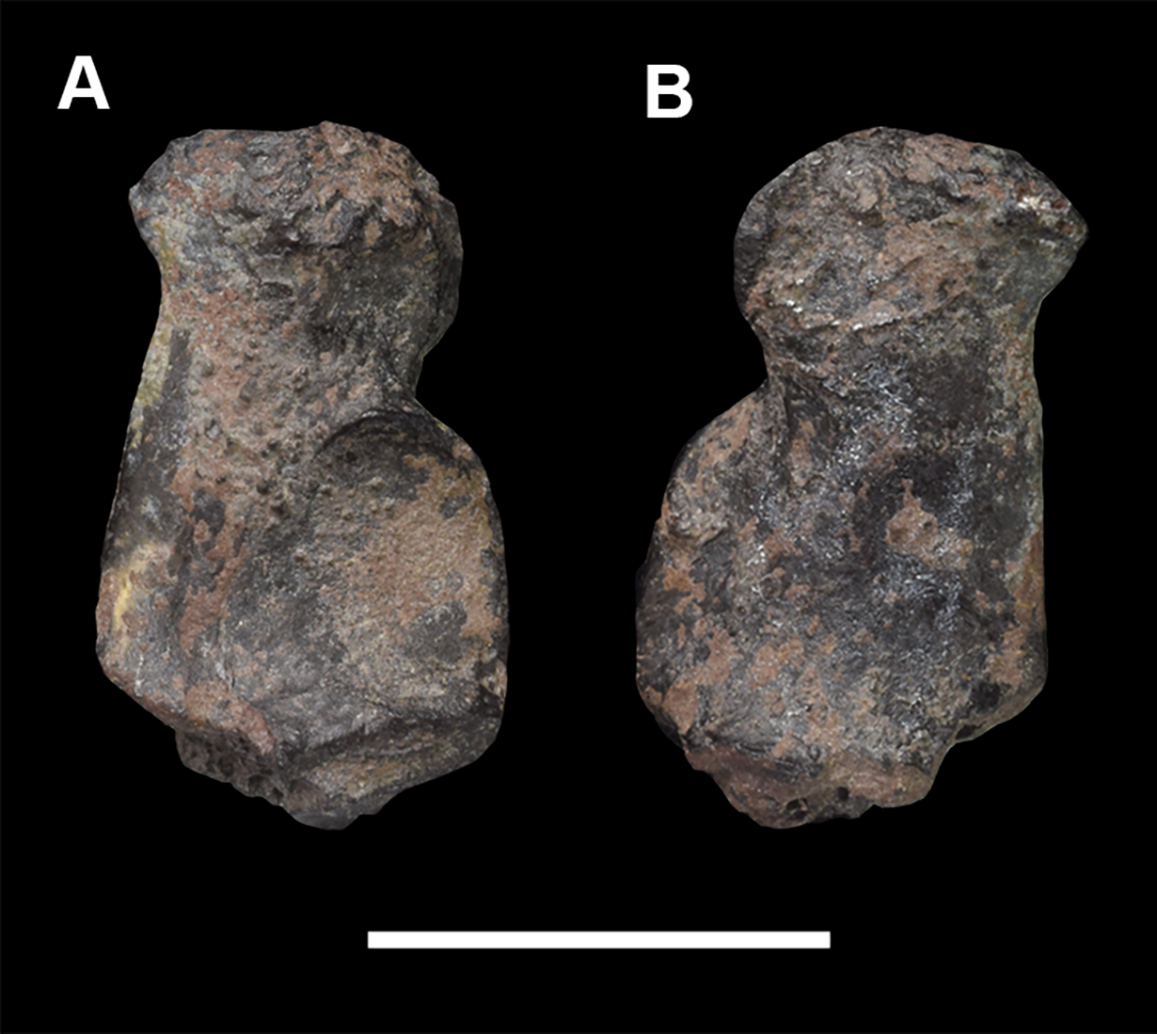
Pisiform of *Periptychus carinidens* (NMMNH P-47693). (A) dorsal view; (B) plantar view. Scale bar: 30mm.

The pisiform of *Periptychus* is relatively large and robust relative to the size and proportions of the other carpal elements. It is positioned on the proximolateral border of the carpus, forming an elongate projection. The bone is proportionally long, as it is approximately 50% of the proximodistal length of metacarpal V on AMNH 17075 (Fig 29). In NMMNH P-47693 the pisiform appears to be proportionally smaller, but the associated metacarpal V is unknown (Fig 30). The pisiform is flattened dorsopalmarly, creating mediolaterally enlarged dorsal and palmar surfaces for muscle attachments. The proximal head of the pisiform is broad, and expands into a rounded tuberosity for the insertion of the flexor carpi ulnaris. The pisiform also provided an origination site for the abductor digiti minimi and attachment for the transverse carpal ligaments.

The ulna articulates with the pisiform via a concave, ovoid facet positioned on the dorsomedial surface. The facet extends onto the distal half of the medial side of the bone and expands out medially from the main body of the pisiform. It is difficult to infer the angle of projection of the pisiform from the main body of the carpus, particularly as the cuneiform is not known for *Periptychus*. Given the size of the ulnar facet, the pisiform would have maintained a large contact with the ulna for approximately half its length. Therefore, it would not have formed a large projection beyond the carpal body. In *Ectoconus*, the pisiform projects posteriorly and somewhat medially, and it is reasonable to infer a similar condition in *Periptychus* given the general similarities in pisiform morphology. Distally, the pisiform articulates with the cuneiform via a mediolaterally broad facet, forming a dorsopalmarly concave depression. The pisiform of *Periptychus* is broadly comparable to that of *Ectoconus* in that the ulnar facet forms a large ovoid facet on the dorsomedial side of bone, with the head forming a rounded tuberosity. *Periptychus* differs from *Ectoconus* in that its pisiform is more dorsopalmarly compressed and the ulnar facet is proportionally much larger. The condition of *Periptychus* and *Ectoconus* is notably different from that of *Arctocyon* and *Pantolambda*, in which the ulnar facet is a relatively smaller triangular structure, positioned on the on the distal end of the pisiform (which is less dorsopalmarly compressed and proportionally much longer, with a smaller head), adjacent to the cuneiform facet.

#### Midcarpal joint

The midcarpal joint is formed by articulations between the scaphoid, centrale, lunate, and cuneiform proximally, with the trapezium, trapezoid, magnum and unciform distally, and with the prepollex sesamoid medially, if it was present. The midcarpal articulations are more closely associated with carpal loading and joint stability than with mobility of the carpus. The midcarpal joints form a sequence that more closely resembles an alternating, rather than a serial, carpus. The scaphoid articulates with the trapezoid and trapezium, but not the magnum, as would be expected in a true alternating carpus. However, the lunate retains a broad contact with the unciform, which is a diagnostic feature of a serial carpus.

#### Centrale

The centrale of *Periptychus* is a distinct and separate element of the carpus. It is mediolaterally and proximodistally small, but dorsopalmarly elongate, so that the bone forms an irregular, sub-rhomboidal prism set at an oblique angle within the carpus (Fig 29). The dorsal exposure of the centrale is greatly expanded so that it is subequal in size to the dorsal exposure of the magnum. The majority of the centrale enlargement has occurred along the mediolateral axis, with expansion towards the scaphoid and the trapezium. As described above, the expansion of the centrale may or may not be enough to exclude the magnum from contacting the lunate. The dorsal surface of the centrale exhibits two, subparallel, short articular surfaces that are slightly offset from the proximodistal axis. Additionally, there are two longer subparallel surfaces (approximately twice the length of the proximodistal surfaces) that are slightly offset from the mediolateral axis. The proximal surface of the centrale articulates with the scaphoid, whereas the distal surface contacts the magnum (regardless of whether the magnum contacts the lunate, the centrale will always contact the magnum distally). The medial and lateral surfaces of the centrale make broad contacts for the trapezoid (not known) and the lunate, respectively.

The carpal configuration of *Periptychus* is very different from that of *Ectoconus* in that the dorsal exposure of the centrale in *Ectoconus* is reduced to a tiny trapezoid-shaped piece of bone (more comparable to *Claenodon* than *Periptychus*), allowing the lunate to make broad contact with the magnum. The condition of *Pantolambda* bears closer resemblance to *Periptychus* in that the centrale is somewhat more enlarged than in *Ectoconus*, but it still allows the magnum a broad contact with the lunate.

#### Magnum

The magnum is a small, separate element on the distal row of carpal bones. The dorsal surface forms a mediolaterally wide, sub-rectangular profile, with a surface area that is slightly smaller than that of the enlarged centrale (Fig 29). The internal portion of the magnum extends proximodistally into the carpus. However, due to the mounting of the specimen, it is not possible to fully observe the internal articular surfaces of the magnum. Proximally, the magnum articulates with the centrale. Distally the magnum sits centrally on metacarpal III and does not overlay it. From what can be observed, the magnum of *Periptychus* is very similar in shape to that of *Ectoconus*, in that the dorsal surface of the bone forms a mediolaterally elongate sub-rectangular profile. In *Pantolambda*, the dorsal exposure of the magnum is relatively smaller with a sub-trapezoidal profile, which is narrower proximally than distally. The dorsal exposure of the magnum in *Pantolambda* is somewhat like that of *Claenodon* in that it is triangular.

#### Trapezium

The trapezium of *Periptychus* is a relatively large and robust element on the medial end of the distal row of carpal bones. The shape of the trapezium is highly irregular, forming a rounded sub-rectangular profile in dorsal view (Fig 29). The shape of the trapezium of *Periptychus* is notably different from that of *Ectoconus*, in which the bone is more triangular in profile and relatively less robust. The condition of *Ectoconus* bears some resemblance to that of *Arctocyon*, where the trapezium is distinctly triangular with a strong proximal apex. In *Periptychus*, the sub-rectangular profile is caused by expansion of the lateral portion of the trapezium.

Interpreting the articular relationships of the trapezium of *Periptychus* is difficult because the specimen is mounted in articulation with metacarpals I and II, both of which have been reconstructed. Therefore, the correct position or orientation of the trapezium cannot be ascertained. In addition, the scaphoid is present, but not positioned in articulation with the trapezium, and so the two bones cannot confidently be placed together in proper articulation. The trapezoid is absent and due to the uncertain position of the trapezium (as well as the other carpal bones), it is difficult to make any assumptions about its size and shape and its relationship with the trapezium. Therefore, the following description is based on AMNH 17075, with the anatomical orientations referring to the specimen as it is currently mounted.

The proximal surface of the trapezium features a large concave facet on its medial portion that is broadest dorsally and tapers somewhat palmarly. The trapezium appears to have a fairly mediolaterally broad contact with the scaphoid, which is broader than in *Ectoconus*. The proximal surface becomes convex laterally and is nearly continuous with the lateral surface. The angle between the scaphoid and trapezoid facets is highly obtuse. The lateral surface is formed from a proximodistally short, concave, sub-triangular facet that would have articulated with the trapezoid. It appears the trapezoid may have overlain the lateral-most portion of the trapezium based on AMNH 17075. In both *Ectoconus* and *Pantolambda* the trapezoid is mediolaterally broad, precluding the trapezium from contacting the centrale. It is therefore likely that this is also the condition in *Periptychus*. However, the centrale of *Periptychus* is much larger than the scaphoid and it is not mediolaterally broad enough to preclude contact between the two elements. Distally, the trapezium articulates with metacarpal I and possibly metacarpal II. The distal portion of the trapezium is mediolaterally broad and exhibits a large, medially orientated, saddle-shaped articular area for the pollex that would have facilitated a reasonably broad range of motion. The lateral portion of the distal trapezium is distally expanded to form a small, but robust, protuberance that displaces the pollex medially while apparently maintaining contact with metacarpal II. In *Ectoconus*, the trapezium is laterally expanded to medially displace the pollex, but it does not contact metacarpal II.

#### Metacarpals

The metacarpals of *Periptychus* are robust and mediolaterally broad, but remain well spaced from each other when in articulation. The proximal and distal epiphyses are broad relative to the diaphysis, which is distinctly dorsopalmarly flattened. Only the third, fourth and fifth metacarpals are definitively known from an associated manus of *Periptychus* (AMNH 17075) (Fig 29). The third metacarpal is the longest, the fourth is slightly shorter (4% shorter than the third), and the fifth is considerably reduced (32% shorter than the fourth). Both *Ectoconus* and *Pantolambda* show the same trend, with the third metacarpal longest and the subsequent metacarpals becoming shorter. In *Ectoconus* the percentage differences between the bones are identical to *Periptychus*. However, in *Pantolambda* there is less shortening between the third and fourth metacarpals (1%) and a 35% difference between the fourth and fifth metacarpals. All three taxa have a 36% gross difference in proximodistal length between the third and fifth metacarpal.

The proportions of the metacarpals can be quantified using two standard indices. The robusticity of the metacarpals can be assessed using the ratio of the mediolateral width of the distal end divided by the proximodistal length of metacarpal III [69]. In *Periptychus* (AMNH 17075) the ratio is 0.35. This is identical to the value for *Pantolambda* (AMNH 16663) and smaller than that of *Ectoconus* (AMNH 16500), which has a ratio of 0.41. Based on this index, the metacarpals of *Periptychus* are proportionally as robust as *Pantolambda* and less robust than *Ectoconus*. It is worth noting that the metacarpal diaphyses of *Ectoconus* constrict towards the middle of the shaft, a feature that isn’t taken into consideration by this index.

The relative lengths of the metacarpals can be assessed using the ratio of the proximodistal length of metacarpal III divided by the proximodistal length of an associated humerus or ulna [69]. In *Periptychus* (AMNH 17075) the ratio based on the humerus length is 0.25, and based on ulna length is 0.28. In *Pantolambda* (AMNH 16663) the humerus ratio is 0.31 and ulna ratio is 0.31, and in *Ectoconus* (AMNH 16500) the two ratios are 0.21 and 0.18, respectively. Therefore, the third metacarpal of *Periptychus* is longer relative to both the humerus and ulna than is the third metacarpal of *Ectoconus*, but proportionally shorter compared to *Pantolambda*.

To summarize, the metacarpals and manus of *Periptychus* are proportionally long relative to its forelimb, whereas in *Ectoconus* the metacarpals and manus are comparatively short relative to the forelimb. The manus of *Pantolambda* is very robust for its size and long relative to the forelimb (its forelimb is short in comparison to the periptychids). It is worth noting that the manus of all three taxa are very similar in overall size despite the varying proportions of their manual elements, disparity in forelimb length and morphology, and overall body size.

The third metacarpal is nearly symmetrical along its proximodistal long axis. Proximally it articulates with the magnum via a large, concave, dorsopalmarly deep facet. The lateral and medial edges of the facet would have contacted the unciform and trapezoid, respectively, and prevented the magnum from contacting the second and fourth metacarpals. The unciform and trapezoid are not known for *Periptychus*, but their articular surfaces on the third metacarpal do not appear to be particularly large. This is notably different from the condition in *Ectoconus*, and also in *Claenodon*, in which the unciform and magnum facet are subequal in width.

AMNH 17075 is mounted so that the third metacarpal overlays the second metacarpal (which is reconstructed). There is no obvious facet on the medial surface of the bone so it seems likely that the third metacarpal either lay over the second (like the condition of *Pantolambda*) or barely contacted it at all (as seen in *Ectoconus*).

In *Periptychus*, the proximal end of metacarpal III is more dorsopalmarly deep than the distal end, but its longest axis is still mediolaterally directed. The dorsal surface of the proximal end is damaged but appears to possess two well-developed tuberosities separated by a defined sulcus. The lateral-most tuberosity is positioned more distally and provided attachment for the transverse head of the adductor pollicis muscle. The medial-most tuberosity, positioned more proximally, provided attachment for oblique head of the adductor pollicis. These tuberosities are prominent in *Periptychus*, whereas in *Pantolambda* they are less developed. *Ectoconus* has even more pronounced tuberosities than *Periptychus*; they are positioned further distally and both at the same level on the dorsal surface of the bone. In *Periptychus*, the lateral tuberosity delimits the proximal edge of a broad ridge, which extends the length of the metacarpal. The medial tuberosity is highly developed, providing a large attachment surface.

The diaphysis of the third metacarpal is mediolaterally broad and dorsopalmarly compressed, with a smooth surface. The broad dorsal surface would have provided large attachment areas for the dorsal interossei. The mediolateral width of the diaphysis is near constant along its length, with little constriction.

The distal end of the third metacarpal is mediolaterally expanded so that it is broader than the proximal end, but not as dorsopalmarly deep. The distal surface of the bone forms a saddle-shaped articular surface with a dorsopalmar convexity. In dorsal view, the mediolateral long axis of the articular surface is somewhat convex rather than flat like in *Pantolambda* and *Ectoconus*. In *Periptychus* the mediolateral long axis of the distal articular surface is orientated perpendicular to the long axis of the diaphysis, whereas in *Ectoconus* the long axis of the articular surface is orientated obliquely to the long axis of the shaft. In *Periptychus* the articular surface extends well onto the dorsal surface, with its proximal border demarcated by a well-defined triangular fossa that received the dorsal sesamoid. The condition of *Periptychus* is like that of *Claenodon* and differs from that of both *Ectoconus* and *Pantolambda*, in which the articular surface is smaller, and barely extends onto the dorsal surface. In *Pantolambda* the dorsal sesamoid fossa is shallower, but larger relative to the metacarpal in terms of surface area, whereas in *Ectoconus* the fossa forms a deep crescent-shaped groove. In *Periptychus* the distal articular surface is flanked on both sides by a prominent tuberosity, which provided large attachment areas for the radial and ulnar collateral ligaments of the metacarpophalangeal joint. In *Ectoconus* and *Claenodon* the tuberosities are less developed but still form small protuberances, whereas in *Pantolambda* they are greatly reduced and barely protrude at all.

The fourth metacarpal is broadly like the third in its overall morphology, although it is somewhat smaller. Proximally it articulates with the unciform via a flat, trapezoidal shaped facet, which is mediolaterally broader dorsally and tapers somewhat palmarly. Medially the metacarpal IV contacts the third metacarpal via a small proximomedially orientated facet and laterally it contacts the fifth metacarpal via a proximolaterally orientated facet. Both facets are subequal in size, although laterally the fourth metacarpal overlays the fifth somewhat. The diaphysis is not as dorsopalmarly flattened as in the third metacarpal. The distal epiphysis displays the same features as the third metacarpal, although the tuberosities for the radial and ulnar collateral ligaments are not as prominent.

The fifth metacarpal is smaller and stouter in comparison to the third and fourth metacarpals, but retains the same overall morphology. Proximally it articulates with the unciform via a smooth, convex facet. In *Periptychus* the proximal end is subequal in mediolateral width to those of the other metacarpals, whereas in *Claenodon* the proximal epiphysis of the fifth (and also the first) metacarpal is much wider than those of the second, third, and fourth metacarpals. A well-developed tuberosity for the insertion of the extensor carpi ulnaris muscle is positioned on the lateral surface of the metacarpal, just distal to the proximal articular surface. The distal epiphysis lacks a distinct fossa for the dorsal phalangeal sesamoid. A prominent tuberosity is positioned on the lateral surface of the distal epiphysis for the insertion of the opponens digiti minimi muscle.

#### Manual phalanges

The manual phalanges of *Periptychus* are robust elements that become progressively shorter distally. The robustness of the phalanges can be quantified using the ratio of the mediolateral width of the proximal end of the fourth phalanx divided by its proximodistal length [69]. Ideally, the ratio should be based on the third proximal phalanx, but only incomplete specimens are known for *Periptychus*, so we use the fourth proximal phalanx as it closely approximates the third in size and is known for all the comparison taxa. The ratio for *Periptychus* (based on AMNH 17075) is 0.62, compared to 0.71 for *Ectoconus* (AMNH 16500), 0.78 for *Pantolambda* (AMNH 16663) and 0.43 for *Claenodon* (AMNH 16543). As such, the proximal phalanges of *Periptychus* are less robust than those of *Ectoconus* and *Pantolambda*, but more robust than those of *Claenodon*.

The proximal phalanges of *Periptychus* all exhibit a broadly similar morphology: they are mediolaterally broad, dorsopalmarly flattened and nearly symmetrical longitudinally (Fig 29). The third proximal phalanx is marginally larger than the second, fourth, and fifth, whereas the first is the smallest (though still not greatly reduced relative to the others). The proximal ends articulate with their associated metacarpals via shallowly concave facets. The proximal articular surfaces of *Periptychus* are more concave than in both *Ectoconus* and *Pantolambda*. The proximal articular surfaces are flanked on both sides by well-expressed tuberosities for the attachment of the radial and ulnar collateral ligaments, as well as the dorsal interossei (on the medial side of II, the medial and lateral sides of III and the lateral side of IV). As seen in the metacarpals, the medial and lateral tuberosities of *Periptychus* are much more prominent than in *Ectoconus* and *Pantolambda*, despite the overall higher robustness of *Ectoconus* and *Pantolambda*.

The shafts of the proximal phalanges are smooth and somewhat narrower than the proximal and distal ends. In *Ectoconus*, *Pantolambda* and *Claenodon* the proximal ends are mediolaterally wider than the distal ends, so that the shafts taper distally, whereas in *Periptychus* the distal ends are subequal to the proximal ones in mediolateral width, so that the shafts constrict midway along the longitudinal length before widening distally. The distal ends of the proximal phalanges of *Periptychus* are mediolaterally broad, with shallow biconvex articular surfaces. The distal articular surface in *Periptychus* is not as biconvex as in *Arctocyon*, but shapelier than the flat surfaces of *Ectoconus* and *Pantolambda*.

**Fig 31:**
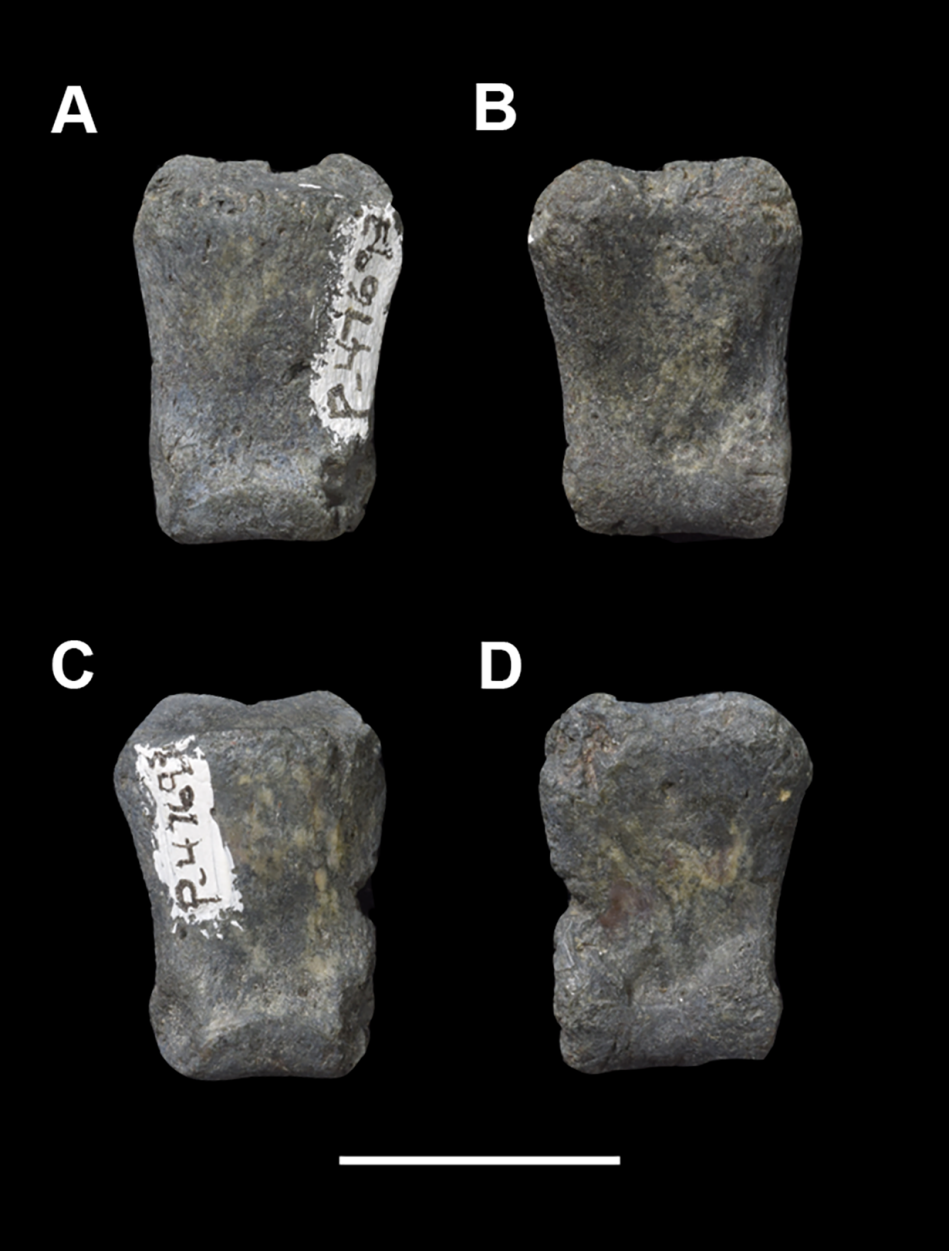
Two intermediate phalanges of *Periptychus carinidens* (NMMNH P-47693). A-B: third intermediate phalange in (A) dorsal view; (B) plantar view. C-D: second intermediate phalange in (C) dorsal view; (D) plantar view. Scale bar: 30mm.

The intermediate phalanges are smaller and shorter than the proximal phalanges (Figs 29 & 31). The third digit of *Periptychus* displays a moderate amount of shortening, with a 39% decrease in proximodistal length from the proximal phalanx to the intermediate phalanx (based on AMNH 17075). This is comparable to the ratio found in *Claenodon*, which has a 37% decrease (based on AMNH 16453); substantially higher than that of *Ectoconus*, which exhibits only a 29% decrease in length (based on AMNH 16500); and less than that of *Pantolambda*, which has a 44% decrease from the proximal to intermediate phalanx.

The proximal ends of the intermediate phalanges of *Periptychus* are reniform in shape, with dorsal edges that provide a small lip for the insertion of the extensor digitorum communis tendon. The shafts are dorsopalmarly compressed. Their dorsal surfaces are smooth and convex mediolaterally and their ventral surface are convex mediolaterally, so that the dorsopalmar depth of each bone is smallest at its longitudinal midpoint (increases proximally and distally). The distal ends are bicondyloid and mediolaterally narrower than the proximal ends.

The distal phalanges are distinctive hoof-like unguals, which are subequal to the intermediate phalanges in proximodistal length. The proximal ends form mediolaterally broad articulations with the intermediate phalanges; these contacts are dorsopalmarly deep in comparison to the other phalangeal contacts. The dorsal edges of the articular surfaces form raised lips for insertion of the extensor digitorum communis tendons. The distal phalanges are mediolaterally broad and dorsopalmarly compressed, each with a rounded tip and a distinct fissure marking the distal half of the longitudinal midline. The hoof-like unguals of *Periptychus* are distinctly different from the facular-like (claw-like) unguals of *Claenodon*. The unguals of *Ectoconus* are like *Periptychus* in that they also exhibit a median fissure, but they are more mediolaterally compressed to give an almost faculate-like profile. The unguals of *Pantolambda bathmodon* are mediolaterally broad and dorsopalmarly deep, terminating in a pointed apex without a median fissure.

#### Innominate

**Fig 32:**
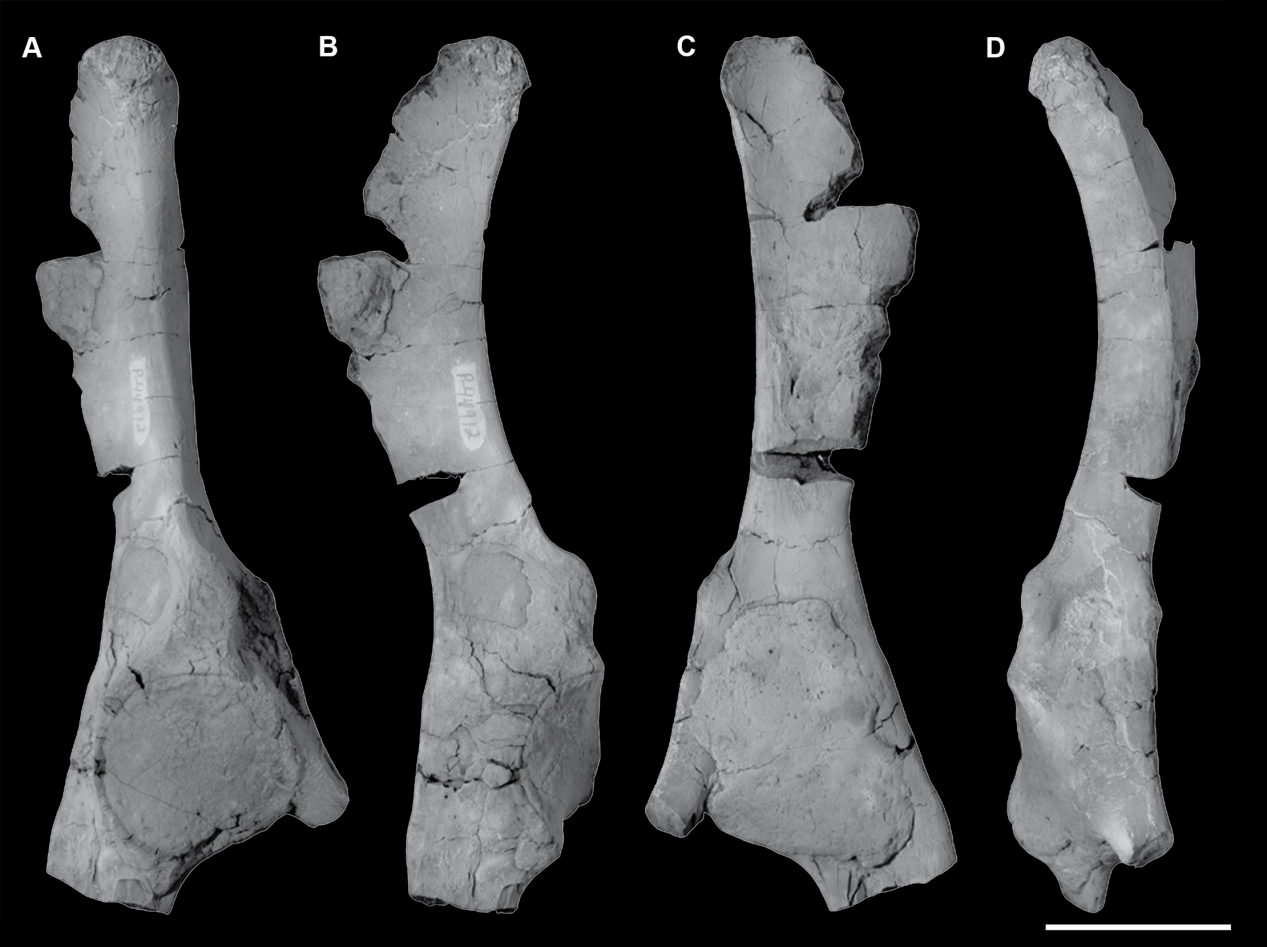
Innominate of *Periptychus carinidens* (NMMNH P-44917). (A) lateral view; (B) dorsal view; (C) medial view; (D) ventral view. Scale bar: 30mm.

**Fig 33:**
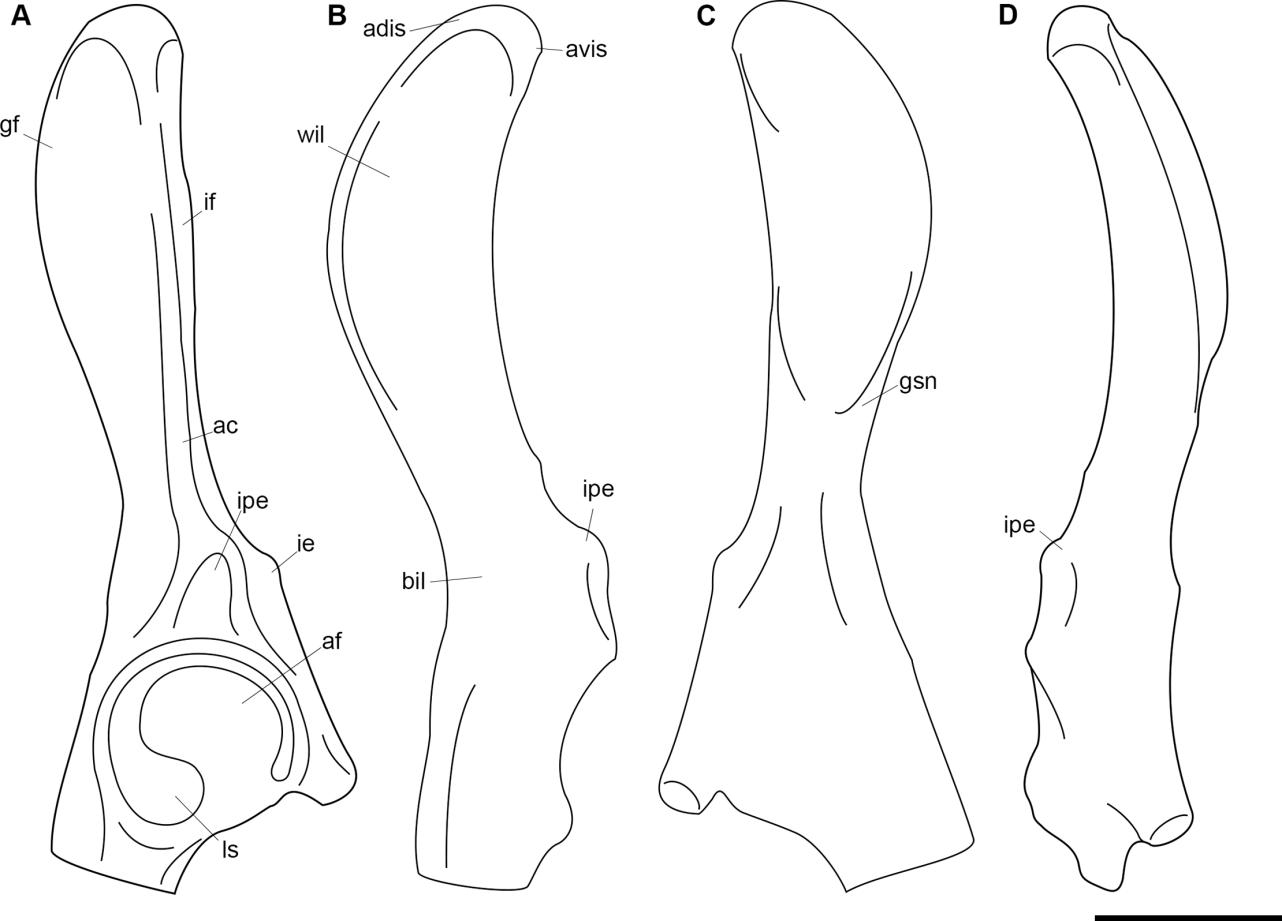
Annotated line drawing of the innominate of *Periptychus carinidens*. (A) lateral view; (B) dorsal view; (C) medial view; (D) ventral view. Abbreviations: ac, acetabular crest; adis, anterodorsal iliac spine; af, acetabular fossa; avis, anteroventral iliac spine; bil, body of the ilium; gf, gluteal fossa; gsn, greater ischiatic notch; if, iliacus fossa; ie, iliopubic eminence; ipe, iliopectineal eminence; ls, lunate surface; wil, wing of the ilium. Scale bar: 30mm.

The innominate of *Periptychus* is not well known, and a complete os coxa has yet to be discovered. NMMNH P-35194 is a near complete skeleton that includes a portion of the right innominate consisting of the ilium, ischium and pubis surrounding the acetabulum; the acetabulum is present, but highly concreted. NMMNH P-44912 is a referred right innominate of *Periptychus* preserving most of the ilium and portions of the ischium and pubis surrounding the acetabulum; the acetabulum has been infilled during preparation (Figs 32 and 33). NMMNH P-30651 is a referred right innominate of *Periptychus* preserving the acetabulum region. NMMNH P-44192 and NMMNH P-30651 are both from Torrejonian aged deposits from the Nacimiento Formation and have been referred to *Periptychus* based on their size and close resemblance to NMMNH P-35194. Comparisons are made to *Ectoconus ditrigonus* (AMNH 3799), *Pantolambda bathmodon* (AMNH 16663, figured in [5]), and *Arctocyon primaevus* (MNHN.F.CR69, CR70, BR17472 and BR12711 figured in [52]).

The innominate of *Periptychus* is broadly similar to *Ectoconus*, *Arctocyon* and *Pantolambda* in its overall morphology, but differs somewhat in its proportions. The ilium of *Periptychus* is slender and elongate. In dorsal aspect, it is concave medially. The ilium of *Periptychus* is not as robust or transversely broad as that of *Ectoconus* and *Arctocyon*, nor as deep as that of *Pantolambda*.

In *Periptychus*, the wing of the ilium is dominated by the gluteal fossa for the attachment of the gluteus maximus and gluteus medius. This fossa forms a smooth, flat, triangular surface. In *Periptychus*, the wing of the ilium gradually expands away from the body of the ilium, whereas in *Ectoconus* and *Arctocyon* the wing of the ilium flares rapidly, creating a notch on the ventral border of the innominate. Anteriorly, the gluteal fossa of *Periptychus* becomes transversely broader and faces dorsolaterally. In *Arctocyon* and apparently in *Ectoconus*, the gluteal fossa is much more transversely expanded, forming a large triangular shelf that is fully visible in lateral view. In contrast to this, in *Periptychus* and *Pantolambda*, the gluteal fossa faces more dorsolaterally and is not fully visible in lateral view. The iliac fossa of *Periptychus* is positioned adjacent to the gluteal fossa and is separated from it by the acetabular crest. The acetabular crest of *Periptychus* (and also *Ectoconus*) is transversely broad and round, rather than a sharp crest as in *Arctocyon* and *Pantolambda*. In *Periptychus*, the iliac fossa is transversely narrow (less than half the width of the gluteal fossa at its narrowest part) and faces ventrolaterally.

The medial surface of the ilium features a large fossa on its anterior portion for the attachment of the erector spineae and sacrospinalis muscles. The iliac neck is anteroposteriorly elongate and only slightly narrower than the body of the ilium, so that there is no clear distinction between the body and neck. *Ectoconus*, *Arctocyon* and *Pantolambda* also possess a proportionally elongate iliac neck, but in these taxa, there is a clearer distinction between the body and neck due to the expansion of the ilium wing and gluteal fossa.

In dorsal aspect, the iliac spine of *Periptychus* extends around the medial edge of the wing of the ilium. The anteroventral portion of the iliac spine is inflected ventrolaterally relative to the posterodorsal portion of the spine. The iliac spine morphology of *Periptychus* is well defined, but it is not as exaggerated as the condition in *Arctocyon* or *Pantolambda*. In *Pantolambda*, the anteroventral portion of the iliac spine is proportionally much larger, with a large muscle attachment area for the origin of the sartorius muscle, and is greatly inflected so that the anterior edge of the ilium is positioned at 90° to the dorsal edge of the bone. The posterodorsal extent of the iliac spine is marked by the greater ischiatic notch, which also demarcates the border between the wing and the body of the ilium. In *Periptychus*, this notch is small and barely interrupts the medial margin of the ilium, whereas in *Arctocyon* and *Pantolambda* the notch forms a distinct incision approximately four fifths along the longitudinal length of the bone.

In dorsal view, the medial border of the ilium and ischium of *Periptychus* form an obtuse angle (170°) relative to one another. This morphology is not evident in *Arctocyon* or *Pantolambda*, where the ilium and ischium sit along the same parasagittal plane. The posterior half of the innominate of *Periptychus* is incompletely known. The ischium and pubis are represented by specimens showing their contributions to the acetabulum, but their posterior portions and how they form the obturator foramen remain unknown.

The cup-shaped acetabulum of *Periptychus* is formed by the ilium, ischium and pubis; however, the individual contributions of each bone are not discernible on the specimens available. The border of the acetabulum opens posteriorly so that the walls of the socket only circumnavigate four fifths of the perimeter. The posteriorly open portion of the acetabular socket forms the acetabular notch. In *Periptychus*, the acetabular notch is deep so that the posterior border of the acetabulum is situated well within the body of the innominate, rather than the posterior opening directly bordering the obturator foramen as seen in *Arctocyon*. The acetabulum of *Periptychus* is notably different from the condition of *Ectoconus*, in that the acetabulum of *Ectoconus* is more widely open posteriorly and the acetabular notch is not as deep, so that socket of the acetabulum opens directly into the obturator foramen. In *Periptychus*, the acetabular notch is continuous with the acetabular fossa (a circular depression positioned within the centre of the socket of the acetabulum).

The border of the acetabulum is bounded by a prominent rim for attachment of the acetabular labrum. The walls of the acetabulum between the rim and the acetabular fossa are formed by the lunate surface, which articulated with the head of the femur. The lunate surface is continuous, with each end forming a distinct lobe. The dorsal lobe is situated within the ischiatic contribution to the acetabulum; it is larger than the ventral lobe, pyriform in shape and well distinguished from the acetabular fossa. The ventral lobe is much smaller, situated within the pubic contribution to the acetabulum and is not as well distinguished from the acetabular fossa as the dorsal lunate lobe. The condition of *Periptychus* closely resembles that of *Ectoconus*, but differs from *Arctocyon*. In *Arctocyon*, the two lobes appear much closer in size; however, the preservation of the specimens observed does limit the reliability of this comparison.

The iliopectineal tuberosity of *Periptychus* is proportionally massive and lies just anterior to the acetabulum along the acetabular crest. It forms an anteroposteriorly elongate prominence with a deeply scarred surface, which provides a large attachment area for the origin of the rectus femoris muscle. The iliopectineal tuberosity of *Periptychus* is proportionally comparable to that of *Ectoconus* and more massive than that of *Arctocyon* and *Pantolambda* in lateral view. However, in dorsal aspect, *Pantolambda* exhibits a substantially more prominent tuberosity than *Periptychus* and the other comparison taxa.

In *Periptychus*, a well-developed iliopubic eminence is present anteroventral to the acetabulum, and ventral to the iliopectineal tuberosity. The iliopubic eminence marks the dorsal border between the ilium and pubis, provides an attachment area for the insertion of the psoas minor, and serves to guide the iliacus and psoas major muscles towards the lesser trochanter on the femur. The eminence of *Periptychus* is notably more developed than that of *Ectoconus* and *Arctocyon*, and positioned further anteriorly, well beyond the anterior border of the acetabulum. This implies an elongate pubic bone or shortened ilium along the ventral border.

The ischium and pubis are poorly known for *Periptychus*. Based on what is known of these bones, the ischium is transversely broader than the pubis, as also seen in *Arctocyon* and *Pantolambda*. However, the pubis of *Periptychus* appears to be more proportionally robust than in either of the comparison taxa.

#### Femur

**Fig 34:**
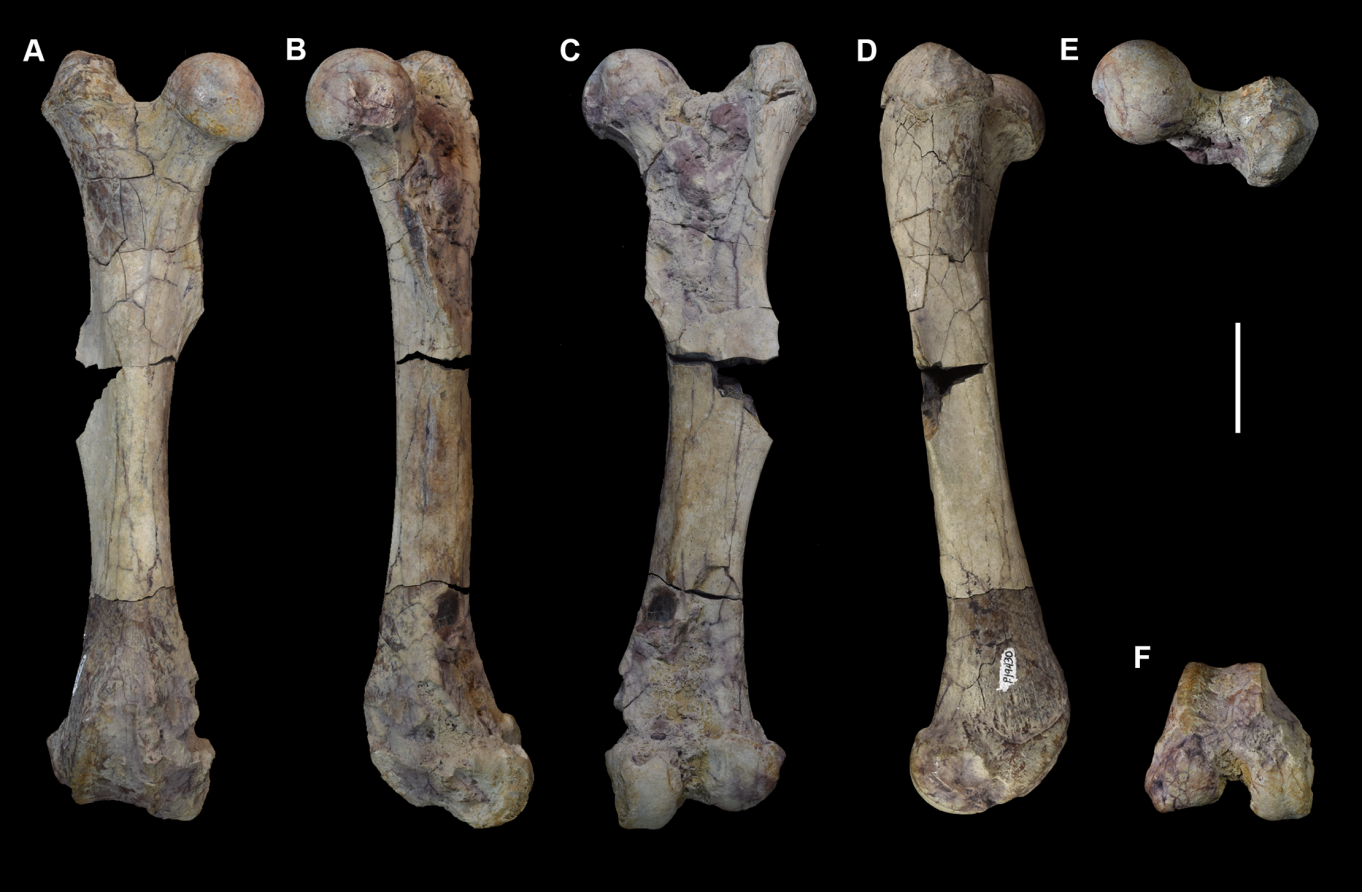
Right femur of *Periptychus carinidens* (NMMNH P-19430). (A) anterior view; (B) medial view; (C) posterior view; (D) lateral view; (E) proximal view, (F) distal view. Scale bar: 30mm.

The description of the femur of *Periptychus* is based on an assortment of specimens that allow for a comprehensive description of the anatomy. NMMNH P-19430 includes a well preserved and near complete right femur and associated patella (Fig 34). The posterior surface of the bone retains some concreted matrix, the lesser trochanter and third trochanter are missing and the medial epicondyle exhibits some light damage. NMMNH P-35194 includes portions of the left and right femora and the left patella. The right femur preserves the proximal epiphysis and diaphysis, but is missing the distal epiphysis. The left femur is known only from the diaphysis and both femora are lightly concreted. AMNH 837 is a complete right femur; the lesser trochanter is missing but the specimen is otherwise well preserved. AMNH 3637 is a fragmentary skeleton of a juvenile individual preserving a near complete right femur. The specimen is generally well preserved, but the femoral head, part of the greater trochanter, the third trochanter and part of the trochlear/distal epiphysis are missing and have been reconstructed. Comparisons are made to *Ectoconus ditrigonus* (AMNH 16500, figured in [5]), *Mithrandir gillianus* (NMMNH P-3083, figured in [48]), *Arctocyon primaevus* (MNHN.F.CR17, CR16 figured in [52]) and *Pantolambda bathmodon* (AMNH 16663, figured in [5]).

**Fig 35:**
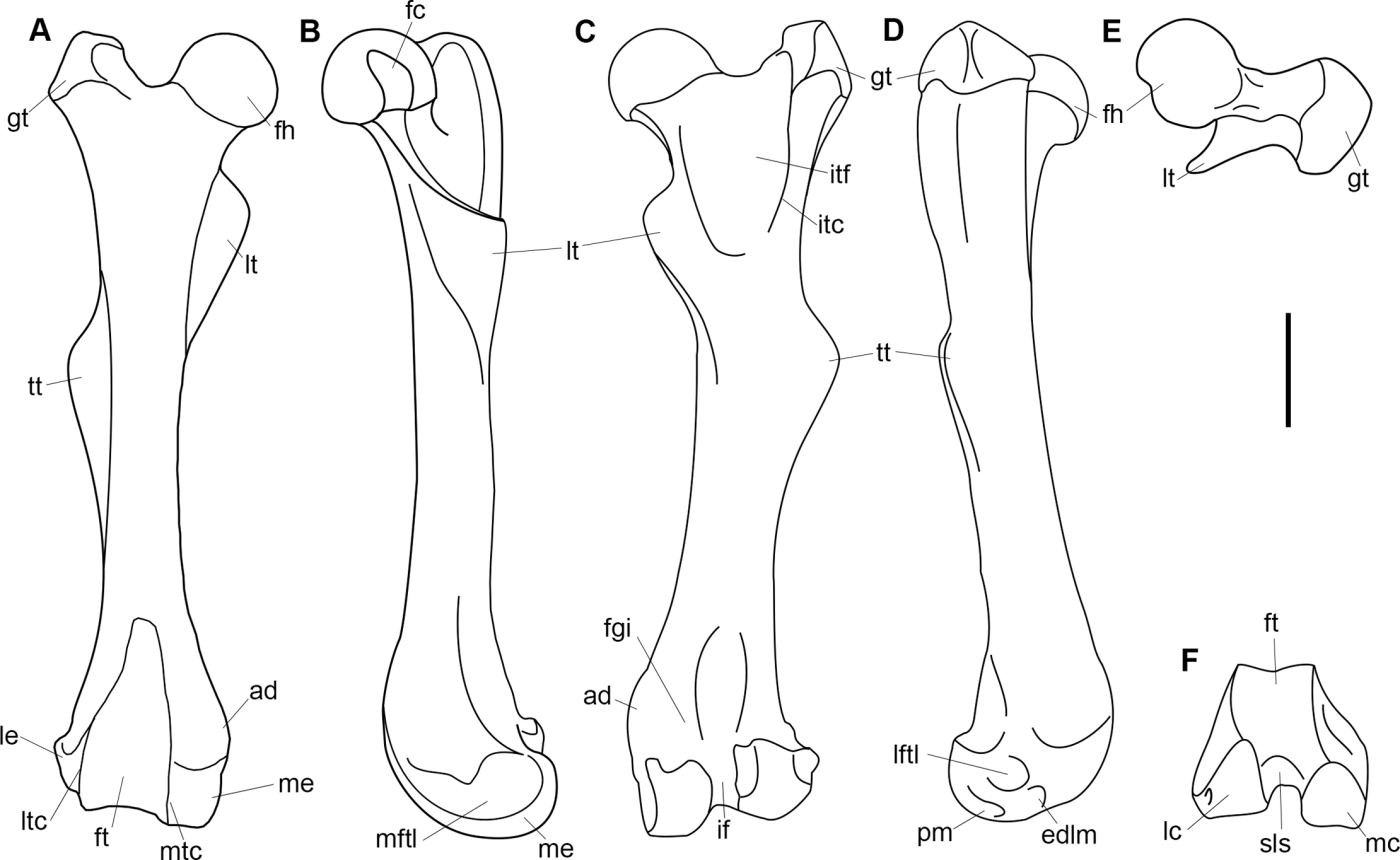
Annotated line drawing of the right femur of *Periptychus carinidens*. (A) anterior view; (B) medial view; (C) posterior view; (D) lateral view; (E) proximal view; (F) distal view. Abbreviations: edlm, fossa for the attachment of the extensor digitorum longus muscle; fc, fovea capitis; fgi, fossa for the gastrocnemius internus; fh, femoral head ft, femoral trochlea; gt, greater trochanter; lc lateral condyle; le, lateral epicondyle; if, intercondylar fossa; lftl, fossa for attachment of the lateral femorotibial ligament; lt, lesser trochanter; ltc, lateral trochlear crest; itc, intertrochanteric crest; itf, trochanteric fossa; mc, medial condyle; me, medial epicondyle; mftl, fossa for attachment of the medial femorotibial ligament; mtc, medial trochlear crest; pm, fossa for the attachment of the popliteus muscle, sls, semi-lunar surface; tt, third trochanter. Scale bars: 30mm.

The femur of *Periptychus* is characteristic of a stoutly built animal: the bone is robust, with mediolaterally broad proximal and distal extremities and well-developed muscle attachment sites (Fig 35). The proximal epiphysis is approximately 25% broader than the distal epiphysis. The femoral head is large and hemispherical in shape, forming approximately two thirds of a sphere. The articular surface of the head is smooth and highly convex, with a small lateral extension onto the femoral neck towards the greater trochanter. The morphology of *Periptychus* is like that of *Ectoconus* and *Mithrandir*, although in *Ectoconus* the articular surface does not extend onto the femoral neck. In *Arctocyon*, the articular surface forms a larger extension onto the neck towards the greater trochanter than in *Periptychus*. In *Pantolambda*, the articular surface of the femoral head is relatively reduced in comparison to *Periptychus*, forming little more than half a sphere with no lateral extension onto the femoral neck.

In *Periptychus*, a well-developed, deep fovea capitis excavates the posteromedial articular surface of the femoral head, which served as an attachment point for the ligamentum teres. It is ovoid in shape and positioned within a triangular shaped depression, which extends distally to interrupt the posteromedial rim of the femoral head. A fovea capitis is also evident in *Ectoconus*, *Mithrandir*, *Arctocyon* and *Pantolambda*; however, it is not as large as in *Periptychus* and does not interrupt the rim of the femoral head in any of the comparison taxa. In lateral aspect, the femoral head of *Periptychus* inflects anteriorly so that the proximodistal axis of the head is orientated at approximately 20° from the long axis of the diaphysis. *Ectoconus* and *Mithrandir* exhibit a similar level of anterior inflexion of the head as *Periptychus*, whereas in *Arctocyon* and *Pantolambda* the head is only slightly anteriorly inflected (~10°).

The femoral neck of *Periptychus* is robust and relatively elongate, and it nearly approximates the head in proximodistal height. The morphology of the femoral neck of *Periptychus* is broadly similar to that in *Ectoconus* and *Pantolambda*, but is not as proportionally elongate as in *Mithrandir*. The neck of *Arctocyon* is proportionally more robust, but not as elongate as in *Periptychus* or any of the comparison taxa. In *Periptychus*, the femoral head is distinguished from the neck by a distinct rim (although not as well-expressed as in *Arctocyon*) and the neck clearly separates the head from the body of the proximal epiphysis. In anterior aspect, the neck and head of *Periptychus* are orientated at approximately 150° to the proximodistal long axis of the femur (this is also observed in all the comparison taxa).

The greater trochanter of *Periptychus* is massive, pyramidal in shape and substantially separated from the femoral head, but projects only slightly superior to the femoral head (by a few millimetres). The ridge separating the greater trochanter from the head forms a straight, anteroposteriorly thick bridge-like structure. In *Ectoconus* and *Pantolambda*, the greater trochanter is similar in morphology to *Periptychus* and is similarly subequal to slightly taller than the femoral head, whereas in *Mithrandir* the trochanter, although very similar in morphology to that in *Periptychus*, *Ectoconus* and *Pantolambda*, is substantially taller than the head. The condition of *Periptychus* differs from that of *Arctocyon*, where the greater trochanter is much more rounded in morphology and remains distal to the femoral head with a less developed apex insertion site for the gluteus medius when compared to *Periptychus*.

In anterior aspect, the greater trochanter of *Periptychus* is mediolaterally broad and subequal to the femoral neck in mediolateral width. The head of the greater trochanter is subdivided into three distinct surfaces, which are sculpted by several muscle attachment areas: the gluteus medius inserted at the apex of the greater trochanter (this insertion site is large and proximally prominent in *Periptychus*), the gluteus profundus likely inserted at a well-defined, scarred area on the anterior surface of the greater trochanter, and the piriformis inserted at a relatively large and deep, subpyriform shaped facet on the posteroproximal surface of the greater trochanter (proximal to the intertrochanteric crest). The muscle attachment sites are well defined in the specimens of *Periptychus* observed, much more so than in the comparison taxa (particularly *Pantolambda*, which is of a similar size and build). The insertion site for the piriformis muscle is remarkably large in comparison to the other Paleocene taxa observed.

The posterior surface of the proximal epiphysis of *Periptychus* is deeply excavated by the intertrochanteric fossa (= digital fossa). The fossa is deep, with its lateral border delimited by the greater trochanter and a well-developed intertrochanteric crest. The condition of *Periptychus* is like that of *Ectoconus* and *Arctocyon*, all of which exhibit a more deeply excavated fossa than *Pantolambda*, but not as deeply excavated as the condition in *Mithrandir*. The intertrochanteric fossa of *Periptychus* is proximodistally elongate, occupying approximately 33% of the proximodistal length of the femur. The intertrochanteric fossae of *Ectoconus* and *Pantolambda* are slightly shorter (28% and 27% respectively) and that of *Arctocyon* is notably shorter (25%).

In *Periptychus*, the lateral component of the intertrochanteric fossa forms a sharp, well-developed ridge. The ridge descends the femur distally providing attachment areas for the insertion of the internal and external obturator and the superior and inferior gemelli muscles along an elongate depression formed on the medial wall of the greater trochanter, where the intertrochanteric crest overhangs the fossa. The medial component of the intertrochanteric crest is not well defined towards the lesser trochanter, and as such the distal border of the intertrochanteric fossa is poorly delimited from the femoral diaphysis. The linea quadrata, which provided attachment for the quadratus femoris, is not evident on any of the specimens of *Periptychus* observed. In this respect, *Periptychus* is like *Pantolambda*: for both taxa the distal border of the intertrochanteric fossa is poorly defined from the femoral diaphysis. In *Ectoconus* and *Mithrandir*, the distal border is somewhat more defined by the intertrochanteric crest. In *Arctocyon*, the borders of the fossa are very clearly defined, and the posterior border is demarcated by a large rugose area for attachment of the quadratus femoris and a distinct linea quadrata.

In *Periptychus*, the intertrochanteric line is not well-defined on the anterior surface of the proximal epiphysis. Instead, a subtle ridge extends proximomedially from the greater trochanter to the lesser trochanter. Despite the poor definition of the ridge, *Periptychus* exhibits the most pronounced intertrochanteric line among the comparative taxa. In *Pantolambda* there is no evidence of an intertrochanteric line and in *Ectoconus*, *Mithrandir* and *Arctocyon* the line is even less pronounced than in *Periptychus*.

In *Periptychus*, the lesser trochanter is positioned on the medial surface of the proximal femur diaphysis, forming an anteroposteriorly flattened triangular (almost blade-like) flange for the insertion of the iliopsoas muscle. The flange is large and projects posteromedially away from the femoral diaphysis, and its proximal edge does not form a continuous shelf that extends to the femoral head. Its posterior edge forms a well-defined and subtly rounded pectineal line for the insertion of the pectineus muscle. The lesser trochanter of *Periptychus* is broadly like *Pantolambda* in shape, size and position on the femur; however, in *Pantolambda* it projects more posteriorly, and the pectineal line is robust but does not form a distinct crest. The condition of *Periptychus* is not as developed as in *Ectoconus*, where the lesser trochanter is proportionally more massive, forming a larger posteromedial projection. The condition of *Periptychus*, *Ectoconus* and *Pantolambda* is very different from that of *Arctocyon*. In *Arctocyon*, the lesser trochanter is not as triangular in shape, its pectineal line is sharp and its medial apex is more rounded, and the whole flange is directed more medially than posteromedially as in *Periptychus*. Further to this, in *Arctocyon*, the lesser trochanter extends further proximally so that it almost reaches the femoral head, and the edge of the flange inflects anteriorly providing a very large attachment area for the iliopsoas muscle.

The third trochanter of *Periptychus* is positioned distal to the lesser trochanter on the lateral side of the femoral diaphysis, approximately 40% down the length of the shaft. It forms an extensive, triangular shaped shelf that projects laterally away from the femoral diaphysis and provided a large attachment area for the insertion of the gluteus superficialis muscle. The condition of *Periptychus* is not as developed as in *Ectoconus*, where the third trochanter forms a proximodistally extensive shelf that extends along nearly the entire length of the lateral femoral diaphysis to the distal epiphysis. *Periptychus* more closely resembles the condition in *Pantolambda*, where the third trochanter also forms a discrete, laterally directed flange; however, in *Pantolambda* the third trochanter is positioned slightly more distal on the femoral diaphysis. The condition of *Periptychus* is very different from *Arctocyon*, where the third trochanter is comparatively small and is positioned more proximally on the diaphysis, so that at its mediolaterally widest point it is almost level to the lesser trochanter.

The femoral diaphysis of *Periptychus* is robust and anteroposteriorly compressed, rendering the transverse cross section of the shaft sub-triangular in shape. *Ectoconus* and *Mithrandir* exhibit a similar condition. In *Periptychus*, the shaft is straight in both lateral and anterior aspect, but the mediolateral axis of the proximal epiphysis is skewed relative to the mediolateral axis of the distal epiphysis. In proximal view, the mediolateral axis of the femoral head and greater trochanter is set at approximately 35° relative to the mediolateral axis of the distal epiphysis and diaphysis. A similar condition characterizes *Ectoconus*, but it is much less pronounced in *Arctocyon* and *Pantolambda*.

The anterior surface of the femoral diaphysis of *Periptychus* is smooth. There is very little scarring from the vastus medius, vastus intermedius and vastus lateralis of the quadriceps femoris muscle. The posterior surface of the femoral diaphysis exhibits a mediolaterally broad linea aspera formed by three ridges. The linea aspera is distinguishable from the diaphysis, but it is not strongly elevated from the bone shaft. The lateral ridge of the linea aspera is formed in conjunction with the third trochanter, and extends almost vertically upward to the base of the greater trochanter; it provided attachment for the gluteus maximus and vastus lateralis. The intermediate ridge is broadly rounded and slightly raised proximally; it is continuous with the pectineal line at the base of the lesser trochanter. The medial ridge is discontinuous along its proximodistal length but is visible extending along the medial side of the femoral diaphysis towards the anterior surface of the lesser trochanter. It would have provided attachment for the origin of the vastus medialis. The surface between the intermediate and medial ridge of the linea aspera provided attachment to a portion of the iliacus at the base of the lesser trochanter. The raised surface of the linea aspera is marked by light scarring for the attachment of the adductor muscles and the biceps femoris muscles.

Distally, the linea aspera diverges into the lateral supracondylar line and the medial supracondylar line, enclosing between them the popliteal surface. The lateral supracondylar line is prominent, forming a broad ridge that descends the distal diaphysis to the summit of the lateral condyle. It provided attachment for the plantaris muscle along its length. A distinct ovoid fossa is positioned lateral to the distal end of the lateral supracondylar line and was a well-developed attachment site for the lateral gastrocnemius muscle. The medial supracondylar line is less distinct than the lateral supracondylar line. The distal portion of the medial supracondylar line forms the medial edge of a posteriorly directed surface, which served as an attachment area for the medial gastrocnemius. A prominent adductor tubercle is positioned on the distomedial edge of the femoral diaphysis, just proximal of the medial condyle. The adductor tubercle forms a proximodistally elongate semi-circular flange, rather than a protuberance or tubercle, and provided a large insertion site for the tendon of the adductor magnus. The adductor tubercle of *Periptychus* is similar in its elongate, semi-circular morphology to that of *Ectoconus* and *Arctocyon*, but differs from the condition in *Mithrandir*, where the adductor forms a small protuberance rather than a flange.

The muscle attachments on the posterior surface of the femoral diaphysis of *Periptychus* are subtle, but are notably more developed and more clearly delimited than in *Pantolambda*. In *Pantolambda*, the linea aspera is barely evident and the popliteal surface is poorly defined from the diaphysis (note that although the femur is well preserved, the lack of clearly defined muscle attachments could be an artefact of preservation). The condition of *Periptychus* is markedly different from that of *Arctocyon*. Overall, the muscle attachment sites of *Periptychus* are proportionally larger than those of *Arctocyon*; however, in *Arctocyon* the muscle attachment sites tend to be more prominent. More specifically, in *Periptychus* the linea aspera is mediolaterally broader than in *Arctocyon*; the popliteal surface is well-defined in both taxa, but in *Periptychus* it is proximodistally longer; the adductor tubercle of *Periptychus* is proportionally proximodistally longer than in *Arctocyon*, but it is not as prominent and does not protrude as far medially.

In *Periptychus*, the distal epiphysis of the femur is slightly deeper anteroposteriorly than it is mediolaterally wide, giving it a cuboid profile. The femoral condyles form two oblong eminences that project posteroexternally from the posterior surface of the distal epiphysis, separated from each other by the intercondylar fossa of femur. The proportions of the distal epiphysis of *Periptychus* differ from *Mithrandir*, *Arctocyon* and *Pantolambda*, where the distal epiphyses are marginally mediolaterally wider than anteroposteriorly deep, rather than subequal as in *Periptychus*.

The anterior side of the distal epiphysis of *Periptychus* is dominated by the patellar surface, which forms a pronounced femoral trochlea. The trochlea is a deep and mediolaterally broad groove with sharp trochlear crests, and is slightly elevated relative to the surrounding bone of the distal epiphysis. The morphology of the femoral trochlea of *Periptychus* is generally like that of *Ectoconus*, *Mithrandir*, *Arctocyon* and *Pantolambda* in that the patellar surface forms a broad and elevated trochlea. The condition of *Periptychus* is not as elevated as in *Ectoconus* or *Arctocyon*, but is more deeply incised. Furthermore, the condition of *Periptychus* is not as mediolaterally broad as in *Arctocyon* (where the trochlea crests are particularly sharp).

The trochlear groove of *Periptychus* is elongate; it extends from the distal surface of the distal epiphysis, where it does not quite contact the intercondylar fossa (separated by the semi-lunar surface), proximally to a point that is proximal to the proximal surface of the femoral condyles. The lateral and medial borders of the trochlea of *Periptychus* are broadly parallel to the longitudinal axis of the femur. The proximal border of the trochlea is marked by a secondary fossa, which is near continuous with the trochlea. Proximally, the secondary fossa tapers to a rounded apex, which is positioned well onto the femoral diaphysis (approximately one quarter of the way up the femur). This proximal extension of the patellar surface in *Periptychus* is also evident in *Ectoconus*, *Mithrandir* and *Arctocyon*, but not in *Pantolambda*. The condition of *Pantolambda* differs in that the femoral trochlea is relatively short and lacks a distinct secondary fossa, with the patellar surface terminating at a point that is approximately level with the proximal surface of the femoral condyles. The condition of *Arctocyon* is also notably distinct from that of *Periptychus* in that the femoral trochlea is quite short, but the secondary fossa is well developed, forming a rounded facet that is well separated from the femoral trochlea.

In anterior aspect, the femoral trochlea of *Periptychus* is asymmetrical: the distal border of its medial rim is mediolaterally broader and projects further distally than the lateral rim, and the medial wall of the medial rim forms a gentle slope rather than a steep wall. The condition of *Periptychus* is broadly like that of *Arctocyon* in that the medial rim is mediolaterally broader and projects distally relative to the lateral rim. The morphology in *Periptychus* (and *Arctocyon*) is subtly different to that in *Pantolambda*, where the medial rim is more rounded than the lateral rim (as in *Periptychus*), but the lateral rim is much sharper in comparison to *Periptychus* and the profile of the lateral border of the trochlear groove is set at an oblique angle to the longitudinal axis of the femur rather than parallel to the longitudinal axis as in *Periptychus*. In distal aspect, the femoral trochlea of *Periptychus* is near symmetrical, the internal walls of the trochlea broadly mirror each other and the anterior apices of the trochlea rims are subequal in height. This morphology is also present in *Mithrandir*, but differs from that of *Arctocyon*, where the medial rim of the trochlea groove projects more anteriorly than the lateral rim, forming a steeper medial trochlea wall.

The medial and lateral epicondyles of the femur of *Periptychus* form relatively flat walls between the patellar surface anteriorly and the femoral condyles posteriorly. The medial epicondyle is expanded relative to the lateral epicondyle; its development is further exaggerated by the presence of the adductor tubercle. A roughened triangular shaped surface positioned posterolateral to the adductor tubercle (on the posteroproximal surface of the femoral diaphysis) provided attachment for the origin of the medial head of the gastrocnemius. The surface of the medial epicondyle is marked by a small, but deep fossa for the attachment of the medial femorotibial ligament (= medial collateral ligament). The fossa is formed by two interconnected depressions that demarcate an open ‘u’ shaped attachment site on the posterodistal portion of the medial epicondyle. This fossa is clearly defined in *Periptychus*, but is not as pronounced as in *Arctocyon* or *Pantolambda*. In *Arctocyon* the fossa is proportionally larger and exhibits a similar interconnected morphology to *Periptychus*, whereas in *Pantolambda* the attachment site is formed by two separate, deep, ovoid depressions.

The lateral femoral epicondyle of *Periptychus* exhibits three distinct muscle attachment sites. The largest fossa is positioned proximally on the lateral epicondyle and provided a large, deep attachment area for the lateral femorotibial ligament (= lateral collateral ligament). Anterodistal to this fossa, bordering the articular surface of the condyle, is a smaller, round fossa for the extensor digitorum longus muscle. Posterior to this fossa, on the distal border of the epicondyle, is a small, elongate and deep fossa for the origin of the popliteus muscle. A small groove extends posteriorly from the fossa for the popliteus muscle, which likely held the popliteus muscle when the knee was fully flexed.

In anterior aspect, the femoral condyles of *Periptychus* are moderately prominent and can be seen projecting distally relative to the distal margin of the femoral trochlea. This condition is also evident in *Ectoconus* and to a lesser extent in *Arctocyon*, but is lacking in *Mithrandir* and *Pantolambda*. In distal aspect, the femoral condyles of *Periptychus* are well separated by the intercondylar fossa. Although the condyles of *Periptychus* are well spaced and form a mediolaterally broad distal epiphysis, they are not as broadly separated as in *Arctocyon* and *Mithrandir*, particularly with respect to the medial condyle, which does not project as far medially in *Periptychus* as in the aforementioned taxa.

Anteriorly on the distal surface of the distal epiphysis, both condyles are separated from the femoral trochlea by an oblique groove above each condyle. The intercondylar fossa and femoral trochlea are separated by a small semi-lunar area and slightly offset relative to one another, so that the longitudinal axis of the femoral trochlea is positioned laterally relative to the longitudinal axis of the intercondylar fossa. The lateral offset of the femoral trochlea in *Periptychus* is similar in degree to that of *Arctocyon*, but is considerably more pronounced in *Mithrandir* and *Pantolambda*.

The lateral femoral condyle of *Periptychus* is transversely broader than the medial condyle, but is not as prominent in its overall morphology. The surface of the lateral condyle is smooth and broadly rounded. In posterior aspect, the lateral condyle does not extend as far distally as the medial condyle. In this respect, the condition of *Periptychus* more closely approximates that of *Arctocyon* and *Pantolambda* rather than *Ectoconus* and *Mithrandir* (where the medial femoral condyle projects to approximately the same level as the lateral condyle/or only slightly less). In lateral aspect, the lateral condyle of *Periptychus* is not prominent and does not project posteriorly to form a shelf as the medial condyle does. In posterior aspect, the longitudinal axis of the lateral condyle is slightly offset at an oblique angle (<10°) relative to the longitudinal axis of the femoral diaphysis (proximolateral to distomedial). The condition in *Periptychus* is also evident in *Arctocyon*, but not in *Ectoconus*, *Pantolambda* or *Mithrandir*, where the longitudinal axis of the condyle is aligned with the longitudinal axis of the diaphysis. The medial face of the lateral condyle of *Periptychus* forms the medial wall of the intercondyloid fossa and is marked by a broad, smooth depression for the origin of the anterior cruciate ligament along its proximal portion.

The medial femoral condyle of *Periptychus* is well developed and more prominent that the lateral condyle. In posterior aspect, the medial condyle projects posteriorly from the femoral diaphysis, with its proximal surface forming a prominent shelf. The articular surface of the medial condyle is narrower than the lateral condyle, and it has well-defined lateral and medial edges. The articular surface of the medial condyle in *Periptychus* is relatively transversely flat and not transversely convex as seen in *Ectoconus*, *Mithrandir*, and *Pantolambda*; instead, it more closely resembles that of *Arctocyon*. Further to this, the surface of the medial condyle of *Periptychus* is marked a shallow longitudinal groove along its length. A similar groove is evident in *Arctocyon*, but not in any of the other comparison taxa. In posterior aspect, the longitudinal axis of the medial condyle is aligned with the longitudinal axis of the femoral diaphysis. The condition of *Periptychus* differs from all the comparison taxa, in which the longitudinal axis of the medial condyle forms an oblique (proximomedial to distolateral) angle relative to the longitudinal axis of the femoral diaphysis. Note that in *Arctocyon* both condyles are offset at the same angle relative to the body of the femur, whereas in *Ectoconus*, *Mithrandir* and *Pantolambda* only the medial condyle is offset. The lateral face of the lateral condyle forms the lateral wall of the intercondyloid fossa and features a shallow depression for the origin of the posterior cruciate ligament.

The intercondyloid fossa of *Periptychus* forms a deep longitudinal groove between the femoral condyles. The anterodistal end of the fossa is demarcated by the posterior margin of the patellar surface. The anterodistal portion of the fossa in *Periptychus* is not as deeply excavated as in *Arctocyon*, and forms a gentle gradient towards the posterior margin of the patellar surface. The posteroproximal border of the fossa of *Periptychus* is delimited by the intercondyloid line, which separates it from the popliteal surface.

#### Patella

**Fig 36:**
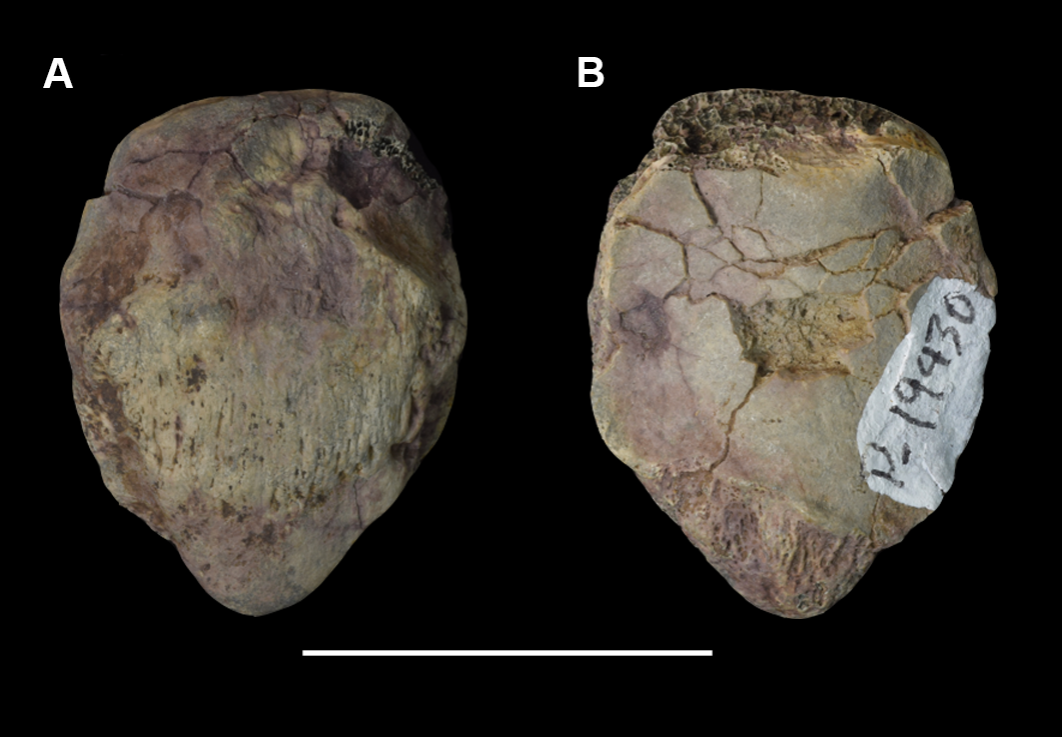
Patella of *Periptychus carinidens* (NMMNH P-19430). (A) anterior view; (B) posterior view. Scale bar: 30mm.

In anterior aspect, the patella of *Periptychus* is pyriform in profile, with a distal apex (Fig 36). The anterior surface of the patella is gently convex and features a raised bony section marked by longitudinal striations and perforated by numerous vascular canaliculi. The posterior surface has two distinct sections. This surface is smooth and saddle shaped, with the articular region subdivided into two facets divided by a shallow longitudinal ridge. The lateral facet is slightly larger. However, the medial side of the patella features a secondary facet that articulated with the medial condyle of the femur when in the knee was in extreme flexion. The external rim of the medial facet has a slight lip, which is not evident on the external rim of the lateral facet.

#### Tibia

**Fig 37:**
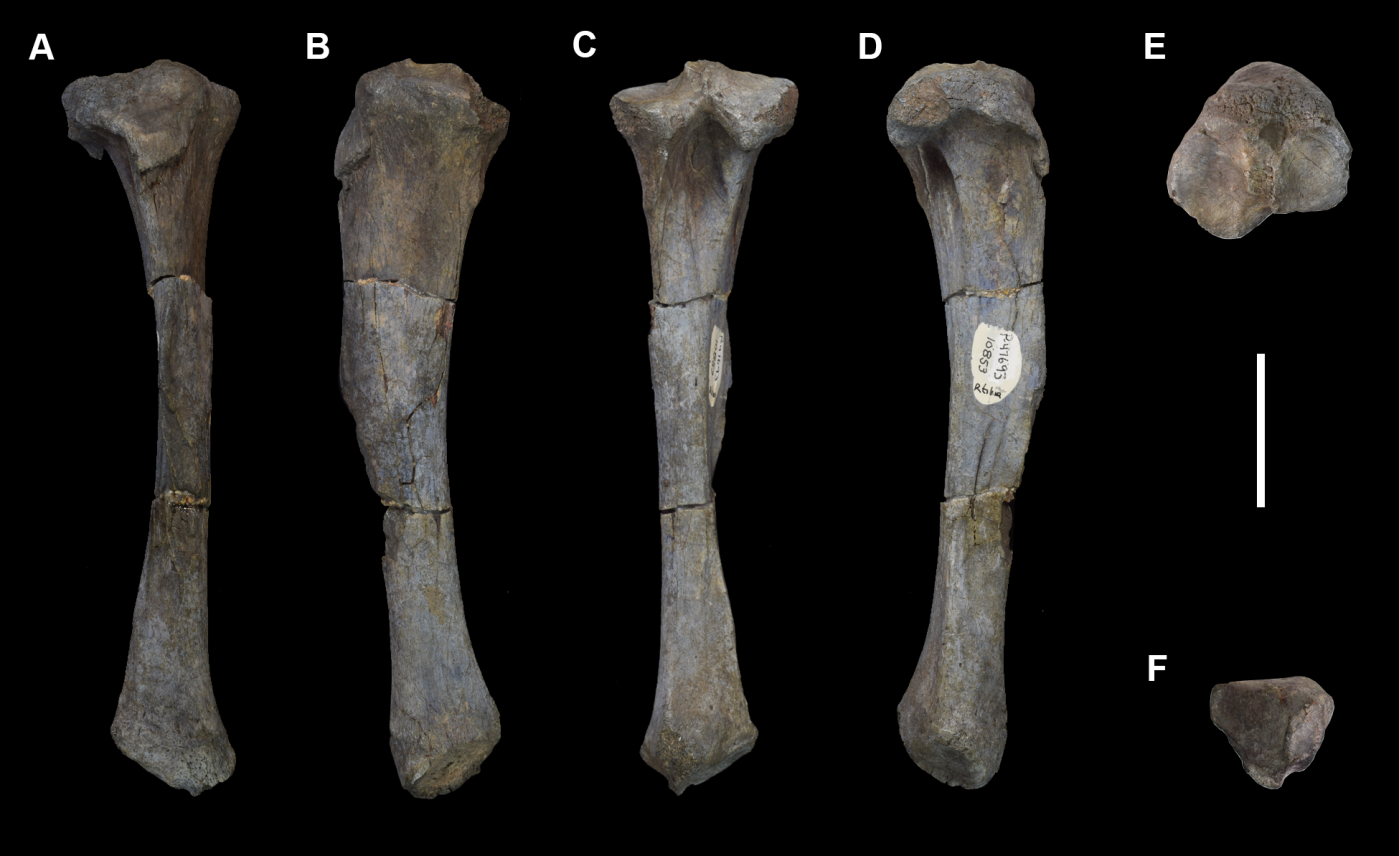
Right tibia of *Periptychus carinidens* (NMMNH P-47693). (A) anterior view; (B) medial view; (C) posterior view; (D) lateral view; (E) proximal view, (F) distal view. Scale bar: 30mm.

The tibia of *Periptychus* is known from an assortment of near complete specimens. NMMNH P-19430 includes a partial proximal right tibia with an associated fibula concreted to the shaft (displaced distally). The specimen is generally well-preserved, albeit slightly concreted. NMMNH P-47693 includes a well preserved, complete right tibia and a partial distal left tibia (Fig 37). The specimen is in good condition, as the bone is well-exposed and not concreted; however, the tibial crests on both bones are damaged and the distal epiphysis of the right tibia is missing the medial malleolus. NMMNH P-35194 includes the left and right tibiae; both tibiae are concreted, which limits the anatomical detail observable on this specimen. AMNH 837 includes a complete right tibia with an associated fibula, femur and tarsal bones. The tibia and fibula are fused and preserved in near perfect condition. Comparisons are made to *Carsioptychus coarctatus* (AMNH 27601), *Ectoconus ditrigonus* (AMNH 16500, figured in [5]), *Mithrandir gillianus* (NMMNH P-3083, figured in [48]), *Arctocyon primaevus* (MNHN.F.CR73 figured in [52]) and *Pantolambda bathmodon* (AMNH 16663, figured in [5]).

**Fig 38:**
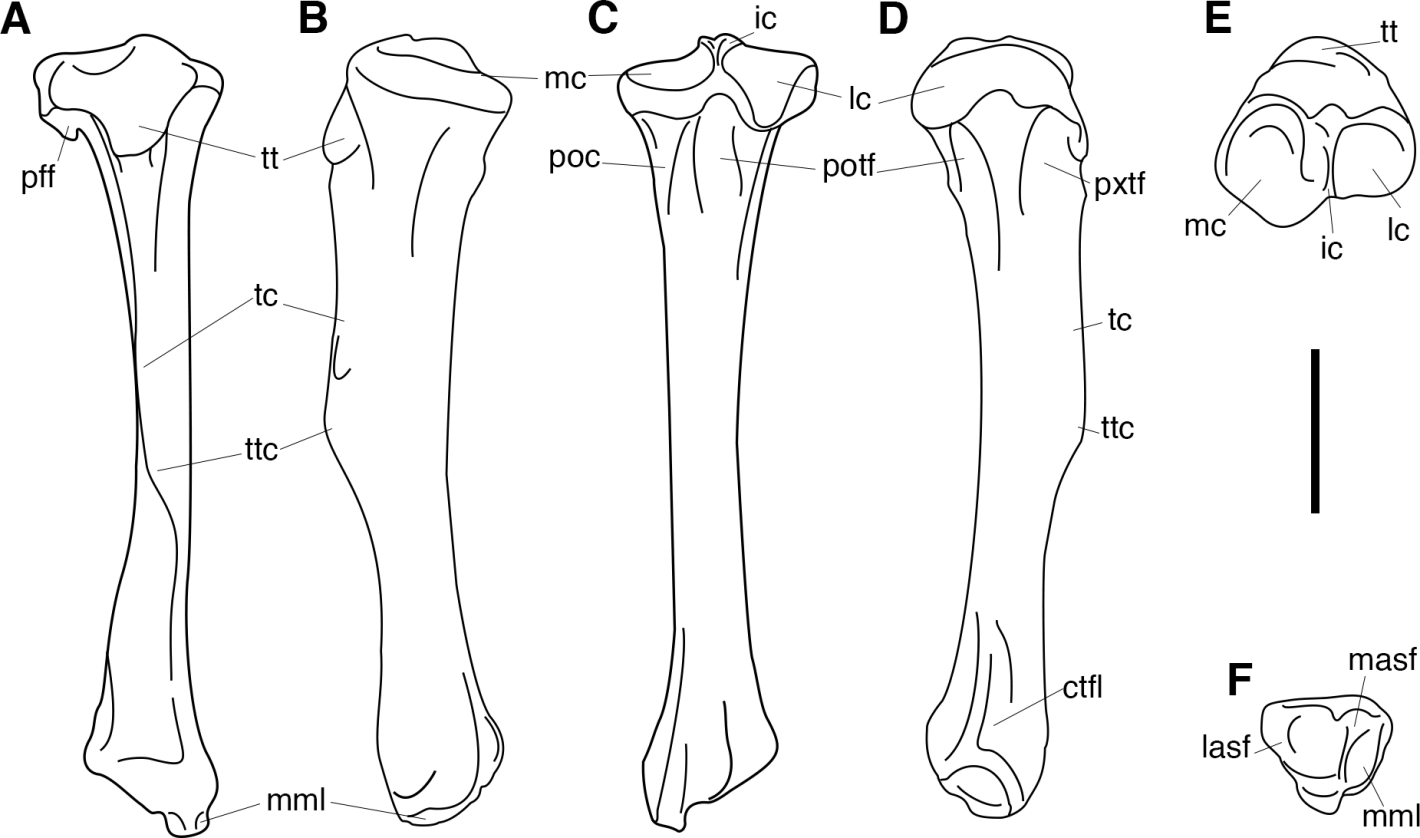
Annotated line drawing of the right tibia of *Periptychus carinidens*. (A) anterior view; (B) medial view; (C) posterior view; (D) lateral view; (E) proximal view; (F) distal view. Abbreviations: ctfl, crest for the tibiofibular ligament; ic, intercondyloid eminence; lasf, lateral astragalar facet; lc, lateral condyle, masf, medial astragalar facet; mc, medial condyle; mml, medial malleolus; pff, proximal fibular facet; poc, popliteus crest; potf; posterior tibial fossa; pxtf, proximal tibial fossa; tc, tibial crest; tt, tibial tuberosity; ttc, tuberosity of the tibial crest. Scale bar: 30mm.

The tibia of *Periptychus* is robust, with well-developed muscle attachment sites, but also proportionally elongate (Fig 38). Based on AMNH 837, the tibia is equivalent to 92% of the total length of the femur. This is proportionally longer than *Ectoconus* (82%), *Pantolambda* (85%) and *Arctocyon* (87%). The bone is relatively straight in both anterior and lateral views, although the proximal epiphysis deflects slightly lateral relative to the distal epiphysis, so that the profile of the bone is not as straight as that of *Arctocyon*. The profile of the tibia of *Periptychus* is straighter than that of *Carsioptychus* and *Pantolambda*, both of which exhibit distinct curvature along the bone length.

In proximal view, the proximal epiphysis of the tibia is subtriangular in shape and 15% mediolaterally wider than anteroposteriorly deep. Both *Arctocyon* and *Pantolambda* exhibit similar proportions to *Periptychus*. In *Arctocyon*, the proximal epiphysis is 10% wider than deep and in *Pantolambda* it is ~20% wider than deep. This contrasts with the condition of *Mithrandir*, where the mediolateral width and anteroposterior depth of the proximal epiphysis are subequal and the epiphysis exhibits a much sharper triangular profile.

The proximal articular surface of the tibia of *Periptychus* is massive. In lateral view, the proximal articular surface slopes posteriorly at approximately 15°. The medial tibial condyle is ovoid in proximal view (long axis orientated anteroposteriorly) and slightly larger than the lateral condyle, with a distinctly concave central depression. The external medial border of the medial condyle is thickened, forming a deep cuff around the condyle for the attachment of the medial collateral ligament. The internal lateral border of the medial tibial condyle extends into the medial intercondylar tubercle. The lateral condyle is more circular than ovoid in proximal view (with the long axis orientated anterolateral to posteromedially). It is smaller than the medial facet, with a near flat articular surface and a convex border. The external lateral border of the lateral condyle is thickened (as seen on the medial condyle) for the attachment of the lateral collateral ligament. The medial border of the lateral tibial condyle extends into the lateral intercondylar tubercle. The posterior border of the condyle is transversely broad and features a thick convex lip, which overhangs the proximal epiphysis for the cyamella of the popliteus. In life, the tendon of the popliteus muscle would have passed over this lip (and an associated tuberosity on the fibula) towards its insertion point on the diaphysis of the tibia. In anterior view, the surface of the lateral condyle is positioned more proximally relative to the medial condyle.

The morphology of the articular facets of *Periptychus* is broadly like the comparison taxa; however, the periptychids (*Periptychus*, *Carsioptychus*, *Ectoconus* and *Mithrandir*) exhibit more rounded tibial condyles than *Arctocyon*, in which the lateral condyle is more squared in proximal view. The attachment sites of the medial and lateral collateral ligaments of *Periptychus* are proportionally larger than those of *Pantolambda*, but not as developed as in *Ectoconus*.

The intercondylar eminence of *Periptychus* is formed by the medial and lateral intercondylar tubercles. The contributions from each tubercle are subequal in size and project to an equal height proximally. A distinct intercondyloid area separates the intercondylar tubercles. The surface of the intercondyloid area is positioned lower than the twin peaks of the intercondylar eminence, and broadens anteriorly and posteriorly to the intercondyloid eminence. *Arctocyon* and *Pantolambda* both exhibit a similar morphology to *Periptychus*. However, in *Arctocyon* and *Pantolambda*, the intercondylar tubercles and intercondylar area appear more subequal in level and are comparatively more developed, to form a taller eminence than in *Periptychus*. In *Periptychus*, the anterior intercondyloid area broadens anteriorly towards the tibial tuberosity and subdivides into two branches, which wrap around the anterior border of the medial and lateral tibial condyles. The anteromedial branch provided attachment for the medial meniscus. It is shallow and almost continuous with the medial tibial condyle (separated only by a low, broad ridge). The anterolateral branch is deeper, with a more pronounced border; it provided attachment for the lateral meniscus. The area between the two branches, just anterior of the intercondylar eminence, is distinctly roughened and provided attachment for the anterior cruciate ligament. The anterior extent of this depression (and the posterior extent of the tibial tuberosity) is demarcated by a small prominent tubercle. The morphology of the anterior intercondyloid area of *Periptychus* differs from *Arctocyon* in that the medial and lateral branches are more clearly differentiated from the tibial tuberosity and the tibial condyles. Furthermore, in *Arctocyon* the attachment area for the anterior cruciate ligament is proportionally larger than in *Periptychus*, and its anterior extent is delimited by a more prominent tubercle.

In *Periptychus*, the posterior intercondyloid area is near continuous with the posterolateral portion of the medial condyle. A small, subtle triangular facet extends posterodistally from the intercondyloid eminence/area and provided attachment to the medial meniscus. The posterior cruciate ligament attached to the smooth lip posterior to the triangular facet. The lateral meniscus attached to either side (anterior and posterior) of the medial tuberosity. The attachment sites for the lateral meniscus in *Periptychus* are markedly large in comparison to *Arctocyon*.

The tibial tuberosity of *Periptychus* is large and extends well onto the proximal surface of the proximal epiphysis. It provided a large attachment area for the insertion of the patellar tendon. In anterior view, the tuberosity is triangular, with its anteroposterior length equalling its mediolateral width. In lateral view, the tuberosity is prominent relative to the tibia diaphysis, forming a prominent anterior projection. It should be noted that there is some disparity in the morphology of the tibial tuberosity between specimens depending on size and age. On smaller individuals (i.e. NMMNH P-47693 and AMNH 837) the tuberosity is very prominent, with an undulating surface that is distinct from the tibial condyles. In larger individuals (i.e. NMMNH P-19430), the tibial tuberosity is heavily worn so that the surface is near flat and the proximal portion of the tuberosity is near continuous with the tibial condyles. Irrespective of this, the tibial tuberosity of *Periptychus* is more salient than the condition in *Pantolambda*. The morphology of the tibial tuberosity of *Periptychus* differs from that in *Carsioptychus*, *Ectoconus*, *Mithrandir* and *Arctocyon*. In *Carsioptychus* and *Ectoconus*, the surface of the tuberosity forms a less steep slope distally than in *Periptychus*, and as such the tuberosity in *Carsioptychus* and *Ectoconus* appears more prominent relative to the tibial diaphysis. In *Mithrandir*, the tibial tuberosity is proportionally small relative to the size of the tibial condyles, but is extremely prominent relative to the diaphysis, forming a similar slope to that seen in *Carsioptychus* and *Ectoconus*. In *Arctocyon*, the tibial tuberosity is more oblong in shape when viewed anteriorly and extends further onto the proximal surface of the proximal epiphysis than in *Periptychus*; it also appears more continuous (or less strongly demarcated) from the tibial condyles.

In *Periptychus*, the fibula facet is situated on the distal surface of the posterolateral edge of the lateral tibial condyle and faces distolaterally. The proximal articular surface of the tibia (for the femoral condyles) is not continuous with the fibular facet; the tibia and fibula are unfused, and form a synovial joint.

A small eminence, identified as likely homologous with the lateral tibial tubercle (= Gerdy’s tubercle) in human anatomy [103,104], is clearly evident on the proximal epiphysis of the tibia of *Periptychus*, which provided an insertion point for the iliotibial band, the vertical component of the fascia lata. It forms a small, but prominent protuberance on the anterolateral surface of the proximal tibia, just below the lateral condyle and between the tibial tuberosity and proximal fibular facet. This feature is well developed in *Periptychus* (and also *Pantolambda*), but is not as striking as the condition in *Ectoconus*. Both *Mithrandir* and *Arctocyon* exhibit a much less well-developed tubercle in comparison to *Periptychus*.

The tibial diaphysis of *Periptychus* is very slightly curved medially in anterior view. The curvature of the diaphysis of *Periptychus* is like that seen in *Ectoconus* and *Mithrandir*, and is more curved than the tibia of *Arctocyon*. The tibial curvature of *Periptychus* is not as pronounced as the condition in *Carsioptychus* and *Pantolambda*, where the tibial diaphysis is distinctly bowed. The shape of the tibia of *Periptychus* in cross section is variable along the length of the shaft. Proximally the shaft is mediolaterally compressed, forming an isosceles triangle shaped cross section. Distally, the shaft broadens mediolaterally but constricts anteroposteriorly, forming an equilateral triangular profile.

The anterior tibial crest (= cnemial crest) of *Periptychus* is well developed, forming a broad longitudinal ridge that extends from the tibial tuberosity distally down 65% of the diaphysis, terminating in a small, poorly-developed tibial crest tuberosity. The condition seen in *Periptychus* is intermediate between the more salient condition seen in *Carsioptychus*, *Ectoconus*, *Mithrandir* and *Pantolambda* and the more gracile condition seen in *Arctocyon*. In *Carsioptychus*, *Ectoconus*, *Mithrandir* and *Pantolambda* the anterior crest is prominent along its entire longitudinal length, terminating in a tuberosity that projects more anteriorly than the anterior crest. In contrast, in *Arctocyon*, the tibial crest is less prominent distally and does not terminate in a prominent tuberosity (even less distinct than the condition of *Periptychus*). In anterior view, the tibial crest of *Periptychus* becomes sharper distally, and exhibits a small lateral deflection along its distal half where the tendon of the part of the semitendinosus muscle would have passed and the tendon of the gracilis muscles would have inserted on the medial surface of the crest (the latter insertion area is demarcated by a small depression). The lateral surface of the tibial crest received the insertion of the biceps femoris muscle. The lateral deflection of the crest in *Periptychus* is broadly like that in *Ectoconus*, but not as extreme as the condition in *Mithrandir*, where the tibial crest is very strongly deflected laterally, so that in anterior view the crest overhangs the lateral side of the diaphysis. This is contrary to the condition in *Arctocyon*, where there is no lateral deflection of the distal portion of the tibial crest; however, it should be noted that the scars from the semitendinosus and gracilis muscles are well developed in *Arctocyon* (forming comparatively deep, elongate, sinuous sulci), more so than in *Periptychus* and the other comparison taxa.

The medial surface of the proximal diaphysis of *Periptychus*, between the tibial crest anteriorly and the popliteus crest posteriorly, is near flat, with a very shallow depression towards its center. In *Ectoconus* and *Mithrandir*, this area appears slightly more excavated, and in *Arctocyon* it is deeply excavated in comparison to the periptychid taxa. The popliteus line of *Periptychus* is well developed, and it extends obliquely downward from the posterior border of the medial tibial condyle. The popliteal line forms the lateral border of a large triangular area for the insertion of the popliteus muscle, and mediolaterally truncates distally into a rounded crest, which extends the entire length of the tibia. The distal apex of the triangular area is demarcated by a small, rounded eminence. The lateral border of the popliteal area forms a sharp crest for the origin of the soleus muscle. *Ectoconus* possesses a similar triangular popliteal area as *Periptychus*, whereas in *Arctocyon* this condition appears to be absent, and instead the popliteal crest forms a longitudinal ridge.

The lateral surface of the proximal diaphysis of *Periptychus* is deeply excavated by two fossae separated by a tall ridge. The anterior-most of these fossa is positioned adjacent to the anterior tibial crest. It is shallow and provided attachment for the origin of the anterior tibialis muscle. The anterior tibialis fossa of *Periptychus* is similar in development to *Mithrandir* and poorly developed in comparison to *Ectoconus*, *Arctocyon* and *Pantolambda*, where the fossa is more clearly excavated and longitudinally extensive (extending distally along the length of the anterior tibial crest and excavating its lateral surface). The posterior fossa of *Periptychus* is for the origin of the posterior tibialis muscle and faces posterolaterally. It is relatively deep and more developed in comparison to the anterior fossa. Proximally it forms a deep, longitudinally ovoid fossa, but it is not distally extensive. The posterior tibialis fossa of *Periptychus* is not as developed as in *Ectoconus*, *Arctocyon* and *Pantolambda*, all of which exhibit a deeply excavated fossa which is more distally extensive than in *Periptychus*. In *Periptychus*, the anterior and posterior fossae are separated by a prominent longitudinal crest, which is positioned directly medial to the fibula and extends along the entire length of the tibial diaphysis to the distal tibiofibular facet; the interosseous membrane would have attached here. The crest of *Periptychus* is more prominent than in *Pantolambda* and *Arctocyon*, where the crest is lower and more rounded in profile, but not as salient as in *Carsioptychus* and *Ectoconus*, where it forms a sharp, tall ridge proximally and a sharp angle on the distal diaphysis.

Distally, the tibial diaphysis of *Periptychus* constricts below the tibial tuberosity, before expanding towards the distal epiphysis. In medial aspect, the medial surface of the distal epiphysis is marked by a sulcus, which extends distally towards the medial malleolus for the insertion of the tibiofibular interosseous ligament. This sulcus likely directed the tendons of the tibialis posterior and flexor digitorum tibialis muscles distally towards the pes. The sulcus extends proximally along 17% of the diaphysis from the edge of the distal epiphysis. This sulcus is well developed in *Periptychus*, but not as much as in *Carsioptychus* and *Mithrandir*. In *Mithrandir*, it forms a comparatively deep longitudinal sulcus, whereas in *Carsioptychus* it forms a triangular depression like that in *Periptychus*, but is comparatively deeper in proportion to the rest of the distal epiphysis. In *Periptychus*, the distal fibula facet is positioned on the medial corner of the distal tibial diaphysis. It is small and shallowly convex, forming a slight prominence. The distal border of the tibial facet forms a right angle with the astragalar facet on the distal surface of the epiphysis.

The distal tibial epiphysis of *Periptychus* is moderately expanded in relation to the proximal epiphysis. In *Periptychus*, the maximum width of the distal epiphysis is 32% smaller than the maximum width of the proximal epiphysis. This is somewhat expanded in comparison to *Arctocyon*, where the distal epiphysis is 40% smaller than the proximal epiphysis, but not as expanded as *Mithrandir*, where the distal epiphysis is only 25% smaller than the proximal epiphysis. The distal epiphysis of *Periptychus* is rotated anticlockwise by approximately 45° relative to the proximal epiphysis.

The medial malleolus of *Periptychus* forms a robust, anteroposteriorly elongate and distally prominent process on the anteromedial border of the distal epiphysis. The anterior border of the medial malleolus is positioned on the anterior border of the distal epiphysis. In distal aspect, the anteroposterior long axis of the malleolus is orientated at a slight angle (anterolateral to posteromedial) relative to the parasagittal axis of the tibia. The lateral surface of the medial malleolus is concave, with a smooth surface that is continuous with the rest of the articular surface of the distal epiphysis. The medial surface of the medial malleolus is flat with a roughened surface. The anterior surface includes the distal-most prominence of the medial malleolus (which is rounded as opposed to a ridge-like protuberance) and is roughened, for the attachment of a portion of the deltoid ligament. The posterior surface is dominated by the malleolar sulcus, which is orientated anteroposteriorly and would have lodged the tendons of the tibialis posterior and flexor digitorum longus muscles (this sulcus extends proximally up the diaphysis of the tibia).

The overall proportions of the medial malleolus of *Periptychus* are broadly comparable to those of *Carsioptychus* and *Pantolambda*. It is more distally prominent than in *Ectoconus* and *Arctocyon*, but not as robust in its transverse proportions as these taxa. The medial malleolus of *Periptychus* is not as distally prominent as that of *Mithrandir*, which forms a strong distal projection.

The articular surface of the distal tibial epiphysis of *Periptychus* is dominated by two facets, which face distolaterally and are divided by a shallow ridge. In *Pantolambda* these two facets are similar in shape to *Periptychus*, but face more distally. In *Periptychus* the medial-most of these facets is larger, circular in profile and more deeply excavated than the lateral facet, which is subrectangular. The distal epiphysis of the tibia of *Periptychus* is transversely broader and more squared in profile than the distal epiphysis of *Arctocyon*. However, the ridge dividing the two distolaterally directed astragalar facets in *Arctocyon* is broader and more offset than that of *Periptychus*.

In anterior aspect, a small tubercle is present on the anterior border of the distal epiphysis, just lateral to the medial malleolus. The tubercle would have fit into a corresponding pit on the astragalus when the foot was in extreme dorsiflexion. A similarly sized tubercle is seen in *Arctocyon* and a weaker prominence is present in *Carsioptychus*, but this feature is apparently absent in *Ectoconus* and *Mithrandir*.

#### Fibula

**Fig 39:**
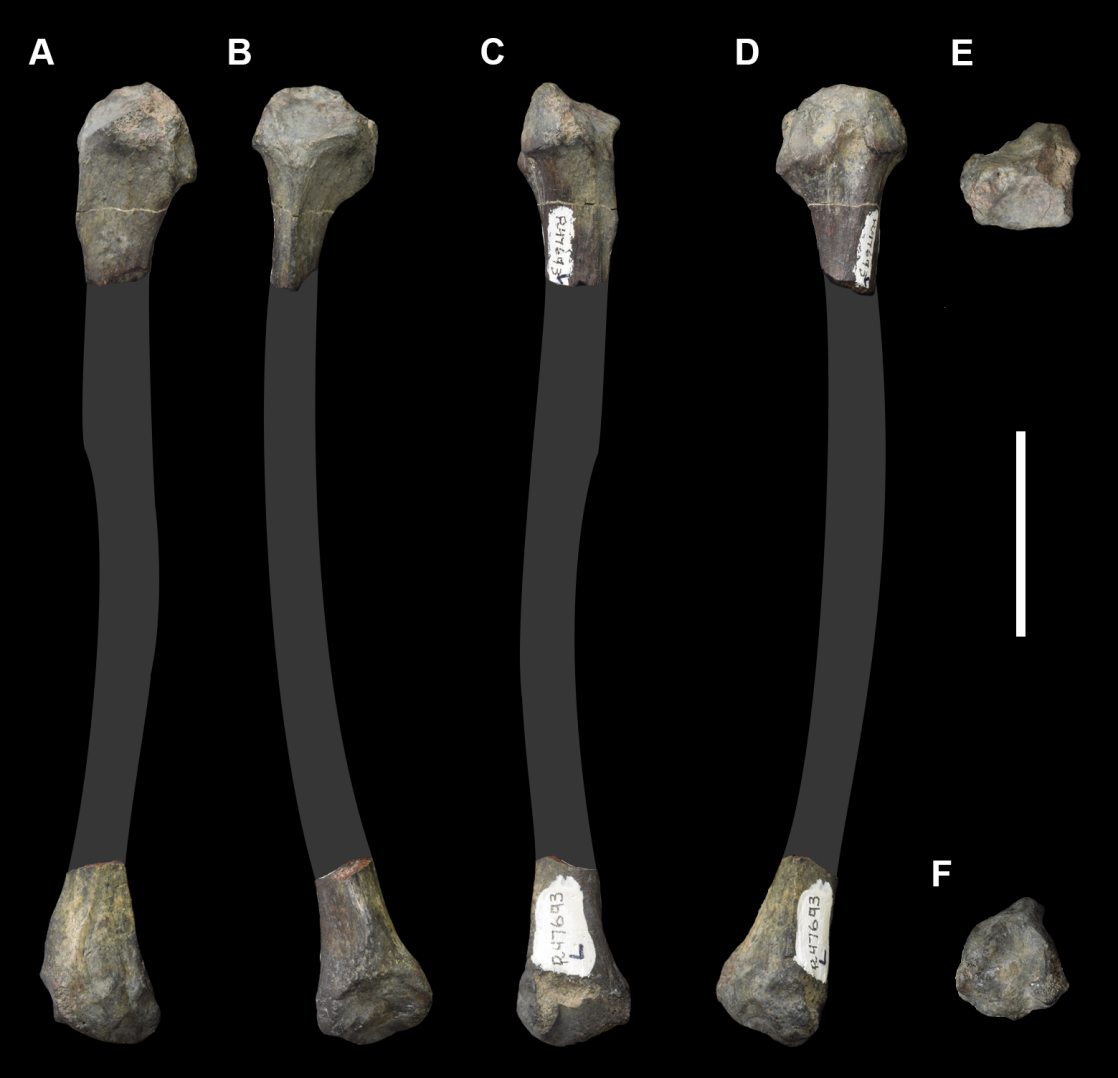
Left fibula of *Periptychus carinidens* (NMMNH P-47693). (A) anterior view; (B) medial view; (C) posterior view; (D) lateral view; (E) proximal view, (F) distal view. Scale bar: 30mm.

**Fig 40:**
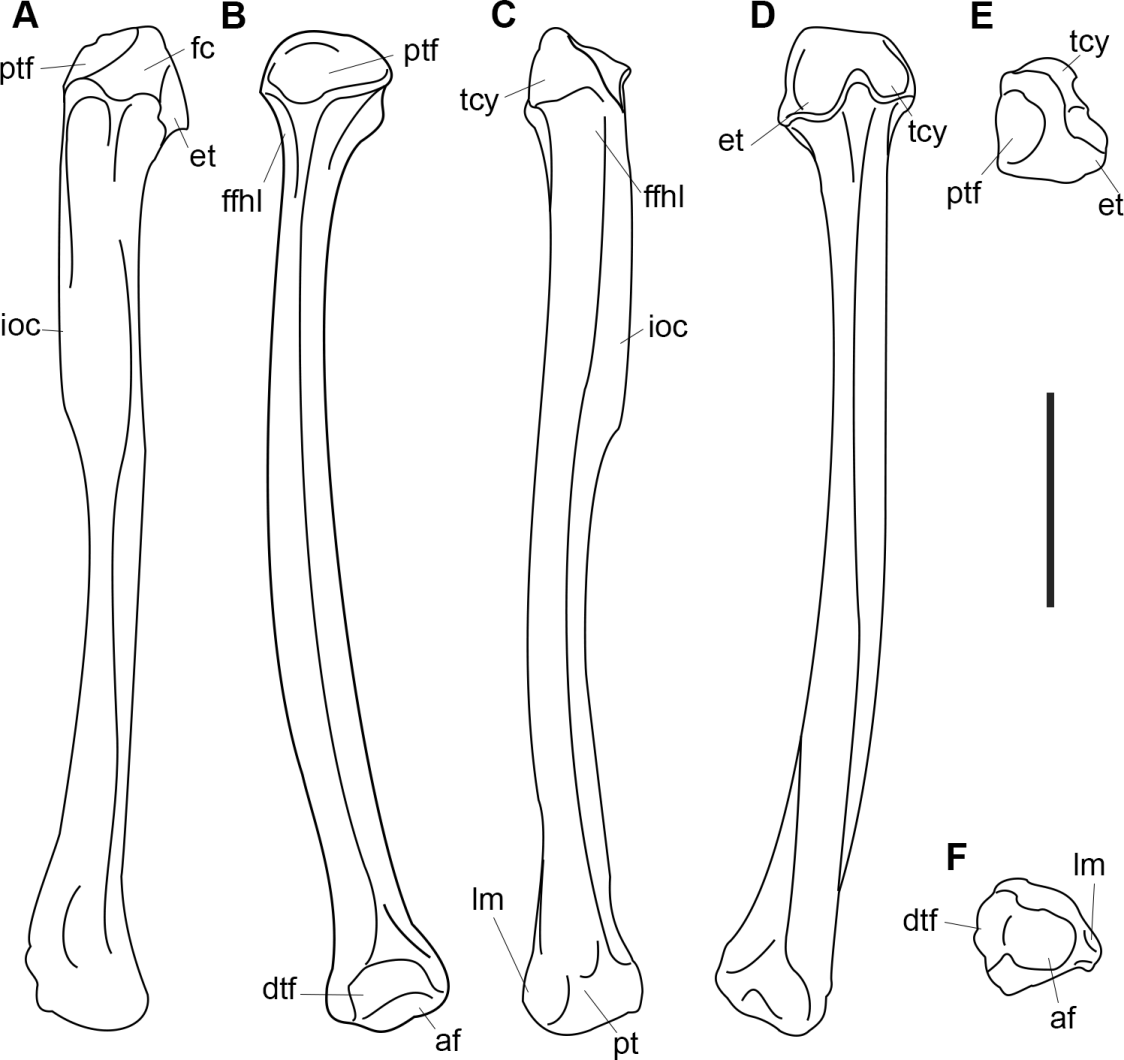
Annotated line drawing of the left fibula of *Periptychus carinidens*. (A) anterior view; (B) medial view; (C) posterior view; D, lateral view; (E) proximal view; (F) distal view. Abbreviations: af, astragalar facet, dtf, distal tibial facet; et, extensor digitorum longus tubercle ffhl, fossa for the flexor hallucis longus; ioc, interosseous crest; lm, lateral malleolus; tcy, tubercle for attachment of the cyamella ligament; ptf, proximal tibial facet. Scale bar: 30mm.

NMMNH P-19430 includes the proximal epiphysis of the right fibula concreted to the shaft (note the fibula is displaced distally and not fused in articulation with the tibia). NMMNH P-47693 includes the proximal and distal epiphyses of the left fibula (Figs 39 and 40). NMMNH P-35194 includes the proximal portion of the left fibula in articulation with the tibia and a tarsal bone. This specimen is highly concreted, limiting detailed observation of the anatomy. AMNH 837 includes a nearly complete right fibula (missing the interosseous crest) in articulation with the tibia. Comparisons are made to *Carsioptychus coarctatus* (AMNH 27601), *Ectoconus ditrigonus* (AMNH 16500, figured in [5]), *Mithrandir gillianus* (NMMNH P-3083, figured in [48]). *Arctocyon primaevus* (MNHN.F.CR74 figured in [52]), and *Pantolambda bathmodon* (AMNH 16663, figured in [5]).

The fibula of *Periptychus* is comparatively gracile in relation to the tibia, and in relation to both the tibiae and fibulae of the comparison taxa. In *Periptychus*, the proximal and distal fibular epiphyses are not fused to the tibia, but instead they form synovial joints. In articulation, the tibia and fibula are widely spaced along their entire length. The proximal epiphysis of the fibula of *Periptychus* is not mediolaterally expanded as in *Ectoconus* and, to a lesser degree, in *Carsioptychus*. However, the proximal epiphysis of *Periptychus* is somewhat proximodistally expanded in comparison to *Pantolambda*, although it does not contact the femur when in articulation. The proximal tibial facet is anteroposteriorly broad, forming two wings on either side of the interosseous crest. The anterior wing is substantially larger than the posterior wing. A similar condition is evident in *Arctocyon*, but differs in that the anterior wing is positioned more proximal relative to the posterior wing, and as such the two wings are more distinctly separated.

In anterior aspect, a moderately well-developed tuberosity is positioned on the lateral edge of the proximal fibular epiphysis of *Periptychus*. This would have been the origin for part of the extensor digitorum longus muscle. This feature is also present in *Carsioptychus*, *Ectoconus* and *Arctocyon*. The condition of *Periptychus* closely resembles that of *Arctocyon* and is more developed than in *Carsioptychus*, but notably differs from the morphology in *Ectoconus*. In *Ectoconus*, the tuberosity is larger in terms of surface area (in proportion to the proximal epiphysis), but it is not as offset relative to the body of the fibula, and is positioned more distally on the proximal epiphysis than in *Periptychus*. In *Periptychus*, a small depression is positioned proximally and slightly medial to the extensor tuberosity on the proximal border of the fibula, for the lateral femorofibular collateral ligament.

In posterior aspect, the posterior surface of the proximal epiphysis of *Periptychus* is dominated by a large triangular tuberosity. This tuberosity is also present in *Ectoconus* and *Arctocyon*, where it is more prominent than that of *Periptychus* (in *Ectoconus* it is particularly massively expanded). Based on observations of *Alcidedorbignya* and comparisons with an articulated crus of *Solenodon*, the tuberosity articulated with the cyamella sesamoid imbedded within the popliteus muscle [69]. *Periptychus*, *Ectoconus* and *Arctocyon* all exhibit a similar condition to *Alcidedorbignya* and *Solenodon*, which also corresponds with the morphology on the lateral condyle of the tibia. An interesting note is the absence of a triangular tuberosity on the fibula of the *Pantolambda* specimen (AMNH 16663) we observed.

The fibular diaphysis of *Periptychus* is slender and subtly sigmoidal in anterior and lateral views. The interosseous crest is moderately well developed, forming a medially projecting flange which extends distally along 50% of the length of the diaphysis. The interosseous crest of *Periptychus* is moderately prominent. It is considerably more striking than the crest in *Arctocyon*, but not as developed as in *Carsioptychus* or *Ectoconus*. In *Periptychus*, *Carsioptychus* and *Ectoconus* the interosseous crest forms a prominent medial flange which extends distally along 50% of the diaphysis, whereas in *Pantolambda* the crest is not as medially prominent as in the aforementioned periptychids, forming only a small crest distally along 30% of the diaphysis.

In *Periptychus*, a small fossa for the flexor hallucis longis is located on the posterior surface of the proximal diaphysis, adjacent to the interosseous crest, and buttressing the tubercle for the cyamella. In *Periptychus*, this fossa is shallow so that it does not strongly excavate the body of the fibula like it does in *Carsioptychus*, *Ectoconus* and *Arctocyon*. The fossa of *Periptychus* is not as poorly developed as that of *Pantolambda*, where there is little evidence of a distinct fossa.

The distal epiphysis of the fibula of *Periptychus* is only slightly larger than the proximal epiphysis (~10%) and is approximately half the size of the distal epiphysis of the tibia. Distally it articulates with the fibular facet of the astragalus, medially it articulates with the distal epiphysis of the tibia and posteriorly is articulates with the calcaneum. The distomedial surface of the distal fibula epiphysis is formed by the astragalar facet and the tibial facet. The astragalar facet is smooth, slightly concave and continuous with the tibial facet. The tibial articular facet is crescentic and faces slightly more medially than the astragalar facet. The tibial facet on the fibula is larger than the associated fibular facet on the tibia, and would have allowed for some movement between the two crural elements over the astragalus. A small tuberosity demarcates the posterior border of the tibial facet and is subequal in size to the lateral tibial malleolus. The calcaneal facet is positioned on the posterior surface of the distal fibular epiphysis. It is smooth, flat and subrectangular in profile, rather than crescentic as is the case in *Arctocyon*. Laterally, the calcaneal facet of *Periptychus* is delimited by the fibial malleolus. The fibial malleolus is laterally prominent, but does not extend distally beyond the astragalar facet. A distinct groove marks its posterior surface, which conveyed the peroneal tendons towards their various insertions within the tarsus. The tibial malleolus of *Periptychus* forms a discrete lateral projection rather than the more distal projection of *Arctocyon*. Interestingly, the lateral malleolus of *Periptychus* is not as well developed as the condition in *Mithrandir*, where the lateral malleolus forms a distinct, flange-like projection. *Ectoconus* exhibits an intermediate condition between *Periptychus* and *Mithrandir*.

#### Pes

The pes of *Periptychus* is known from an assortment of well-preserved specimens. AMNH 17075 is partial skeleton that includes a near complete articulated left pes, consisting of the astragalus, calcaneum, navicular, cuboid, ectocuneiform, mesocuneiform, entocuneiform and metatarsals II-V, with three of the proximal phalanges—I (proximal portion only), II (proximal portion only), IV (complete) and three of the intermediate phalanges—II (complete), III (complete), IV (proximal portion only). The specimen is mounted in articulation on a solid base, limiting observation to the exposed surfaces of the pes only. NMMNH P-47693 is a includes a left astragalus (with associated tibia and fibula). NMMNH P-19430 includes a concreted block composed of right tarsal elements (astragalus, calcaneum, cuboid, two proximal metatarsals and the distal epiphysis of the right tibia). NMMNH P-15356 includes two near complete and well-preserved left and right calcanea. AMNH 3636 includes a near complete pes composed of the astragalus, partial calcaneum, navicular, cuboid, ectocuneiform and metatarsals I-V (I and V are damaged). AMNH 3637 includes a right astragalus.

**Fig 41:**
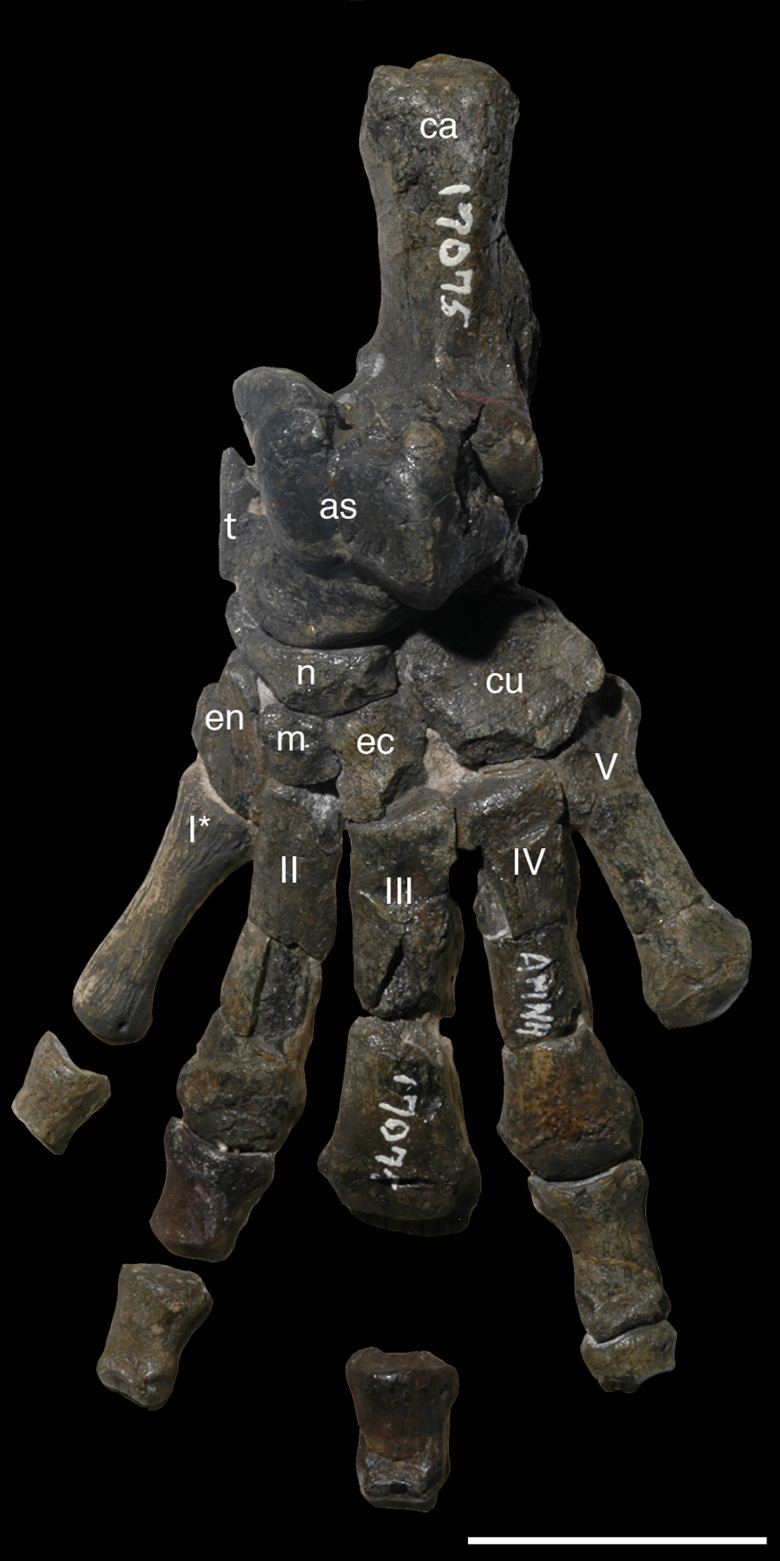
Left pes of *Periptychus carinidens* (AMNH 17075) in dorsal view. Abbreviations: as, astragalus; ca, calcaneum; cu, cuboid, ec, ectocuneiform; en, entocuneiform; m, mesocuneiform; n, navicular; t, tibiale; I-V refer to digit number; * denotes reconstructed elements. Scale bar: 30mm. Note that this specimen is reconstructed and mounted on a resin base. Photographs of the individual bones are illustrated and photographed in [5].

The following description of *Periptychus* will highlight noteworthy comparisons, but will not make exhaustive comparisons due to the sheer amount of material available. Comparisons of the pes of *Periptychus* are primarily made with *Carsioptychus coarctatus* (AMNH 27601), *Conacodon entoconus* (AMNH 3503), *Ectoconus ditrigonus* (AMNH 16500, figured in [5]), *Hemithlaeus kowalevskianus* (AMNH 3505a), *Mithrandir gillianus* (NMMNH P-3083, figured in [48]), *Oxyacodon* (AMNH 16368), *Protungulatum donnae* (referred specimen AMNH 118060, figured in [71]), *Arctocyon primaevus* (MNHN.F.CR750, CR752, CR17475, CR17474, CR 41, CR42, CR58, CR67, BR11862, CR335; figured in [52]), *Claenodon corrugatus* (AMNH 16543, figured in [5]), and *Pantolambda bathmodon* (AMNH 16663, figured in [5]),

The pes of *Periptychus* is plantigrade, pentadactyl and paraxonic, with broad, widely spaced digits much like the manus (Fig 41). The first and fifth digits are shorter than the second, third and fourth digits. The tarsus is composed of seven known bony elements: the astragalus, calcaneum, navicular, cuboid, ectocuneiform, mesocuneiform and entocuneiform. The presence of an eighth tarsal element in *Periptychus*, the so called ‘tibiale’ and/or ‘pre-hallux’, has been briefly mentioned by previous workers [5]. We will comment on this issue below. The tarsal arrangement of *Periptychus* is a non-serial (alternating) sequence, whereby the head of the astragalus articulates with both the navicular and the cuboid. The digits of *Periptychus* are robust, and include a well-developed hallux. The phalangeal formula is 2-3-3-3-3. The anatomical axes used for the description of the pes follow those used for the manus. They are anteroposterior, lateromedial and dorsoplantar. The dorsoplantar axis corresponds to dorsoventral axis, as the pes of *Periptychus* is plantigrade (or near plantigrade).

#### Proximal cruropedal joint

The proximal cruropedal joint is formed by articulation between the tibia and fibula proximally with the astragalus and calcaneum distally. The tibia and fibula are unfused but together form a broad distal crus that is mediolaterally wider than dorsoplantarly deep. The pedal articular surface is primarily formed by the astragalus, with a small contribution from the calcaneum. The tibial malleolus of *Periptychus* extends distally over the medial surface of the astragalus and would have served to securely brace the astragalus during dorsiflexion. The tibial malleolus is not distally prominent and would have provided no support to the astragalus. When considering the relative positions of the cruropedal elements it becomes evident that the astragalus must have articulated with the crus at an oblique angle, so that the mediolateral transverse axis of the astragalar body is angled at approximately 45°, with the surface of the trochlea facing medially.

#### Astragalus

**Fig 42:**
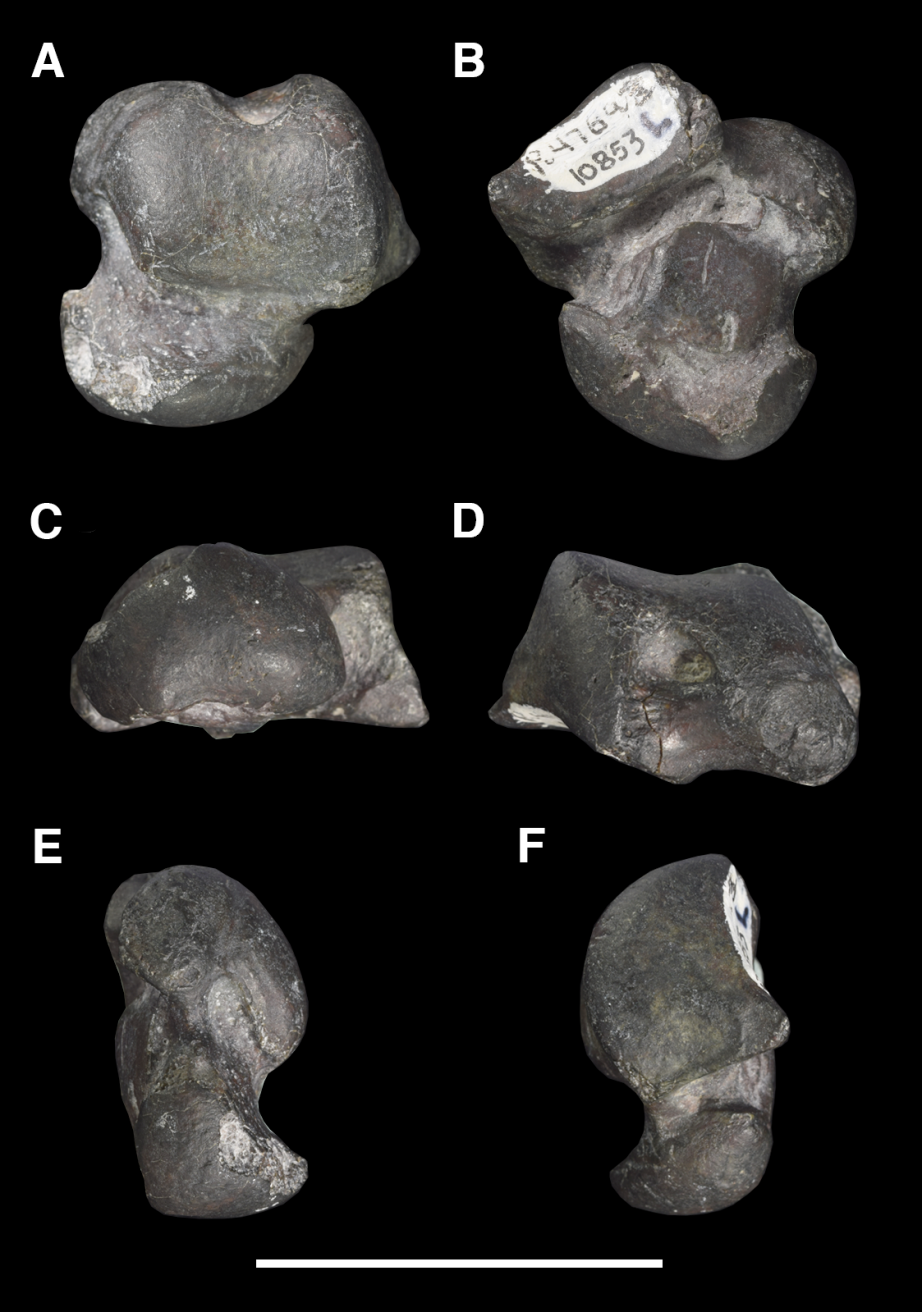
Left astragalus of *Periptychus carinidens* (NMMNH P-47693). (A) dorsal view; (B) plantar view; (C) anterior view; (D) posterior view; (E) medial view, (F) lateral view. Scale bar: 30mm.

**Fig 43:**
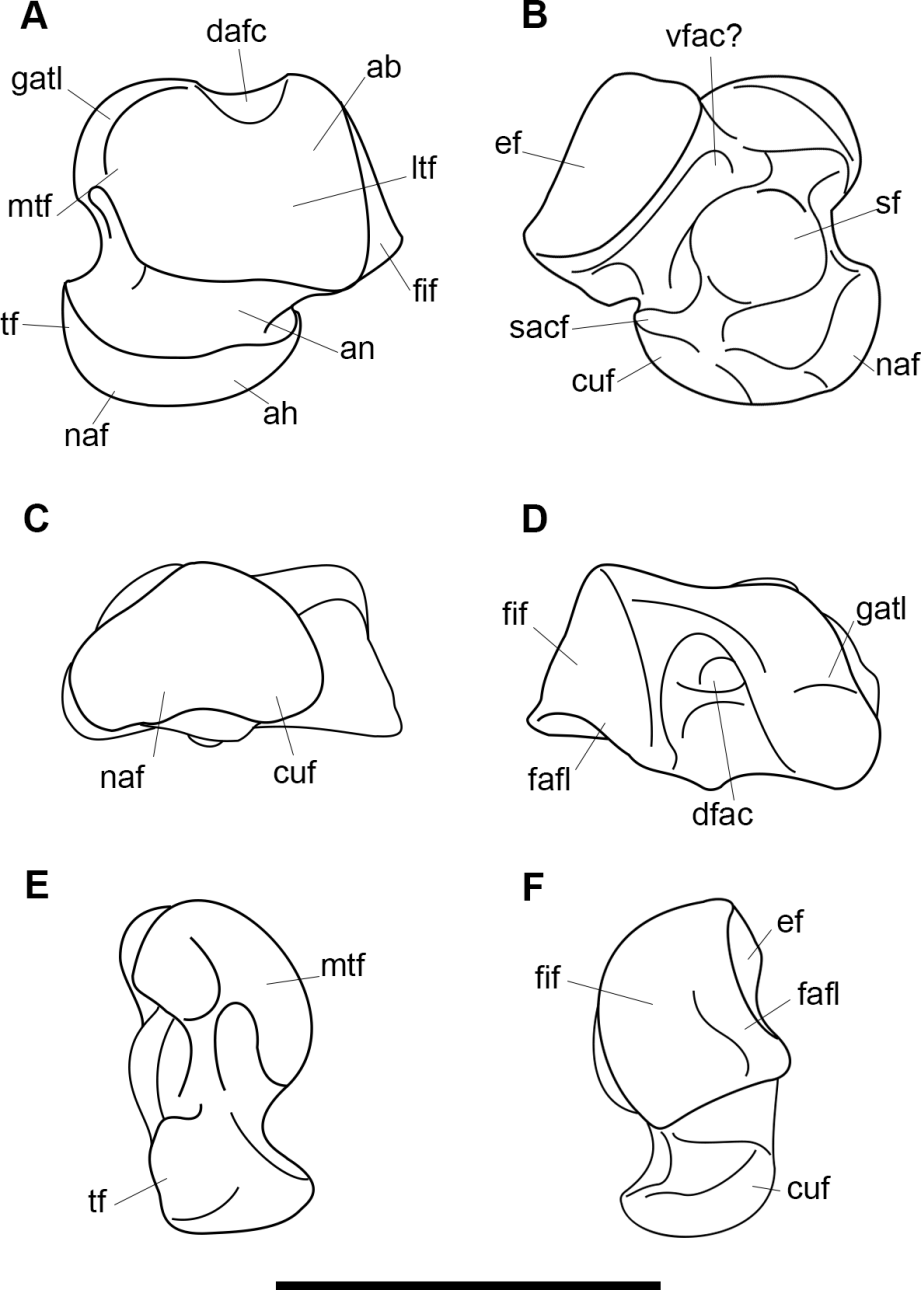
Annotated line drawing of the left astragalus of *Periptychus carinidens*. (A) dorsal view; (B) plantar view; (C) anterior view; (D) posterior view; (E) medial view, (F) lateral view. Abbreviations: ab, astragalar body; ah, astragalar head; an, astragalar neck; cuf, cuboid facet; dfac, dorsal foramen of the astragalar canal; ef, ectal facet; fafl, fossa for the astragalofibular ligament; fif, fibular facet; gatl, groove for the astragalotibial ligament; ltf, lateral tibial facet; mtf, medial tibial facet; naf, navicular facet; sacf, supplementary astragolocalcaneal facet; sf, sustentacular facet; tf, tibiale facet; vfac?, ventral foramen of the astragalar canal (opening not discernible). Scale bar: 30mm.

The astragalus forms the lower component of the cruropedal joint. Proximally it articulates with the distal epiphyses of the tibia and fibula, plantarly it articulates with the calcaneum and distally it articulates with the navicular and cuboid. No obvious muscles attached to the astragalus. The astragalus of *Periptychus* is distinctive: it is a robust, dorsoplantarly compressed bone with a quadrate body and a short and broad neck and head (Figs 42 and 43). The proportions are most like those of *Ectoconus* and *Pantolambda*, in that the mediolateral width of the astragalus is equal to slightly wider than the anteroposterior length (width/length = ~1). This differs from the arctocyonids, where the astragalus is typically longer than it is wide (*Protungulatum* 0.84, *Arctocyon* 0.91). The trochlea of *Periptychus* is short: the anteroposterior length of the trochlea relative to the anteroposterior length of the astragalus is 0.60, similar to *Pantolambda* (0.61). This is relatively short in comparison to *Ectoconus* (0.68) and *Arctocyon* (0.68), but longer than *Protungulatum* (0.57).

The astragalar body of *Periptychus* is subquadrate in dorsal view and dominated by the lateral tibial articular facet, with a well-developed posteromedial expansion of the astragalar body. The lateral tibial facet forms a shallow trochlea with low-lying lateral and medial keels. The lateral keel is marginally taller than the medial keel, and strongly convex with a prominent fibula facet. The medial keel is positioned lower and is more rounded. The posteromedial border of the astragalar body is strongly expanded, forming a rounded projection from underneath the trochlea. In dorsal aspect, the anteroposterior long axis of the trochlea and its associated groove are offset at a 20° oblique angle (posterolateral to anteromedial) relative to the anteroposterior long axis of the pes.

In medial view, the trochlea of *Periptychus* covers an arc of approximately 140° compared to only 120° in *Protungulatum* [71]. The trochlea is consistent in mediolateral width along its entire anteroposterior length. Anteriorly, the trochlea is clearly delimited from the astragalar neck by a subtle crease. The condition exhibited by *Periptychus* (which is also seen in *Ectoconus* and *Hemithlaeus*) differs from that in *Protungulatum*, *Arctocyon* and *Claenodon*, in which the medial half of the anterior border of the trochlea extends anteriorly onto the neck of the astragalus to form a shelf (beyond the anterior border of the lateral half of the trochlea). The condition in *Pantolambda* is proportionally like *Periptychus*, but morphologically distinct; the head is not strongly differentiated from the body of the astragalus, with the medial border of the trochlea and medial tibial facet extending anteriorly to form a near continuous surface with the head.

The astragalus of *Periptychus* is perforated by a large astragalar foramen (= canal), positioned on the posterodorsal surface of the tibial trochlea. Plantarly, the foramen opens into a deep and broad, obliquely orientated sulcus for the tendons of the flexor digitorum fibularis. The sulcus continues around onto the plantar surface of the body of astragalus. The relative size and position of the astragalar foramen and associated sulcus of *Periptychus* are broadly like the morphology exhibited by *Ectoconus*, *Hemithlaeus* and *Pantolambda*. The condition exhibited by *Periptychus* is proportionally enlarged in comparison to *Protungulatum* and *Arctocyon*. In *Periptychus*, the lateral side of the tibial trochlea does not extend posteroplantarly beyond the proximal border of the astragalar foramen, whereas the medial side of the tibial trochlea extends slightly further posterodorsally to reach the plantad border of the foramen. This implies that the tibia may have partially covered the contents of the astragalar foramen during extreme plantar flexion. This condition is also observed in all the comparison taxa.

The border between the lateral and medial tibial articular facets forms the medial rim of the trochlea. In *Periptychus*, this border is poorly demarcated, forming a rounded continuous rim that, in medial view, is not as sharp or strongly convex as the lateral rim of the trochlea. In posterior aspect, the lateral and medial tibial facets meet at an obtuse (~130°) angle, so that the medial tibial facet forms a proximomedially directed surface. The surface of the medial tibial facet is smooth and gently convex. The posterior portion of the facet is dorsoplantarly deeper than the anterior portion. The posterior border of the facet is demarcated from the posteromedial projection of the astragalar body by a shallow sulcus, where part of the astragalotibial ligament, which forms part of the deltoid ligament, inserted. This morphology is clearly defined in *Periptychus*, but is not as strongly defined as in *Ectoconus*, *Protungulatum* or *Pantolambda*, where the sulcus forms a distinct crease (curiously, this feature is lacking in *Arctocyon*, but present in *Claenodon*).

The lateral component of the astragalar body is formed by the fibular articular facet and the lateral process of the astragalus. In *Periptychus*, the fibular articular facet is prominent, producing a large, laterally projecting conical protuberance on the lateral wall of the body of the astragalus, with the apex of the cone formed by the lateral process of astragalus. The fibular articular facet of *Periptychus* (and the other periptychids) is proportionally and morphological like the condition seen in *Protungulatum*: in these taxa, the lateral process and fibular facet are laterally prominent, forming a conical protuberance. Medially, the fibular facet of *Periptychus* is clearly delimited from the tibial articular facet by the lateral keel of the trochlea, defining an angle of 115°, so that the surface of the fibular articular facet faces proximolaterally and is slightly convex in dorsal view. The lateral keel of the trochlea is sharpest anteriorly, becoming more rounded posteriorly. The condition of *Periptychus* differs from that of *Arctocyon* and *Claenodon*, where the fibular facet is not as laterally prominent, but instead projects more plantarly at a ~90° angle with the tibial trochlea. In *Periptychus*, a small circular depression is present on the anterior portion of the fibular articular facet, which acted to stabilize the fibula and demarcates the fibular facet from the lateral process of the astragalus. A shallow, but relatively broad sulcus is seen on the posterior border of the fibular facet of *Periptychus*, which is where the posterior astragalofibular ligament would have inserted. This sulcus is consistently observed in the comparison taxa, but is variable in its morphology. *Ectoconus*, *Pantolambda* and *Protungulatum* exhibit a morphologically similar, but proportionally deeper sulcus than in *Periptychus*. In *Arctocyon*, the sulcus is particularly well developed, forming a dorsoplantarly deep, triangular shaped pit.

In *Periptychus*, the plantar surface of the astragalar body articulates with the calcaneum via two points of contact: the ectal (calcaneoastragalar) and the sustentacular facet. the ectal facet extends obliquely along the posterolateral border of the ventral surface of the astragalus, with the surface of the facet facing plantarly. In plantar aspect, the facet is transversely elongate and relatively anteroposteriorly deep, tapering to a sharp point laterally, which comprises the well-developed lateral process of the astragalus. The ectal facet is more laterally prominent in *Periptychus* (and the other periptychids) than in *Protungulatum*, *Arctocyon* and *Claenodon*. In *Periptychus*, the plantar tuberosity forms a small, rounded eminence on the posteromedial border of the ectal facet. This feature is also present and more strongly developed, projecting further plantarly, in *Arctocyon* and *Claenodon*.

The sustentacular facet of *Periptychus* is positioned towards the center of the astragalus on the plantar surface. It is separated from the ectal facet by a deep, but narrow sulcus astragali. A deep pit at the medial end of the sulcus suggests that the astragalar canal opened in this region, but an open canal is not evident on any of the specimens observed. A similar pit is seen in *Ectoconus* and *Protungulatum* and an intact astragalar canal is present in *Arctocyon*, opening at the same position. The sustentacular facet of *Periptychus* is large relative to the size of the astragalus: the maximum mediolateral width of the sustentacular facet is equivalent to 60% of the mediolateral width of the astragalar head. This is substantially larger than in *Protungulatum* (35%) and *Arctocyon* (40%). In *Periptychus*, the sustentacular facet is circular, subtly convex along its longitudinal axis and positioned at a 45° oblique angle (posteromedial-anterolateral) relative to the long axis of the astragalus. The anterior end of the facet is near continuous with the articular surface of the head of the astragalus. The posterior end of the facet is not as continuous with the body of the astragalus as the anterior end is with the head, but is not distinctly separate from it either. The sustentacular facets of *Ectoconus* and *Hemithlaeus* closely resemble one another and exhibit a broadly similar morphology to *Periptychus*. However, they differ in that they both possess a more ovoid sustentacular facet (transversely narrower than *Periptychus*) that is not posteriorly continuous with the body of the astragalus. *Protungulatum* and *Arctocyon* exhibit a different sustentacular morphology due, in part, to the expansion of the astragalar neck. As such, the facet is longer, and in *Arctocyon* the anterior portion of the facet is strongly continuous with the astragalar head. Further to this, in *Arctocyon* and *Claenodon* the facet is shallowly concave, particularly along its transverse plane. The sulcus astragali in both *Protungulatum* and *Arctocyon* is much broader than in *Periptychus*.

The astragalar neck of *Periptychus* is short, contributing to only ~18% of the anteroposterior length of the astragalus, and mediolaterally broad, corresponding to 55% of the mediolateral width of the astragalar body. Despite its robust proportions, the neck is still well defined from the astragalar body and head, and the anterior border of the trochlea is clearly delimited. In dorsal aspect, the astragalar neck is medially offset relative to the astragalar body by an angle of approximately 45°. The dorsal surface of the astragalar neck is excavated relative to the body and head of the astragalus, forming a distinct shelf that apparently would have buttressed the tibia during extreme dorsiflexion of the pes.

A small, shallow pit adjacent to the anteromedial corner of the astragalar trochlea appears to fit the anterior tubercle on the distal epiphysis of the tibia. *Periptychus* does not possess a cotylar fossa or check facet [105,106]. A deep fossa is present on the medial side of the astragalus, forming a dorsoplantarly compressed, ovoid pit that is anteriorly continuous with the astragalar neck. The pit does not appear to articulate with the medial malleolus in any way and most likely served as an insertion point for a portion of the tibioastragalar component of the deltoid ligament. A homologous morphology is evident in *Protungulatum*, but the fossa is shallower and broadly open anteriorly. *Ectoconus* exhibits a more developed morphology than *Periptychus*, with a deep fossa that strongly undercuts the tibial trochlea.

The astragalar head of *Periptychus* is mediolaterally broad and dorsoplantarly compressed. The surface of the head is smooth and broadly convex. In anterior aspect, the lateral portion of the articular surface of the head is more extensive than the medial portion, and is positioned slightly more dorsally, so that the mediolateral transverse axis of the astragalar head is tilted relative to the mediolateral transverse axis of the body.

The navicular facet dominates the surface of the head, forming a large, near convex facet. A very shallow groove is observed towards the plantar-most surface of the navicular facet, with a small protuberance marking the medial edge of the groove. The surface of the navicular facet medial to this groove faces medially, interrupting the arc of the articular facet surface. A similar grooved morphology is also seen in *Ectoconus*, *Mithrandir* and *Arctocyon*, but not in *Protungulatum*.

Laterally on the astragalar head, the navicular facet of *Periptychus* is poorly demarcated from the cuboid facet, as the two meet at an almost uninterrupted articular surface (marked only by a slight, rounded protuberance). This differs from the condition in *Ectoconus*, where the transition between the navicular facet and cuboid facet is evidenced by a shallow ridge and a discontinuity in the arc of the articular surface. In *Periptychus*, the cuboid facet is orientated obliquely (proximoplantarolateral to distodorsomedial) relative to the proximodistal long axis of the astragalus. The surface of the facet is convex and faces plantarolaterally. In *Ectoconus*, the surface of the cuboid facet is marked by a shallow groove on its plantar edge. This morphology is absent in *Periptychus*.

The medial side of the astragalar head of *Periptychus* exhibits a distinct, distomedially facing articular surface, which extends from the astragalar head onto the neck. A similar facet is observed in all the comparison taxa (albeit reduced in *Protungulatum*); however, in *Ectoconus*, *Arctocyon* and *Claenodon* the facet faces more medially than distomedially. Previous workers have associated this facet with the presence of an additional tarsal ossicle, the so-called ‘tibiale’, most notably in *Claenodon* [107] and also mentioned by Matthew [5] with regards to *Ectoconus* and *Pantolambda* (the tibiale of *Periptychus* is discussed below).

#### Calcaneum

**Fig 44:**
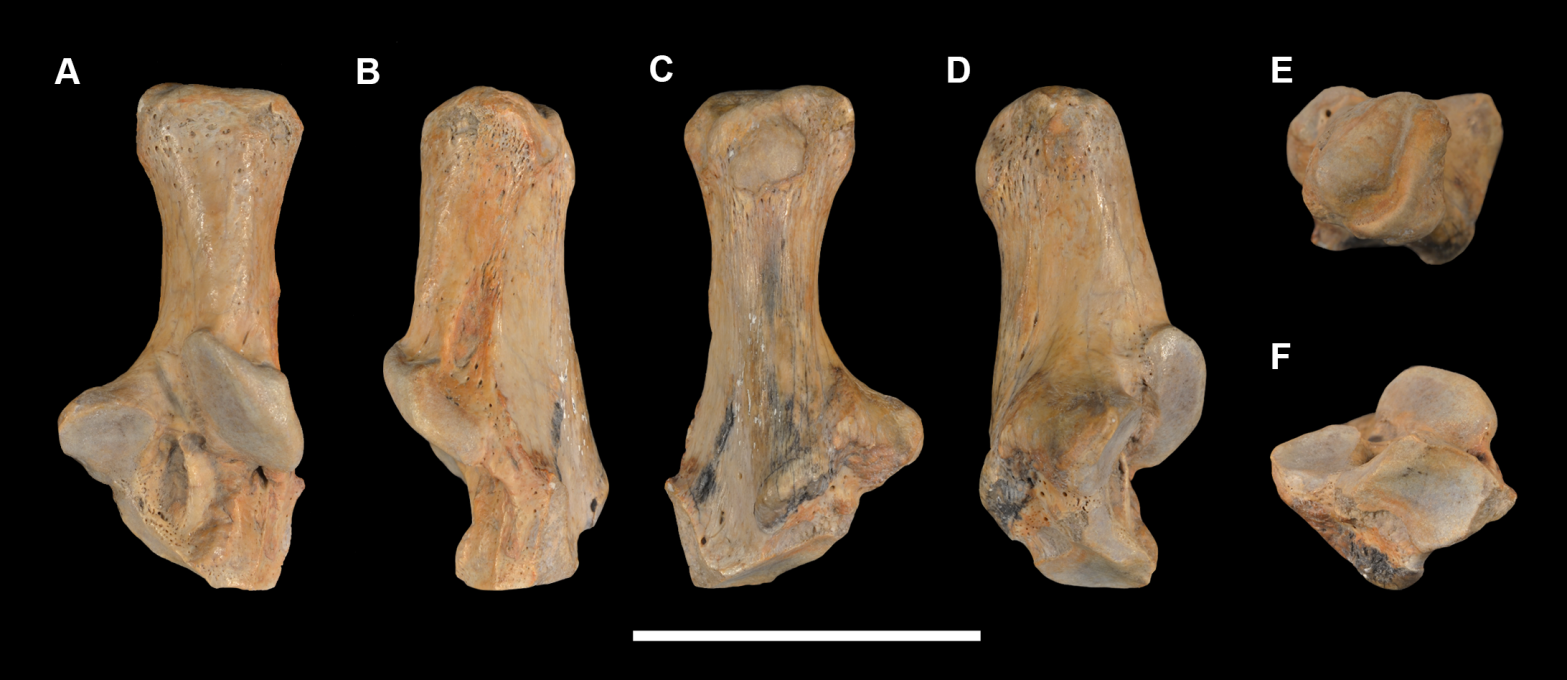
Left calcaneum of *Periptychus carinidens* (NMMNH P-48429). (A) dorsal view; (B) lateral view; (C) plantar view; (D) medial view; (E) distal view, (F) anterior view. Scale bar: 30mm.

**Fig 45:**
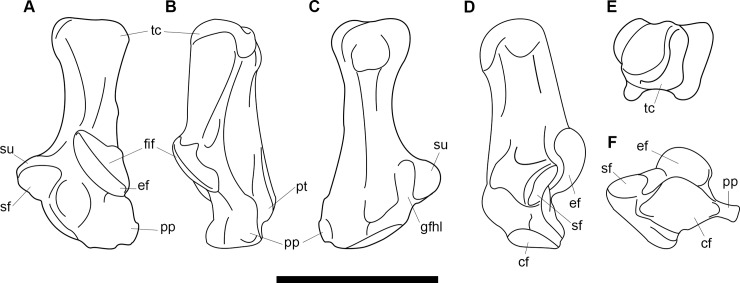
Annotated line drawing of the left calcaneum of *Periptychus carinidens*. (A) dorsal view; (B) lateral view; (C) plantar view; (D) medial view; (E) distal view, (F) anterior view. Abbreviations: cf, cuboid facet; ef, ectal facet; fif, fibular facet; gfhl, groove for the flexor hallucis longus tendon, pp, peroneal process; pt, plantar tubercle; sf, sustentacular facet; su, sustentaculum; tc, tuber calcanei. Scale bars: 30mm.

In *Periptychus*, the calcaneum is the largest tarsal bone in the pes and it forms a prominent, robust heel (Figs 44 and 45). The tuber of the calcaneum is moderately elongate, representing 50% of the anteroposterior length of the bone. The tuber of *Periptychus* is proportionally shorter in comparison to that of *Ectoconus*, where it represents 60% of the total length of the calcaneum. Interestingly, *Protungulatum*, *Arctocyon* and *Pantolambda* all possess a tuber that accounts for close to 50% of the total length of the calcaneum. The posterior apex of the calcaneal tuber of *Periptychus* is bulbous and enlarged relative to the rest of the structure. The roughened surface of the apex served for the insertion of the tendon of the gastrocnemius and soleus posteriorly, the plantaris medially and the plantar aponeurosis plantarly. The apex of the tuber of *Periptychus* is similar in morphology, but not as robust as that of *Ectoconus*, and both these taxa exhibit a proportionally more swollen apex than *Protungulatum*, *Arctocyon* and *Pantolambda*.

The calcaneal body of *Periptychus*, defined as the part of the calcaneum that seats the articular facets, is relatively expanded, equating to half the overall length of the bone. The facets are widely separated and allowed for the superposition of the astragalus directly over the calcaneum. In *Periptychus*, the astragalus is more directly superimposed over the calcaneum than in *Arctocyon* and *Claenodon*, where the astragalus is more medially positioned relative to the calcaneum.

The ectal (= astragalocalcaneal) and sustentacular facets are positioned towards the anterior end of the calcaneum, but are not immediately adjacent to the cuboid facet and are separated by a deep, but relatively narrow calcaneal sulcus. The calcaneal sulcus corresponds to an equally deep groove on the astragalus, and together the two structures form a large tarsal sinus that transmitted the interosseous astragalocalcaneal ligament, blood vessels and nerves. In *Protungulatum*, *Arctocyon* and *Claenodon* the calcaneal sulcus is proportionally broader than in *Periptychus* and any of the other periptychid taxa observed.

In *Periptychus*, the ectal facet is positioned at a posteromedial-anterolateral oblique angle on the dorsal surface of the body of the calcaneum. The facet is roughly ovoid in shape, elongate along its subanteroposterior axis. The articular surface is highly convex along its longitudinal plane and faces broadly anterodorsomedially. The articular surface of *Periptychus* forms a continuous, arched surface, whereas in *Arctocyon* (but not *Claenodon*) the surface of the ectal facet is subtly angled, and therefore is subdivided into a more medially directed posterior portion and a more anteriorly directed anterior portion. In *Periptychus*, the posterior border of the facet does not reach the medial border of the calcaneum, and the anterior border is positioned well posterior of the cuboid facet. In *Arctocyon* and *Claenodon*, the posterior border of the facet extends to reach the medial border of the calcaneum. In *Protungulatum*, the facet extends more medially than in the periptychids, but does not reach the medial border.

In *Periptychus*, the ectal facet is laterally buttressed by the calcaneal fibular facet. The fibular facet forms a slim band along the dorsal edge of the ectal facet. The former facet is narrower posteriorly than anteriorly, with a convex articular surface. The border between the ectal and fibular facets is poorly delimited, forming a simple rounded ridge. The fibular facet morphology of *Periptychus* is also seen in the other periptychid taxa observed, with *Periptychus* possessing a proportionally broader facet than *Ectoconus*. The fibular facet in periptychids is highly reduced in comparison to *Protungulatum*, *Arctocyon* and *Claenodon*, where the facet forms a broad band, the surface of which is grooved in *Arctocyon* and positioned slightly ventral to the ectal facet.

The sustentaculum of *Periptychus* is transversely expanded, forming a prominent medial protrusion that projects at a 90° angle to the anteroposterior long axis of the calcaneum. The sustentacular facet is positioned on the dorsal side of the sustentaculum. It is ovoid to subquadrate in shape, shallowly concave and faces anterodorsally. In anterior aspect, the sustentacular facet is positioned slightly anterior to the ectal facet and closer to the anterior border of the calcaneum and the cuboid facet. A small accessory facet connects the sustentacular facet to the cuboid facet. The sustentacular facet of *Periptychus* (and the other periptychid taxa observed) is relatively more transversely expanded than that of *Protungulatum*, *Arctocyon* and *Claenodon*. In *Periptychus*, a broad groove extends along the plantar surface of the sustentacular facet, which transmitted the flexor hallucis longis. The medial border of the groove is demarcated by a large protuberance, which is also seen in *Ectoconus*, but not in *Protungulatum* or *Arctocyon*.

In *Periptychus*, the surface formed by the sustentacular and ectal facets, when viewed anteriorly, forms a relatively open configuration (~110°). This morphology is manifested in the axis of rotation between the astragalus and calcaneum. Movement between these two bones would have occurred around an axis set at a 45° oblique angle to the long axis of the calcaneum, with a small component of dorsoplantar movement due to the way the astragalus sits on the calcaneum (and considering the position of the tibia and fibula, so that astragalar trochlea faces dorsomedially). The sustentacular and ectal facets of *Periptychus* form a more open configuration than in *Protungulatum* (90°), suggesting that *Protungulatum* was capable of less dorsoplantar movement between the astragalus and calcaneum than *Periptychus*.

The cuboid facet of *Periptychus* is positioned on the anterior surface of the calcaneum. The facet forms an irregular oval in anterior aspect, which is strongly expanded laterally, but also retains some dorsoplantar depth. The articular surface is shallowly concave (more strongly concave along the transverse axis than the dorsoplantar axis) and set at an oblique angle (25°) relative mediolateral axis of the calcaneal body. The facet faces anteromedially and the dorsoplantar axis is orientated vertically. The transverse axis of the cuboid facet of *Periptychus* is not as obliquely orientated as in *Pantolambda*, where the facet is set at a 45° angle. The cuboid facet of *Periptychus* is broadly like that in the other ‘condylarth’ taxa, but it is proportionally more transversely expanded than in *Protungulatum* and *Arctocyon* (particularly the lateral portion). The cuboid facet of *Periptychus* is not as transversely expanded as in *Ectoconus*, although it is more expanded in the dorsoplantar direction.

The peroneal process of *Periptychus* forms a large, well-defined protuberance on the distal end of the calcaneum, with a small but sharp crest extending proximally along the calcaneal tuber. In dorsal view, the process is positioned close to the cuboid facet anteriorly, but it does not extend beyond the anterior border of the cuboid facet. Dorsally and plantarly, the peroneal process is flanked by shallow grooves for the passage of the peroneus brevis dorsally and the peroneus longus plantarly. The peroneal process of *Periptychus* is more anteroposteriorly elongate than the process in *Protungulatum*, but not as laterally prominent. Furthermore, the condition exhibited by *Periptychus*, although similar in morphology, is not as well developed as the condition in *Ectoconus*, where the peroneal process is massive, forming a laterally prominent shelf which extends the length of the calcaneum.

In *Periptychus*, a large anterior plantar tubercle is positioned on the anteroplantar border of the calcaneum for the attachment of the plantar calcaneocuboid ligament. In anterior view, the plantar tubercle is dorsoplantarly compressed against the body of the calcaneum. A narrow, transverse sulcus separates the anterior border of the plantar tubercle from the cuboid facet. Laterally, the anterior plantar tubercle is delimited from the peroneal process by a deep sulcus for the passage of the abductor digiti minimi muscle. The plantar tubercle of *Periptychus* is not as dorsoplantarly deep as in *Protungulatum* and *Arctocyon*.

#### Cuboid

**Fig 46:**
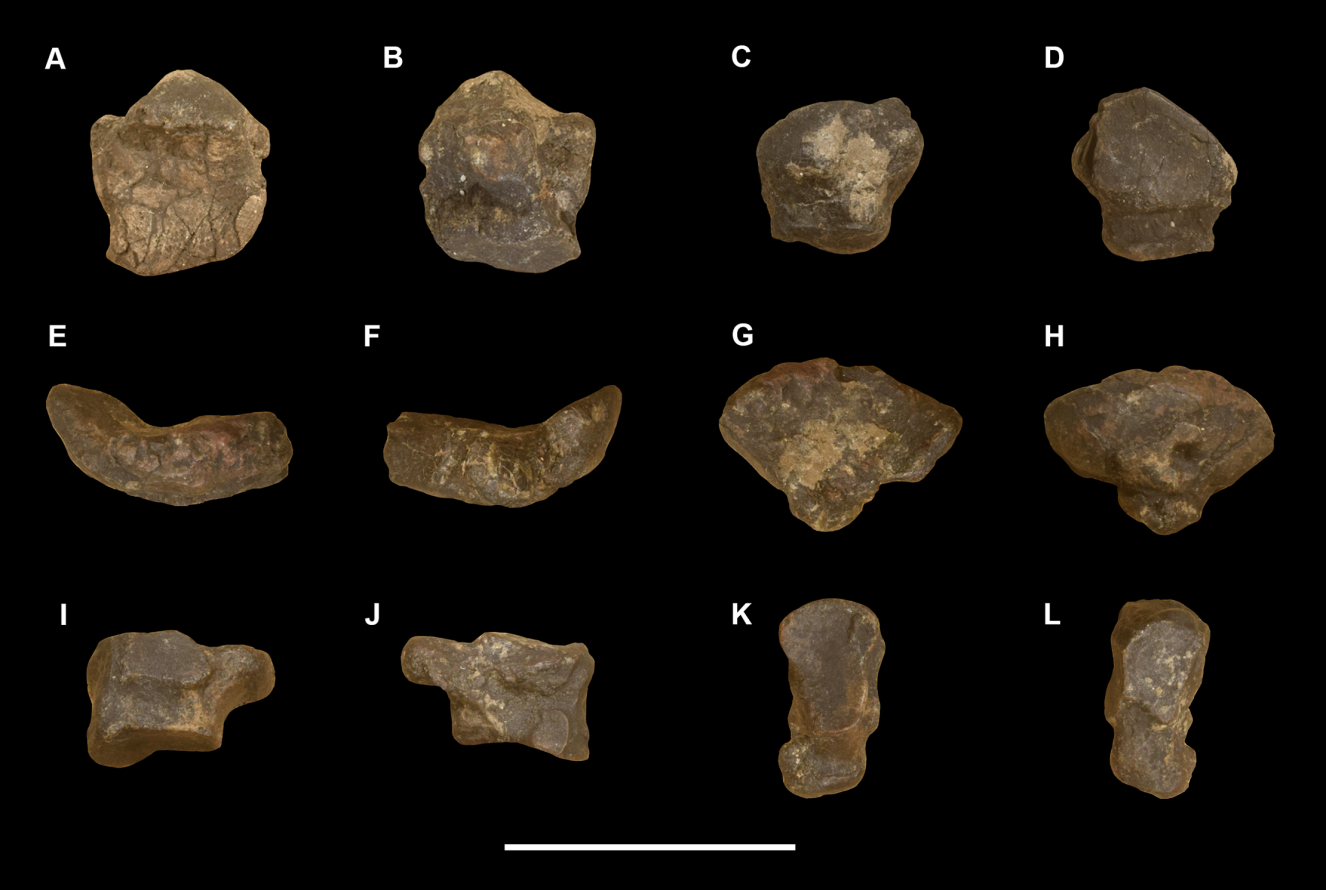
Tarsal elements of *Periptychus carinidens* (AMNH 3636). A-D: left cuboid: (A) dorsal view; (B) plantar view; (C) proximal view; (D) distal view. E-H: left navicular: (E) dorsal view; (F) plantar view; (G) proximal view; (H) distal view. I-L: left ectocuneiform: (I) medial view; (J) lateral view; (K) proximal view; (L) distal view. Scale bar: 30mm.

The cuboid of *Periptychus* is known from only two specimens: AMNH 17075 (Fig 41) and 3636 (Fig 46). AMNH 3636 is more intact than 17075, which is mounted in articulation, obscuring the plantar and articular surfaces from view. Neither specimen, however, preserves the entire cuboid.

The cuboid of *Periptychus* is quadrate and massively proportioned. In dorsal aspect, the mediolateral width of the cuboid is subequal to its anteroposterior depth, giving it a broad (rather than elongate) profile. The proportions of the cuboid of *Periptychus* are broadly similar to those of *Ectoconus* (in which the cuboid is mediolaterally broader than anteroposteriorly long), both of which feature a more massive construction than that exhibited by *Arctocyon* and *Claenodon* (where the cuboid is more anteroposteriorly elongate than mediolaterally broad).

Proximally, the cuboid of *Periptychus* articulates with the calcaneum and the head of the astragalus in an alternating sequence. The calcaneal facet accounts for approximately 60% of the proximal surface of the cuboid and forms a large convex facet. In proximal view, the profile of the facet is somewhat irregular and is not well preserved in either specimen observed. There is a prominent apex towards the medial side of the facet, with a large lateral flank, which is well expanded onto the dorsal surface of the cuboid. The morphology of the cuboid calcaneal facet of *Periptychus* differs considerably from that seen in *Arctocyon*, where the facet is much less convex and is not as dorsally expanded, with the articular surface facing proximolaterally. *Claenodon* exhibits a broadly similar morphology to *Arctocyon*, but possesses a larger dorsal expansion of the calcaneal facet, albeit not as expanded as that of *Periptychus*. The condition in *Periptychus* is generally most like *Ectoconus* in terms of overall proportions, but *Ectoconus* does not exhibit a large dorsal expansion of the cuboid facet.

Medially, the calcaneal facet of *Periptychus* is poorly demarcated from the astragalar facet. The astragalar facet is relatively large and accounts for the remaining 40% of the proximal surface of the cuboid. The surface of the facet is strongly concave, with well-defined proximal and medial surfaces. In *Periptychus*, the contact between the cuboid and astragalus appears to be more extensive than that in *Ectoconus*, with the astragalar facet accounting for approximately 20% of the proximal surface of the cuboid, forming a smaller, proximomedially facing, shallowly concave facet.

The medial surface of the cuboid of *Periptychus* features two facets: a proximal facet that articulates with the navicular and a distal facet that articulates with the ectocuneiform. Note that the navicular and ectocuneiform facets are poorly preserved on the specimen observed (AMNH 3636), so the following observations are somewhat tentative. The navicular facet is proximodistally short and slightly convex along its dorsoplantar and proximodistal planes. In medial aspect, the facet is orientated at an oblique (dorsoproximal to plantodistal) angle relative to the dorsoplantar axis of the cuboid. The proximal border of the facet borders the astragalar facet and the distal border appears to border the ectocuneiform facet along its entire dorsoplantar length. This is broadly like the condition in *Ectoconus*, although in *Ectoconus* the facet is not set an oblique angle relative to the dorsoplantar axis of the cuboid. The morphology exhibited by *Periptychus* and *Ectoconus* differs from the condition in *Arctocyon*, where the navicular facet is orientated at a proximodistal to dorsoproximal angle and the distal border of the facet borders the ectocuneiform facet along its entire dorsoplantar length.

In *Periptychus*, the ectocuneiform facet on the cuboid is slightly larger than the navicular facet. It is dorsoplantarly more elongate than the navicular facet, but the surface is flat and not as convex. In medial aspect, the facet is parallel to the navicular facet and set at a dorsoproximal-plantodistal oblique angle relative to the dorsoplantar axis of the cuboid. The navicular facet of *Periptychus* appears to be proportionally expanded (both dorsoplantarly and proximodistally) on the medial surface of the cuboid in comparison to the navicular facet of *Arctocyon*, which is more dorsoplantarly elongate. Distal to the two medial facets for the navicular and ectocuneiform, the medial surface of the cuboid of *Periptychus* is slightly excavated to form a dorsoplantar sulcus.

The plantar surface of the cuboid of *Periptychus* features a mediolaterally broad, hook-shaped plantar process, which overhangs a deep transverse sulcus for the tendon of the peroneus longus. The sulcus continues onto the lateral face of the cuboid. The anterior surface of the cuboid is formed by a large, concave facet that articulates with metatarsals IV and V. The border between the individual facets for the metatarsals is poorly demarcated. The facet is subtriangular in profile, broadest dorsally, and tapers to a round apex plantarly.

#### Navicular

The following description is based on AMNH 3636 (Fig 46) and 17075 (Fig 41), both of which include a complete navicular for *Periptychus*. The navicular articulates proximally with the astragalus, distally with the three cuneiform bones, and laterally with the cuboid. Furthermore, a possible medial articulation with an eighth tarsal ossicle, the so called ‘tibiale’, will be discussed. There is no contact between the navicular and calcaneum.

In dorsal aspect, the navicular is anteroposteriorly shallow and forms a partial cup around the distal portion of the head of the astragalus. *Periptychus* (and *Ectoconus*) possesses an anteroposteriorly shallower navicular in comparison to *Arctocyon* and *Claenodon*, but not as shortened as the condition in *Pantolambda*. In quantitative terms, the lateral edge of the navicular in *Periptychus*, *Ectoconus*, *Arctocyon* and *Claenodon* equates to approximately half the anteroposterior length of the cuboid (note that in the periptychids the transverse tarsal elements are proportionally shorter in relation to the other tarsal elements when compared to the arctocyonid taxa). In contrast to this, in *Pantolambda* the navicular is distinctly thin and is equivalent to less than one quarter of the anteroposterior length of the cuboid.

In *Periptychus*, the medial process of the navicular is reduced, leaving the medial surface of the astragalar head exposed when the pes is held in the neutral position. The medial process of *Periptychus* is highly reduced in comparison to *Ectoconus* and to a lesser extent *Arctocyon*, where the process extends proximally to more completely cup the astragalar head. The proportions of the navicular of *Periptychus* more closely resemble the somewhat reduced condition exhibited by *Claenodon*, which possesses a smaller medial process than *Arctocyon*. The proportions of the proximal navicular surface of *Periptychus* are somewhat similar to *Pantolambda*; however, in *Pantolambda* the medial process of the navicular is a separate and unfused bone [5]. Matthew goes further to equate the unfused medial process of the navicular with the ‘tibiale’. However, a ‘tibiale’ has been found in taxa which also possess a medial process, for example *Claenodon* [107].

In proximal aspect, the astragalar facet of *Periptychus* is mediolaterally broader than dorsoplantarly deep. The highly reduced medial process of *Periptychus* is not well distinguished from the distal astragalar facet, whereas in *Arctocyon*, and to a lesser extent in *Ectoconus*, a narrow ‘neck’ separates the proximal and medial facets. The proximal navicular profile exhibited by *Periptychus* is dorsopalmarly reduced in comparison to *Arctocyon*, where the astragalar facet is more rounded in profile. The plantar tuberosity of *Periptychus* is mediolaterally robust, but is not as ventrally prominent as that of *Arctocyon*. The distal surface of the plantar tuberosity of *Periptychus* is deeply grooved for the passage of the tendon of the peroneus longus.

The medial edge of the navicular possesses a small, trapezoidal shaped cuboid facet, which articulates with the navicular facet on the medial surface of the cuboid. The articular surface of the facet is very slightly concave along its dorsoplantar axis. The navicular cuboid facet of *Periptychus* differs from the condition in *Arctocyon*, where the facet is dorsoplantarly sigmoidal, forming a tight articulation with the cuboid; a similar condition is observed in *Claenodon* [107]. In proximal aspect, the navicular and cuboid of *Periptychus* form a continuous surface with a sigmoidal profile, with both the navicular and cuboid forming a tight articulation around the lateral portion of the head of the astragalus.

The distal surface of the body of the navicular is convex and subdivided into three smooth surfaces, which articulate with the cuneiforms. The facet for the ectocuneiform is mediolaterally broad. The articular surface is slightly concave and orientated distolaterally. The facet for the mesocuneiform is roughly subequal in mediolateral width, but dorsoplantarly deeper. Its articular surface is convex and faces distally. The ectocuneiform facet is the smallest of the three cuneiform facets on the navicular. The articular surface is convex, faces distomedially and extends on to the medial process of the navicular. The extension of the ectocuneiform facet onto the medial navicular process in *Periptychus* differs from the condition in both *Ectoconus* and *Arctocyon*, where the ectocuneiform facet is restricted to the body of the navicular. The extension of the ectocuneiform facet onto the medial process of the navicular constitutes a ‘well-consolidated’ navicular, as described by Matthew ([5] p.143).

#### Tibiale

The tibiale is a sesamoid bone of the navicular, embedded within the tendon of the tibialis posterior muscle [105]. Note that the tibiale, as thus defined, is not homologous with the reptilian tibiale. The tibiale is known for *Periptychus* based on AMNH 17075 (Fig 41). Matthew figured the tibiale on AMNH 17075, but did not make any reference to it within the text [5]. The tibiale of AMNH 17075 is a small, flat, thin piece of bone positioned proximal to the medial process of the navicular, covering the exposed medial surface of the astragalar head which is not covered by the navicular.

There are several considerations of the tarsal anatomy of *Periptychus* that help elucidate the anatomy and function of the tibiale sesamoid. Previous workers have inferred the existence of a tibiale based on the presence of a medial facet on the astragalar head, as exhibited by *Claenodon*, a taxon for which the tibiale is putatively known [5,7,59,107]. Such a facet is present and well developed in *Periptychus*, extending distally onto the astragalar neck. A similar facet is seen in *Ectoconus*, *Mithrandir*, *Protungulatum*, *Arctocyon* and *Pantolambda*.

However, there are several other anatomical details to consider when interpreting this facet. First, the medial astragalar facet is associated with the plantar calcaeneonavicular ligament (the ‘spring ligament’) in plesiadapid primates [71]. The plantar calcaneonavicular ligament attaches the sustentaculum to the navicular and serves to support the head of the astragalus against the navicular. It is not explicitly clear how such a configuration would affect the positioning and function of a tibiale. More problematically, this also raises the possibility that a facet in this position is not always indicative of a bony tibiale, but may in some cases be a ligament pit.

Secondly, several of the taxa with an observed medial astragalar facet that have been proposed to have possessed a tibiale (namely *Ectoconus* [5] and *Mithrandir* [48]) also possess an enlarged medial process of the navicular, which would completely omit a tibiale from contacting the head of the astragalus (dismissing the tarsal configuration proposed above). Where a tibiale is present alongside an enlarged medial navicular process, the primary function of the tibiale could be to solely increase the moment arm of the tibialis posterior. Both *Periptychus* and *Claenodon* possess a reduced medial navicular process, which would allow for the articulation of the tibiale with the astragalus. Therefore, it is plausible that both a tibiale and an expanded medial navicular process represent different conditions converging on the same function: to support the astragalar head during inversion. It is also likely that the unfused medial navicular process described for *Pantolambda* [5] is actually a tibiale.

#### Ectocuneiform

The ectocuneiform of *Periptychus* is the largest of the three tarsal cuneiforms. The following description is based on AMNH 3636 (Fig 46) and 17075 (Fig 41), both of which include a complete ectocuneiform. The body of the ectocuneiform is a robust, dorsoplantarly elongate, quadrate bone with a prominent plantar process. Proximally the ectocuneiform articulates with the navicular, laterally it articulates with the cuboid, medially with the mesocuneiform and distally with the second and third metatarsals. The ectocuneiform of *Periptychus* is not as proportionally robust as that of *Ectoconus*, in which the bone is proximodistally shorter but mediolaterally broader.

The proximal surface of the ectocuneiform of *Periptychus* possesses a weakly convex, subrectangular navicular facet. The convex morphology of this facet in *Periptychus* differs from the condition in *Ectoconus*, where the articular surface is near flat, and *Arctocyon* where the articular surface is shallowly concave. A large hook-shaped plantar process dominates the ventral surface of the ectocuneiform of *Periptychus*, overhanging a transverse sulcus for the tendon of the peroneus longus distally. The lateral surface of the ectocuneiform of *Periptychus* features a dorsoplantarly elongate, rectangular cuboid facet. The distal portion of the lateral surface of the ectocuneiform is excavated to form a dorsoplantar sulcus, which corresponds to a similar morphology on the cuboid.

The medial surface of the ectocuneiform features a proximal facet for the mesocuneiform. This facet is dorsoplantarly elongate and trapezoidal in shape. A second, smaller, subquadrate facet is positioned on the dorsal-most edge of the distal half of the medial surface of the ectocuneiform. This facet is shallowly concave and articulated with the proximal epiphysis of the second metatarsal. A second facet for metatarsal II extends plantarly from the first and is not as clearly delimited from the body of the ectocuneiform. The condition in *Periptychus* is broadly similar to that of *Ectoconus*, as in both taxa the facets on the ectocuneiform for the second metatarsal are in close proximity with one another, forming a near continuous surface. This condition differs from that seen in *Arctocyon*, in which the two facets are clearly delimited from one another. The distal surface of the ectocuneiform of *Periptychus* features a large, subtriangular concave facet for the proximal epiphysis of the third metatarsal.

#### Mesocuneiform

The mesocuneiform of *Periptychus* is known from AMNH 17075; the specimen is mounted in articulation, limiting observation of this element to its dorsal surface only (Fig 41).

The mesocuneiform is the smallest of the cuneiforms and the smallest bone in the tarsus. Proximally it articulates with the navicular, laterally with the ectocuneiform, medially with the entocuneiform and distally with the second metatarsal. In dorsal aspect, the mesocuneiform is quadrate in profile and proximodistally shortened relative to the ectocuneiform and entocuneiform, so that the second metatarsal extends proximally into the tarsal region, with its proximal epiphysis buttressed by the ectocuneiform and entocuneiform.

#### Entocuneiform

This entocuneiform of *Periptychus* is only known from AMNH 17075, and like the mesocuneiform, can only be observed in dorsal view (Fig 41). The entocuneiform is mediolaterally broad and proximodistally long, but dorsoplantarly shallow. Proximally the entocuneiform exhibits a relatively mediolaterally broad contact with the navicular, so that the entocuneiform contacts the medial process of navicular unlike the condition in *Ectoconus*. The proximal border of the entocuneiform remains distal to the proximal border of the navicular. On its medial edge, the entocuneiform articulates with the mesocuneiform proximally and the second metatarsal distally. Distally the entocuneiform exhibits a mediolaterally broad, but dorsoplantarly shallow contact with the first metatarsal.

#### Metatarsals

The metatarsals of *Periptychus* are generally similar to the metacarpals: they are robust and mediolaterally broad, but remain well spaced from each other, and the proximal and distal epiphysis of each metatarsal is mediolaterally broad relative to the diaphysis, which is distinctly dorsoplantarly flattened (Fig 41). The second, third, fourth and fifth metatarsals are known from an associated, near complete pes of *Periptychus* (AMNH 17075). Because this specimen is mounted in articulation, observation of the plantar surface is not possible. The third metatarsal is the longest, the second metatarsal is 12% shorter than the third, the fourth metatarsal is slightly shorter than the third (4%) and the fifth is considerably reduced, 23% shorter than the fourth metacarpal. Both *Ectoconus* and *Pantolambda* show a similar trend, with the third metatarsal being the longest and the subsequent metatarsals becoming shorter. In *Ectoconus*, the second metatarsal is 16% shorter than the third, the fourth metatarsal is 2% shorter than the third metatarsal and the fifth metatarsal is 16% shorter than the fourth. In *Pantolambda* the second metatarsal is 9% shorter than the third, the fourth metatarsal is 3% shorter than the third metatarsal and the fifth metatarsal is 23% shorter than the fourth.

The robusticity of the metatarsals can be quantified using the ratio of the mediolateral width of the distal epiphysis of the third metatarsal divided by its proximodistal length [69]. In *Periptychus* (AMNH 17075), the ratio is 0.39; this is slightly higher than *Pantolambda* (0.34, AMNH 16663) and much higher than *Ectoconus* (0.29, AMNH 16500); *Arctocyon* (0.27, MNHN.F.CR42), and *Claenodon* (0.27, AMNH 3268).

The relative robusticity of the metatarsals of *Periptychus*, *Ectoconus* and *Pantolambda* do not fit the same trend seen with the metacarpals. The third metacarpal of *Periptychus* is proportionally as robust as *Pantolambda*, but less robust than *Ectoconus*. Further to this, the third metatarsal of *Periptychus* is longer than the associated third metacarpal (based on AMNH 17075). Both *Ectoconus* and *Pantolambda* exhibit similar metatarsal/metacarpal proportions, whereby the third metatarsal is longer than its associated third metacarpal (based on AMNH 16500 and 16663 respectively). The metatarsals of *Periptychus* are disproportionately robust in comparison to the metacarpals, whereas, the metacarpals of *Ectoconus* are disproportionately robust in comparison to the metatarsals.

The second metatarsal of *Periptychus* is broadly like the third in its overall morphology, albeit slightly shorter in length. Proximally it articulates with all three cuneiform bones, with a larger proximal facet contacting the mesocuneiform and smaller medial and lateral facets contacting the entocuneiform and ectocuneiform, respectively. The diaphysis is not as dorsopalmarly flattened as the third metacarpal. The distal epiphysis is broadly similar to that of the third metatarsal; however, the distal articular surface is asymmetrical, rather than symmetrical, due to the reduction of its medial portion.

The third metatarsal is asymmetrical along its proximodistal long axis due to the expansion of the proximolateral portion of the proximal epiphysis and medial expansion of the distal epiphysis. Proximally, the third metacarpal articulates with the ectocuneiform via a large, convex facet. The transverse axis of the facet is set at an oblique angle, so that lateral surface of the facet is positioned proximally relative to the medial surface. The lateral surface of the proximal epiphysis exhibits a large flat articular facet which abuts the fourth metatarsal, but does not overlap it. The medial surface of the proximal epiphysis barely contacts the proximal epiphysis of the second metatarsal, due to the proximal placement of the second metatarsal and the proximodistal shortening of the mesocuneiform. The proximal epiphysis of the third metatarsal is dorsoplantarly deeper than the distal epiphysis, but not as mediolaterally broad. The dorsal surface of the proximal epiphysis is lightly damaged, but there is little evidence of the two tuberosities for the insertion of the tarso-metatarsal ligaments, like those seen in *Ectoconus*. Such tuberosities are also absent in *Claenodon*.

The diaphysis of the third metatarsal of *Periptychus* is mediolaterally broad and dorsopalmarly compressed, with a smooth surface. The broad dorsal surface provided a large attachment area for the dorsal interossei. The mediolateral width of the diaphysis is near constant along its length, with some mediolateral broadening towards the distal epiphysis.

The distal epiphysis is mediolaterally expanded so that it is broader than the proximal epiphysis, but not as dorsopalmarly deep. The distal surface of the bone forms a saddle-shaped articular surface with a dorsoplantar convexity that is broadly symmetrical. There is little evidence of a median keel on the dorsal articular surface, which is likely restricted to the plantar surface. The medial edge of the distal epiphysis is expanded and marked by a small, but prominent, tuberosity. A similar medial protuberance is present in *Ectoconus*, but is not as developed as in *Periptychus*; there is little evidence of a medial protuberance in *Claenodon*. In *Periptychus*, the distal articular surface extends well onto the dorsal surface. The proximal border of the distal articular surface is not demarcated by a fossa like that seen in the metacarpals of *Periptychus*, and both the metacarpals and metatarsals of *Claenodon* and *Pantolambda*.

The fourth metatarsal is generally like the second in its overall morphology, and in how it differs from the third metatarsal. Proximally, it articulates with the medial half of the distal surface of the cuboid. Medially, it contacts the third metatarsal via a small medially orientated facet, and laterally it contacts the fifth metatarsal via a laterally orientated facet. There is no evidence of overlap between the third, fourth and fifth metatarsals. The distal epiphysis displays the reverse morphology of the second metatarsal. The distal articular surface is asymmetrical due to the reduction of its lateral portion, to mirror the morphology of the second metatarsal.

The fifth metatarsal exhibits a somewhat different morphology to the second, third and fourth due to the expansion of its proximal epiphysis. Proximally it articulates with the lateral half of the distal surface of the cuboid, projecting distolaterally from the body of the pes. It does not contact the lateral surface of the cuboid, differentiating it from *Claenodon*. A large lateral tuberosity projects from the proximal epiphysis and provided a large grooved insertion site for the peroneus brevis. A large, medial facet on the proximal epiphysis contacts the fourth metatarsal. The distal epiphysis is mediolaterally broad, as is the case with the other metatarsals; however, the articular surface is not as dorsally expanded and appears to be hemispherically convex to surround the head of the bone rather than forming a dorsoplantarly convex saddle-shaped articular area.

#### Tarsal phalanges

The tarsal phalanges of *Periptychus* are essentially morphologically identical to the manual phalanges, albeit somewhat larger. The robustness of the tarsal phalanges can be quantified using the ratio of the mediolateral width of the proximal epiphysis of the fourth phalanx divided by its proximodistal length [69]. Ideally, the ratio should be based on the third proximal phalanx, but only incomplete specimens are not known for *Periptychus* so we are using the fourth proximal phalanx, as it closely approximates the third in size and is known for all the comparison taxa. The ratio for *Periptychus* (based on AMNH 17075) is 0.76 compared to 0.66 for *Ectoconus* (AMNH 16500), 0.75 for *Pantolambda* (AMNH 16663) and 0.51 for *Claenodon* (AMNH 16543). As such, we can infer that the proximal phalanges of *Periptychus* are only slightly more robust than those of *Pantolambda* (1%), but 13% more robust than those of *Ectoconus* and 33% more robust than those of *Claenodon*.

## Conclusion

New specimens of the Paleocene periptychid, *Periptychus carinidens*, are described here. These include some of the most complete specimens known for the species and provide new anatomical information on this abundant taxon, which was among the first eutherian mammals to evolve moderately large body size and distinct adaptations (particularly related to diet) after the end-Cretaceous mass extinction. These specimens also provide new data with which to examine periptychid and Paleocene mammal phylogeny and paleobiology.

*Periptychus* is an unusual taxon in that it unites a suite of dental, cranial and postcranial specializations with an otherwise relatively generalized skeleton. The overall shape of the skull of *Periptychus* is broadly concurrent with the inferred plesiomorphic eutherian condition [54,69,108], albeit more robust in its overall construction. Derived dental specializations included crenulated enamel, enlarged upper and lower premolars with a tall centralised paracone/protoconid and lingual shoulder. The relatively small canines and broad bunodont postcanine teeth, combined with a greatly expanded mandibular angle, raised mandibular condyle and broad zygomatic arches indicate *Periptychus* was herbivorous. The enlarged premolars are highly suggestive of an animal adapted towards durophagy and adept at crushing tough foodstuffs.

The postcranial skeleton of *Periptychus* is that of a robust, stout-limbed animal that was incipiently mediportal (adapted to moving slowly over land but also having some characteristics conducive to quick movements when needed) and adopted a plantigrade stance. Features of note in the forelimb of *Periptychus* include: a shortened humerus relative to the ulna and radius; a hemispherical humeral head with large but low tuberosities; a broad and elongate deltopectoral region; a reduced insertion for teres major on the humerus; expanded lateral and medial epicondyles; an open humeroradial joint, a relatively straight ulna with little posterior bowing of the diaphysis or anterior bowing of the olecranon process; a broad carpus with enlarged centrale; and relatively short digital bones terminating in hoof-like unguals.

Key features of the hindlimb of *Periptychus* include: a relatively unspecialized innominate with a widely open acetabulum; robust femoral trochanters including a third trochanter; the greater trochanter of the femur is tall but does not extend beyond the femoral head; a dorsoplantarly compressed astragalus which in articulation is wedged between the tibia and fibula permitting the fibula to contact the calcaneum; and a retained tibiale.

In describing the anatomy of *Periptychus*, it is apparent that it closely resembles other medium-sized Paleocene mammals, given their array of shared ‘primitive’ characteristics compared to the vast range of morphologies and adaptations exhibited by extant mammals. Consequently, the task of distinguishing between ‘primitive’ Paleocene mammals gets distorted by our bias from observing features in extant mammals, which often serve to define what a constitutes an animal adapted for a certain lifestyle. The skeleton of *Periptychus* bears numerous resemblances to the other Paleocene taxa observed during the course of this study; however, there are subtle distinctions between the Paleocene taxa indicative of adaptations that are not easily comparable to extant mammals. This suggests that, far from being just a generalised ancestral stock for extant orders, Paleocene mammals were experimenting with their own unique morphologies.

**Fig 47:**
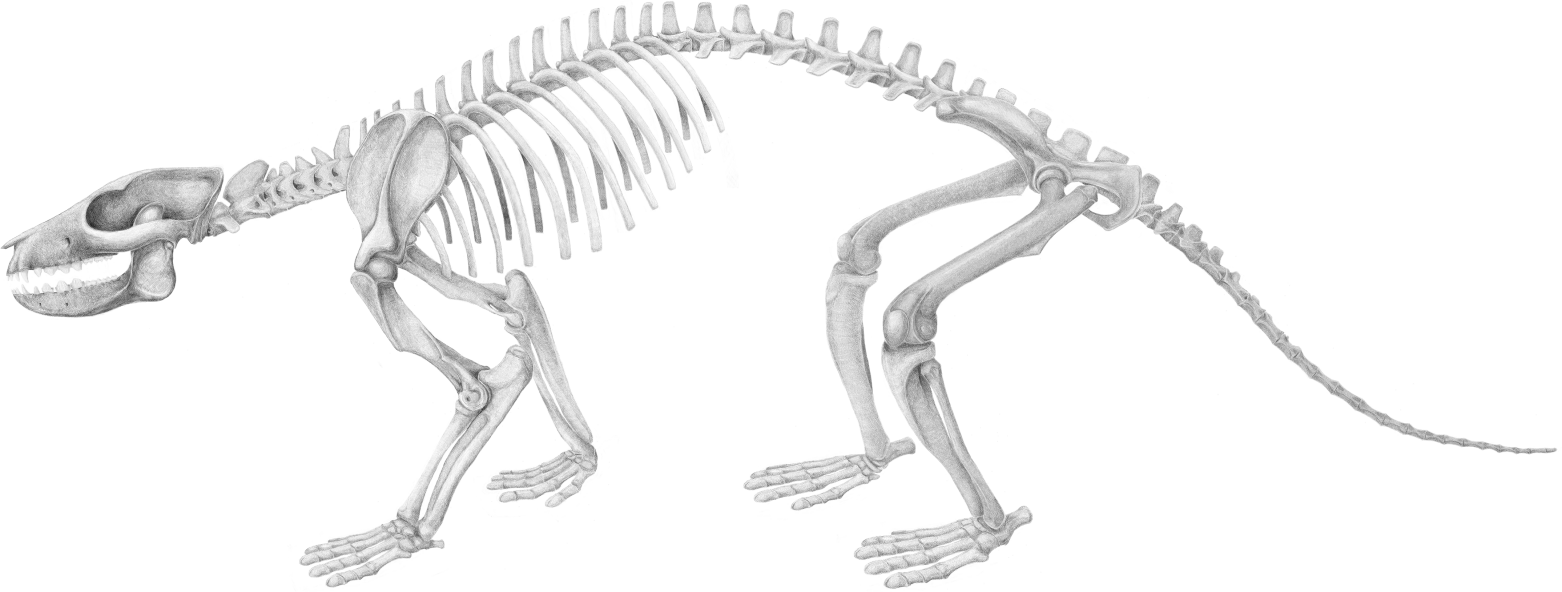
Skeletal reconstruction of *Periptychus carinidens*.

To this end, how can we describe *Periptychus*? In the broadest sense, it is a medium-sized obligate terrestrial generalist, albeit a versatile one, adept at moving through and over obstacles on the forest floor with adaptations of the limbs which do not preclude some scansorial and fossorial ability, and a simple but effectively modified dentition adapted to a plant-based durophagus diet (Fig 47). During this study, we studied numerous other medium-sized Paleocene mammals and could not help postulating on their paleobiology, albeit not to the same depth as *Periptychus*. In relation to *Periptychus*, *Ectoconus*, a considerably larger (~100Kg body mass) appears to be more fossorially adapted with dental adaptations indicative of an herbivorous diet. *Arctocyon* was likely more scansorial with dental adaptations towards an omnivorous to carnivorous diet [52]. *Pantolambda* exhibits some traits which suggest it to be more fossorially adapted than *Periptychus* but not to the same degree as *Ectoconus*, but it also lacks several key fossorial adaptions (no indication of a fossorially adapted triceps, and relatively reduced manual elements). Further more detailed study is required, but during the course of study our observations of *Pantolambda* have led us to hypothesise it might have been semi-aquatic, an ecology which has also been inferred for some larger, Eocene pantodont species [3].

From a wider perspective, the anatomy of *Periptychus* is broadly concurrent with what has been inferred as representative as the primitive eutherian condition. The dentition retains a primitive formula, occlusal pattern and cusp configuration albeit with numerous, modifications, but these are simple in their alteration and easily discerned. The skull is largely like other contemporaneous Paleogene taxa, although more robust in construction. The basicranial anatomy provides some interesting information, with *Periptychus* exhibiting a petrosal that is generally comparable the anatomy observed in taxa such as *Pantolambda*, *Deltatherium* and *Eoconodon*, but is somewhat divergent from the morphology exhibited by *Protungulatum* and *Arctocyon*, potentially suggesting a deeper division between periptychids and ‘arctocyonids’ than has previously hypothesised (see [3,6,9]).

Throughout its evolutionary history *Periptychus* was apparently a highly successful taxon as evidenced by its abundant dental fossil record, and was one of the few periptychids to persist through the Torrejonian and into the Tiffanian. Consider also, that while periptychids were abundant during the Puercan, they spent most of their evolutionary history exhibiting high turnover rates, which makes the persistence and widespread abundance of *Periptychus* even more notable. Consequently, *Periptychus*–and to a broader extent, periptychids–are prime exemplars by which to tackle the taxonomic and systematic conundrum that is ‘Condylarthra’.

## Supporting information

S1 Appendix *Periptychus carinidens* raw anatomical measurements. This file is formatted as an excel file and includes raw measurements for the *Periptychus* specimens described in this paper. Individual bones are listed in separate tabs.
(XLSX)
